# Enantioselective installation of adjacent tertiary benzylic stereocentres using lithiation–borylation–protodeboronation methodology. Application to the synthesis of bifluranol and fluorohexestrol†
†Electronic supplementary information (ESI) available: Detailed experimental procedures and spectroscopic data for all new compounds. X-Ray data analysis for compound **2**. See DOI: 10.1039/c4sc03901g


**DOI:** 10.1039/c4sc03901g

**Published:** 2015-04-28

**Authors:** Stefan Roesner, Daniel J. Blair, Varinder K. Aggarwal

**Affiliations:** a School of Chemistry , University of Bristol , Cantock's Close , Bristol , BS8 1TS , UK . Email: v.aggarwal@bristol.ac.uk ; Fax: +44 (0)117 925 1295 ; Tel: +44 (0)117 954 6315

## Abstract

1,2-Diaryl ethanes bearing 1,2-stereogenic centres show interesting biological activity but their stereocontrolled synthesis has not been reported forcing a reliance of methods involving diastereomer and enantiomer separation. We have found that this class of molecules can be prepared with very high stereocontrol using lithiation–borylation methodology. The reaction of an enantioenriched benzylic lithiated carbamate with an enantioenriched benzylic secondary pinacol boronic ester gave a tertiary boronic ester with complete diastereo- and enantiocontrol. It was essential to use MgBr_2_/MeOH after formation of the boronate complex, both to promote the 1,2-migration and to trap any lithiated carbamate/benzylic anion that formed from fragmentation of the ate complex, anions that would otherwise racemise and re-form the boronate complex eroding both er and dr of the product. When the benzylic lithiated carbamate and benzylic secondary pinacol boronic ester were too hindered, boronate complex did not even form. In these cases, it was found that the use of the less hindered neopentyl boronic esters enabled successful homologation to take place even for the most hindered reaction partners, with high stereocontrol and without the need for additives. Protodeboronation of the product boronic esters with TBAF gave the target 1,2-diaryl ethanes bearing 1,2-stereogenic centres. The methodology was applied to the stereocontrolled synthesis of bifluranol and fluorohexestrol in just 7 and 5 steps, respectively.

## Abstract 2

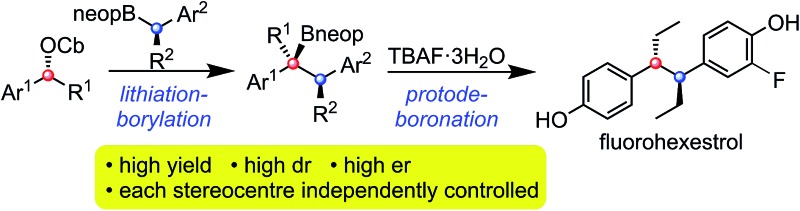
Highly hindered benzylic carbamates have been reacted with hindered boronic esters to give tertiary boronic esters with very high diastereo- and enantiocontrol and the methodology has been applied to otherwise difficult-to-access molecules.

## Introduction

**Fig. 1:**
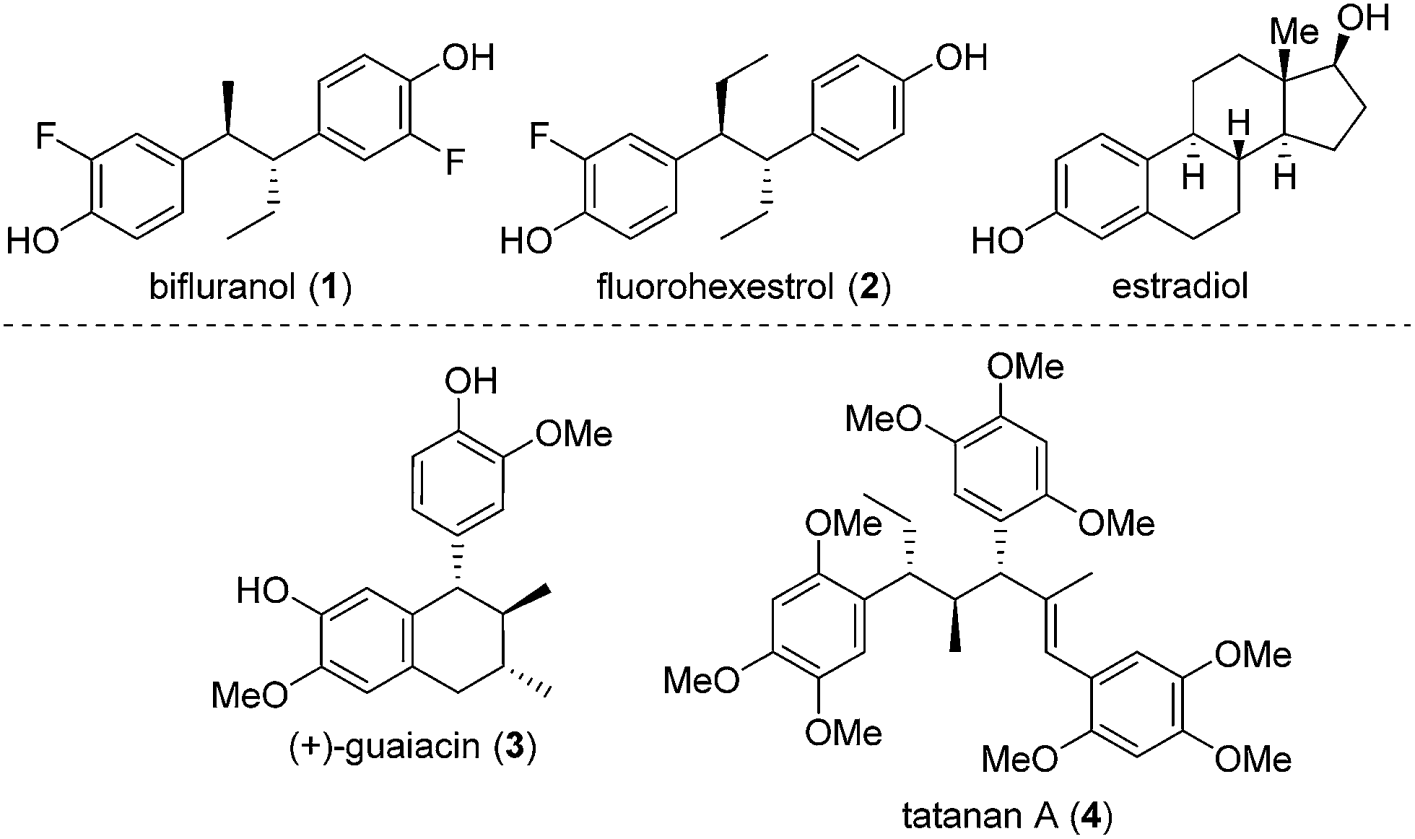
Representative biologically active molecules.

Numerous methods have been developed for acyclic stereocontrol, the most highly developed being the aldol reaction where high levels of 1,2- and 1,3-stereocontrol can be achieved.^1^ For molecules without hydroxyl functionality, 1,2- and 1,3-stereocontrol is much more difficult and general synthetic methods are sparse. This problem is highlighted in the synthesis of the antiandrogen, bifluranol^2^ (Prostarex, **1**), and the potential imaging agent, fluorohexestrol^3^**2** (used for the visualisation of human breast tumours), where neither relative nor absolute stereocontrol could be achieved.^4^ These rather unusual molecules bear a structural similarity to the hormone estradiol, accounting for their particular biological activity.^5^ Other challenging molecules bearing contiguous alkyl groups but devoid of other functionality include the lignin, (+)-guaiacin^6^ (**3**), and the potent glucokinase-activating agent, tatanan A^7^ (**4**) (Fig. 1).

**Scheme 1:**
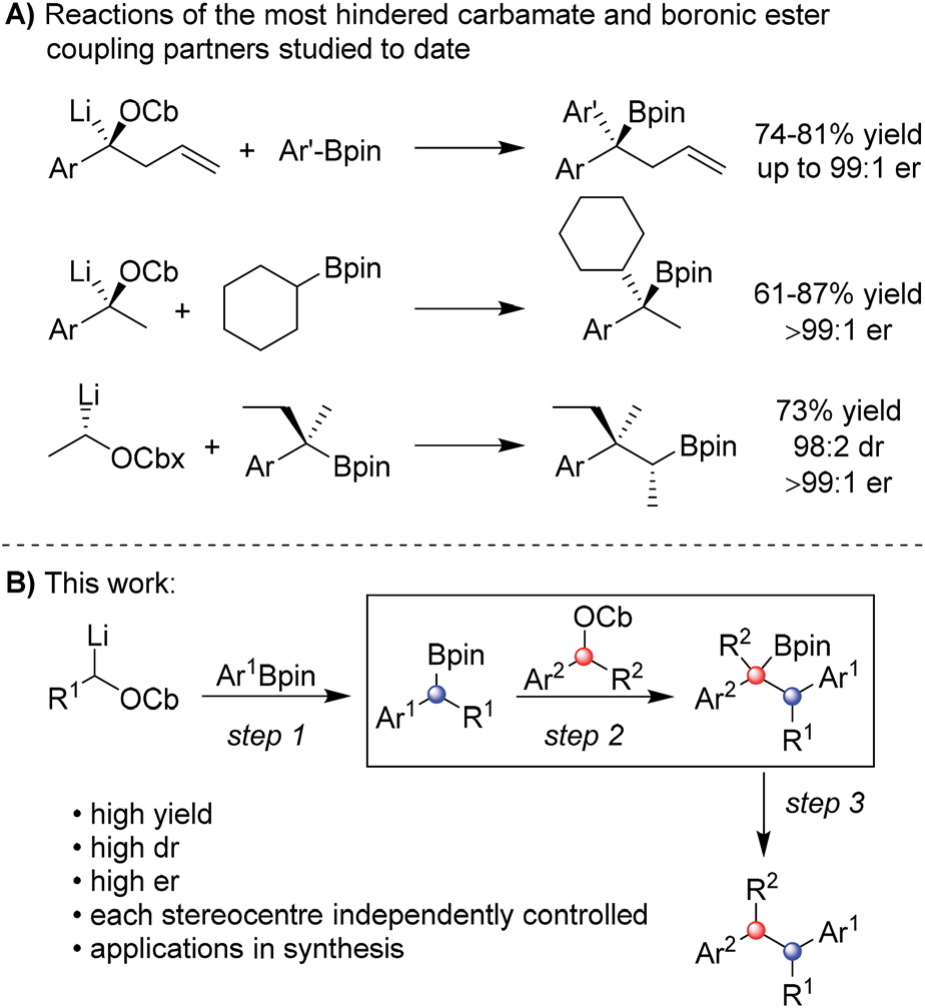
(A) Reactions of hindered carbamates with hindered boronic esters. (B) Proposed reactions of hindered carbamates with hindered boronic esters for the synthesis of contiguous chiral benzylic centres.

In recent years, we have developed lithiation–borylation methodology of primary^8^ and secondary^9^ carbamates for the stereocontrolled synthesis of secondary and tertiary boronic esters (Scheme 1A). The process is related to Blakemore's reactions of α-lithiated alkylchlorides^10^ and follows the fundamental work of Matteson on 1,2-metallate rearrangements of boronic esters.^11^ We considered the consecutive use of this reaction coupled with protodeboronation methodology^12^ for the synthesis of adjacent tertiary stereocentres (Scheme 1B). Furthermore, this strategy allows control of each stereocentre independently, thereby enabling the synthesis of any stereoisomer at will. Whilst key steps 1 and 3 had good literature precedent, step 2, the reaction of the secondary carbamate with a highly hindered boronic ester did not (Scheme 1B). Only reactions of unhindered secondary carbamates (R^1^ = Me) with moderately hindered boronic esters had been reported.9a,b,^13^ In this paper we show the limits of lithiation–borylation reactions and how, under the right conditions, the coupling of even highly hindered secondary carbamates with hindered boronic esters can be achieved with very high stereocontrol. We have also demonstrated its strategic use in the enantio- and diastereoselective synthesis of the antiandrogen, bifluranol (**1**), and the potential imaging agent, fluorohexestrol (**2**), validating the synthetic utility of this methodology.

## Results and discussion

### Effect of steric hindrance in the lithiation–borylation reaction

We began our studies with a systematic investigation of the effect of steric hindrance on the outcome of the lithiation–borylation reaction between a secondary racemic benzylic carbamate and a secondary (racemic) boronic ester (Table 1). Two representative secondary benzylic carbamates (**5** and **6**) and four representative secondary pinacol boronic esters (**7–10**) were chosen as substrates, each of increasing steric demand. The reactions were conducted under two sets of standard conditions: (i) conditions **A**: addition of the boronic ester to the lithiated carbamate at –78 °C followed by warming to room temperature for 16 h; (ii) conditions **B**: addition of the boronic ester to the lithiated carbamate at –78 °C followed after 2 h by addition of 1.3 equivalents of a solution of MgBr_2_ in MeOH and subsequent warming to room temperature for 16 h. Under conditions **A**, the reaction of the least sterically hindered carbamate **5** and boronic ester **7** gave tertiary boronic ester **13** in high yield (81%). Increasing the steric demand of the secondary boronic ester (conditions **A**) resulted in decreasing yields (**15**: 71%, **17**: 47%, **19**: 22%). Increasing the steric demand of the carbamate **6** had an even bigger impact on the yield of the product boronic esters, which were now only obtained in poor yields. In fact, in the case of the most sterically hindered substrates (carbamate **6** and boronic ester **10**) the boronate complex did not even form as determined by ^11^B NMR. Conditions **B**, which used MgBr_2_ in MeOH, were then explored for reactions involving pinacol boronic esters. This additive is known to have two distinct effects on lithiation–borylation reactions: (i) it increases the relative rate of 1,2-migration of the intermediate boronate complex over reversibility back to the starting components and (ii) any anions formed from reversibility are quenched, thus preventing re-addition.9*b* In almost all cases, the yield of the boronic ester was significantly increased with this additive, thus demonstrating its ability in promoting 1,2-migration over reversal. Without this additive, the lower yields are likely to be due to decomposition of the boronate complex back to the starting materials. Only in the case of the most hindered boronic ester **10** was no improvement observed (**20**).

**Table 1:** Investigation of the steric influence in the lithiation–borylation reaction^*a*^ ^,^^*b*^

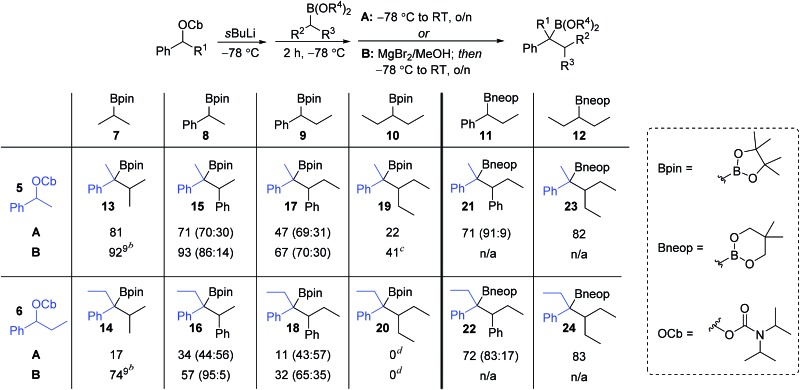

^*a*^Reaction conditions: **A**: (i) 1.3 equiv. *s*BuLi, Et_2_O (0.3 M), –78 °C, 1 h; (ii) 1.5 equiv. boronic ester in Et_2_O (1.0 M), –78 °C, 2 h; (iii) –78 °C → r.t., o/n; **B**: (i) and (ii) as **A**; (iii) 1.3 equiv. of MgBr_2_ in MeOH (1.0 M), then –78 °C → r.t., o/n.

^*b*^The ratio of *anti* to *syn* diastereoisomers determined by ^1^H NMR spectroscopy is shown in parentheses (for details see ESI).

^*c*^Yield determined by ^1^H NMR spectroscopy with internal standard.

^*d*^Traces of product could be detected by GC/MS but isolation was unsuccessful.

The low yields observed with the hindered secondary pinacol boronic esters **9** and **10** prompted us to explore the corresponding neopentyl boronic esters, **11** and **12**. In fact, these substrates worked very well and high yields were restored even with the highly hindered carbamates. Furthermore, with the neopentyl boronic esters no further additives were required to promote the 1,2-migration.^14^ For substrates that are prone to reversibility due to steric hindrance of the carbamate (*e.g.* secondary benzylic)9*b* or are electronically stabilised (*e.g.* propargylic)^15^ the use of less hindered diol esters is often beneficial, leading to both enhanced yields and selectivities.

An intriguing observation in this study was that the additive MgBr_2_/MeOH had a major impact on the diastereomeric ratio (*syn*/*anti* ratio) of the reaction. This is most dramatically illustrated in the reaction of carbamate **6** with boronic ester **8**: under conditions **A**, a ∼1 : 1 ratio of *syn* : *anti* isomers were formed, but in the presence of MgBr_2_/MeOH (conditions **B**) the *anti* diastereoisomer (*R*,*S*) of **16** was formed almost exclusively (95 : 5).

**Scheme 2:**
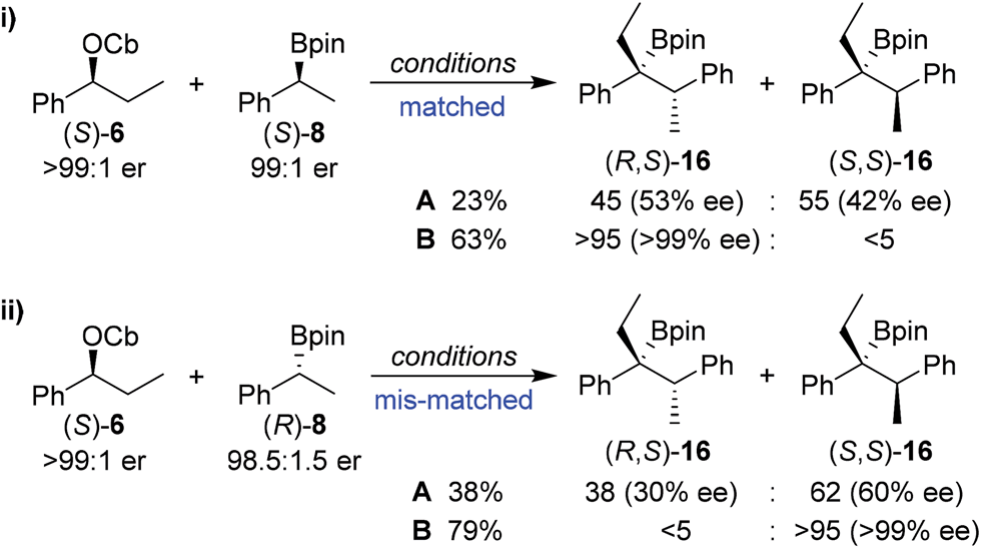
Mapping the fate of the stereocentres when using different reaction conditions. Reaction conditions: **A**: 1.3 equiv. *s*BuLi, Et_2_O (0.3 M), –78 °C, 1 h; 1.5 equiv. boronic ester in Et_2_O (1.0 M), –78 °C, 2 h; –78 °C → r.t., o/n; **B**: 1.3 equiv. *s*BuLi, Et_2_O (0.3 M), –78 °C, 1 h; 1.5 equiv. boronic ester in Et_2_O (1.0 M); 1.3 equiv. of MgBr_2_ in MeOH (1.0 M), then –78 °C → r.t., o/n.

**Scheme 3:**
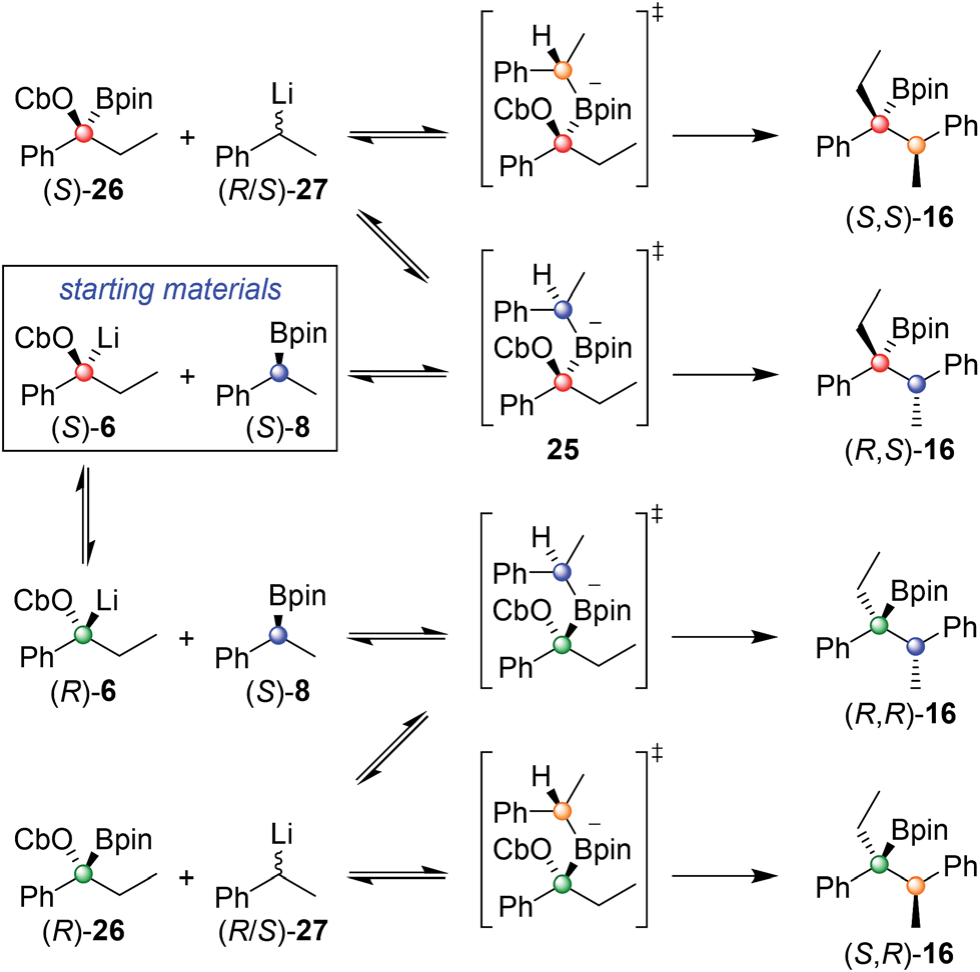
Origin of the four stereoisomers observed from the reaction of (*S*)-**6** with (*S*)-**8** under reaction conditions **A**.

In order to understand this reaction further, the fate of each stereocentre during the transformation was mapped out by carrying out the reaction with enantioenriched materials (Scheme 2). Without any additive (conditions **A**) the products from equations (i) and (ii) were obtained as a mixture of diastereoisomers and with substantial erosion in enantiomeric enrichment of both diastereoisomers. This means that erosion of both stereocentres occurred during the process of the reaction to a significant degree. From these experiments it is clear that reversibility is occurring but in different ways (Scheme 3). The intermediate boronate complex **25** has three competing fates: it can undergo (i) 1,2-metallate rearrangement to give boronic ester **16**, (ii) fragmentation back to the starting materials or (iii) fragmentation to boronic ester **26** and benzylic carbanion **27**. Racemisation of **6** and **27**, re-addition to the appropriate boronic ester, and 1,2-rearrangement then leads to a mixture of diastereoisomers with low enantiomeric excess (Scheme 3). Evidently, these reaction partners are most challenging since they are not only hindered and prone to reversibility but because they are both benzylic, they can fragment in either way to give stabilised benzylic anions.

In contrast, in the presence of MgBr_2_/MeOH, reaction of (*S*)-**6** with (*S*)-**8** or (*R*)-**8** gave the *anti* (*R*,*S*)-**16** (i) or the *syn* (*S*,*S*)-**16** (ii) isomer with high selectivity as a single enantiomer in high yield (Scheme 2). Under these conditions, boronate complex formation is non-reversible and is followed by stereospecific 1,2-metallate rearrangement.

Any boronate complex that does undergo fragmentation back to the starting materials is quenched by the MeOH and no longer participates in the reaction. The selectivity is therefore determined in the addition step leading to the boronate complex **25**. The high diastereoselectivity observed when (*rac*)-**6** was reacted with (*rac*)-**8** showed that there was a strong matched/mis-matched effect in operation, *i.e.* (*S*)-**6** reacted with (*S*)-**8** considerably faster than with (*R*)-**8** giving the *anti* (*R*,*S*) diastereoisomer preferentially (note, there is a change in priority of one of the centres). However, when using enantioenriched materials even in the mis-matched case [(*S*)-**6** with (*R*)-**8**; Scheme 2, equation ii], high yield was still obtained showing that the slower rate of formation of the boronate complex was not accompanied by undesired side reactions.

**Scheme 4:**
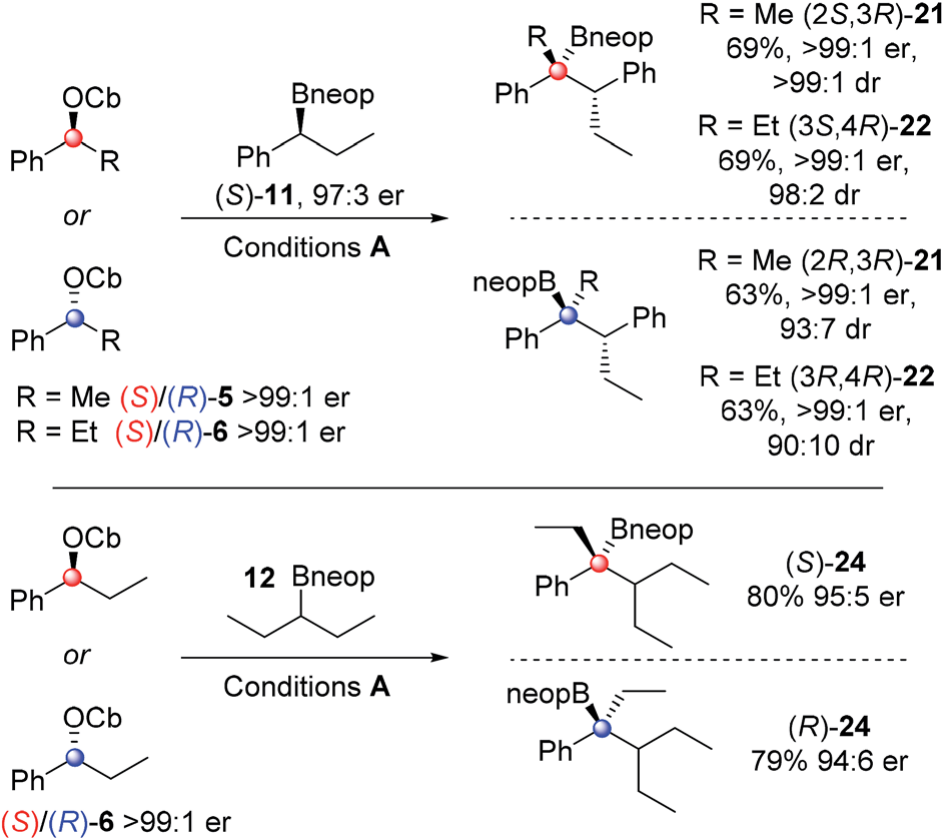
Scope of stereoselective synthesis of hindered tertiary neopentyl boronic esters. Reaction conditions: 1 equiv. carbamate, 1.3 equiv. *s*BuLi, Et_2_O (0.3 M), –78 °C, 1 h; 1.5 equiv. boronic ester in Et_2_O (1.0 M), 3 h; then –78 °C → r.t., o/n. The ratios of enantiomers and diastereoisomers were determined by chiral HPLC.

To explore the scope of the asymmetric reactions, four representative enantioenriched carbamates (*S*)-/(*R*)-**5**/**6** were reacted with two representative hindered neopentyl boronic esters (*S*)-**11** and **12** (Scheme 4). Neopentyl boronic esters were chosen because they gave higher yields than pinacol boronic esters (Table 1). In all cases, high diastereo- and enantio-selectivity was observed indicating that the reactions were essentially non-reversible. Since both diastereoisomers **21** and **22** were formed in similar yields and selectivities it shows that once again the reactions are dominated by reagent control. A small but detectable matched/mis-matched effect was observed since (2*S*,3*R*)-**21** was formed with slightly higher dr than (2*R*,3*R*)-**21** (>99 : 1 *vs.* 93 : 7). The low level of erosion of er in the case of the highly hindered substrate **24** (95 : 5 er) is most likely due to a small degree of reversibility in this case. Compounds **16**, **21**, **22** and **24** were all oxidised to the corresponding tertiary alcohols for ee determination.

### Enantioselective synthesis of bifluranol

**Scheme 5:**
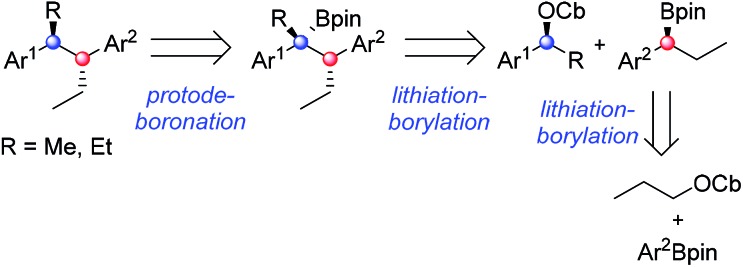
Retrosynthetic scheme for the synthesis of bifluranol **1** and fluorohexestrol **2**.

Having found conditions under which high diastereoselectivity could be achieved, we sought to apply this methodology to the enantioselective synthesis of bifluranol (**1**), an antiandrogen with the ability to treat benign prostate enlargement,^2^ and fluorohexestrol (**2**), a potential non-steroidal oestrogen receptor based imaging agent for the visualisation of human breast tumours.^3^ The retrosynthetic analysis of **1** and **2** is illustrated in Scheme 5. By using a convergent synthetic strategy, we proposed to build up both stereogenic centres by applying two consecutive lithiation–borylation reactions followed by protodeboronation.

**Scheme 6:**
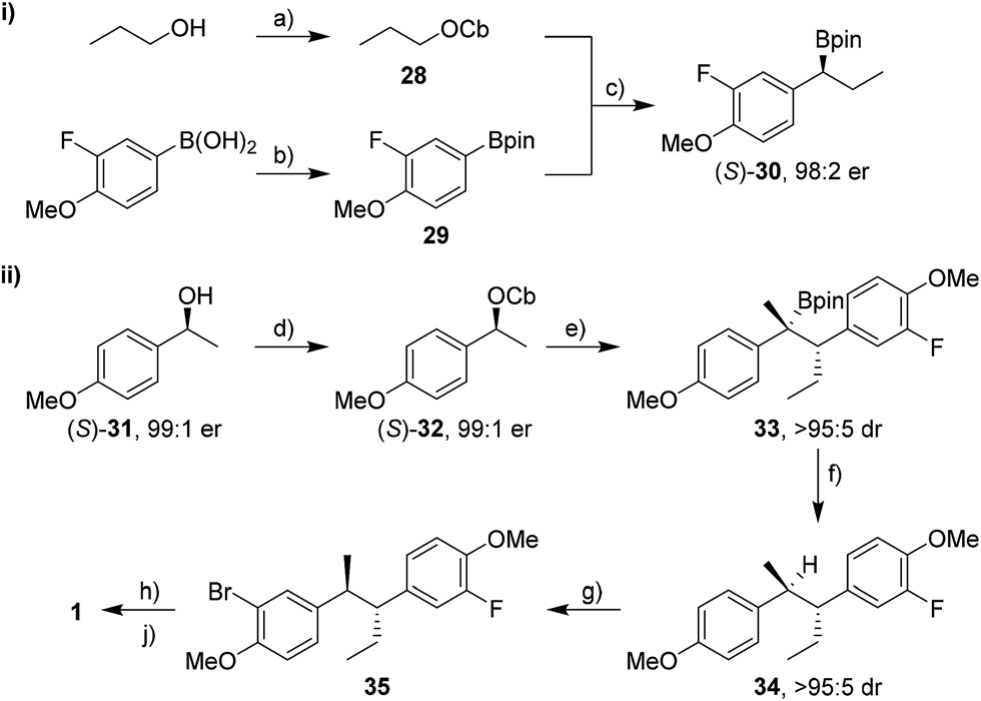
Synthesis of bifluranol **1**. Reagents and conditions: (a) CbCl (1.0 equiv.), Et_3_N (1.3 equiv.), *n*-propanol, sealed tube, μW, 150 °C, 1 h, 92% yield; (b) pinacol (1.0 equiv.), Et_2_O, r.t., 16 h; MgSO_4_ (3.0 equiv.), r.t., 2 h, quant.; (c) (+)-sparteine (1.3 equiv.), *s*BuLi (1.3 equiv.), Et_2_O, –78 °C, 5 h; **29** (1.5 equiv.), –78 °C, 2 h; r.t., Et_2_O → CHCl_3_; reflux, 15 h, 76% yield; (d) NaH (1.5 equiv.), THF, r.t., 75 min; CbCl (1.2 equiv.), THF, reflux, 24 h, >99% yield; (e) TMEDA (1.3 equiv.), *s*BuLi (1.3 equiv.), Et_2_O, –78 °C, 1 h; (*S*)-**30** (1.5 equiv.), –78 °C, 2 h; r.t., 14 h, 95% yield; (f) TBAF·3H_2_O (3.0 equiv.), toluene, reflux, 3 h, 99% yield; (g) NBS (1.1 equiv.), MeCN, r.t., 21 h, 94% yield; (h) *n*BuLi (1.3 equiv.), THF, –78 °C, 30 min; NFSI (1.2 equiv.), –78 °C, 2 h; (j) BBr_3_ (3.0 equiv.), CH_2_Cl_2_, –20 °C; 30 min; 4 °C, 16 h, 43% yield over 2 steps. CbCl = *N*,*N*-diisopropylcarbamoyl chloride, *s*BuLi = *sec*-butyllithium, TMEDA = *N*,*N*,*N*′,*N*′-tetramethylethylenediamine, TBAF·3H_2_O = tetrabutylammonium fluoride trihydrate, NBS = *N*-bromosuccinimide, *n*BuLi = *n*-butyllithium, THF = tetrahydrofuran, NFSI = *N*-fluorobenzenesulfonimide.

We began with the synthesis of bifluranol. Carbamate **28** was prepared from *n*-propanol and subsequent lithiation in the presence of (+)-sparteine^16^ followed by borylation with pinacol boronic ester **29** gave the secondary boronic ester **30** in 76% yield and with 98 : 2 er (Scheme 6-i). Aryl groups are not good migrating groups^17^ in lithiation–borylation reactions and often need assistance by either using MgBr_2_ in Et_2_O8*a* or solvent exchange.^18^ In this case we employed a solvent exchange from diethyl ether to CHCl_3_ to promote the 1,2-migration and thereby increase the yield of the reaction.

**Scheme 7:**
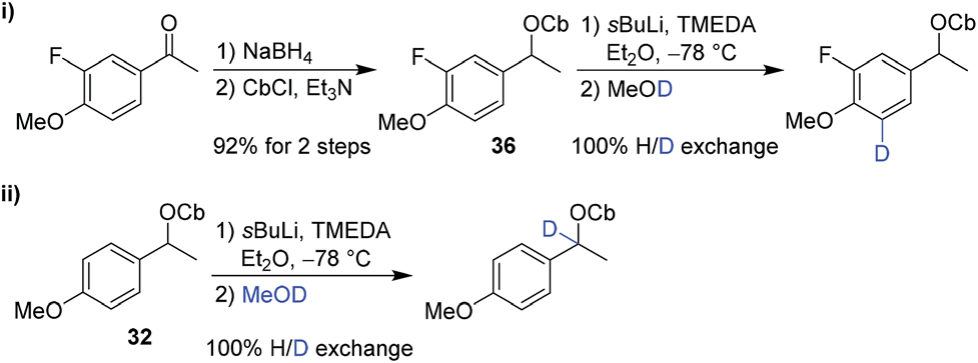
Lithiation–deuteration of carbamate **32** and **36** to determine site of lithiation.

The second partner, carbamate **36**, was prepared from the corresponding ketone. However, in test reactions we found that lithiation–deuteration of carbamate **36** gave complete H/D exchange, not at the benzylic position as required, but instead in the position *ortho* to the methoxy group, presumably as a result of its greater acidity and the directing effects of the F and OMe substituents (Scheme 7).^19^ We therefore decided to introduce this fluorine substituent at the end of the synthesis.

Carbamate (*S*)-**32** was prepared from alcohol (*S*)-**31** in quantitative yield and with 98% ee (Scheme 6-ii).^20^ Lithiation followed by addition of boronic ester (*S*)-**30** gave tertiary boronic ester **33** in 95% yield and with >95 : 5 dr in favour of the desired *anti* isomer. Surprisingly, no additives were necessary to accelerate the 1,2-migration of the intermediate boronate complex, presumably because the *p*-MeO group on the carbamate accelerates the 1,2-migration by electron donation. Tertiary boronic ester **33** was protodeboronated in nearly quantitative yield to furnish **34** using TBAF·3H_2_O with retention of stereochemistry and without any erosion of dr. Electrophilic aromatic bromination with NBS, lithiation and fluorination with NFSI (*N*-fluorobenzenesulfonimide)^21^ and finally deprotection of both methoxy groups gave **1** in 40% yield over three steps. Overall, bifluranol was synthesised in 7 steps (longest linear sequence) and 27% overall yield as a single stereoisomer.

### Enantioselective synthesis of fluorohexestrol

**Scheme 8:**
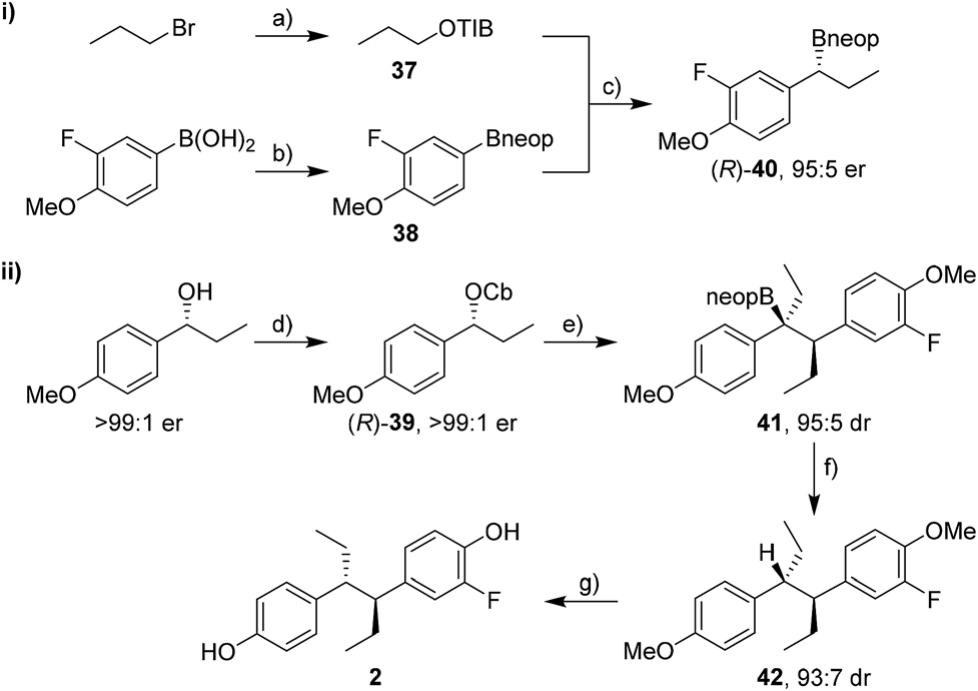
Synthesis of fluorohexestrol **2**. Reaction conditions: (a) 1-bromopropane (3.0 equiv.), 2,4,6-triisopropylbenzoic acid (1.0 equiv.), NBu_4_HSO_4_ (0.08 equiv.), NaOH (3.0 equiv.), CHCl_3_/H_2_O (1 : 1), 84% yield; (b) neopentylglycol (1.0 equiv.), Et_2_O, r.t., 16 h; MgSO_4_ (3.0 equiv.), r.t., 2 h, 95% yield; (c) **37** (1.8 equiv.), (–)-sparteine (1.7 equiv.), *s*BuLi (1.7 equiv.), Et_2_O, –78 °C, 5 h; **38** (1 equiv.), –78 °C, 1 h; reflux, 15 h, 46% yield; (d) CbCl (1.2 equiv.), Et_3_N (1.3 equiv.), toluene, μW, 150 °C, 2 h, 98% yield; (e) TMEDA (1.3 equiv.), *s*BuLi (1.3 equiv.), Et_2_O, –78 °C, 1 h; (*R*)-**40** (1.5 equiv.), –78 °C, 3 h; r.t., 14 h, 70% yield; (f) TBAF·3H_2_O (3.0 equiv.), toluene, reflux, 3 h, 81% yield; (g) BBr_3_ (3.0 equiv.), CH_2_Cl_2_, –20 °C, 30 min; 4 °C, 15 h, 72% yield. TIB = 2,4,6-triisopropylbenzoyl, *s*BuLi = *sec*-butyllithium, TMEDA = *N*,*N*,*N*′,*N*′-tetramethylethylenediamine, TBAF·3H_2_O = tetrabutylammonium fluoride trihydrate.

The synthesis of fluorohexestrol (**2**) was expected to be more challenging as it required the lithiation–borylation reaction of a more hindered carbamate, a reaction that was especially challenging with hindered boronic esters. Indeed, the lithiation–borylation reaction between carbamate (*R*)-**39** (>99 : 1 er) and pinacol boronic ester (*R*)-**30** under conditions **A** or **B** failed to provide the desired boronic ester. However, using the corresponding neopentylglycol boronic ester (*R*)-**40** (95 : 5 er) instead gave the homologated boronic ester **41** in 70% yield and 95 : 5 dr (Scheme 8). Subsequent protodeboronation using TBAF·3H_2_O gave diarylethane **42** in 81% yield with minimal erosion of dr. Deprotection of the methoxy groups using BBr_3_ and separation of the minor *syn* diastereoisomer by column chromatography gave fluorohexestrol **2**. The *anti* configuration of fluorohexestrol was confirmed by single-crystal X-ray diffraction analysis (see ESI†). Fluorohexestrol was obtained in 18% overall yield in just 5 linear steps from commercially available starting materials and with very high selectivity.

## Conclusions

In conclusion, by studying the reactions of the most challenging of substrates we have been able to define the scope and limitations of the lithiation–borylation reaction between a hindered secondary benzylic carbamate and a hindered benzylic pinacol boronic ester. The ate complexes derived from these very hindered substrates are especially prone to revert back to either stabilised benzylic anions, which can undergo racemisation and re-addition. However, by using MgBr_2_/MeOH as an additive to promote 1,2-migration over reversion back to starting materials the yield and the diastereo- and enantioselectivity of this reaction can be enhanced dramatically. For the most hindered of coupling partners where no boronate complexes even form, the use of neopentyl boronic esters enables coupling to take place in high yield and with high selectivity. Furthermore, we have demonstrated the application of this methodology in the first enantioselective and diastereoselective synthesis of bifluranol and fluorohexestrol in a short number of steps (7 and 5 steps respectively).

## Supplementary Material

Supplementary information
