# Supplementary material for: Enantioselective installation of adjacent tertiary benzylic stereocentres using lithiation–borylation–protodeboronation methodology. Application to the synthesis of bifluranol and fluorohexestrol
Source: Chem Sci. 2015 Apr 28;6(7):3718–23. doi: 10.1039/c4sc03901g (PMC5707455; doi:10.1039/c4sc03901g)
Supplement: Supplementary file 1 [file SC-006-C4SC03901G-s001.pdf]

Electronic Supplementary Material (ESI) for Chemical Science.
This journal is © The Royal Society of Chemistry 2015

Enantioselective Installation of Adjacent Tertiary Benzylic Stereocentres Using Lithiation–Borylation–Protodeboronation Methodology. Application to the Synthesis of Bifluranol and Fluorohexestrol.

Stefan Roesner, Daniel J. Blair, Varinder K. Aggarwal*

School of Chemistry, University of Bristol, Cantock's Close, Bristol, BS8 1TS, U.K.

E-mail: V.Aggarwal@bristol.ac.uk

Supporting Information

Table of Contents

1. General experimental information S2

2. Detailed procedures and analytical data S3

2.1 Preparation of secondary benzylic alcohols S3

2.2 Preparation of carbamates from alcohols S6

2.3 Preparation of aryl pinacol boronic esters from boronic acids S13

2.4 Preparation of secondary boronic esters S14

2.5 Preparation of tertiary boronic esters S23

2.6 Synthesis of bifluranol S38

2.7 Synthesis of fluorohexestrol S42

3. X-ray structure of fluorohexestrol S48

4. ^{1}H NMR and ^{13}C NMR spectra S49

5. Determination of diastereomeric ratio of tertiary boronic esters *via* ^{1}H NMR S92

6. References S94

1. General experimental information

Reaction mixtures were stirred magnetically. Air- and moisture-sensitive reactions were carried out in flame-dried glassware under argon atmosphere using standard Schlenk manifold technique. All required fine chemicals were purchased from Acros Organics, Alfa Aesar, Inochem-Frontier Scientific or Sigma-Aldrich and used as received unless otherwise mentioned. *sec*-Butyllithium (*s*BuLi) was received from Acros Organics as 1.3 M solution in cyclohexane/hexane 92:8 and the molarity was verified by titration with *N*-benzylbenzamide.[1] Petrol refers to the fraction of petroleum ether boiling at 40-60 °C. 1 M $MgBr_2$ solutions in MeOH were prepared in advance by adding anhydrous MeOH to $MgBr_2$ solid. TMEDA was distilled over CaH_2 before use, (–)-sparteine and (+)-sparteine were isolated from the commercially available sulfate salt following a procedure by Beak.[2] Anhydrous THF, CH_2Cl_2, toluene, hexane, acetonitrile and Et_2O were dried by passing through a modified Grubbs system[3] of alumina columns, manufactured by Anhydrous Engineering. and were transferred under argon *via* syringes. Microwave reactions were carried out in a Biotage Initiator EXP EU microwave synthesiser. ^{1}H Nuclear Magnetic Resonance (NMR) spectra were recorded in $CDCl_3$ or acetone-*d6* at 301, 400 or 500 MHz on a Joel Lambda 300, Joel ECP 400, a Varian 400-MR or a VNMRS500a Fourier transform spectrometer. Chemical shifts (δ_H) are quoted in parts per million (ppm) and referred to the residual protio solvent signals of $CHCl_3$ (7.27 ppm) or acetone (2.05 ppm). ^{1}H NMR coupling constants are reported in hertz and refer to apparent multiplicities. Data are reported as follows: chemical shift, multiplicity (s = singlet, br. s = broad singlet, d = doublet, t = triplet, q = quartet, quin = quintet, sext = sextet, sept = septet, m = multiplet, dd = doublet of doublet, etc.), coupling constant, integration, and assignment. ^{13}C NMR spectra were recorded at 101 or 126 MHz. Chemical shifts (δ_C) are quoted in ppm referenced to $CHCl_3$ (77.0 ppm) or acetone (29.92 ppm). ^{11}B NMR spectra were measured using Norell S-200-QTZ quartz NMR tubes at 96 or 128 MHz with complete proton decoupling. ^{19}F NMR spectra were recorded at 283, 376 or 470 MHz. Mass spectra were recorded by the University of Bristol, School of Chemistry departmental mass spectrometry service using electron impact ionisation (EI), chemical ionisation (CI) or electrospray ionisation (ESI) techniques for low- and high-resolution mass spectra. HRMS EI and CI were performed on a VG Analytical Autospec mass spectrometer at 70 eV. HRMS ESI was performed on either a Bruker Daltonics Apex IV, 7-Tesla FT-ICR or micrOTOF II. Samples were submitted in EtOAc. For low resolution mass spectra (*m/z*) only molecular ions (M^+ or MH^+) and major peaks are reported with intensities quoted as percentage of the base peak. All infrared spectra were recorded on the neat compounds using a PerkinElmer Spectrum One FT-IR spectrometer, irradiating between 4000 cm^{-1} and 600 cm^{-1}. Only strong and selected absorbances (ν_{max}) are reported. Analytical TLC was performed on aluminium backed silica plates (Merck, Silica Gel 60 F_{254}, 0.25 mm). Compounds were visualised by fluorescence quenching or by staining the plates with 5% solution of phosphomolybdic acid

($H_3PMo_{12}O_{40}$) in EtOH followed by heating. Flash column chromatography was performed on silica gel (Aldrich, Silica Gel 60, 40–63 µm). All mixed solvent eluents are reported as v/v solutions. Optical rotations were obtained using a Bellingham + Stanley Ltd. ADP220 polarimeter at 589 nm (Na D-line) in a cell with a path length of 1 dm. Specific rotation values are given in (deg mL)/(g dm). Melting points were measured with a Reichert hot stage apparatus and are uncorrected. Chiral high performance liquid chromatography (HPLC) separations were performed on an Agilent 1100 Series HPLC unit equipped with UV-vis diode-array detector monitored at 210.8 nm, using Daicel Chiralpak ADH, IA, IB or IC columns (4.6 × 250 mm^2, 5 µm) fitted with respective guards (4 × 10 mm^2). Supercritical fluid chromatography (SFC) was performed on a Thar SFC investigator using a Daicel Chiralpak IA column (4.6 × 250 mm^2, 5 µm).

2. Detailed procedures and analytical data

2.1 Preparation of secondary benzylic alcohols

(*S*)-1-(4-Methoxyphenyl)ethanol ((*S*)-31)

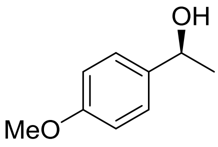

Methylmagnesium bromide solution (20.0 mL, 3.0 M in diethyl ether, 60.0 mmol, 1.2 equiv) was added slowly to a stirred solution of 4-methoxybenzaldehyde (6.81 g, 50.0 mmol, 1.0 equiv) in anhydrous diethyl ether (50 mL) at 0 °C. The reaction was allowed to warm to room temperature and stirred for 20 h. The mixture was then cooled to 0 °C, aq. NH_4Cl solution (2%, 50 mL) was added slowly, and the mixture was stirred for 10 min. The solution was extracted with diethyl ether (4 × 100 mL). The combined organic fractions were dried over anhydrous $MgSO_4$, filtered, and the solvent was removed *in vacuo* to give racemic alcohol **31** (7.61 g, 50.0 mmol) as a colourless oil in quantitative yield, which was used without further purification. According to a procedure by *Xu* and co-workers,[4] carrier-bound lipase from *Candida antarctica* (Novozym 435) (240 mg) was added to a solution of racemic alcohol **31** (6.01 g, 40.0 mmol, 1.0 equiv) and vinyl acetate (18.2 mL, 0.20 mol, 5.0 equiv) in diisopropyl ether (16.7 mL) and stirred for 12 h at 50 °C. The reaction was filtered, the solids were thoroughly washed with EtOAc, and the filtrate was concentrated *in vacuo*. The residue was subjected to column chromatography (SiO_2, pentane/EtOAc 9:1 → 4:1) to obtain alcohol (*S*)-**31** (2.96 g, 19.4 mmol, 49%) as a colourless oil and (*R*)-1-(4-methoxyphenyl)ethyl acetate (3.97 g, 20.4 mmol, 51%) as a colourless oil.

R_f (pentane/EtOAc 9:1) 0.08.

^{1}H NMR (500 MHz, $CDCl_3$) δ_H ppm 7.31 (AA'BB', *J* = 8.5 Hz, 2 H, CH_{Ar}), 6.89 (AA'BB', *J* = 8.5 Hz, 2 H, CH_{Ar}), 4.86 (q, *J* = 6.4 Hz, 1 H, C*H*OH), 3.81 (s, 3 H, OCH_3), 1.91 (br. s, 1 H, OH), 1.48 (d, *J* = 6.4 Hz, 3 H, CH_3).

^{13}C NMR (126 MHz, $CDCl_3$) δ_C ppm 158.9 (C), 137.9 (C), 126.6 (CH), 113.7 (CH), 69.9 (CHOH), 55.2 (OCH_3), 25.0 (CH_3).

$[\alpha]_D^{21}$ –50.0 (*c* 1.00, $CHCl_3$, for 99% ee). Lit. $[\alpha]_D^{22}$ –40.3 (*c* 1.20, $CHCl_3$, for 97% ee).[5]

The spectral data match those reported in literature.[5]

HPLC separation conditions: Chiralpak IB column with guard, 2.0% *i*PrOH in hexane, flow rate 0.7 mL/min, 20 °C; t_R 26.5 min for (*R*)-enantiomer (minor) and t_R 28.2 min for (*S*)-enantiomer (major).
e.r. = 99.6:0.4.

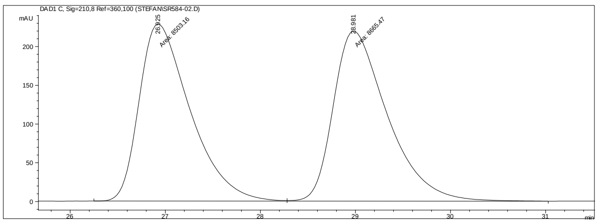

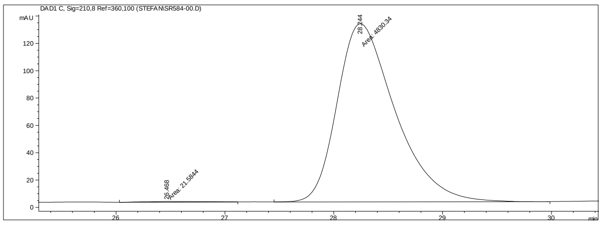

(*S*)-1-(4-Methoxyphenyl)propan-1-ol ((*S*)-43)

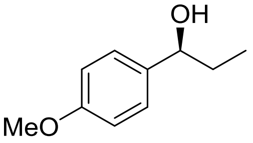

Following the procedure for the synthesis of (*S*)-1-(4-methoxyphenyl)ethanol ((*S*)-**31**) (*vide supra*), 4-methoxybenzaldehyde (6.81 g, 50.0 mmol, 1.0 equiv) and ethylmagnesium bromide solution (20.0 mL, 3.0 M in diethyl ether, 60.0 mmol, 1.2 equiv) in anhydrous diethyl ether (50 mL) afforded racemic alcohol **41** (8.30 g, 50.0 mmol) as colourless oil in quantitative yield. The crude alcohol **43** (7.65 g, 46.1 mmol, 1.0 equiv), vinyl acetate (23.0 mL, 0.25 mol, 5.4 equiv) and carrier-bound lipase from *Candida antarctica* (Novozym 435) (2.75 g) in diisopropyl ether (21.0 mL) gave after stirring for 26.5 h at 50 °C and column chromatography (SiO_2, pentane/ EtOAc 4:1) alcohol (*S*)-**43** (3.60 g, 21.7 mmol, 47%) as a colourless oil and (*R*)-1-(4-methoxy-phenyl)propyl acetate (4.32 g, 20.7 mmol, 45%) as a colourless oil.

R_f (pentane/EtOAc 4:1) 0.16.

^{1}H NMR (400 MHz, $CDCl_3$) δ_H ppm 7.27 (AA'BB', *J* = 8.8 Hz, 2 H, H_{Ar}), 6.89 (AA'BB', *J* = 8.8 Hz, 2 H, H_{Ar}), 4.55 (t, *J* = 6.7 Hz, 1 H, C*H*OH), 3.82 (s, 3 H, OCH_3), 1.89–1.68 (m, 3 H, CH_2+OH), 0.91 (t, *J* = 7.5 Hz, 3 H, CH_3).

^{13}C NMR (101 MHz, $CDCl_3$) δ_C ppm 159.0 (C), 136.7 (C), 127.2 (CH), 113.7 (CH), 75.6 (CHOH), 55.2 (OCH_3), 31.7 (CH_2), 10.2 (CH_3).

$[\alpha]^{22}_{D}$ −43.0 (*c* 1.00, $CHCl_3$, for 99% ee). Lit. $[\alpha]^{24}_{D}$ −23.4 (*c* 0.30, $CHCl_3$, for 65% ee).[6]

The analytical data correspond to the literature known compound.[7]

HPLC separation conditions: Chiralpak IB column with guard, 3.0% *i*PrOH in hexane, flow rate 0.7 mL/min, 20 °C; t_R 20.6 min for (*R*)-enantiomer (minor) and t_R 22.0 min for (*S*)-enantiomer (major).
e.r. = 99.9:0.1.

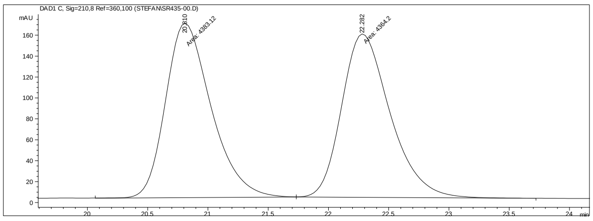

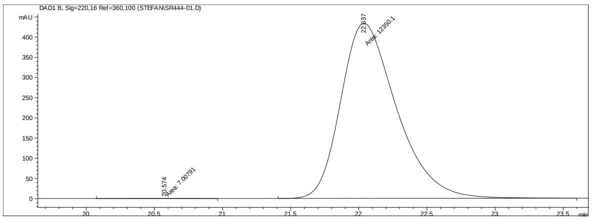

(*S*)-1-(4-Methoxyphenyl)propan-1-ol ((*R*)-43)

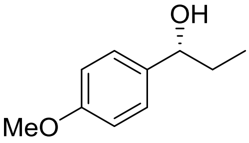

The (*R*)-1-(4-methoxy-phenyl)propyl acetate isolated from the synthesis of (*S*)-**43** was dissolved in MeOH (24 mL) and treated with 6 M aq. NaOH and stirred overnight. The solvent was removed, water (50 mL) was added to the residue which was extracted with EtOAc (4 × 50 mL). The combined organics were washed with brine (20 mL), dried over MgSO4, filtered and concentrated *in vacuo* to afford (R)-**43** (3.43 g, 20.7 mmol, 45%)

$[\alpha]^{22}_{D}$ +35.0 (*c* 1.00, $CHCl_3$, for 99% ee). Lit. $[\alpha]^{24}_{D}$ −23.4 (*c* 0.30, $CHCl_3$, for 65% ee).[6]

HPLC separation conditions: Chiralpak IB column with guard, 3.0% *i*PrOH in hexane, flow rate 0.7 mL/min, 20 °C; t_R 20.6 min for (*R*)-enantiomer (major) and t_R 22.0 min for (*S*)-enantiomer (minor).
e.r. = 99.9:0.1.

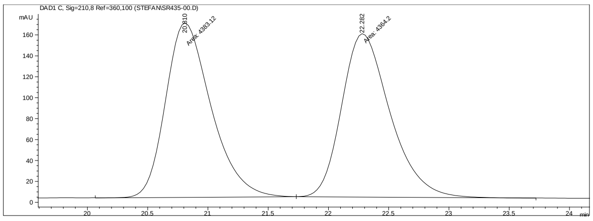

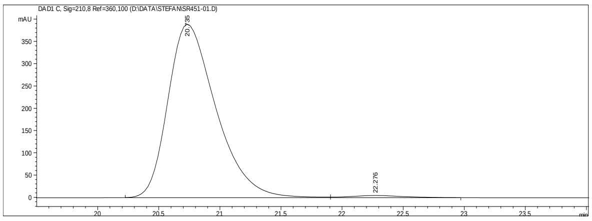

1-(3-Fluoro-4-methoxyphenyl)ethanol (44)

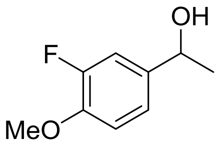

Sodium borohydride (284 mg, 7.5 mmol, 1.5 equiv) was added slowly to a solution of 1-(3-fluoro-4-methoxyphenyl)ethanone (841 mg, 5.0 mmol, 1.0 equiv) in anhydrous MeOH (10 mL) at 0 °C. After stirring for 2 h at room temperature H_2O (10 mL) was added slowly and the mixture was extracted with EtOAc (3 × 10 mL). The combined organic layers were washed with brine (15 mL), dried over anhydrous $MgSO_4$, filtered, and concentrated under reduced pressure to give alcohol **44** (851 mg, 5.0 mmol) as colourless oil in quantitative yield, which required no further purification.

^{1}H NMR (400 MHz, $CDCl_3$) δ_H ppm 7.13 (d, J = 12.3 Hz, 1 H, H_{Ar}), 7.07 (d, J = 8.4 Hz, 1 H, H_{Ar}), 6.93 (dd, J_{HF} = 8.4 Hz, J_{HH} = 8.4 Hz, 1 H, H_{Ar}), 4.85 (q, J = 6.4 Hz, 1 H, C*H*OH), 3.89 (s, 3 H, OCH_3), 1.83 (br. s, 1 H, OH), 1.47 (d, J = 6.4 Hz, 3 H, CH_3).

^{13}C NMR (101 MHz, $CDCl_3$) δ_C ppm 152.3 (d, ^1J = 246.0 Hz, CF), 146.8 (d, ^2J = 10.9 Hz, *C*OMe), 139.0 (d, ^3J = 5.5 Hz, C), 121.0 (d, ^3J = 3.1 Hz, CH), 113.3 (d, ^2J = 18.7 Hz, CH), 113.2 (d, ^4J = 2.3 Hz, CH), 69.5 (CHOH), 56.3 (OCH_3), 25.1 (CH_3).

^{19}F NMR (376 MHz, $CDCl_3$) δ_F ppm –137.1 (dd, J = 12.1, 8.2 Hz, CF).

ν_{max} (neat) = 3347, 2970, 1623, 1515, 1269, 1125, 1028, 875, 811, 760 cm^{-1}.

m/z (%) (CI^+) 171 ($[M+H]^+$, 64), 155 ($[M–Me]^+$, 27), 153 ($[M–OH]^+$, 100), 127 ($[Ar+H]^+$, 28).

HRMS (CI^+) calcd. for $C_9H_{12}O_2F$ $[M+H]^+$ 171.0821, found 171.0817.

2.2 Preparation of carbamates from alcohols

Propyl diisopropylcarbamate (28)

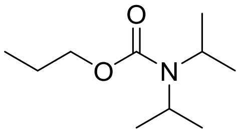

N,*N*-Diisopropylcarbamoyl chloride (1.64 g, 10.0 mmol, 1.0 equiv) was dissolved in 10.0 mL anhydrous *n*-propanol under an inert atmosphere in a microwave vial. The mixture was cooled to 0 °C and Et_3N (1.80 mL, 13.0 mmol, 1.3 equiv) was added slowly, the vial was sealed, and heated for 1 h at 150 °C in a microwave reactor. After cooling to ambient temperature, the salts were removed by filtration through a plug of silica and the solids were thoroughly washed with diethyl ether. The solvent was removed *in vacuo* to give primary carbamate **28** (1.73 g, 9.25 mmol, 92%) as a colourless oil, which showed no impurities in its NMR spectra.

^{1}H NMR (400 MHz, $CDCl_3$) δ_H ppm 4.04 (t, J = 7.1 Hz, 2 H, CH_2OCb), 4.01 (br. m, 1 H, C*H*$(CH_3)_2$), 3.90 (br. m, 1 H, C*H*$(CH_3)_2$), 1.67 (sext, J = 7.1 Hz, 2 H, CH_2), 1.21 (d, J = 6.8 Hz, 12 H, 4×CH_3), 0.97 (t, J = 7.1 Hz, 3 H, CH_3).

^{13}C NMR (101 MHz, $CDCl_3$) δ_C ppm 156.0 (NCO), 66.3 (CH_2OCb), 45.7 (br., *C*H$(CH_3)_2$), 22.4 (CH_2), 21.0 (br., CH_3), 10.8 (CH_3).

The NMR data correspond to the literature known compound.[8]

General procedure 1A (GP1A). An alcohol (1.0 equiv) was added slowly to a suspension of sodium hydride (60% dispersion in mineral oil, 1.5 equiv) in anhydrous THF (0.2 M) and the mixture was stirred for 75 min at room temperature. A solution of *N,N*-diisopropylcarbamoyl chloride (1.2 equiv) in anhydrous THF (1.0 M) was added and the reaction mixture was heated under reflux for 24 h. The solvent was removed *in vacuo* and the residue was portioned between H_2O and diethyl ether. The phases were separated and the aqueous layer was re-extracted with diethyl ether (3 ×). The combined organic layers were dried over anhydrous $MgSO_4$ and concentrated under reduced pressure. The residue was subjected to flash chromatography (SiO_2, pentane/EtOAc) to give the pure carbamate.

General procedure 1B (GP1B). A secondary benzylic alcohol (1.0 equiv) and *N,N*-diisopropylcarbamoyl chloride (1.2 equiv) were dissolved in anhydrous toluene (1.0 M) under an inert atmosphere in a microwave vial. Et_3N (1.3 equiv) was added, the vial was sealed, and heated for 1 h at 150 °C in a microwave reactor. After cooling to ambient temperature, the salts were removed by filtration through a plug of silica and the solids were thoroughly washed with diethyl ether. The solvent was removed *in vacuo* and the residue was subjected to column chromatography (SiO_2, pentane/EtOAc) to afford the pure secondary benzylic carbamate.

(*S*)-1-Phenylethyl diisopropylcarbamate ((*S*)-5)

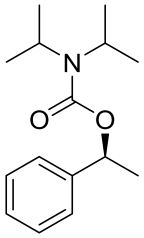

According to GP1A, (*S*)-1-phenylethanol (5.82 g, 47.6 mmol, 1.0 equiv), *N,N*-diisopropylcarbamoyl chloride (9.36 g, 57.2 mmol, 1.2 equiv) and sodium hydride (60% dispersion in mineral oil, 2.86 g, 71.5 mmol, 1.5 equiv) in anhydrous THF (150 mL) afforded after purification by column chromatography (SiO_2, pentane/EtOAc 6:1) carbamate (*S*)-**5** (11.7 g, 46.9 mmol, 98%) as a colourless oil.

R_f (pentane/EtOAc 6:1) 0.36.

^{1}H NMR (400 MHz, $CDCl_3$) δ_H ppm 7.39–7.33 (m, 4 H, H_{Ar}), 7.28 (m, 1 H, H_{Ar}), 5.86 (q, J = 6.7 Hz, 1 H, CHOCb), 4.08 (br. m, 1 H, C*H*(CH_3)$_2$), 3.83 (br. m, 1 H, C*H*(CH_3)$_2$), 1.56 (d, J = 6.7 Hz, 3 H, CH_3), 1.28–1.17 (br. m, 12 H, 4×CH_3).

^{13}C NMR (101 MHz, $CDCl_3$) δ_C ppm 155.0 (NCO), 142.8 (C), 128.3 (CH), 127.4 (CH), 126.0 (CH), 72.7 (CHOCb), 46.1 (br., *C*H(CH_3)$_2$), 45.3 (br., *C*H(CH_3)$_2$), 22.8 (CH_3), 21.3 (br., CH_3), 20.8 (br., CH_3).

$[\alpha]^{22}_{D}$ –6.5 (*c* 1.0, $CHCl_3$, for 99% ee). Lit. $[\alpha]^{20}_{D}$ –5.5 (*c* 1.2, CH_2Cl_2, for 99% ee).[9]

The analytical data match those reported in literature.[10]

HPLC separation conditions: Chiralpak IA column with guard, 5.0% *i*PrOH in hexane, flow rate 0.7 mL/min, 20 °C; t_R 8.3 min for (*R*)-enantiomer (minor) and t_R 9.5 min for (*S*)-enantiomer (major).
e.r. = 99.9:0.1.

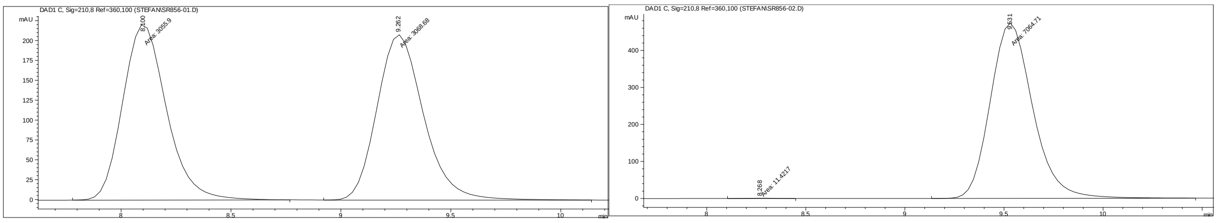

N.B. For the synthesis of racemic 1-phenylethyl diisopropylcarbamate **5** racemic 1-phenylethanol was used as starting material.

(*R*)-1-Phenylethyl diisopropylcarbamate ((*R*)-5)

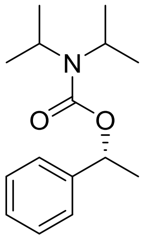

(*R*)-1-phenylethanol (12 g, 92 mmol, 1.0 equiv), *N,N*-diisopropylcarbamoyl chloride (18.0 g, 110 mmol, 1.2 equiv) and triethylamine (15.5mL, 110 mmol, 1.2 equiv) in anhydrous dichloromethane (200 mL) were heated at reflux for 48 h. The reaction mixture was cooled to room temperature and water (300 mL) was added. The organic phase was separated and the aqueous phase extracted with dichloromethane (3 ×300 mL) the organic phases were combined, dried (MgSO4), filtered and concentrated *in vacuo*. Purification by column chromatography (SiO_2, pentane/EtOAc 6:1) afforded carbamate (*R*)-**5** (21.6 g, 86 mmol, 94%) as a colourless oil.

$[\alpha]^{20}_{D}$ +7 (*c* 1.0, $CHCl_3$, for 99% ee). Lit. $[\alpha]^{20}_{D}$ –5.5 (*c* 1.2, CH_2Cl_2, for –99% ee).

Chiralpak IA column, eluent: 96% CO_2, 2% hexane, 2% *i*PrOH, flow rate 4.0 mL/min, 39.8 °C; t_R 2.27 min for (*S*)-enantiomer (minor) and t_R 2.74 min for (*R*)-enantiomer (major).

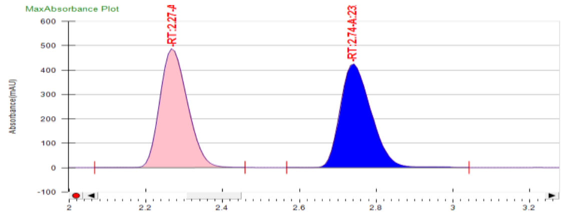

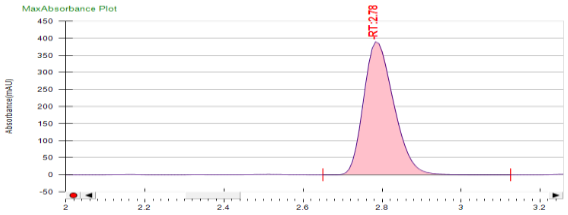

(*S*)-1-Phenylpropyl diisopropylcarbamate ((*S*)-6)

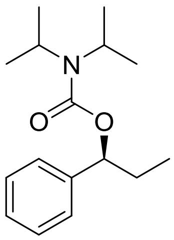

According to GP1B, (*S*)-1-phenylpropan-1-ol (1.01 g, 7.38 mmol, 1.0 equiv), *N*,*N*-diisopropylcarbamoyl chloride (1.45 g, 8.86 mmol, 1.2 equiv) and Et_3N (1.33 mL, 9.60 mmol, 1.3 equiv) in 8.0 mL anhydrous toluene afforded after purification by column chromatography (SiO_2, pentane/EtOAc 9:1) secondary benzylic carbamate (*S*)-**6** (1.90 g, 7.21 mmol, 98%) as a colourless oil.

R_f (pentane/EtOAc 9:1) 0.23.

^{1}H NMR (500 MHz, $CDCl_3$) δ_H ppm 7.38–7.29 (m, 4 H, H_{Ar}), 7.26 (m, 1 H, H_{Ar}), 5.65 (t, *J* = 7.2 Hz, 1 H, CHOCb), 4.05 (br. m, 1 H, C*H*(CH_3)$_2$), 3.85 (br. m, 1 H, C*H*(CH_3)$_2$), 1.96 (dquin, *J* = 14.3, 7.2 Hz, 1 H, C*H*H), 1.84 (dquin, *J* = 14.3, 7.2 Hz, 1 H, CH*H*), 1.22 (br. m, 12 H, 4×CH_3), 0.91 (t, *J* = 7.2 Hz, 3 H, CH_3).

^{13}C NMR (126 MHz, $CDCl_3$) δ_C ppm 155.1 (NCO), 141.5 (C), 128.2 (CH), 127.4 (CH), 126.5 (CH), 77.8 (CHOCb), 46.2 (br., *C*H(CH_3)$_2$), 45.3 (br., *C*H(CH_3)$_2$), 29.8 (CH_2), 21.5 (br., CH_3), 20.9 (br., CH_3), 10.0 (CH_3).

$[\alpha]_D^{20}$ –8 (*c* 1.0, $CHCl_3$, for 99% ee). Lit. $[\alpha]_D^{25}$ –8.0 (*c* 11, CH_2Cl_2, for 99% ee).[11]

The spectral data are consistent with the literature known compound.[11]

HPLC separation conditions: Chiralpak IA column with guard, 5.0% *i*PrOH in hexane, flow rate 0.7 mL/min, 20 °C; t_R 8.1 min for (*R*)-enantiomer (minor) and t_R 9.8 min for (*S*)-enantiomer (major).
e.r. = 99.1:0.9.

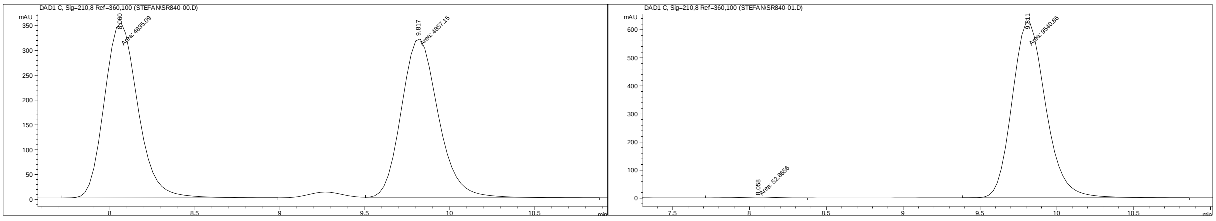

N.B. For the synthesis of racemic 1-phenylpropyl diisopropylcarbamate **6** racemic 1-phenylpropan-1-ol was used as starting material.

(*R*)-1-Phenylpropyl diisopropylcarbamate ((*R*)-6)

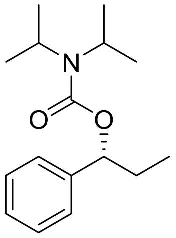

According to GP1B, (*R*)-1-phenylpropan-1-ol (1.01 g, 7.38 mmol, 1.0 equiv), *N*,*N*-diisopropylcarbamoyl chloride (1.45 g, 8.86 mmol, 1.2 equiv) and Et_3N (1.33 mL, 9.60 mmol, 1.3 equiv) in 8.0 mL anhydrous toluene afforded after purification by column chromatography (SiO_2, pentane/EtOAc 9:1) secondary benzylic carbamate (*S*)-**6** (1.80 g, 6.86 mmol, 93%) as a colourless oil.

$[\alpha]_D^{22}$ +7 (*c* 1.0, $CHCl_3$, for 99% ee). Lit. $[\alpha]_D^{25}$ –8.0 (*c* 11, CH_2Cl_2, for −99% ee).[11]

HPLC separation conditions: Chiralpak IA column with guard, 5.0% *i*PrOH in hexane, flow rate 1 mL/min, 20 °C; t_R 4.2 min for (*R*)-enantiomer (major) and t_R 4.7 min for (*S*)-enantiomer (minor).
e.r. = 99.1:0.9.

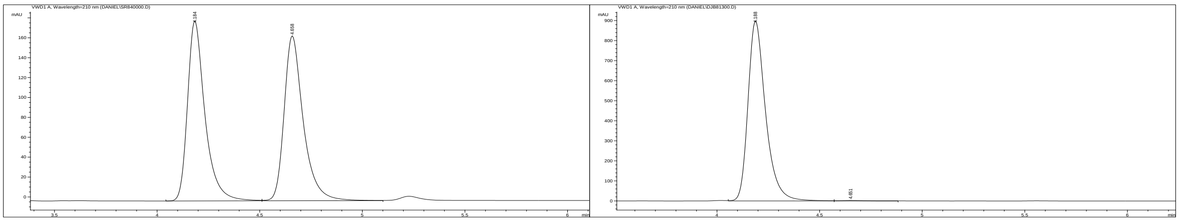

(*S*)-1-(4-Methoxyphenyl)ethyl diisopropylcarbamate ((*S*)-32)

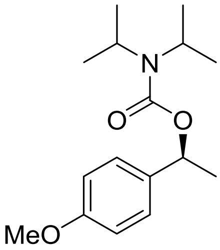

According to GP1A, (*S*)-1-(4-methoxyphenyl)ethanol ((*S*)-**31**) (7.61 g, 50.0 mmol, 1.0 equiv), *N*,*N*-diisopropylcarbamoyl chloride (9.82 g, 60.0 mmol, 1.2 equiv) and sodium hydride (60% dispersion in mineral oil, 3.00 g, 75.0 mmol, 1.5 equiv) in anhydrous THF (150 mL) afforded after purification by flash chromatography (SiO_2, pentane/EtOAc 9:1 → 4:1) carbamate (*S*)-**32** (13.9 g, 49.9 mmol, >99%) as a colourless oil.

$\mathbf{R_f}$ (pentane/EtOAc 4:1) 0.49.

^{1}H NMR (400 MHz, $CDCl_3$) δ_H ppm 7.31 (AA'BB', *J* = 8.5 Hz, 2 H, H_{Ar}), 6.88 (AA'BB', *J* = 8.5 Hz, 2 H, H_{Ar}), 5.81 (q, *J* = 6.5 Hz, 2 H, CHOCb), 4.12 (br. m, 1 H, C*H*$(CH_3)_2$), 3.80 (s, 3 H, OCH_3), 3.73 (br. m, 1 H, C*H*$(CH_3)_2$), 1.54 (d, *J* = 6.5 Hz, 3 H, CH_3), 1.19 (d, *J* = 6.8 Hz, 12 H, 4×CH_3).

^{13}C NMR (101 MHz, $CDCl_3$) δ_C ppm 158.9 (C), 155.1 (NCO), 134.9 (C), 127.4 (CH), 113.7 (CH), 72.3 (CHOCb), 55.2 (OCH_3), 46.3 (br., *C*H$(CH_3)_2$), 45.1 (br., *C*H$(CH_3)_2$), 22.6 (CH_3), 21.5 (br., CH_3), 20.8 (br., CH_3).

$[\alpha]_D^{22}$ −14.3 (*c* 1.12, CH_2Cl_2, for 99% ee). Lit. $[\alpha]_D^{24}$ −40.0 (*c* 1.0, CH_2Cl_2, for 96% ee).[12]

The analytical data correspond to the literature known compound.[12]

HPLC separation conditions: Chiralpak IC column with guard, 2.0% *i*PrOH in hexane, flow rate 0.7 mL/min, 20 °C; t_R 19.1 min for (*R*)-enantiomer (minor) and t_R 27.0 min for (*S*)-enantiomer (major).
e.r. = 99.9:0.1.

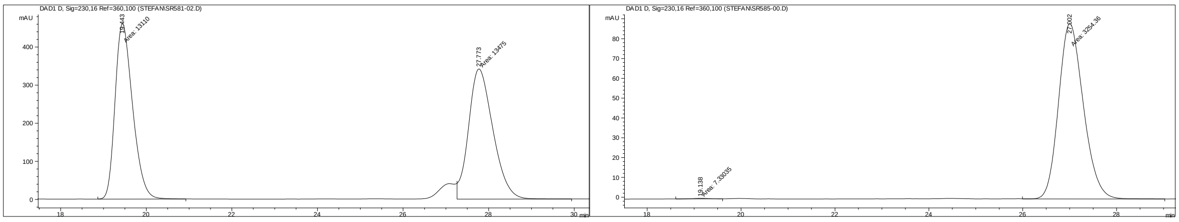

(*R*)-1-(4-Methoxyphenyl)propyl diisopropylcarbamate ((*R*)-39)

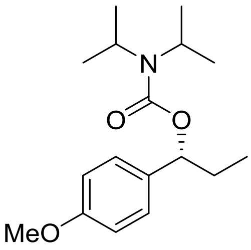

According to GP1B, (*S*)-1-(4-methoxyphenyl)propan-1-ol ((*R*)-**43**) (1.80 g, 10.8 mmol, 1.0 equiv), *N*,*N*-diisopropylcarbamoyl chloride (2.13 g, 13.0 mmol, 1.2 equiv) and Et_3N (1.95 mL, 14.0 mmol, 1.3 equiv) in anhydrous toluene (10 mL) afforded after purification by column chromatography (SiO_2, pentane/EtOAc 4:1) secondary benzylic carbamate (*S*)-**37** (3.12 g, 10.6 mmol, 98%, 99:1 er) as a colourless oil.

R_f (pentane/EtOAc 4:1) 0.48.

^{1}H NMR (400 MHz, $CDCl_3$) δ_H ppm 7.27 (AA’BB’, *J* = 8.8 Hz, 2 H, H_{Ar}), 6.87 (AA’BB’, *J* = 8.8 Hz, 2 H, H_{Ar}), 5.59 (t, *J* = 6.9 Hz, 1 H, CHOCb), 4.07 (br. m, 1 H, C*H*(CH_3)$_2$), 3.80 (s, 3 H, OCH_3), 3.79 (br. m, 1 H, C*H*(CH_3)$_2$), 1.96 (m, 1 H, C*H*H), 1.80 (m, 1 H, CH*H*), 1.21 (br. m, 12 H, 4×CH_3), 0.88 (t, *J* = 7.3 Hz, 3 H, CH_3).

^{13}C NMR (101 MHz, $CDCl_3$) δ_C ppm 158.8 (NCO), 155.2 (C), 133.6 (C), 127.9 (CH), 113.6 (CH), 77.5 (CHOCb), 55.2 (OCH_3), 45.8 (br., *C*H(CH_3)$_2$), 29.7 (CH_2), 21.2 (br., CH_3), 10.1 (CH_3).

ν_{max} (neat) = 2968, 1682, 1514, 1435, 1285, 1247, 1047, 828 cm^{-1}.

m/z (%) (CI^+) 294 ($[M+H]^+$, 24), 149 ($[M–OCb]^+$, 95), 121 (12), 102 ([NH(*i*Pr)$_2$+H]$^+$, 40).

HRMS (CI^+) calcd. for $C_{17}H_{28}NO_3$ $[M+H]^+$ 294.2069, found 294.2065.

$[\alpha]_D^{23}$ +10 (*c* 1.0, $CHCl_3$, for 96% ee).

SFC separation conditions: Chiralpak IA column, eluent: 80% CO_2, 18% hexane, 2% *i*PrOH, flow rate 4.0 mL/min, 39.8 °C; t_R 4.95 min for (*R*)-enantiomer (major) and t_R 5.62 min for (*S*)-enantiomer (minor).

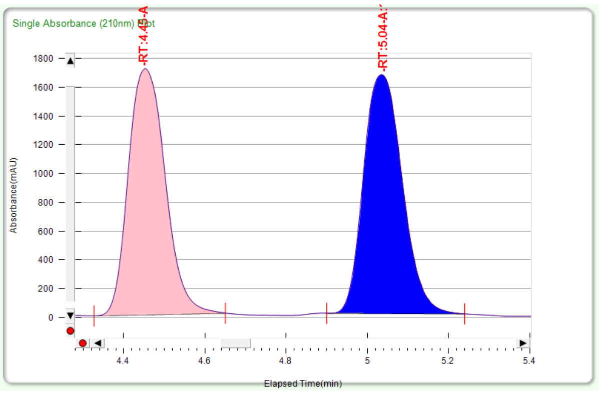

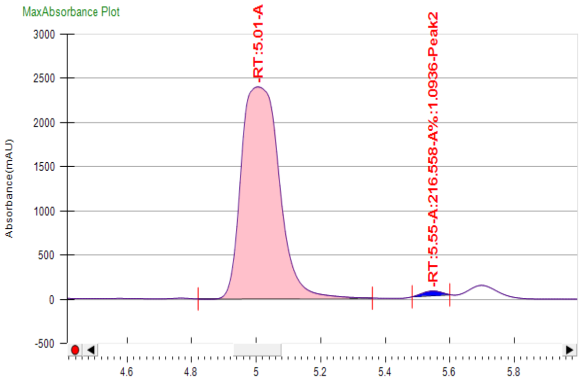

1-(3-Fluoro-4-methoxyphenyl)ethyl diisopropylcarbamate (36)

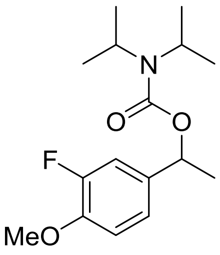

According to GP1B, 1-(3-fluoro-4-methoxyphenyl)ethanol (**44**) (851 mg, 5.00 mmol, 1.0 equiv), *N*,*N*-diisopropylcarbamoyl chloride (982 mg, 6.00 mmol, 1.2 equiv) and Et_3N (901 µL, 6.50 mmol, 1.3 equiv) in 10 mL anhydrous toluene afforded after purification by column chromatography (SiO_2, pentane/EtOAc 9:1) secondary benzylic carbamate **36** (1.38 g, 4.64 mmol, 93%) as a colourless oil.

R_f (pentane/EtOAc 9:1) 0.16.

^{1}H NMR (400 MHz, $CDCl_3$) δ_H ppm 7.13–7.05 (m, 2 H, H_{Ar}), 6.92 (dd, J_{HF} = 8.4 Hz, J_{HH} = 8.4 Hz, 1 H, H_{Ar}), 5.77 (q, *J* = 6.6 Hz, 1 H, CHOCb), 4.08 (br. m, 1 H, C*H*(CH_3)$_2$), 3.88 (s, 3 H, OCH_3), 3.74 (br. m, 1 H, C*H*(CH_3)$_2$), 1.52 (d, *J* = 6.6 Hz, 3 H, CH_3), 1.20 (d, *J* = 6.6 Hz, 12 H, 4×CH_3).

^{13}C NMR (101 MHz, $CDCl_3$) δ_C ppm 154.9 (NCO), 152.2 (d, ^1J = 245.5 Hz, CF), 146.8 (d, ^2J = 10.9 Hz, *C*OMe), 135.9 (d, ^3J = 5.7 Hz, C), 121.9 (d, ^3J = 3.5 Hz, CH), 113.8 (d, ^2J = 18.9 Hz, CH), 113.1 (d, ^4J = 2.0 Hz, CH), 71.8 (d, ^4J = 1.3 Hz, CHOCb), 56.2 (OCH_3), 46.3 (br., *C*H(CH_3)$_2$), 45.2 (br., *C*H(CH_3)$_2$), 22.6 (CH_3), 21.4 (br., CH_3), 20.8 (br., CH_3).

^{19}F NMR (376 MHz, $CDCl_3$) δ_F ppm –139.8 (dd, *J* = 12.1, 8.7 Hz, CF).

ν_{max} (neat) = 2971, 1682, 1520, 1433, 1271, 1130, 1046, 900, 810, 761 cm^{-1}.

m/z (%) (CI^+) 298 ($[M+H]^+$, 21), 153 ($[M–OCb]^+$, 100), 128 ($[Cb]^+$, 12).

HRMS (CI^+) calcd. for $C_{16}H_{25}NO_3F$ $[M+H]^+$ 298.1818, found 298.1810.

2.3 Preparation of aryl pinacol boronic esters from boronic acids

2-(3-Fluoro-4-methoxyphenyl)-4,4,5,5-tetramethyl-1,3,2-dioxaborolane (29)

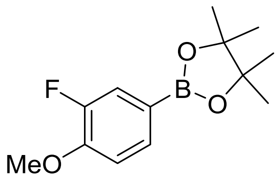

Based on a procedure by *Roush* and co-workers,[13] 3-fluoro-4-methoxyphenylboronic acid (2.55 g, 15.0 mmol, 1.0 equiv) and pinacol (1.77 g, 15.0 mmol, 1.0 equiv) in 22.5 mL anhydrous diethyl ether were stirred at room temperature for 16 h. Flame-dried $MgSO_4$ (5.42 g, 45.0 mmol, 3.0 equiv) was added and the mixture was stirred for additional 2 h at room temperature. The solution was filtered through a plug of anhydrous $MgSO_4$ and the solids were thoroughly washed with diethyl ether. The combined filtrates were concentrated *in vacuo* and dried under high vacuum to give the boronic ester **29** (3.78 g, 15.0 mmol) as a white solid in quantitative yield, which required no further purification.

mp 88–89 °C (diethyl ether).

^{1}H NMR (400 MHz, $CDCl_3$) δ_H ppm 7.54 (d, J = 8.2 Hz, 1 H, H_{Ar}), 7.50 (d, J = 11.7 Hz, 1 H, H_{Ar}), 6.96 (dd, J_{HF} = 8.2 Hz, J_{HH} = 8.2 Hz, 1 H, H_{Ar}), 3.92 (s, 3 H, OCH_3), 1.34 (s, 12 H, 4×CH_3).

^{13}C NMR (101 MHz, $CDCl_3$) δ_C ppm 152.0 (d, ^1J = 246.0 Hz, CF), 150.2 (d, ^2J = 10.7 Hz, *C*OMe), 131.4 (d, ^3J = 3.7 Hz, CH), 121.7 (d, ^2J = 16.4 Hz, CH), 112.5 (d, ^4J = 1.2 Hz, CH), 83.8 (O*C*(CH_3)$_2$), 56.0 (OCH_3), 24.8 (CH_3).

^{11}B NMR (128 MHz, $CDCl_3$) δ_B ppm 30.3 (br. s).

^{19}F NMR (376 MHz, $CDCl_3$) δ_F ppm –137.1 (dd, J = 11.3, 7.8 Hz, CF).

ν_{max} (neat) = 2978, 1616, 1422, 1353, 1269, 1130, 1027, 967, 915, 853, 813, 758, 676 cm^{-1}.

m/z (%) (CI$^+$) 253 ([M+H]$^+$, 100), 252 ([M]$^+$, 38), 237 ([M–CH_3]$^+$, 4), 233 ([M–F]$^+$, 4) 127 ([Bpin]$^+$, 5).

HRMS (CI$^+$) calcd. for $C_{13}H_{19}O_3{}^{11}BF$ [M+H]$^+$ 253.1411, found 253.1408.

2-(3-fluoro-4-methoxyphenyl)-5,5-dimethyl-1,3,2-dioxaborinane (38)

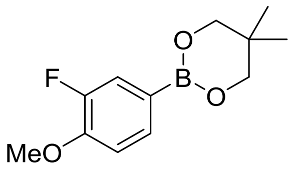

Based on a procedure by *Roush* and co-workers,[13] 3-fluoro-4-methoxyphenylboronic acid (2.55 g, 15.0 mmol, 1.0 equiv) and neopentyl glycol (1.56 g, 15.0 mmol, 1.0 equiv) in 22.5 mL anhydrous diethyl ether were stirred at room temperature for 16 h. Flame-dried $MgSO_4$ (5.42 g, 45.0 mmol, 3.0 equiv) was added and the mixture was stirred for additional 2 h at room temperature. The solution was filtered through a plug of anhydrous

$MgSO_4$ and the solids were thoroughly washed with diethyl ether. The combined filtrates were concentrated *in vacuo* and dried under high vacuum to give the boronic ester **38** (3.40 g, 14.3 mmol, 95%) as a white solid, which required no further purification.

mp 65–66 °C (Et_2O).

^{1}H NMR (300 MHz, $CDCl_3$) δ_H ppm 7.59 – 7.42 (m, 2H), 6.95 (t, J=7.9, 1H), 3.91 (s, 3H), 3.76 (s, 4H), 1.02 (s, 6H).

^{13}C NMR (101 MHz, $CDCl_3$) δ_C ppm 152.16 (d, J=245.4, CF), 149.81 (d, J=10.8, COMe), 130.41 (d, J=3.6, CH), 120.95 (d, J=16.0, CH), 112.48 (d, J=1.6, CH), 72.38 (CH_2), 56.09 (CH_3), 31.98 (C), 21.97 (CH_3).

^{11}B NMR (96 MHz, $CDCl_3$) δ_B ppm 25.4 (br. s).

^{19}F NMR (283 MHz, $CDCl_3$) δ_F ppm −137.38 (dd, *J*=12.3, 8.3).

ν_{max} (neat) = 2957, 2914, 2872, 1611, 1518, 1308, 1251, 1133, 1024, 814, 757, 669 cm^{-1}.

HRMS (EI^+) calcd. for $C_{12}H_{16}O_3{}^{11}BF$ $[M]^+$ 238.1177, found 238.1178.

2.4 Preparation of secondary boronic esters

(*S*)-4,4,5,5-Tetramethyl-2-(1-phenylethyl)-1,3,2-dioxaborolane ((*S*)-8)

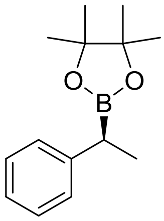

Following a procedure by *Aggarwal* and co-workers,[14] a solution of (*S*)-1-phenylethyl diisopropylcarbamate ((*S*)-**5**) (748 mg, 3.00 mmol, 1.0 equiv) in anhydrous diethyl ether (9.0 mL) was cooled to –78 °C. *s*BuLi (3.00 mL, 3.90 mmol, 1.3 equiv) was added dropwise and the reaction mixture was stirred at this temperature for 1 h. A solution of pinacolborane (871 µL, 6.00 mmol, 2.0 equiv) in anhydrous diethyl ether (4.5 mL) was added dropwise and the mixture was stirred for 2 h at –78 °C. The cooling bath was removed and the reaction mixture was stirred at ambient temperature for additional 2 h. The reaction mixture was then cooled to 0 °C and 1 M aqueous KH_2PO_4 was added slowly. After stirring for 10 min at room temperature, the phases were separated, and the aqueous phase was extracted with diethyl ether (4 × 20 mL). The combined organic layers were dried over anhydrous $MgSO_4$, filtered, and the solvent was removed *in vacuo*. The crude product was purified by column chromatography (SiO_2, pentane/EtOAc 30:1) to give secondary benzylic boronic ester (*S*)-**8** (641 mg, 2.76 mmol, 92%) as a colourless oil. Enantiomeric excess of the chiral boronic ester was determined by HPLC analysis of an aliquot oxidised to 1-phenylethan-1-ol according to GP3 (*vide infra*).

R_f (pentane/EtOAc 30:1) 0.11.

^{1}H NMR (500 MHz, $CDCl_3$) δ_H ppm 7.30–7.22 (m, 4 H, H_{Ar}), 7.14 (m, 1 H, H_{Ar}), 2.45 (q, *J* = 7.6 Hz, 1 H, CHBpin), 1.34 (d, *J* = 7.6 Hz, 3 H, CH_3), 1.22 (s, 6 H, 2×CH_3), 1.21 (s, 6 H, 2×CH_3).

^{13}C NMR (126 MHz, $CDCl_3$) δ_C ppm 144.9 (C), 128.3 (CH), 127.8 (CH), 125.0 (CH), 83.3 (O*C*(CH_3)$_2$), 24.62 (CH_3), 24.57 (CH_3), 17.0 (CH_3).

^{11}B NMR (96 MHz, $CDCl_3$) δ_B ppm 32.6 (br. s).

$[\alpha]_D^{21}$ +10.0 (*c* 1.00, $CHCl_3$, for 98% ee). Lit. $[\alpha]_D^{20}$ −12.0 (*c* 1.50, $CHCl_3$, for 94% ee of the (*R*)-isomer).[8]

The analytical data are consistent with the known product.[15]

HPLC separation conditions for 1-phenylethan-1-ol: Chiralpak IB column with guard, 2.0% *i*PrOH in hexane, flow rate 0.7 mL/min, 20 °C; t_R 23.4 min for (*R*)-enantiomer (minor) and t_R 26.9 min for (*S*)-enantiomer (major).
e.r. = 98.9:1.1.

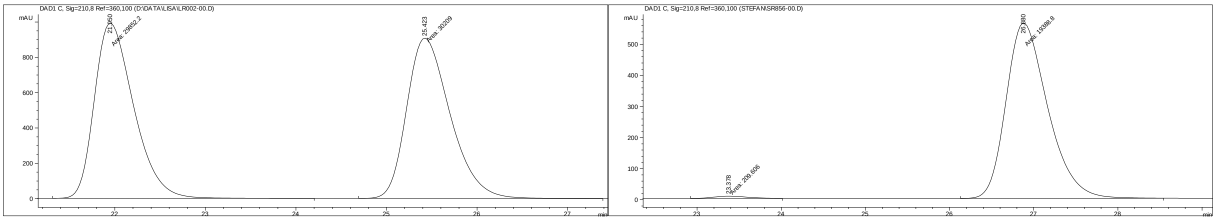

N.B. For the synthesis of racemic 4,4,5,5-tetramethyl-2-(1-phenylethyl)-1,3,2-dioxaborolane (**8**) racemic 1-phenylethyl diisopropylcarbamate (**5**) was used as starting material. Alternatively, a rhodium-catalysed hydroboration procedure by *Shibata* was followed.[16]

4,4,5,5-Tetramethyl-2-(1-phenylpropyl)-1,3,2-dioxaborolane (9)

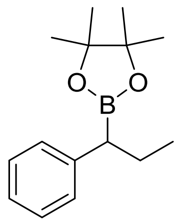

A solution of propyl 2,4,6-triisopropylbenzoate[17] (307 mg, 1.06 mmol, 1.0 equiv) and TMEDA (205 µL, 1.37 mmol, 1.3 equiv) in anhydrous diethyl ether (3.0 mL) was cooled to −78 °C. *s*BuLi (1.06 mL, 1.37 mmol, 1.3 equiv) was added dropwise and the reaction mixture was stirred for 30 min. A solution of 4,4,5,5-tetramethyl-2-phenyl-1,3,2-dioxaborolane (325 mg, 1.59 mmol, 1.5 equiv) in anhydrous diethyl ether (1.5 mL) was added dropwise and the mixture was stirred for 1 h at −78 °C. Afterwards, the cooling bath was removed and the reaction mixture was heated under reflux for 17 h. The reaction mixture was cooled to 0 °C and 1.0 M aq. KH_2PO_4 (2.0 mL) was added slowly. After stirring for 10 min at room temperature, the phases were separated, and the aqueous phase was extracted with diethyl ether (4 × 20 mL). The combined organic phases were dried over anhydrous $MgSO_4$, filtered, and the solvent was removed *in vacuo*. The crude product was purified by column

chromatography (SiO_2, pentane/EtOAc 30:1) to afford secondary boronic ester **9** (213 mg, 0.87 mmol, 82%) as a colourless oil.

R$_f$ (pentane/EtOAc 30:1) 0.23.

^{1}H NMR (400 MHz, $CDCl_3$) δ_H ppm 7.29–7.19 (m, 4 H, H_{Ar}), 7.14 (m, 1 H, H_{Ar}), 2.23 (t, *J* = 7.8 Hz, 1 H, CHBpin), 1.89 (m, 1 H, C*H*H), 1.68 (m, 1 H, CH*H*), 1.22 (s, 6 H, 2×CH_3), 1.21 (s, 6 H, 2×CH_3), 0.92 (t, *J* = 7.3 Hz, 3 H, CH_3).

^{13}C NMR (126 MHz, $CDCl_3$) δ_C ppm 143.3 (C), 128.4 (CH), 128.2 (CH), 125.1 (CH), 83.2 (O*C*(CH_3)$_2$), 34.3 (br., CHBpin), 25.8 (CH_2), 24.64 (CH_3), 24.56 (CH_3), 13.9 (CH_3).

^{11}B NMR (96 MHz, $CDCl_3$) δ_B ppm 32.9 (br. s).

The spectral data match those reported in literature.[18]

(*S*)-5,5-dimethyl-2-(1-phenylpropyl)-1,3,2-dioxaborinane ((S)-11)

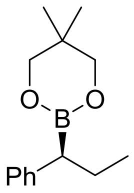

A solution of propyl 2,4,6-triisopropylbenzoate[17] (3.89 g, 13.4 mmol, 1.5 equiv) and (+)-spartiene (2.93 mL, 12.5 mmol, 1.4 equiv) in anhydrous diethyl ether (40 mL) was cooled to −78 °C. *s*BuLi (9.60 mL, 12.5 mmol, 1.4 equiv) was added dropwise and the reaction mixture was stirred for 5 hours. A solution of 5,5-dimethyl-2-phenyl-1,3,2-dioxaborinane (1.70 g, 8.90 mmol, 1 equiv) in anhydrous diethyl ether (9 mL) was added dropwise and the mixture was stirred for 1 h at −78 °C. Afterwards, the cooling bath was removed and the reaction mixture was heated under reflux for 17 h. The reaction mixture was cooled to room temperature diluted with Et_2O (100 mL) and quenched through addition of 1M HCl (40 mL). The organic layer was separated, dried over $MgSO_4$, filtered, and the solvent was removed *in vacuo*. The crude product was purified by column chromatography (SiO_2, pentane/Et_2O 2.5% *then* 10% with 1% Et_3N) to afford secondary boronic ester (*S*)-**11** (633 mg, 2.76 mmol, 31%, 97:3 er) as a colourless oil.

N.B. (*S*)-**11** Decomposes on silica gel so it is important to perform column chromatography quickly using 2.5% Et_2O to remove excess propyl 2,4,6-triisopropylbenzoate and then 10% Et_2O to elude (*S*)-**11**.

R$_f$ (pentane/Et_2O 9:1) 0.51.

^{1}H NMR (300 MHz, $CDCl_3$) δ_H ppm 7.28-7.16 (m, J = 14.0, 4H, H_{Ar}), 7.11 (m, 1H, H_{Ar}), 3.57 (s, 4H, 2×OCH_2), 2.09 (s, 1H, CH), 1.86 (dq, J = 14.1, 7.1, 1H, CH*H*), 1.62 (dq, J=14.1, 7.1, 1H, C*H*H), 0.89 (s, 6H, C(CH_3)$_2$), 0.88 (t, J = 7.3, 3H, CH_2CH_3).

^{13}C NMR (101 MHz, $CDCl_3$) δ_C ppm 144.6 (C), 128.4 (CH), 128.2 (CH), 125.0 (CH), 72.2 (CH_2), 31.8 (C), 25.3 (CH_2), 21.9 (CH_3), 14.2 (CH_3).

^{11}B NMR (96 MHz, $CDCl_3$) δ_B ppm 28.7 ppm

ν_{max} (neat) = 2959, 2931, 2871, 1601, 1476, 1416, 1327, 1284, 1252, 1166, 699 cm^{-1}.

HRMS (EI^+) calcd. for $C_{14}H_{21}BO_2$ $[M]^+$ 232.1635, found 232.1625.

$[\alpha]_D^{22}$ +21 (*c* 1, $CHCl_3$).

HPLC separation conditions: Chiralcel OD column, 2.0% *i*PrOH in hexane, flow rate 0.7 mL/min, 20 °C; t_R 14.8 min for (*R*)-enantiomer (minor) and t_R 16.9 min for (*S*)-enantiomer (major).

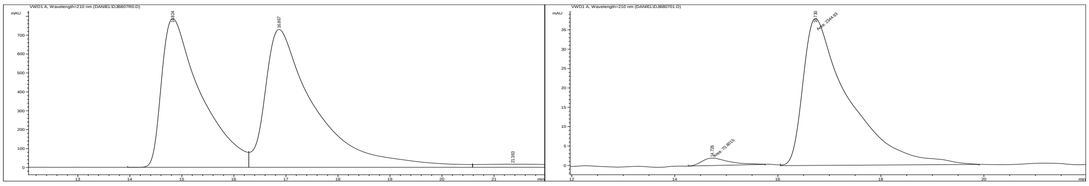

(*R*)-2-(1-(3-fluoro-4-methoxyphenyl)propyl)-5,5-dimethyl-1,3,2-dioxaborinane ((*R*)-40)

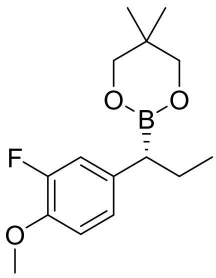

A solution of propyl 2,4,6-triisopropylbenzoate[17] (3.60 g, 12.4 mmol, 1.8 equiv) and (−)-sparteine (2.72 mL, 11.6 mmol, 1.7 equiv) in anhydrous diethyl ether (50 mL) was cooled to −78 °C. *s*BuLi (9.00 mL, 11.6 mmol, 1.7 equiv) was added dropwise and the reaction mixture was stirred for 5 hours. A solution of 2-(3-fluoro-4-methoxyphenyl)-5,5-dimethyl-1,3,2-dioxaborinane **38** (1.77 g, 7.02 mmol, 1 equiv) in anhydrous diethyl ether (8 mL) was added dropwise and the mixture was stirred for 1 h at −78 °C. Afterwards, the cooling bath was removed and the reaction mixture was heated under reflux for 17 h. The reaction mixture was cooled to room temperature diluted with Et_2O (100 mL) and quenched through addition of 1M HCl (100 mL). The organic layer was separated, dried over $MgSO_4$, filtered, and the solvent was removed *in vacuo*. The crude product was purified by column chromatography (SiO_2, pentane/Et_2O 7:3 with 1% Et_3N) to afford secondary boronic ester (*R*)-**40** (907 mg, 3.24 mmol, 46%) as a colourless oil.

R_f (pentane/Et_2O 7:3) 0.55.

$[\alpha]_D^{22}$ +10 (*c* 0.4, $CHCl_3$).

^{1}H NMR (400 MHz, $CDCl_3$) δ_H ppm 6.99 – 6.81 (m, 3H), 3.86 (s, 3H), 3.59 (s, 4H), 2.02 (t, J=7.8, 1H), 1.83 (m, 1H), 1.58 (m, 1H), 0.90 (s, 6H), 0.87 (t, J=7.3, 1H).

^{13}C NMR (101 MHz, $CDCl_3$) δ_C ppm 152.39 (d, J=244.2, CF), 144.99 (d, J=10.9, *C*OMe), 137.89 (d, J=6.0, C), 123.72 (d, J=3.3, CH), 115.85 (d, J=17.8, CH), 113.37 (d, J=2.4, CH), 72.23 (CH_2), 73.1 (CH_2), 56.42 (OCH_3), 31.75 (C), 25.40 (CH_2), 21.85 (CH_3), 13.99 (CH_3).

^{11}B NMR (96 MHz, $CDCl_3$) δ_B ppm 28.9 ppm

^{19}F NMR (283 MHz, $CDCl_3$) δ_F ppm −136.04 (dd, *J*=12.9, 7.4)

ν_{max} (neat) = 2960, 2933, 2873, 1734, 1583, 1514, 1477, 1418, 1255, 1219, 1172, 1127 cm^{-1}.

HRMS (EI^+) calcd. for $C_{15}H_{22}O_3{}^{11}BF$ $[M]^+$ 280.1646, found 280.1652.

HPLC separation conditions: Chiralpak IB column with guard, 3.0% *i*PrOH in hexane, flow rate 1 mL/min, 20 °C; t_R 24.4 min for (*R*)-enantiomer (major) and t_R 26.1 min for (*S*)-enantiomer (minor).

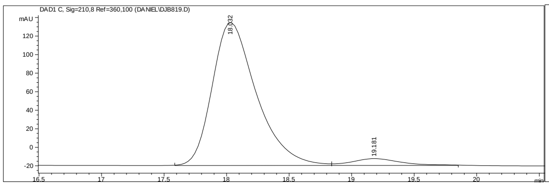
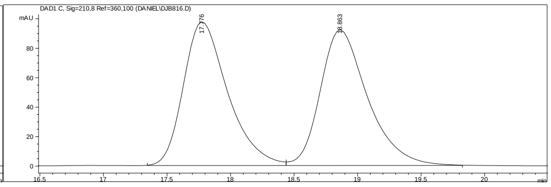

General procedure 2A (GP2A). A solution of a primary carbamate (1.0 equiv) and TMEDA, (+)-sparteine or (–)-sparteine (1.3 equiv respectively) in anhydrous diethyl ether (0.33 M) was cooled to −78 °C. *s*BuLi (1.3 equiv) was added dropwise and the reaction mixture was stirred at this temperature for 5 h. A solution of the boronic ester (1.5 equiv) in anhydrous diethyl ether (0.75 M) was added dropwise and the mixture was stirred for 2 h at −78 °C. Afterwards, the mixture was allowed to warm to room temperature and the solvent was removed under reduced pressure. Then anhydrous $CHCl_3$ (0.20 M) was added and the reaction mixture was heated under reflux until disappearance of the boron ate complex (5–8 ppm) monitored by ^{11}B NMR. The reaction mixture was cooled to 0 °C and 1.0 M aq. KH_2PO_4 was added slowly. After stirring for 10 min at room temperature, the phases were separated, and the aqueous phase was extracted with diethyl ether (4 ×). The combined organic phases were dried over anhydrous $MgSO_4$, filtered, and the solvent was removed *in vacuo*. The crude product was purified by flash chromatography (SiO_2, pentane/EtOAc 30:1) to afford the boronic ester.

General procedure 2B (GP2B). Following GP2A, after warming to ambient temperature the solvent was not removed *in vacuo*. The ethereal solution was heated under reflux until disappearance of the boron ate complex (5–8 ppm) monitored by ^{11}B NMR.

(*S*)-2-(1-(3-Fluoro-4-methoxyphenyl)propyl)-4,4,5,5-tetramethyl-1,3,2-dioxaborolane ((*S*)-30)

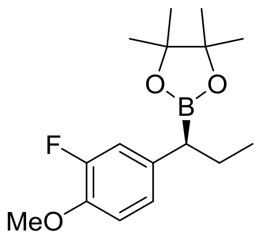

According to GP2A, propyl diisopropylcarbamate (**28**) (936 mg, 5.00 mmol, 1.0 equiv), (+)-sparteine (1.49 mL, 1.30 mmol, 1.3 equiv), *s*BuLi (5.00 mL, 1.30 mmol, 1.3 equiv), and 2-(3-fluoro-4-methoxyphenyl)-4,4,5,5-tetramethyl-1,3,2-dioxaborolane (**29**) (1.89 g, 1.50 mmol, 1.5 equiv) in 25 mL anhydrous solvent were heated under reflux for 15 h. After purification by column chromatography (SiO_2, pentane/EtOAc 30:1) secondary boronic ester (*S*)-**30** was obtained (1.12 g, 3.81 mmol, 76%) as a colourless oil. Enantiomeric excess of the chiral boronic ester was determined by HPLC analysis of an aliquot oxidised according to GP3 (*vide infra*).

R_f (pentane/EtOAc 30:1) 0.16.

^{1}H NMR (400 MHz, $CDCl_3$) δ_H ppm 6.96 (dd, J = 12.8, 1.8 Hz, 1 H, H_{Ar}), 6.92–6.82 (m, 2 H, H_{Ar}), 3.86 (s, 3 H, OCH_3), 2.15 (t, J = 7.9 Hz, 1 H, CHBpin), 1.82 (m, 1 H, C*H*H), 1.63 (m, 1 H, CH*H*), 1.22 (s, 6 H, 2×CH_3), 1.21 (s, 6 H, 2×CH_3), 0.90 (t, J = 7.3 Hz, 3 H, CH_3).

^{13}C NMR (101 MHz, $CDCl_3$) δ_C ppm 152.3 (d, ^1J = 244.4 Hz, CF), 145.1 (d, ^2J = 10.8 Hz, *C*OMe), 136.6 (d, ^3J = 6.0 Hz, C), 123.8 (d, ^3J = 3.3 Hz, CH), 115.9 (d, ^2J = 18.0 Hz, CH), 113.3 (d, ^4J = 2.2 Hz, CH), 83.3 (O*C*(CH_3)$_2$), 56.3 (OCH_3), 33.2 (br., CHBpin), 25.8 (CH_2), 24.64 (CH_3), 24.58 (CH_3), 13.7 (CH_3).

^{11}B NMR (128 MHz, $CDCl_3$) δ_B ppm 33.3 (br. s).

^{19}F NMR (376 MHz, $CDCl_3$) δ_F ppm –136.0 (dd, J = 12.1, 8.7 Hz, CF).

ν_{max} (neat) = 2976, 1514, 1358, 1322, 1268, 1141, 1031, 968, 867, 760 cm^{-1}.

m/z (%) (CI^+) 295 ($[M+H]^+$, 100), 294 ($[M]^+$, 78), 279 ($[M–Me]^+$, 16), 275 ($[M–F]^+$, 12), 265 ($[M–Et]^+$, 35), 167 ($[M–Bpin]^+$, 10).

HRMS (CI^+) calcd. for $C_{16}H_{25}O_3{}^{11}BF$ $[M+H]^+$ 295.1881, found 295.1877.

$[\alpha]_D^{21}$ +18.5 (*c* 1.13, $CHCl_3$, for 96% ee).

(*R*)-2-(1-(3-Fluoro-4-methoxyphenyl)propyl)-4,4,5,5-tetramethyl-1,3,2-dioxaborolane ((*R*)-30)

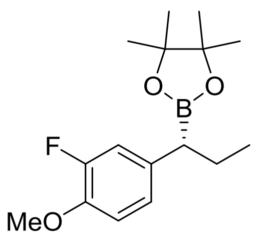

According to GP2B, propyl diisopropylcarbamate (**28**) (94 mg, 0.50 mmol, 1.0 equiv), (–)-sparteine (149 µL, 0.65 mmol, 1.3 equiv), *s*BuLi (500 µL, 0.65 mmol, 1.3 equiv), and 2-(3-fluoro-4-methoxyphenyl)-4,4,5,5-tetramethyl-1,3,2-dioxaborolane (**29**) (189 mg, 0.75 mmol, 1.5 equiv) in 2.5 mL anhydrous diethyl ether were heated

under reflux for 40 h. After purification by flash chromatography (SiO_2, pentane/EtOAc 30:1) secondary boronic ester (*R*)-**30** was obtained (108 mg, 0.37 mmol, 73%) as a colourless oil. Enantiomeric excess of the chiral boronic ester was determined by HPLC analysis of an aliquot oxidised according to GP3 (*vide infra*).

$[\alpha]_D^{21}$ –18.0 (*c* 1.00, $CHCl_3$, for 96% ee).

General procedure 3 (GP3). A solution of the secondary benzylic boronic ester (0.1 mmol, 1.0 equiv) in THF (4.0 mL) was cooled to 0 °C and a mixture of 2 M aq. NaOH (2.0 mL) and 30% H_2O_2 (1.0 mL) was added under vigorous stirring. The cooling bath was removed and the reaction mixture was stirred at room temperature for 2 h. The solvent was removed *in vacuo* and the residue was portioned between H_2O (15 mL) and diethyl ether (15 mL). The phases were separated and the aqueous layer was re-extracted with diethyl ether (2 × 15 mL). The combined organic layers were washed with brine (25 mL), dried over anhydrous $MgSO_4$, filtered, and the solvent was removed under reduced pressure. The residue was purified by flash chromatography (SiO_2, pentane/EtOAc 4:1) to give the pure alcohol.

(*S*)-1-(3-Fluoro-4-methoxyphenyl)propan-1-ol ((*S*)-45)

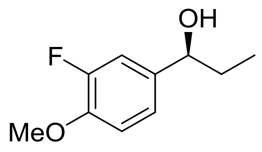

According to GP3, oxidation of boronic ester (*S*)-**30** (108 mg, 0.37 mmol, 1.0 equiv) afforded after purification by column chromatography (SiO_2, pentane/EtOAc 4:1) secondary benzylic alcohol (*S*)-**45** (61 mg, 0.33 mmol, 90%) as a colourless oil.

R_f (pentane/EtOAc 4:1) 0.18.

^{1}H NMR (400 MHz, $CDCl_3$) δ_H ppm 7.10 (dd, *J* = 12.2, 2.1 Hz, 1 H, H_{Ar}), 7.04 (m, 1 H, H_{Ar}), 6.93 (dd, J_{HF} = 8.4 Hz, J_{HH} = 8.4 Hz, 1 H, H_{Ar}), 4.55 (t, *J* = 6.6 Hz, 1 H, C*H*OH), 3.89 (s, 3 H, OCH_3), 1.86–1.66 (m, 3 H, CH_2+OH), 0.91 (t, *J* = 7.4 Hz, 3 H, CH_3).

^{13}C NMR (126 MHz, $CDCl_3$) δ_C ppm 152.3 (d, ^1J = 245.1 Hz, CF), 146.8 (d, ^2J = 11.4 Hz, *C*OMe), 137.7 (d, ^3J = 5.7 Hz, C), 121.6 (d, ^3J = 3.8 Hz, CH), 113.7 (d, ^2J = 18.1 Hz, CH), 113.1 (d, ^4J = 1.9 Hz, CH), 75.1 (CHOH), 56.3 (OCH_3), 31.8 (CH_2), 10.0 (CH_3).

^{19}F NMR (470 MHz, $CDCl_3$) δ_F ppm –135.0 (dd, *J* = 12.7, 8.5 Hz, CF).

ν_{max} (neat) = 3361, 2964, 1514, 1442, 1271, 1125, 1025, 871, 810, 760 cm^{-1}.

m/z (%) (CI^+) 185 ($[M+H]^+$, 34), 169 ($[M–Me]^+$, 17), 167 ($[M–OH]^+$, 57), 155 ($[M–Et]^+$, 7), 152 (10), 139 (10).

HRMS (CI$^+$) calcd. for $C_{10}H_{14}O_2F$ [M+H]$^+$ 185.0978, found 185.0981.

[α]$_D^{21}$ –31.0 (*c* 1.47, $CHCl_3$, for 96% ee).

HPLC separation conditions: Chiralpak IB column with guard, 3.0% *i*PrOH in hexane, flow rate 0.7 mL/min, 20 °C; t_R 21.7 min for (*R*)-enantiomer (minor) and t_R 23.1 min for (*S*)-enantiomer (major).
e.r. = 97.9:2.1.

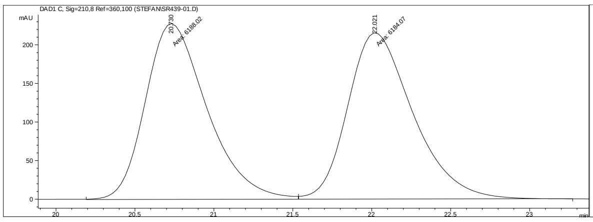

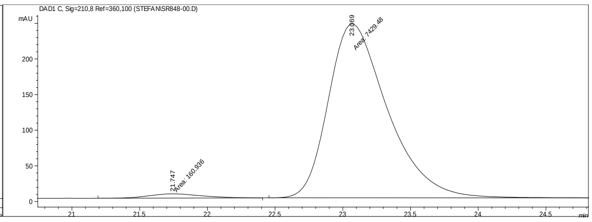

(*R*)-1-(3-Fluoro-4-methoxyphenyl)propan-1-ol ((*R*)-45)

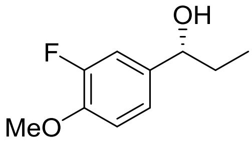

According to GP3, oxidation of boronic ester (*R*)-**30** (29 mg, 0.1 mmol, 1.0 equiv) afforded after purification by column chromatography (SiO_2, pentane/EtOAc 4:1) secondary benzylic alcohol (*R*)-**45** (16 mg, 87 µmol, 87%) as a colourless oil.

[α]$_D^{23}$ +31.0 (*c* 1.00, $CHCl_3$, for 96% ee).

HPLC separation conditions: Chiralpak IB column with guard, 3.0% *i*PrOH in hexane, flow rate 0.7 mL/min, 20 °C; t_R 24.4 min for (*R*)-enantiomer (major) and t_R 26.1 min for (*S*)-enantiomer (minor).
e.r. = 98.2:1.8.

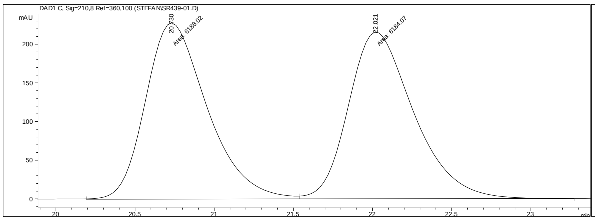

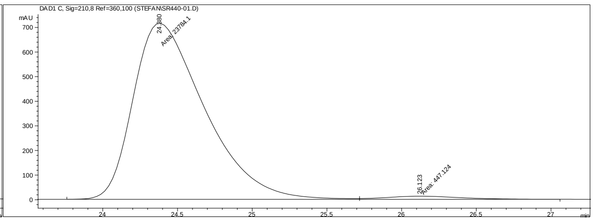

4,4,5,5-Tetramethyl-2-(pentan-3-yl)-1,3,2-dioxaborolane (10)

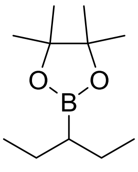

According to GP2B, propyl diisopropylcarbamate (**22**) (214 mg, 1.14 mmol, 1.0 equiv), TMEDA (221 µL, 1.49 mmol, 1.3 equiv), *s*BuLi (1.14 mL, 1.49 mmol, 1.3 equiv), and 2-ethyl-4,4,5,5-tetramethyl-1,3,2-dioxaborolane[19] (267 mg, 1.71 mmol, 1.5 equiv) in anhydrous diethyl ether (6.0 mL) afforded

after purification by flash chromatography (SiO_2, pentane/EtOAc 30:1) boronic ester **10** (120 mg, 0.61 mmol, 53%) as a colourless oil.

R_f (pentane/EtOAc 30:1) 0.28.

^{1}H NMR (400 MHz, $CDCl_3$) δ_H ppm 1.47–1.37 (m, 4 H, 2×CH_2), 1.26 (s, 12 H, 4×CH_3), 0.91 (t, J = 7.4 Hz, 6 H, 2×CH_3), 0.85 (m, 1 H, CHBpin).

^{13}C NMR (101 MHz, $CDCl_3$) δ_C ppm 82.8 (O*C*(CH_3)$_2$), 27.8 (br., CHBpin), 24.8 (CH_3), 24.0 (CH_2), 13.7 (CH_3).

^{11}B NMR (96 MHz, $CDCl_3$) δ_B ppm 33.5 (br. s).

ν_{max} (neat) = 2959, 1463, 1370, 1215, 1142, 967, 856 cm^{-1}.

m/z (%) (EI^+) 198 ($[M]^+$, 21), 183 ($[M–Me]^+$, 68), 112 (25).

HRMS (EI^+) calcd. for $C_{11}H_{23}O_2{}^{11}B$ $[M]^+$ 198.1791, found 198.1789.

5,5-dimethyl-2-(pentan-3-yl)-1,3,2-dioxaborinane (12)

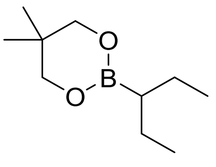

3-Bromopentane (2.00 g, 13.2 mmol, 1 equiv) was added to a mixture of CuI (250 mg, 1.32 mmol, 0.1 equiv), PPh_3 (449 mg, 1.72 mmol, 0.13 equiv), LiOMe (1.50 g, 39.6 mmol, 2 equiv) and B_2neo_2 (4.49 g, 19.8 mmol, 1.5 equiv) in DMF (13 mL) and stirred at 40 °C for 48 h. The reaction mixture was cooled to room temperature, diluted with pentane (200 mL) and filtered through a short plug of celite. The filtrate was washed with brine (4 × 50 mL), dried over $MgSO_4$ and concentrated under mild vacuum ~20 mbar. The crude residue was purified by Kugelrohr distillation (1 mbar, 110 °C) to afford **12** as a colourless oil (1.00 g, 5.40 mmol, 41%).

^{1}H NMR (400 MHz, $CDCl_3$) δ_H ppm 3.62 (s, 4H, 2×OCH_2), 1.46-1.28 (m, 4H, 2×C*H_2*CH_3), 5 0.91 (t, J = 7.4, 6H, 2×CH_2C*H_3*), 0.68 (p, J=6.8, 1H, CH).

^{13}C NMR (101 MHz, $CDCl_3$) δ_C ppm 71.8 (CH_2), 31.6 (C), 23.9 (CH_2), 21.9 (CH_3), 13.8 (CH_3).

^{11}B NMR (96 MHz, $CDCl_3$) δ_B ppm 30.0 (br. s).

ν_{max} (neat) = 2959, 2930, 2874, 1598, 1476, 1270, 1245, 1140, 699 cm^{-1}.

HRMS (EI^+) calcd. for $C_{10}H_{20}BO_2$ $[M–H]^+$ 183.1556, found 183.1559.

2.5 Preparation of tertiary boronic esters

General procedure 4A (GP4A). A solution of secondary benzylic carbamate (**5** or **6**) (1.00 mmol, 1.0 equiv) in anhydrous diethyl ether (3.0 mL) was cooled to −78 °C. *s*BuLi (1.00 mL, 1.30 mmol, 1.3 equiv) was added dropwise and the reaction mixture was stirred at this temperature for 1 h. A solution of the boronic ester (**7**, **8**, **9**, **10**, **11** or **12**) (1.50 mmol, 1.5 equiv) in anhydrous diethyl ether (1.5 mL) was added dropwise and the mixture was stirred for 2 h at −78 °C [*ate complex formation*]. Afterwards, the cooling bath was removed and the reaction mixture was stirred at ambient temperature until disappearance of the boron ate complex (5–8 ppm) monitored by ^{11}B NMR. The reaction mixture was then cooled to 0 °C and 1.0 M aq. KH_2PO_4 (2.0 mL) was added slowly. After stirring for 10 min, the phases were separated, and the aqueous phase was re-extracted with diethyl ether (4 × 20 mL). The combined organic phases were dried over anhydrous $MgSO_4$, filtered, and the solvent was removed *in vacuo*. The crude product was purified by column chromatography (SiO_2, pentane/EtOAc 30:1) to afford the tertiary boronic ester. If the starting pinacol boronic ester was still present, the mixture was dissolved in diethyl ether, the organic phase was washed several times with 0.5 M aq. NaOH solution, and the solvent was removed *in vacuo* to give the pure tertiary pinacol boronic ester.

General procedure 4B (GP4B). Following GP4A, 2 h after addition of the boronic ester a 1.0 M solution of $MgBr_2$ in anhydrous MeOH (1.30 mL, 1.30 mmol, 1.3 equiv) was added slowly at −78 °C. After 5 min, the cooling bath was removed and stirring was continued at room temperature.

4,4,5,5-Tetramethyl-2-(3-methyl-2-phenylbutan-2-yl)-1,3,2-dioxaborolane (13)

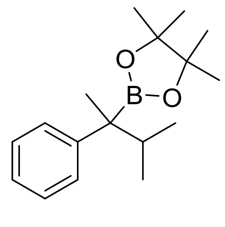

According to GP4A, 1-phenylethyl diisopropylcarbamate (**5**) (249 mg, 1.00 mmol, 1.0 equiv), *s*BuLi (1.00 mL, 1.30 mmol, 1.3 equiv), and 2-isopropyl-4,4,5,5-tetramethyl-1,3,2-dioxaborolane (**7**)[20] (255 mg, 1.50 mmol, 1.5 equiv) in anhydrous diethyl ether (4.5 mL) afforded after purification by flash chromatography (SiO_2, pentane/EtOAc 30:1) tertiary boronic ester **13** (222 mg, 0.81 mmol, 81%) as a colourless oil.

R$_f$ (pentane/EtOAc 30:1) 0.14.

^{1}H NMR (400 MHz, $CDCl_3$) δ_H ppm 7.40–7.34 (m, 2 H, H_{Ar}), 7.30–7.24 (m, 2 H, H_{Ar}), 7.13 (m, 1 H, H_{Ar}), 2.37 (sept, J = 6.8 Hz, 1 H, CH(CH_3)$_2$), 1.26 (s, 3 H, CH_3), 1.20 (s, 6 H, 2×CH_3), 1.17 (s, 6 H, 2×CH_3), 1.00 (d, J = 6.8 Hz, 3 H, CH_3), 0.59 (d, J = 6.8 Hz, 3 H, CH_3).

^{13}C NMR (126 MHz, $CDCl_3$) δ_C ppm 146.2 (C), 127.7 (CH), 127.3 (CH), 124.8 (CH), 83.1 (O*C*(CH_3)$_2$), 34.2 (CH), 24.6 (CH_3), 24.5 (CH_3), 20.3 (CH_3), 16.5 (CH_3), 13.9 (CH_3).

^{11}B NMR (96 MHz, $CDCl_3$) δ_B ppm 33.0 (br. s).

The analytical data match those reported in literature.[11]

4,4,5,5-Tetramethyl-2-(2-methyl-3-phenylpentan-3-yl)-1,3,2-dioxaborolane (14)

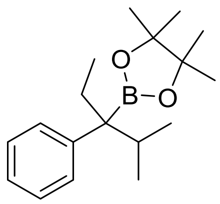

According to GP4B, 1-phenylpropyl diisopropylcarbamate (**6**) (263 mg, 1.00 mmol, 1.0 equiv), *s*BuLi (1.00 mL, 1.30 mmol, 1.3 equiv), 2-isopropyl-4,4,5,5-tetramethyl-1,3,2-dioxaborolane (**7**)[20] (255 mg, 1.50 mmol, 1.5 equiv), and $MgBr_2$ (1.30 mL, 1.0 M solution in anhydrous MeOH, 1.30 mmol, 1.3 equiv) in anhydrous diethyl ether (4.5 mL) furnished after purification by flash chromatography (SiO_2, pentane/EtOAc 30:1) tertiary boronic ester **14** (173 mg, 0.60 mmol, 60%) as a colourless oil.

R_f (pentane/EtOAc 30:1) 0.29.

^{1}H NMR (400 MHz, $CDCl_3$) δ_H ppm 7.32–7.23 (m, 4 H, H_{Ar}), 7.15 (m, 1 H, H_{Ar}), 2.14 (sept, J = 6.8 Hz, 1 H, C*H*(CH_3)$_2$), 1.98–1.80 (m, 2 H, CH_2), 1.33 (s, 6 H, 2×CH_3), 1.32 (s, 6 H, 2×CH_3), 0.92 (d, J = 6.8 Hz, 3 H, CH_3), 0.77 (t, J = 7.3 Hz, 3 H, CH_3), 0.77 (d, J = 6.8 Hz, 3 H, CH_3).

^{13}C NMR (101 MHz, $CDCl_3$) δ_C ppm 143.5 (C), 129.5 (CH), 127.3 (CH), 125.1 (CH), 83.2 (O*C*(CH_3)$_2$), 34.0 (CH), 28.1 (CH_2), 25.1 (CH_3), 24.9 (CH_3), 20.3 (CH_3), 18.5 (CH_3), 10.6 (CH_3).

^{11}B NMR (96 MHz, $CDCl_3$) δ_B ppm 34.0 (br. s).

The spectral data correspond to the literature known compound.[11]

2-(2,3-Diphenylbutan-2-yl)-4,4,5,5-tetramethyl-1,3,2-dioxaborolane (15)

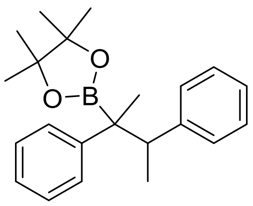

According to GP4B, carbamate **5** (249 mg, 1.00 mmol, 1.0 equiv), *s*BuLi (929 µL, 1.30 mmol, 1.3 equiv), 4,4,5,5-tetramethyl-2-(1-phenylethyl)-1,3,2-dioxaborolane (**8**) (348 mg, 1.50 mmol, 1.5 equiv), and $MgBr_2$ (1.30 mL, 1.0 M solution in anhydrous MeOH, 1.30 mmol, 1.3 equiv) in anhydrous diethyl ether (4.5 mL) gave after purification by flash chromatography (SiO_2, pentane/EtOAc 30:1) tertiary boronic ester **15** (312 mg, 0.93 mmol, 93%) as a colourless oil. The product was obtained as a mixture of diastereomers (*anti*:*syn* 86:14) [ratio of diastereomers for reaction without $MgBr_2$/MeOH (*anti*:*syn* 70:30)].

R_f (pentane/EtOAc 30:1) 0.20.

Analytical data of the major *anti* diastereomer.

^{1}H NMR (500 MHz, $CDCl_3$) δ_H ppm 7.26–7.15 (m, 5 H, H_{Ar}), 7.08–7.00 (m, 3 H, H_{Ar}), 6.84–6.75 (m, 2 H, H_{Ar}), 3.47 (q, *J* = 7.2 Hz, 1 H, CH), 1.45 (d, *J* = 7.2 Hz, 3 H, CH_3), 1.29 (s, 6 H, 2×CH_3), 1.28 (s, 3 H, CH_3), 1.25 (s, 6 H, 2×CH_3).

^{13}C NMR (126 MHz, $CDCl_3$) δ_C ppm 145.4 (C), 143.2 (C), 129.0 (CH), 127.6 (CH), 127.5 (CH), 126.8 (CH), 125.5 (CH), 125.2 (CH), 83.4 (O*C*(CH_3)$_2$), 47.0 (CH), 24.8 (CH_3), 24.6 (CH_3), 17.9 (CH_3), 15.1 (CH_3).

^{11}B NMR (96 MHz, $CDCl_3$) δ_B ppm 33.0 (br. s).

ν_{max} (neat) = 2975, 1600, 1451, 1306, 1144, 964, 852, 770, 699 cm^{-1}.

m/z (%) (CI$^+$) 337 ([M+H]$^+$, 100), 336 ([M]$^+$, 47), 321 ([M–Me]$^+$, 10), 259 ([M–Ph]$^+$, 58), 231 ([PhCCH$_3$Bpin]$^+$, 81), 209 ([M–Bpin]$^+$, 10), 131 ([PhC$_4$H$_6$]$^+$, 32), 105 ([PhC$_2$H$_4$]$^+$, 31).

HRMS (CI$^+$) calcd. for $C_{22}H_{30}O_2{}^{11}B$ [M+H]$^+$ 337.2339, found 337.2338.

Analytical data of the minor *syn* diastereomer.

^{1}H NMR (500 MHz, $CDCl_3$) δ_H ppm 7.40–7.28 (m, 4 H, H_{Ar}), 7.26–7.18 (m, 4 H, H_{Ar}), 7.13–7.08 (m, 2 H, H_{Ar}), 3.53 (q, *J* = 7.3 Hz, 1 H, CH), 1.29 (s, 3 H, CH_3), 1.18 (s, 6 H, 2×CH_3), 1.10 (s, 6 H, 2×CH_3), 1.06 (d, *J* = 7.0 Hz, 3 H, CH_3).

^{13}C NMR (126 MHz, $CDCl_3$) δ_C ppm 144.6 (C), 144.4 (C), 129.6 (CH), 128.4 (CH), 127.5 (CH), 127.2 (CH), 125.9 (CH), 125.3 (CH), 83.3 (O*C*(CH_3)$_2$), 45.9 (CH), 24.8 (CH_3), 24.3 (CH_3), 16.5 (CH_3), 15.0 (CH_3).

2-(2,3-Diphenylpentan-3-yl)-4,4,5,5-tetramethyl-1,3,2-dioxaborolane (16)

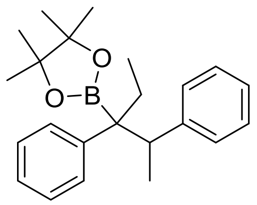

According to GP4B, carbamate **6** (263 mg, 1.00 mmol, 1.0 equiv), *s*BuLi (1.00 mL, 1.30 mmol, 1.3 equiv), 4,4,5,5-tetramethyl-2-(1-phenylethyl)-1,3,2-dioxaborolane (**8**) (348 mg, 1.50 mmol, 1.5 equiv), and $MgBr_2$ (1.30 mL, 1.0 M solution in anhydrous MeOH, 1.30 mmol, 1.3 equiv) in anhydrous diethyl ether (4.5 mL) afforded after purification by column chromatography (SiO_2, pentane/EtOAc 30:1) tertiary boronic ester **16** (200 mg, 0.57 mmol, 57%) as a colourless oil. The product was obtained as a mixture of diastereomers (*anti*:*syn* 95:5) [ratio of diastereomers for reaction without $MgBr_2$/MeOH (*anti*:*syn* 44:56)].

R_f (pentane/EtOAc 30:1) 0.31.

Analytical data for the major *anti* diastereomer.

^{1}H NMR (300 MHz, $CDCl_3$) δ_H ppm 7.25–7.11 (m, 8 H, H_{Ar}), 7.00–6.93 (m, 2 H, H_{Ar}), 3.28 (q, *J* = 7.2 Hz, 1 H, CH), 1.96 (m, 1 H, C*H*H), 1.82 (m, 1 H, CH*H*), 1.30 (s, 6 H, 2×CH_3), 1.27 (s, 6 H, 2×CH_3), 1.21 (d, *J* = 7.2 Hz, 3 H, CH_3), 0.80 (t, *J* = 7.3 Hz, 3 H, CH_3).

^{13}C NMR (126 MHz, $CDCl_3$) δ_C ppm 145.1 (C), 142.4 (C), 130.0 (CH), 129.5 (CH), 127.1 (CH), 127.0 (CH), 125.8 (CH), 125.3 (CH), 83.3 (O*C*(CH_3)$_2$), 47.2 (CH), 27.6 (CH_2), 25.5 (CH_3), 24.9 (CH_3), 16.8 (CH_3), 10.9 (CH_3).

^{11}B NMR (96 MHz, $CDCl_3$) δ_B ppm 33.2 (br. s).

ν_{max} (neat) = 2976, 1451, 1371, 1303, 1255, 1143, 968, 909, 855, 731, 700 cm^{-1}.

m/z (%) (CI$^+$) 351 ([M+H]$^+$, 47), 335 ([M–Me]$^+$, 12), 321 ([M–Et]$^+$, 4), 273 ([M–Ar]$^+$, 12), 245 ([PhC_3H_5Bpin]$^+$, 100), 217 (18), 173 (25), 147 (32), 145 (25), 117 (25), 105 ([PhC_2H_4]$^+$, 55), 101 (96), 91 ([PhCH_2]$^+$, 78).

HRMS (CI$^+$) calcd. for $C_{23}H_{32}O_2{}^{11}B$ [M+H]$^+$ 351.2495, found 351.2502.

Analytical data for the minor *syn* diastereomer.

^{1}H NMR (500 MHz, $CDCl_3$) δ_H ppm 7.25–7.06 (m, 6 H, H_{Ar}), 7.04–6.95 (m, 2 H, H_{Ar}), 6.57 (d, *J* = 7.6 Hz, 2 H, H_{Ar}), 3.26 (q, *J* = 7.3 Hz, 1 H, CH), 1.77 (q, *J* = 7.3 Hz, 2 H, CH_2), 1.37 (s, 6 H, 2×CH_3), 1.35 (s, 6 H, 2×CH_3), 1.26 (d, *J* = 7.0 Hz, 3 H, CH_3), 0.86 (t, *J* = 7.3 Hz, 3 H, CH_3).

^{13}C NMR (126 MHz, $CDCl_3$) δ_C ppm 143.2 (C), 139.9 (C), 131.0 (CH), 129.7 (CH), 126.8 (CH), 126.7 (CH), 125.8 (CH), 125.5 (CH), 83.5 (O*C*(CH_3)$_2$), 46.5 (CH), 27.9 (CH_2), 25.1 (CH_3), 24.9 (CH_3), 20.0 (CH_3), 10.7 (CH_3).

(2*S*,3*R*)-2,3-Diphenylpentan-3-ol ((2*S*,3*R*)-46)

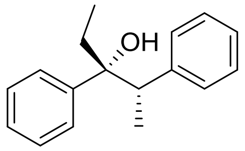

According to GP3, oxidation of 2-((2*R*,3*S*)-2,3-diphenylpentan-3-yl)-4,4,5,5-tetramethyl-1,3,2-dioxaborolane ((2*R*,3*S*)-**16**) (110 mg, 0.31 mmol, 1.0 equiv) afforded after purification by column chromatography (SiO_2, pentane/EtOAc 19:1) tertiary benzylic alcohol (2*S*,3*R*)-**46** (56 mg, 0.23 mmol, 74%) as a white solid.

mp 64–65 °C (diethyl ether).

R_f (pentane/EtOAc 19:1) 0.27.

^{1}H NMR (400 MHz, $CDCl_3$) δ_H ppm 7.45–7.22 (m, 10 H, H_{Ar}), 3.13 (q, *J* = 7.1 Hz, 1 H, CH), 1.87 (dq, *J* = 14.5, 7.3 Hz, 1 H, C*H*H), 1.54 (br. s, 1 H, OH), 1.41 (dq, *J* = 14.5, 7.3 Hz, 1 H, CH*H*), 1.06 (d, *J* = 7.1 Hz, 3 H, CH_3), 0.56 (t, *J* = 7.3 Hz, 3 H, CH_3).

^{13}C NMR (101 MHz, $CDCl_3$) δ_C ppm 144.5 (C), 143.0 (C), 129.3 (CH), 128.1 (CH), 127.9 (CH), 126.6 (CH), 126.2 (CH), 125.8 (CH), 78.9 (COH), 50.4 (CH), 34.3 (CH_2), 16.0 (CH_3), 7.8 (CH_3).

ν_{max} (neat) = 3582, 2970, 2917, 1493, 1451, 1148, 963, 903, 700 cm^{-1}.

m/z (%) (ESI^+) 263 ($[M+Na]^+$, 100), 223 ($[M–OH]^+$, 13), 167 (14), 105 ($[PhC_2H_4]^+$, 15).

HRMS (ESI^+) calcd. for $C_{17}H_{20}ONa$ $[M+Na]^+$ 263.1406, found 263.1404.

$[\alpha]_D^{20}$ –21.0 (*c* 1.00, $CHCl_3$, for 99% ee).

SFC separation conditions: Chiralpak IA column, eluent: 90% CO_2, 5% hexane, 5% *i*PrOH, flow rate 4.0 mL/min, 39.8 °C, 123 bar; t_R 3.96 min for (*S*,*R*)-enantiomer (major) and t_R 5.67 min for (*R*,*S*)-enantiomer (minor).
e.r. = 99.8:0.2.

Run Information

Instrument Method	Inj. Vol.	Solvent	Column	Sample	Well Location	Temp.	Flow	% Modifier	Pressure
iso 10%, 4 ml per min, 125 bar	10	50% IPA/Hex	IA	SR866b	21E	39.8	4	10	123

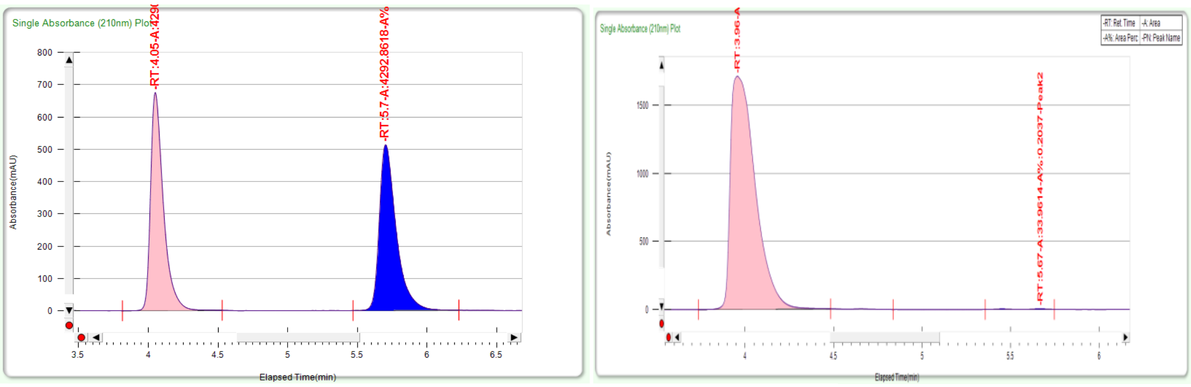

Peak Information

Peak No	% Area	Area	Ret. Time	Height	Cap. Factor
1	99.7963	16640.588 3	3.96 min	1709.2161	0
2	0.2037	33.9614	5.67 min	3.2357	0

(2*R*,3*R*)-2,3-Diphenylpentan-3-ol ((2*R*,3*R*)-46)

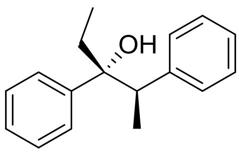

According to GP3, oxidation of 2-((2*S*,3*S*)-2,3-diphenylpentan-3-yl)-4,4,5,5-tetramethyl-1,3,2-dioxaborolane ((2*S*,3*S*)-**16**) (69 mg, 0.20 mmol, 1.0 equiv) afforded after purification by column chromatography (SiO_2, pentane/EtOAc 19:1) tertiary benzylic alcohol

(2*R*,3*R*)-**46** (44 mg, 0.18 mmol, 93%) as a colourless oil.

R$_f$ (pentane/EtOAc 19:1) 0.20.

^{1}H NMR (400 MHz, $CDCl_3$) δ_H ppm 7.29–7.23 (m, 2 H, H$_{Ar}$), 7.22–7.12 (m, 6 H, H$_{Ar}$), 6.98–6.92 (m, 2 H, H$_{Ar}$), 3.18 (q, *J* = 7.1 Hz, 1 H, CH), 2.04 (dq, *J* = 14.4, 7.3 Hz, 1 H, C*H*H), 1.92 (dq, *J* = 14.4, 7.3 Hz, 1 H, CH*H*), 1.76 (br. s, 1 H, OH), 1.35 (d, *J* = 7.1 Hz, 3 H, CH_3), 0.73 (t, *J* = 7.3 Hz, 3 H, CH_3).

^{13}C NMR (101 MHz, $CDCl_3$) δ_C ppm 144.1 (C), 142.4 (C), 129.3 (CH), 127.6 (CH), 127.5 (CH), 126.4 (CH), 126.3 (CH), 126.2 (CH), 79.1 (COH), 50.7 (CH), 30.8 (CH_2), 15.2 (CH_3), 7.9 (CH_3).

ν_{max} (neat) = 3570, 2971, 1493, 1451, 1137, 963, 910, 771, 754, 699 cm^{-1}.

m/z (%) (ESI$^+$) 263 ([M+Na]$^+$, 100), 223 ([M–OH]$^+$, 17), 146 (19), 105 ([PhC_2H_4]$^+$, 15).

HRMS (ESI$^+$) calcd. for $C_{17}H_{20}ONa$ [M+Na]$^+$ 263.1406, found 263.1407.

$[\alpha]_D^{21}$ +92.0 (*c* 1.29, $CHCl_3$, for 99% ee).

SFC separation conditions: Chiralpak IA column, eluent: 95% CO_2, 4.5% hexane, 0.5% *i*PrOH, flow rate 4.0 mL/min, 41.5 °C, 124 bar; t_R 10.51 min for (*R*,*R*)-enantiomer (major) and t_R 11.84 min for (*S*,*S*)-enantiomer (minor).
e.r. = 99.9:0.1.

Run Information

Instrument Method	Inj. Vol.	Solvent	Column	Sample	Well Location	Temp.	Flow	% Modifier	Pressure
iso 5%, 4 ml per min, 125 bar	10	10% IPA/Hex	IA	SR864a	21C	41.5	4	5	124

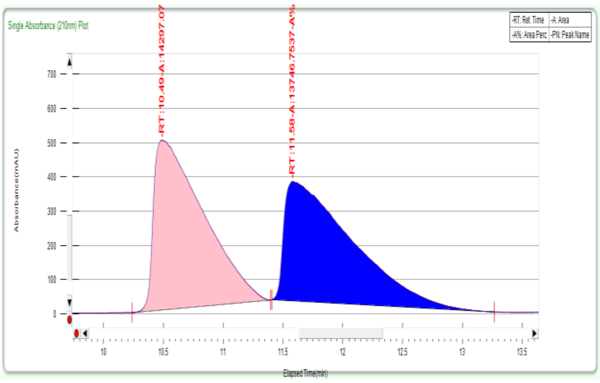

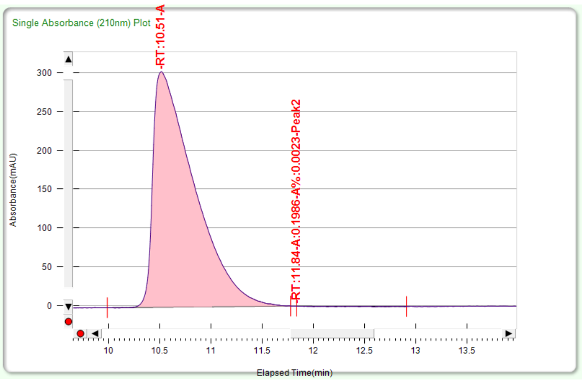

Peak Information

Peak No	% Area	Area	Ret. Time	Height	Cap. Factor
1	99.9977	8492.246	10.51 min	303.4144	0.2387
2	0.0023	0.1986	11.84 min	0.0844	0.3946

2-(2,3-Diphenylpentan-2-yl)-4,4,5,5-tetramethyl-1,3,2-dioxaborolane (17)

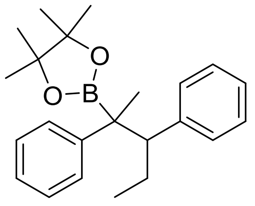

13

According to GP4B, carbamate **5** (54 mg, 0.22 mmol, 1.0 equiv), *s*BuLi (217 µL, 0.28 mmol, 1.3 equiv), 4,4,5,5-tetramethyl-2-(1-phenylpropyl)-1,3,2-dioxaborolane (**9**) (80 mg, 0.33 mmol, 1.5 equiv), and $MgBr_2$ (282 µL, 1.0 M solution in anhydrous MeOH, 0.28 mmol, 1.3 equiv) in anhydrous diethyl ether (1.5 mL) afforded after purification by column chromatography (SiO_2, pentane/EtOAc 30:1) tertiary boronic ester **17** (51 mg, 0.15 mmol, 67%) as a colourless oil. The product was obtained as a mixture of diastereomers (*anti*:*syn* 70:30) [ratio of diastereomers for reaction without $MgBr_2$/MeOH (*anti*:*syn* 69:31)].

R_f (pentane/EtOAc 30:1) 0.20.

Analytical data for the major *anti* diastereomer.

^{1}H NMR (400 MHz, $CDCl_3$) δ_H ppm 7.25–7.12 (m, 5 H, H_{Ar}), 7.05–7.01 (m, 3 H, H_{Ar}), 6.85–6.79 (m, 2 H, H_{Ar}), 3.11 (dd, *J* = 11.7, 2.7 Hz, 1 H, CH), 1.97 (m, 1 H, C*H*H), 1.76 (m, 1 H, CH*H*), 1.29 (s, 3 H, CH_3), 1.28 (s, 6 H, 2×CH_3), 1.24 (s, 6 H, 2×CH_3), 0.76 (t, *J* = 7.2 Hz, 3 H, CH_3).

^{13}C NMR (126 MHz, $CDCl_3$) δ_C ppm 145.4 (C), 140.9 (C), 129.6 (CH), 127.49 (CH), 127.48 (CH), 126.9 (CH), 125.5 (CH), 125.0 (CH), 83.4 (O*C*(CH_3)$_2$), 55.7 (CH), 25.4 (CH_2), 24.73 (CH_3), 24.67 (CH_3), 16.0 (CH_3), 13.2 (CH_3).

^{11}B NMR (96 MHz, $CDCl_3$) δ_B ppm 33.0 (br. s).

ν_{max} (neat) = 2975, 1600, 1451, 1306, 1144, 966, 849, 775, 699 cm^{-1}.

m/z (%) (ESI$^+$) 373 ([M+Na]$^+$, 100).

HRMS (ESI$^+$) calcd. for $C_{23}H_{31}O_2{}^{11}BNa$ [M+Na]$^+$ 373.2309, found 373.2297.

Analytical data for the minor *syn* diastereomer.

^{1}H NMR (400 MHz, $CDCl_3$) δ_H ppm 7.41–7.37 (m, 2 H, H_{Ar}), 7.32–7.28 (m, 2 H, H_{Ar}), 7.25–7.12 (m, 4 H, H_{Ar}), 7.09–7.05 (m, 2 H, H_{Ar}), 3.18 (dd, *J* = 12.0, 2.9 Hz, 1 H, CH), 1.60 (m, 1 H, C*H*H), 1.44 (m, 1 H, CH*H*), 1.32 (s, 3 H, CH_3), 1.14 (s, 6 H, 2×CH_3), 1.03 (s, 6 H, 2×CH_3), 0.58 (t, *J* = 7.3 Hz, 3 H, CH_3).

^{13}C NMR (126 MHz, $CDCl_3$) δ_C ppm 144.7 (C), 142.1 (C), 130.5 (CH), 128.3 (CH), 127.5 (CH), 127.2 (CH), 125.9 (CH), 125.2 (CH), 83.2 (O*C*(CH_3)$_2$), 54.6 (CH), 24.7 (CH_3), 24.2 (CH_3), 22.7 (CH_2), 16.9 (CH_3), 12.8 (CH_3).

2-(2,3-diphenylpentan-2-yl)-5,5-dimethyl-1,3,2-dioxaborinane (21)

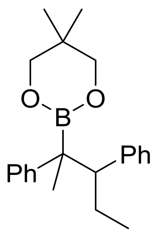

According to GP4A, 1-phenylethyl diisopropylcarbamate **5** (125 mg, 0.5 mmol, 1 equiv), *s*BuLi (0.5 mL, 0.65 mmol, 1.3 equiv) and 5,5-dimethyl-2-(1-phenylpropyl)-1,3,2-dioxaborinane **11** (174 mg, 0.75 mmol, 1.5 equiv) in anhydrous diethyl ether (3.25 mL) afforded after purification by flash chromatography (SiO_2, pentane/Et_2O 9:1) tertiary boronic ester **21** (119 mg, 0.36 mmol, 71%) as a colourless oil. The product was obtained as a mixture of diastereomers (anti:syn 91:9).

R_f (pentane/Et_2O 9:1) 0.50.

Analytical data of the major *anti* diastereomer.

^{1}H NMR (400 MHz, $CDCl_3$) δ_H ppm 7.24-7.09 (m, 4 H, H_{Ar}), 7.06-6.97 (m, 4 H, H_{Ar}), 6.84-6.75 (m, 2 H, H_{Ar}), 3.66 (d, *J* = 11.2, 2H, 2×OC*H*H), 3.62 (d, *J* = 11.2, 2H, 2×OCH*H*), 3.16 (dd, *J*=11.7, 2.8, 1H, C*H*), 1.93 (ddq, J=14.2, 11.7, 7.3, 1H, C*H*H), 1.75 (dqd, *J*=14.2, 7.3, 2.8, 1H, CH*H*), 1.23 (s, 3H, CH_3), 0.92 (s, 6H, $C(CH_3)_2$), 0.74 (t, *J* = 7.3, 3H, CH_2CH_3).

^{13}C NMR (101 MHz, $CDCl_3$) δ_C ppm 146.4 (C), 141.3 (C), 129.7 (CH), 127.5 (CH), 127.3 (CH), 126.8 (CH), 125.3 (CH), 124.8 (CH), 72.1 (CH_2), 55.2 (CH), 31.5 (C), 25.6 (CH_2), 22.1f (CH_3), 15.7 (CH_3), 13.3 (CH_3).

^{11}B NMR (96 MHz, $CDCl_3$) δ_B ppm 28.7 (br. s).

Analytical data of the minor *syn* diastereomer.

^{1}H NMR (400 MHz, $CDCl_3$) δ_H ppm 7.42-7.09 (m, 10 H, HAr), 3.49 (d, J=11.0, 2H), 3.44 (d, J=11.0, 2H), 3.25 (dd, J=12.0, 2.9, 1H), 1.58 (ddq, *J*=14.7, 12.0, 7.2, 1H), 1.39 (dqd, *J*=14.7, 7.2, 2.9, 1H), 1.27 (s, 3H), 0.73 (s, 6H), 0.57 (t, J=7.2, 1H).

^{13}C NMR (101 MHz, $CDCl_3$) δ_C ppm 146.0 (C), 142.8 (C), 130.4 (CH), 128.1 (CH), 127.5 (CH), 127.3 (CH), 125.7 (CH), 124.9 (CH), 71.9 (CH_2), 53.9 (CH), 31.4 (C), 22.6 (CH_2), 21.8 (CH_3), 16.7 (CH_3), 13.0 (CH_3).

2-((2*S*,3*R*)-2,3-diphenylpentan-2-yl)-5,5-dimethyl-1,3,2-dioxaborinane ((2*S*,3*R*)-21)

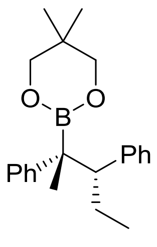

According to GP4A, (*S*)-1-phenylethyl diisopropylcarbamate (*S*)-**5** (107 mg, 0.43 mmol, 1 equiv), *s*BuLi (0.43 mL, 0.56 mmol, 1.3 equiv) and (*S*)-5,5-dimethyl-2-(1-phenylpropyl)-1,3,2-dioxaborinane (150 mg, 0.75 mmol, 1.5 equiv) in anhydrous diethyl ether (2.80 mL) afforded after purification by flash chromatography (SiO_2, pentane/Et_2O 9:1) tertiary boronic ester (2*S*,3*R*)-**21** (100

mg, 0.30 mmol, 69%, >99:1 dr, >99:1 er) as a white solid.

R$_f$ (pentane/Et_2O 9:1) 0.50.

mp 139–140 °C (pentane/Et_2O).

[α]$_D^{22}$ −115 (*c* 1, $CHCl_3$).

^{1}H NMR (400 MHz, $CDCl_3$) δ_H ppm 7.24-7.09 (m, 4 H, H_{Ar}), 7.06-6.97 (m, 4 H, H_{Ar}), 6.84-6.75 (m, 2 H, H_{Ar}), 3.66 (d, *J* = 11.2, 2H, 2×OC*H*H), 3.62 (d, *J* = 11.2, 2H, 2×OCH*H*), 3.16 (dd, *J*=11.7, 2.8, 1H, C*H*), 1.93 (ddq, J=14.2, 11.7, 7.3, 1H, C*H*H), 1.75 (dqd, *J*=14.2, 7.3, 2.8, 1H, CH*H*), 1.23 (s, 3H, CH_3), 0.92 (s, 6H, $C(CH_3)_2$), 0.74 (t, *J* = 7.3, 3H, CH_2CH_3).

^{13}C NMR (101 MHz, $CDCl_3$) δ_C ppm 146.4 (C), 141.3 (C), 129.7 (CH), 127.5 (CH), 127.3 (CH), 126.8 (CH), 125.3 (CH), 124.8 (CH), 72.1 (CH_2), 55.2 (CH), 31.5 (C), 25.6 (CH_2), 22.1 (CH_3), 15.7 (CH_3), 13.3 (CH_3).

ν_{max} (neat) = 3027, 2962, 2932, 2873, 1600, 1476, 1413, 1375, 1266, 1246, 1126, 700 cm^{-1}.

HRMS (ESI) calcd. for $C_{22}H_{29}BO_2Na$ $[M+Na]^+$ 359.2157, found 359.2153.

HPLC separation conditions: Chiralpak IA column with guard, 5.0% *i*PrOH in hexane, flow rate 1 mL/min, 20 °C; Major diasteroisomer: t_R 6.5 min for (*S*,*R*)-enantiomer (major) 7.2 min for (*R*,*S*)-enantiomer (minor). Minor diasteroisomer: t_R 6.8 min for (*S*,*S*)-enantiomer, 7.8 min for (*R*,*R*)-enantiomer.

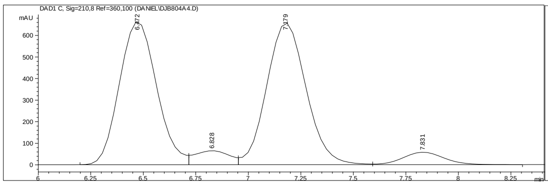
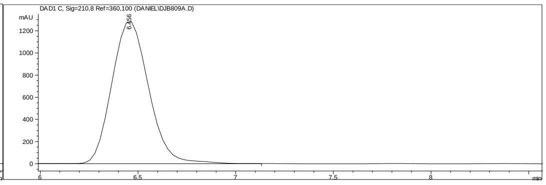

2-((2*R*,3*R*)-2,3-diphenylpentan-2-yl)-5,5-dimethyl-1,3,2-dioxaborinane ((2*R*,3*R*)-21)

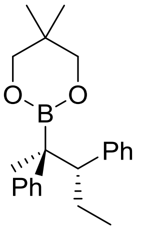

According to GP4A, (*R*)-1-phenylethyl diisopropylcarbamate (*R*)-**5** (107 mg, 0.43 mmol, 1 equiv), *s*BuLi (0.43 mL, 0.56 mmol, 1.3 equiv) and (*S*)-5,5-dimethyl-2-(1-phenylpropyl)-1,3,2-dioxaborinane (150 mg, 0.75 mmol, 1.5 equiv) in anhydrous diethyl ether (2.80 mL) afforded after purification by flash chromatography (SiO_2, pentane/Et_2O 9:1) tertiary boronic ester **xx** (90 mg, 0.27 mmol, 63%, 93:7 dr, >99:1 er) as a viscous oil.

R$_f$ (pentane/Et_2O 9:1) 0.50.

[α]$_D^{22}$−24 (*c* 1, $CHCl_3$).

^{1}H NMR (400 MHz, $CDCl_3$) δ_H ppm 7.42-7.09 (m, 10 H, HAr), 3.49 (d, J=11.0, 2H), 3.44 (d, J=11.0, 2H), 3.25 (dd, J=12.0, 2.9, 1H), 1.58 (ddq, *J*=14.7, 12.0, 7.2, 1H), 1.39 (dqd, *J*=14.7, 7.2, 2.9, 1H), 1.27 (s, 3H), 0.73 (s, 6H), 0.57 (t, J=7.2, 1H).

^{13}C NMR (101 MHz, $CDCl_3$) δ_C ppm 146.0 (C), 142.8 (C), 130.4 (CH), 128.1 (CH), 127.5 (CH), 127.3 (CH), 125.7 (CH), 124.9 (CH), 71.9 (CH_2), 53.9 (CH), 31.4 (C), 22.6 (CH_2), 21.8 (CH_3), 16.7 (CH_3), 13.0 (CH_3).

ν_{max} (neat) = 3028, 2960, 2932, 2872, 1599, 1476, 1414, 1266, 1248, 1136, 701 cm^{-1}.

HRMS (ESI) calcd. for $C_{22}H_{29}BO_2Na$ $[M+Na]^+$ 359.2157, found 359.2153.

HPLC separation conditions: Chiralpak IA column with guard, 5.0% *i*PrOH in hexane, flow rate 1 mL/min, 20 °C; Major diasteroisomer: t_R 6.8 min for (*S*,*S*)-enantiomer (minor) 7.8 min for (*R*,*R*)-enantiomer (major). Minor diasteroisomer: t_R 6.5 min for (*S*,*R*)-enantiomer 7.2 min for (*R*,*S*)-enantiomer .

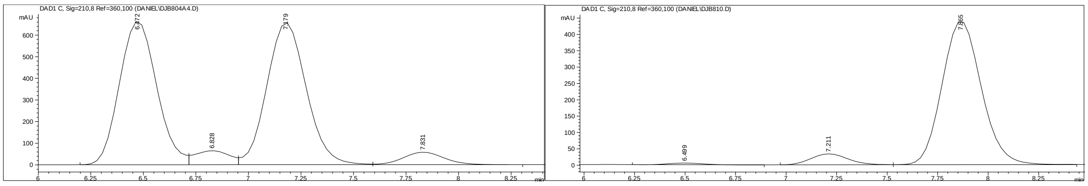

2-(3,4-Diphenylhexan-3-yl)-4,4,5,5-tetramethyl-1,3,2-dioxaborolane (18)

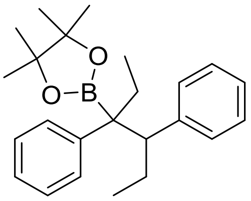

According to GP4B, 1-phenylpropyl diisopropylcarbamate (**6**) (51 mg, 0.20 mmol, 1.0 equiv), *s*BuLi (195 µL, 0.25 mmol, 1.3 equiv), 4,4,5,5-tetramethyl-2-(1-phenylpropyl)-1,3,2-dioxaborolane (**9**) (72 mg, 0.29 mmol, 1.5 equiv), and $MgBr_2$ (253 µL, 1.0 M solution in anhydrous MeOH, 0.25 mmol, 1.3 equiv) in anhydrous diethyl ether (1.5 mL) afforded after purification by column chromatography (SiO_2, pentane/EtOAc 30:1) tertiary boronic ester **18** (22 mg, 62 µmol, 32%) as a colourless oil as an inseparable mixture with starting material **9**. The product was obtained as a mixture of diastereomers (*anti*:*syn* 65:35) [ratio of diastereomers for reaction without $MgBr_2$/MeOH (*anti*:*syn* 43:57)].

R_f (pentane/EtOAc 30:1) 0.18.

2-(3,4-diphenylhexan-3-yl)-5,5-dimethyl-1,3,2-dioxaborinane 22

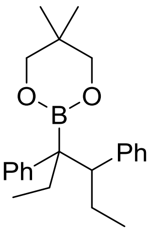

According to GP4A, 1-phenylpropyl diisopropylcarbamate **6** (132 mg, 0.5 mmol, 1 equiv), *s*BuLi (0.5 mL, 0.65 mmol, 1.3 equiv) and 5,5-dimethyl-2-(1-phenylpropyl)-1,3,2-dioxaborinane (174 mg, 0.75 mmol, 1.5 equiv) in anhydrous diethyl ether (3.25 mL) afforded after purification by flash

chromatography (SiO_2, pentane/Et_2O 9:1) tertiary boronic ester **22** (126 mg, 0.36 mmol, 72%) as a colourless oil. The product was obtained as a mixture of diastereomers (anti:syn 83:17).

R_f (pentane/Et_2O 9:1) 0.50.

Analytical data of the major *anti* diastereomer.

^{1}H NMR (400 MHz, $CDCl_3$) δ_H ppm 7.30 – 6.97 (m, 10H, H_{Ar}), 3.62 (s, 4H, 2×OCH_2), 2.94 (dd, *J* = 10.9, 4.0, 1H, CH), 1.78 – 1.58 (m, 4H, 2×CH_2), 1.00 (s, 6H, $C(CH_3)_2$), 0.60 (t, *J* = 7.4, 1H, CH_2CH_3), 0.59 (t, *J* = 7.4, 1H, CH_2CH_3).

^{13}C NMR (101 MHz, $CDCl_3$) δ_C ppm 144.7 (C), 143.3 (C), 129.8 (CH), 129.5 (CH), 127.4 (CH), 127.3 (CH), 125.8 (CH), 125.0 (CH), 71.8 (CH_2), 56.5 (CH), 31.2 (C), 30.0 (CH_2), 23.9 (CH_2), 22.3 (CH_3), 13.1 (CH_3), 10.9 (CH_3).

^{11}B NMR (96 MHz, $CDCl_3$) δ_B ppm 30.0 (br. s).

Analytical data of the minor *syn* diastereomer.

^{1}H NMR (400 MHz, $CDCl_3$) δ_H ppm 7.25 – 6.96 (m, 8H), 6.47 (d, *J* = 7.1, 2H), 3.68 (s, 4H), 2.96 (dd, *J* = 12.1, 2.8, 0H), 1.82 (dqd, *J* = 14.7, 7.2, 2.8, 1H), 1.64 (t, *J* = 7.5, 2H), 1.52 (ddq, *J* = 14.7, 12.1, 7.2, 1H), 1.05 (s, 6H), 0.83 (t, *J* = 7.4, 3H), 0.59 (t, *J* = 7.2, 3H).

^{13}C NMR (101 MHz, $CDCl_3$) δ_C ppm 141.5 (C), 141.3 (C), 131.0 (CH), 130.3 (CH), 126.7 (CH), 126.58 (CH), 125.62 (CH), 125.3 (CH), 72.1 (CH_2), 54.6 (CH), 31.5 (C), 28.0 (CH_2), 27.8 (CH_2), 22.3 (CH_3), 13.1 (CH_3), 10.8 (CH_3).

2-((3*S*,4*R*)-3,4-diphenylhexan-3-yl)-5,5-dimethyl-1,3,2-dioxaborinane ((3*S*,4*R*)-22)

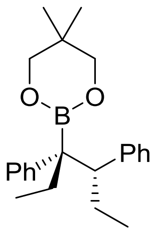

According to GP4A, (*S*)-1-phenylpropyl diisopropylcarbamate (*S*)-**6** (113 mg, 0.43 mmol, 1 eq.), *s*BuLi (0.43 mL, 0.56 mmol, 1.3 eq.) and (*S*)-5,5-dimethyl-2-(1-phenylpropyl)-1,3,2-dioxaborinane (150 mg, 0.65 mmol, 1.5 eq.) in anhydrous diethyl ether (2.80 mL) afforded after purification by flash chromatography (SiO_2, pentane/Et_2O 9:1) tertiary boronic ester (*S*,*R*)-**22** (103 mg, 0.30 mmol, 69%, 98:2 dr, >99:1 er) as a colourless oil.

R_f (pentane/Et_2O 9:1) 0.50.

$[\alpha]_D^{22}$ +2 (*c* 1, $CHCl_3$).

^{1}H NMR (400 MHz, $CDCl_3$) δ_H ppm 7.30 – 6.97 (m, 10H, H_{Ar}), 3.62 (s, 4H, 2×OCH_2), 2.94 (dd, *J* = 10.9, 4.0, 1H, CH), 1.78 – 1.58 (m, 4H, 2×CH_2), 1.00 (s, 6H, $C(CH_3)_2$), 0.60 (t, *J* = 7.4, 1H, CH_2CH_3), 0.59 (t, *J* = 7.4, 1H, CH_2CH_3).

^{13}C NMR (101 MHz, $CDCl_3$) δ_C ppm 144.7 (C), 143.3 (C), 129.8 (CH), 129.5 (CH), 127.4 (CH), 127.3 (CH), 125.8 (CH), 125.0 (CH), 71.8 (CH_2), 56.5 (CH), 31.2 (C), 30.0 (CH_3), 23.9 (CH_2), 22.3 (CH_3), 13.1 (CH_3), 10.9 (CH_3).

ν_{max} (neat) = 3025, 2962, 2874, 1600, 1475, 1412, 1245, 756, 701 cm^{-1}.

HRMS (ESI) calcd. for $C_{23}H_{31}BO_2Na$ $[M+Na]^+$ 373.2313, found 373.2325.

HPLC separation conditions: Chiralpak IA column with guard, 2.0% *i*PrOH in hexane, flow rate 1 mL/min, 20 °C; Major diasteroisomer: t_R 6.4 min for (*S*,*R*)-enantiomer (major) 9.5 min for (*R*,*S*)-enantiomer (minor). Minor diasteroisomer: t_R 7.9 min for (*S*,*S*)-enantiomer and 8.8 min for (*R*,*R*)-enantiomer.

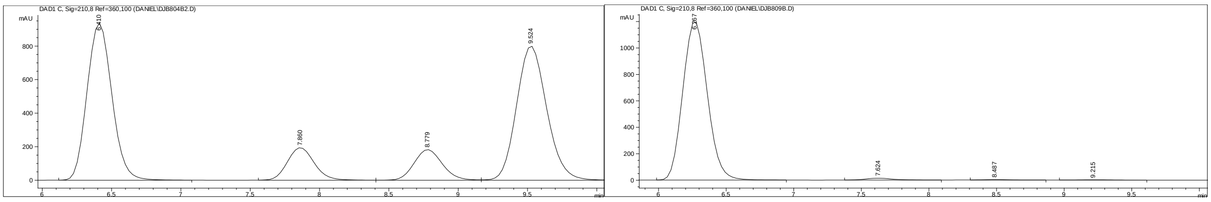

2-((3*R*,4*R*)-3,4-diphenylhexan-3-yl)-5,5-dimethyl-1,3,2-dioxaborinane ((3*R*,4*R*)-22)

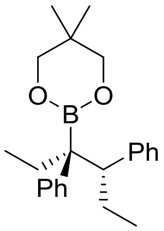

According to GP4A, (*R*)-1-phenylpropyl diisopropylcarbamate (R)-**5** (113 mg, 0.43 mmol, 1 eq.), *s*BuLi (0.43 mL, 0.56 mmol, 1.3 eq.) and (*S*)-5,5-dimethyl-2-(1-phenylpropyl)-1,3,2-dioxaborinane (150 mg, 0.65 mmol, 1.5 eq.) in anhydrous diethyl ether (2.80 mL) afforded after purification by flash chromatography (SiO_2, pentane/Et_2O 9:1) tertiary boronic ester (*R*,*R*)-**22** (95 mg, 0.27 mmol, 63%, 90:10 dr, >99:1 er) as a colourless oil.

R_f (pentane/Et_2O 9:1) 0.50.

^{1}H NMR (400 MHz, $CDCl_3$) δ_H ppm 7.25 – 6.96 (m, 8H), 6.47 (d, *J* = 7.1, 2H), 3.68 (s, 4H), 2.96 (dd, *J* = 12.1, 2.8, 0H), 1.82 (dqd, *J* = 14.7, 7.2, 2.8, 1H), 1.64 (t, *J* = 7.5, 2H), 1.52 (ddq, *J* = 14.7, 12.1, 7.2, 1H), 1.05 (s, 6H), 0.83 (t, *J* = 7.4, 3H), 0.59 (t, *J* = 7.2, 3H).

^{13}C NMR (101 MHz, $CDCl_3$) δ_C ppm 141.5 (C), 141.3 (C), 131.0 (CH), 130.3 (CH), 126.7 (CH), 126.58 (CH), 125.62 (CH), 125.3 (CH), 72.1 (CH_2), 54.6 (CH), 31.5 (C), 28.0 (CH_2), 27.8 (CH_2), 22.3 (CH_3), 13.1 (CH_3), 10.8 (CH_3).

ν_{max} (neat) = 3025, 2961, 2932, 2873, 1600, 1476, 1412, 1243, 1137, 701 cm^{-1}.

HRMS (ESI) calcd. for $C_{23}H_{31}BO_2Na$ $[M+Na]^+$ 373.2313, found 373.2325.

$[\alpha]_D^{22}$ +55 (*c* 1, $CHCl_3$).

HPLC separation conditions: Chiralpak IA column with guard, 2.0% *i*PrOH in hexane, flow rate 1 mL/min, 20 °C; Major diasteroisomer: t_R 7.9 min for (*S*,*S*)-enantiomer (minor) and 8.8 min for (*R*,*R*)-enantiomer (major). Minor diasteroisomer: t_R 6.4 min for (*S*,*R*)-enantiomer, 9.5 min for (*R*,*S*)-enantiomer.

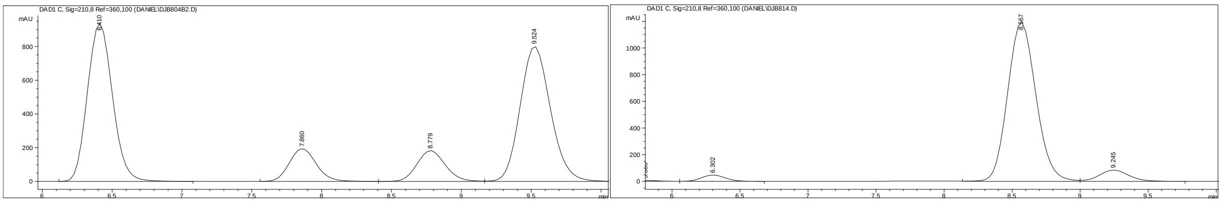

2-(3-Ethyl-2-phenylpentan-2-yl)-4,4,5,5-tetramethyl-1,3,2-dioxaborolane (19)

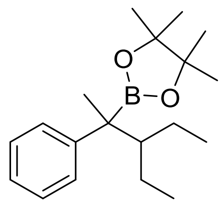

According to GP4B, 1-phenylethyl diisopropylcarbamate (**5**) (50 mg, 0.20 mmol, 1.0 equiv), *s*BuLi (200 µL, 0.26 mmol, 1.3 equiv), and 4,4,5,5-tetramethyl-2-(pentan-3-yl)-1,3,2-dioxaborolane (**10**) (59 mg, 0.30 mmol, 1.5 equiv), in anhydrous diethyl ether (1.5 mL) afforded after purification by column chromatography (SiO_2, pentane/EtOAc 30:1) tertiary boronic ester **19** (20 mg, 66 µmol, 33%) as a colourless oil. Before purification the yield of **19** in the crude reaction mixture was determined by ^{1}H NMR spectroscopy with 1,3,5-trimethoxybenzene as internal standard and was measured to be 41%.

$\mathbf{R_f}$ (pentane/EtOAc 30:1) 0.27.

$\mathbf{^1H}$ **NMR** (500 MHz, $CDCl_3$) δ_H ppm 7.43–7.38 (m, 2 H, H_{Ar}), 7.29–7.24 (m, 2 H, H_{Ar}), 7.12 (tt, *J* = 7.3, 1.2 Hz, 1 H, H_{Ar}), 1.89 (tt, *J* = 7.7, 3.7 Hz, 1 H, C*H*$(CH_2)_2$), 1.43–1.32 (m, 2 H, CH_2), 1.26 (s, 3 H, CH_3), 1.16 (s, 6 H, 2×CH_3), 1.13 (s, 6 H, 2×CH_3), 1.10 (m, 1 H, C*H*H), 1.05 (t, *J* = 7.4 Hz, 3 H, CH_3), 0.98 (m, 1 H, CH*H*), 0.69 (t, *J* = 7.4 Hz, 3 H, CH_3).

$\mathbf{^{13}C}$ **NMR** (126 MHz, $CDCl_3$) δ_C ppm 146.2 (C), 127.7 (CH), 127.5 (CH), 124.7 (CH), 83.1 (O*C*$(CH_3)_2$), 48.1 (CH), 28.1 (CH_2), 24.5 (CH_3), 24.4 (CH_3), 23.3 (CH_2), 14.7 (CH_3), 14.5 (CH_3), 14.3 (CH_3).

$\mathbf{^{11}B}$ **NMR** (96 MHz, $CDCl_3$) δ_B ppm 33.1 (br. s).

$\mathbf{\nu_{max}}$ (neat) = 2962, 1464, 1334, 1305, 1135, 965, 849, 699 cm^{-1}.

m/z (%) (ESI^+) 325 ($[M+Na]^+$, 100), 320 ($[M+NH_4]^+$, 12), 303 ($[M+H]^+$, 13).

HRMS (ESI^+) calcd. for $C_{19}H_{31}O_2{}^{11}BNa$ $[M+Na]^+$ 325.2309, found 325.2319.

2-(3-ethyl-2-phenylpentan-2-yl)-5,5-dimethyl-1,3,2-dioxaborinane (23)

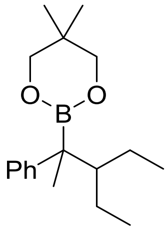

According to GP4A, 1-phenylethyl diisopropylcarbamate **5** (125 mg, 0.5 mmol, 1 equiv), *s*BuLi (0.5 mL, 0.65 mmol, 1.3 equiv) and 5,5-dimethyl-2-(pentan-3-yl)-1,3,2-dioxaborinane (138 mg, 0.75 mmol, 1.5 equiv) in anhydrous diethyl ether (3.25 mL) afforded after purification by flash chromatography (SiO_2, pentane/Et_2O 9:1) tertiary boronic ester **23** (118 mg, 0.41 mmol, 82%) as a colourless oil.

R_f (pentane/Et_2O 9:1) 0.50.

^{1}H NMR (400 MHz, $CDCl_3$) δ_H ppm 7.39 (d, J = 7.3, 2H, H_{Ar}), 7.24 (t, J = 7.7, 2H, H_{Ar}), 7.09 (t, J = 7.3, 1H, H_{Ar}), 3.55 (d, J = 11.4, 2H, 2×OC*H*H), 3.52 (d, J = 11.4, 2H, 2×OCH*H*), 1.93 (tt, J = 7.7, 3.7, 1H, CH), 1.45 – 1.30 (m, 2H, 2×C*H*HCH$_3$), 1.18 (s, 3H), 1.07 (m, 1H, CH*H*CH$_3$), 1.01 (t, J = 7.5, 3H, CH$_2$C*H*$_3$), 0.92 (m, 1H, CH*H*CH$_3$), 0.79 (s, 6H, C(CH$_3$)$_2$), 0.70 (t, J = 7.5, 3H, CH$_2$CH$_3$).

^{13}C NMR (101 MHz, $CDCl_3$) δ_C ppm 147.5 (C), 127.7 (CH), 127.3 (CH), 124.5 (CH), 72.0 (CH_2), 47.5 (CH), 31.5 (C), 28.1 (CH_2), 23.4 (CH_2), 21.8 (CH_3), 14.5 (CH_3), 14.4 (CH_3), 14.2 (CH_3).

^{11}B NMR (96 MHz, $CDCl_3$) δ_B ppm 29.9 (br. s).

ν_{max} (neat) = 2959, 2930, 2874, 1598, 1476, 1270, 1245, 1140, 699 cm^{-1}.

HRMS (ESI) calcd. for $C_{18}H_{29}BO_2Na$ [M+Na]$^+$ 311.2156, found 311.2154.

2-(4-ethyl-3-phenylhexan-3-yl)-5,5-dimethyl-1,3,2-dioxaborinane 24

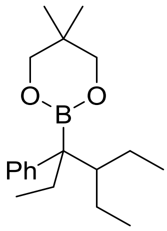

According to GP4A, 1-phenylpropyl diisopropylcarbamate **6** (125 mg, 0.5 mmol, 1 equiv), *s*BuLi (0.5 mL, 0.65 mmol, 1.3 equiv) and 5,5-dimethyl-2-(pentan-3-yl)-1,3,2-dioxaborinane (138 mg, 0.75 mmol, 1.5 equiv) in anhydrous diethyl ether (3.25 mL) afforded after purification by flash chromatography (SiO_2, pentane/Et_2O 9:1) tertiary boronic ester **24** (125 mg, 0.42 mmol, 83%) as a colourless oil.

R_f (pentane/Et_2O 9:1) 0.50.

^{1}H NMR (400 MHz, $CDCl_3$) δ_H ppm 7.31 – 7.20 (m, 4H, H_{Ar}), 7.15 – 7.06 (m, 1H, H_{Ar}), 3.61 (s, 4H, 2×OCH$_2$), 1.89 (dq, J = 14.9, 7.6, 1H, C*H*HCH$_3$), 1.81 (dq, J = 14.9, 7.6, 1H, CH*H*CH$_3$), 1.65 (app. td, J = 7.9, 3.9, 1H, C*H*), 1.55 (m, 1H, C*H*HCH$_3$), 1.44 (dtd, J = 14.9, 7.5, 2.9, 1H, CH*H*CH$_3$), 1.13 (dq, J = 14.6, 7.4, 1H, C*H*HCH$_3$), 1.01 (dq, J = 14.6, 7.4, 1H, CH*H*CH$_3$), 0.96 (s, 6H, C(CH$_3$)$_2$), 0.91 (t, J = 7.5, 3H, CH$_2$C*H*$_3$), 0.86 (t, J = 7.5, 3H, CH$_2$C*H*$_3$), 0.74 (t, J = 7.4, 3H, CH$_2$C*H*$_3$).

^{13}C NMR (101 MHz, $CDCl_3$) δ_C ppm 145.4 (C), 129.2 (CH), 127.3 (CH), 124.7 (CH), 71.9 (CH_2), 48.3 (CH), 31.3 (C), 27.6 (CH_2), 26.6 (CH_2), 25.7 (CH_2), 22.2 (CH_3), 14.8 (CH_3), 14.5 (CH_3), 10.8 (CH_3).

^{11}B NMR (96 MHz, $CDCl_3$) δ_B ppm 29.9 (br. s).

ν_{max} (neat) = 2959, 2931, 2874, 1599, 1475, 1410, 1242, 1147, 699 cm^{-1}.

HRMS (ESI) calcd. for $C_{19}H_{31}BO_2Na$ $[M+Na]^+$ 325.2313, found 325.2309.

(*S*)-2-(3-ethyl-2-phenylpentan-2-yl)-5,5-dimethyl-1,3,2-dioxaborinane (*S*)-24

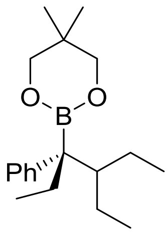

According to GP4A, (*S*)-1-phenylpropyl diisopropylcarbamate (*S*)-**6** (125 mg, 0.5 mmol, 1 eq.), *s*BuLi (0.5 mL, 0.65 mmol, 1.3 eq.) and 5,5-dimethyl-2-(pentan-3-yl)-1,3,2-dioxaborinane (138 mg, 0.75 mmol, 1.5 eq.) in anhydrous diethyl ether (3.25 mL) afforded after purification by flash chromatography (SiO_2, pentane/Et_2O 9:1) tertiary boronic ester (*S*)-**24** (121 mg, 0.40 mmol, 80%) as a colourless oil.

$[\alpha]_D^{22}$ +2 (*c* 1, $CHCl_3$).

HPLC separation conditions: Chiralpak IA column with guard, 2.0% *i*PrOH in hexane, flow rate 1 mL/min, 20 °C; t_R 6.6 min for (*S*)-enantiomer (major) and 7.3 min for (*R*)-enantiomer (minor).

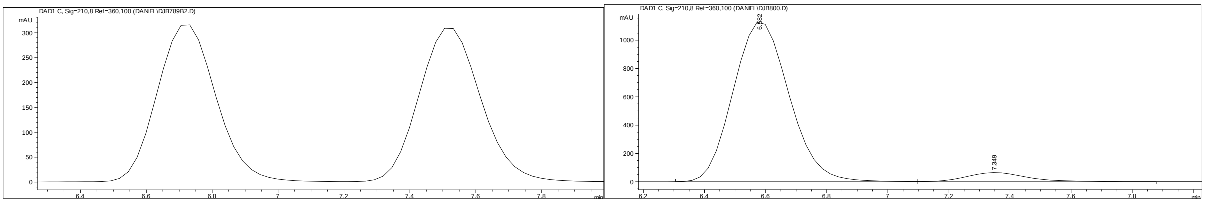

(*R*)-2-(3-ethyl-2-phenylpentan-2-yl)-5,5-dimethyl-1,3,2-dioxaborinane (*R*)-24

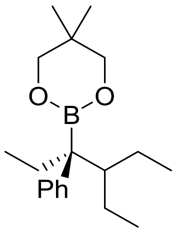

According to GP4A, (*R*)-1-phenylpropyl diisopropylcarbamate (*R*)-**6** (125 mg, 0.5 mmol, 1 eq.), *s*BuLi (0.5 mL, 0.65 mmol, 1.3 eq.) and 5,5-dimethyl-2-(pentan-3-yl)-1,3,2-dioxaborinane (138 mg, 0.75 mmol, 1.5 eq.) in anhydrous diethyl ether (3.25 mL) afforded after purification by flash chromatography (SiO_2, pentane/Et_2O 9:1) tertiary boronic ester (*R*)-**24** (120 mg, 0.40 mmol, 79%) as a colourless oil.

$[\alpha]_D^{21}$ −2 (*c* 1, $CHCl_3$).

HPLC separation conditions: Chiralpak IA column with guard, 2.0% *i*PrOH in hexane, flow rate 1 mL/min, 20 °C; t_R 6.6 min for (*S*)-enantiomer (major) and 7.3 min for (*R*)-enantiomer (minor).

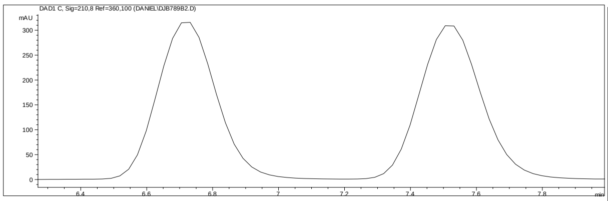

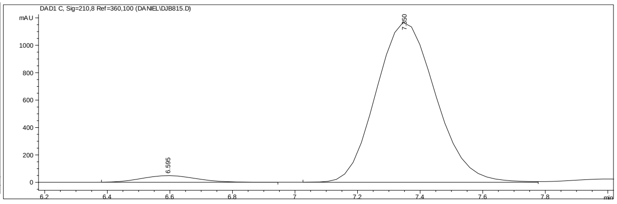

2.6 Synthesis of bifluranol

2-((2*S*,3*R*)-3-(3-Fluoro-4-methoxyphenyl)-2-(4-methoxyphenyl)pentan-2-yl)-4,4,5,5-tetramethyl-1,3,2-dioxaborolane (33)

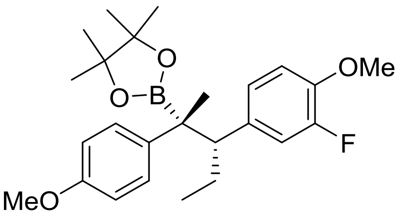

A solution of (*S*)-1-(4-methoxyphenyl)ethyl diisopropylcarbamate ((*S*)-**32**) (92 mg, 0.33 mmol, 1.0 equiv) and TMEDA (64 µL, 0.43 mmol, 1.3 equiv) in anhydrous diethyl ether (2.0 mL) was cooled to −78 °C. *s*BuLi (330 µL, 0.43 mmol, 1.3 equiv) was added dropwise and the reaction mixture was stirred at this temperature for 1 h. A solution of (*S*)-2-(1-(3-fluoro-4-methoxyphenyl)propyl)-4,4,5,5-tetramethyl-1,3,2-dioxaborolane ((*S*)-**30**) (145 mg, 0.49 mmol, 1.5 equiv) in anhydrous diethyl ether (1.0 mL) was added dropwise and the mixture was stirred for 2 h at −78 °C. The cooling bath was removed and stirring was continued at room temperature for 14 h. The reaction mixture was cooled to 0 °C and 1.0 M aqueous KH_2PO_4 (2.0 mL) was added slowly. After stirring for 10 min at room temperature, the phases were separated, and the aqueous phase was extracted with diethyl ether (4 × 10 mL). The combined organic phases were dried over anhydrous $MgSO_4$, filtered, and the solvent was removed *in vacuo*. The crude product was purified by column chromatography (SiO_2, pentane/EtOAc 30:1) to afford tertiary boronic ester **33** (134 mg, 0.31 mmol, 95%) as a white solid. The ratio of diastereomers was measured by ^{1}H NMR and accounted to >20:1 (*anti*:*syn*).

mp 108–109 °C ($CHCl_3$).

$\mathbf{R_f}$ (pentane/EtOAc 30:1) 0.07.

^{1}H NMR (500 MHz, $CDCl_3$) δ_H ppm 7.10 (AA’BB’, *J* = 8.9 Hz, 2 H, H_{Ar}), 6.73 (AA’BB’, *J* = 8.9 Hz 2 H, H_{Ar}), 6.67–6.60 (m, 2 H, H_{Ar}), 6.45 (d, *J* = 8.2 Hz, 1 H, H_{Ar}), 3.80 (s, 3 H, OCH_3), 3.76 (s, 3 H, OCH_3), 2.98 (dd, *J* = 11.7, 2.6 Hz, 1 H, CH), 1.82 (m, 1 H, C*H*H), 1.71

(m, 1 H, CH*H*), 1.27 (s, 6 H, 2×CH_3), 1.24 (s, 3 H, CH_3), 1.22 (s, 6 H, 2×CH_3), 0.74 (t, J = 7.2 Hz, 3 H, CH_3).

^{13}C NMR (126 MHz, $CDCl_3$) δ_C ppm 157.2 (*C*OMe), 151.5 (d, ^1J = 244.4 Hz, CF), 145.2 (d, ^2J = 10.8 Hz, *C*OMe), 137.1 (C), 134.5 (d, ^3J = 5.7 Hz, C), 128.4 (CH), 125.5 (d, ^3J = 3.8 Hz, CH), 116.7 (d, ^2J = 17.2 Hz, CH), 113.0 (CH), 111.8 (CH), 83.4 (O*C*(CH_3)$_2$), 56.1 (OCH_3), 55.1 (OCH_3), 55.0 (CH), 25.3 (CH_2), 24.72 (CH_3), 24.67 (CH_3), 16.3 (CH_3), 13.1 (CH_3).

^{11}B NMR (96 MHz, $CDCl_3$) δ_B ppm 33.0 (br. s).

^{19}F NMR (470 MHz, $CDCl_3$) δ_F ppm –137.4 (dd, J = 12.7, 8.5 Hz, CF).

ν_{max} (neat) = 2963, 1512, 1458, 1308, 1266, 1130, 1086, 1029, 854, 740 cm^{-1}.

m/z (%) (ESI^+) 451 ($[M+Na]^+$, 100), 321 ($[M–ArOMe]^+$, 16), 303 ($[M–ArFOMe]^+$, 13).

HRMS (ESI^+) calcd. for $C_{25}H_{34}O_4{}^{11}BFNa$ $[M+Na]^+$ 451.2431, found 451.2427.

$[\alpha]_D^{21}$ –132 (*c* 0.29, $CHCl_3$).

2-Fluoro-1-methoxy-4-((2*S*,3*R*)-2-(4-methoxyphenyl)pentan-3-yl)benzene (34)

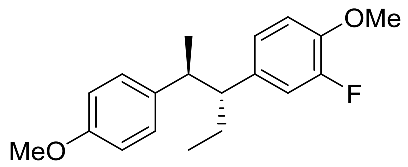

A solution of tertiary boronic ester **33** (120 mg, 0.28 mmol, 1.0 equiv) and TBAF·3H_2O (265 mg, 0.84 mmol, 3.0 equiv) in anhydrous toluene (3.0 mL) was heated under reflux for 3 h. After cooling to ambient temperature, H_2O (15 mL) and diethyl ether (15 mL) were added and the organic phase was washed with H_2O (3 × 15 mL), dried over anhydrous $MgSO_4$, filtered, and the solvent was removed under reduced pressure. The residue was purified by column chromatography (SiO_2, pentane/EtOAc 30:1) to give protodeboronated product **34** (84 mg, 0.28 mmol, 99%) as a white solid. The product was obtained as a mixture of diastereomers (*anti*:*syn* >20:1).

mp 80–81 °C (pentane/EtOAc).

R_f (pentane/EtOAc 30:1) 0.20.

^{1}H NMR (400 MHz, $CDCl_3$) δ_H ppm 7.11 (AA'BB', J = 8.6 Hz, 2 H, H_{Ar}), 6.94–6.83 (m, 5 H, H_{Ar}), 3.90 (s, 3 H, OCH_3), 3.82 (s, 3 H, OCH_3), 2.74 (dq, J = 10.1, 7.1 Hz, 1 H, CH), 2.42 (td, J = 10.1, 3.4 Hz, 1 H, CH), 1.46 (m, 1 H, C*H*H), 1.30 (m, 1 H, CH*H*), 0.97 (d, J = 7.1 Hz, 3 H, CH_3), 0.58 (t, J = 7.3 Hz, 3 H, CH_3).

^{13}C NMR (126 MHz, $CDCl_3$) δ_C ppm 157.9 (*C*OMe), 152.4 (d, ^1J = 245.1 Hz, CF), 145.7 (d, ^2J = 10.5 Hz, *C*OMe), 138.6 (C), 137.5 (d, ^3J = 5.7 Hz, C), 128.3 (CH), 124.1 (d, ^3J = 2.9 Hz,

CH), 115.4 (d, ^{2}J = 18.1 Hz, CH), 113.7 (CH), 113.0 (CH), 56.3 (OCH_3), 55.2 (OCH_3), 54.5 (CH), 45.4 (CH), 27.2 (CH_2), 20.9 (CH_3), 12.2 (CH_3).

^{19}F NMR (470 MHz, $CDCl_3$) δ_F ppm –135.9 (dd, *J* = 12.7, 8.5 Hz, CF).

ν_{max} (neat) = 2961, 1614, 1511, 1457, 1308, 1252, 1029, 854, 832 cm^{-1}.

m/z (%) (EI^+) 302 ($[M]^+$, 39), 273 ($[M–Et]^+$, 5), 243 (14), 167 ($[ArFOMeC_3H_6]^+$, 18), 139 (15), 135 ($[ArOMeC_2H_4]^+$, 100).

HRMS (EI^+) calcd. for $C_{19}H_{23}O_2F$ $[M]^+$ 302.1682, found 302.1674.

$[\alpha]_D^{22}$ –14.2 (*c* 0.32, $CHCl_3$).

2-Bromo-4-((2*S*,3*R*)-3-(3-fluoro-4-methoxyphenyl)pentan-2-yl)-1-methoxybenzene (35)

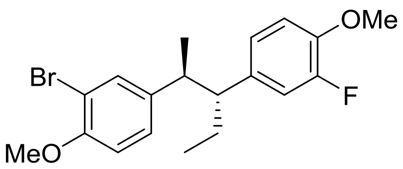

A mixture of 2-fluoro-1-methoxy-4-((2*S*,3*R*)-2-(4-methoxyphenyl)pentan-3-yl)benzene (**34**) (82 mg, 0.27 mmol, 1.0 equiv) and NBS (53 mg, 0.30 mmol, 1.1 equiv) in anhydrous MeCN (2.0 mL) was stirred at room temperature for 21 h. The mixture was concentrated under reduced pressure, H_2O (5.0 mL) was added and the mixture was extracted with EtOAc (4 × 10 mL). The combined organic phases were washed with brine, dried over anhydrous $MgSO_4$, filtered, and the solvent was removed *in vacuo*. After purification by column chromatography (SiO_2, pentane/EtOAc 30:1) 2-bromo-4-((2*S*,3*R*)-3-(3-fluoro-4-methoxyphenyl)pentan-2-yl)-1-me-thoxybenzene (**35**) (97 mg, 0.25 mmol, 94%) was obtained as a white solid as a mixture of diastereomers (*anti*:*syn* >20:1).

mp 85.5–86.5 °C ($CHCl_3$).

R_f (pentane/EtOAc 30:1) 0.10.

^{1}H NMR (500 MHz, $CDCl_3$) δ_H ppm 7.37 (d, *J* = 2.1 Hz, 1 H, H_{Ar}), 7.07 (dd, *J* = 8.2, 2.1 Hz, 1 H, H_{Ar}), 6.93–6.81 (m, 4 H, H_{Ar}), 3.90 (s, 6 H, 2×OCH_3), 2.71 (dq, *J* = 10.2, 7.0 Hz, 1 H, CH), 2.40 (dt, *J* = 10.2, 3.5 Hz, 1 H, CH), 1.45 (m, 1 H, C*H*H), 1.30 (m, 1 H, CH*H*), 0.96 (d, *J* = 7.0 Hz, 3 H, CH_3), 0.59 (t, *J* = 7.3 Hz, 3 H, CH_3).

^{13}C NMR (126 MHz, $CDCl_3$) δ_C ppm 154.1 (*C*OMe), 152.4 (d, ^{1}J = 244.2 Hz, CF), 145.8 (d, ^{2}J = 10.5 Hz, *C*OMe), 140.2 (C), 136.9 (d, ^{3}J = 4.8 Hz, C), 132.1 (CH), 127.5 (CH), 124.2 (d, ^{3}J = 3.8 Hz, CH), 115.3 (d, ^{2}J = 18.1 Hz, CH), 113.1 (CH), 111.8 (CH), 111.5 (CBr), 56.3 (OCH_3), 56.2 (OCH_3), 54.4 (CH), 45.2 (CH), 27.1 (CH_2), 20.8 (CH_3), 12.2 (CH_3).

^{19}F NMR (470 MHz, $CDCl_3$) δ_F ppm –135.7 (dd, J = 12.7, 8.5 Hz, CF).

ν_{max} (neat) = 2959, 1519, 1491, 1452, 1277, 1256, 1131, 1053, 872, 806, 760 cm^{-1}.

m/z (%) (ESI^+) 403 ($[M+Na]^+$, 100), 255 ($[M–ArFOMe]^+$, 6), 195 ($[M–ArBrOMe]^+$, 12).

HRMS (ESI^+) calcd. for $C_{19}H_{22}O_2{}^{79}BrFNa$ $[M+Na]^+$ 403.0679, found 403.0680.

$[\alpha]_D^{22}$ –14.0 (c 0.72, $CHCl_3$).

4,4'-((2*S*,3*R*)-Pentane-2,3-diyl)bis(2-fluorophenol), Bifluranol (1)

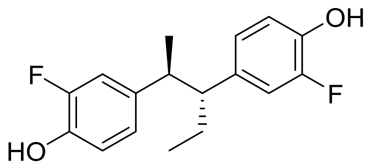

A solution of 2-bromo-4-((2*S*,3*R*)-3-(3-fluoro-4-methoxyphenyl)pentan-2-yl)-1-methoxybenzene (**35**) (42 mg, 0.11 mmol, 1.0 equiv) in anhydrous THF (2.0 mL) was cooled to −78 °C. *n*-Butyllithium (89 µL, 1.60 M solution in hexanes, 0.14 mmol, 1.3 equiv) was added dropwise and the mixture was stirred at this temperature for 30 min. A solution of NFSI (42 mg, 0.13 mmol, 1.2 equiv) in anhydrous THF (0.5 mL) was added dropwise and the mixture was stirred for 2 h at −78 °C. The cooling bath was removed and the mixture was allowed to warm to room temperature. H_2O (5.0 mL) was added slowly and the solution was extracted with EtOAc (4 × 10 mL). The combined organic layers were washed with H_2O, dried over anhydrous Na_2SO_4, filtered, and the solvent was removed *in vacuo*. The crude product was dissolves in anhydrous CH_2Cl_2 (1.5 mL) and cooled to –20 °C. According to a procedure of *Katzenellenbogen* and co-workers,[21] a solution of BBr_3 (330 µL, 1.0 M in CH_2Cl_2, 0.33 mmol, 3.0 equiv) was added dropwise. The reaction mixture was stirred for 30 min at –20 °C and then allowed to warm to 4 °C and stirred for 16 h. Afterwards, the mixture was cooled to –78 °C and anhydrous MeOH (0.5 mL) was added dropwise followed by conc. aq. NH_3 solution (0.5 mL). The mixture was allowed to warm to room temperature, the solvent was removed *in vacuo*, and the residue was portioned between H_2O (10 mL) and EtOAc (10 mL). The aqueous layer was extracted with EtOAc (3 × 10 mL) and the combined organic phases were washed with brine (20 mL), dried over anhydrous $MgSO_4$, filtered, and the solvent was removed under reduced pressure. The residue was purified by column chromatography (SiO_2, pentane/EtOAc 9:1 → 4:1) to afford bifluranol (**1**) (14 mg, 48 µmol, 43%) as a white solid as single diastereomer.

mp 155–157 °C (acetone).

R_f (pentane/EtOAc 4:1) 0.25.

^{1}H NMR (500 MHz, acetone-d6) δ_H ppm 8.38 (s, 2 H, OH), 7.03–6.86 (m, 6 H, H_{Ar}), 2.79 (dq, *J* = 10.3, 7.0 Hz, 1 H, CH), 2.49 (dt, *J* = 10.3, 3.7 Hz, 1 H, CH), 1.42 (m, 1 H, C*H*H), 1.34 (m, 1 H, CH*H*), 0.94 (d, *J* = 7.0 Hz, 3 H, CH_3), 0.56 (t, *J* = 7.3 Hz, 3 H, CH_3).

^{13}C NMR (126 MHz, acetone-d6) δ_C ppm 152.4 (d, ^{1}J = 239.4 Hz, CF), 152.3 (d, ^{1}J = 239.4 Hz, CF), 143.9 (d, ^{2}J = 10.5 Hz, COH), 143.8 (d, ^{2}J = 10.5 Hz, COH), 140.0 (d, ^{3}J = 4.8 Hz, C), 137.2 (d, ^{3}J = 5.7 Hz, C), 125.4 (d, ^{4}J = 3.8 Hz, CH), 124.5 (d, ^{4}J = 2.9 Hz, CH), 118.5 (d, ^{3}J = 2.9 Hz, CH), 118.4 (d, ^{3}J = 2.9 Hz, CH), 116.2 (d, ^{2}J = 18.1 Hz, CH), 115.5 (d, ^{2}J = 17.2 Hz, CH), 55.0 (CH), 46.1 (CH), 28.0 (CH_2), 21.5 (CH_3), 12.6 (CH_3).

^{19}F NMR (283 MHz, acetone-d6) δ_F ppm –138.6 (dd, *J* = 13.0, 8.1 Hz, CF), –138.8 (dd, *J* = 12.2, 8.9 Hz, CF).

ν_{max} (neat) = 3307, 2961, 1603, 1515, 1439, 1273, 1231, 1107, 866, 817, 780 cm^{-1}.

m/z (%) (EI^+) 292 ($[M]^+$, 36), 201 (18), 199 (24), 153 ($[ArC_3H_6]^+$, 100), 139 ($[ArC_2H_4]^+$, 92), 125 ($[ArCH_2]^+$, 67).

HRMS (EI^+) calcd. for $C_{17}H_{18}O_2F_2$ $[M]^+$ 292.1275, found 292.1270.

$[\alpha]_D^{22}$ –4.6 (*c* 0.44, acetone).

2.7 Synthesis of fluorohexestrol

2-((3R,4S)-4-(3-fluoro-4-methoxyphenyl)-3-(4-methoxyphenyl)hexan-3-yl)-5,5-dimethyl-1,3,2-dioxaborinane (3*R*,4*S*)-41

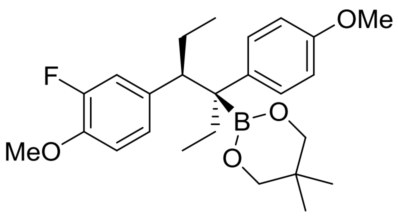

A solution of (*S*)-1-(4-methoxyphenyl)propyl diisopropylcarbamate ((*R*)-**39**) (293 mg, 1.00 mmol, 1.0 equiv) and TMEDA (0.204 mL, 1.30 mmol, 1.3 equiv) in anhydrous diethyl ether (5.0 mL) was cooled to –78 °C. *s*BuLi (1.00 mL, 1.30 mmol, 1.3 equiv) was added dropwise and the reaction mixture was stirred at this temperature for 1 h. A solution of (*R*)-2-(1-(3-fluoro-4-methoxyphenyl)propyl)-5,5-dimethyl-1,3,2-dioxaborinane ((*R*)-**40**) (420 mg, 1.50 mmol, 1.5 equiv) in anhydrous diethyl ether (1.5 mL) was added dropwise and the mixture was stirred for 3 h at –78 °C. The cooling bath was removed and stirring was continued at room temperature for 14 h. The reaction mixture was quenched through the addition of water (20 mL), the phases were separated, and the aqueous phase was extracted with diethyl ether (3 × 20 mL). The combined organic phases were dried over anhydrous $MgSO_4$, filtered, and the solvent was removed *in vacuo*. The crude product was purified by column chromatography (SiO_2, pentane/Et_2O 85:15) to afford tertiary boronic ester **41** (301 mg,

0.70 mmol, 70%) as a colourless oil. The ratio of diastereomers was measured by ^{1}H NMR and accounted to 20:1 (*anti*:*syn*).

R$_f$ (pentane/EtOAc 9:1) 0.27.

^{1}H NMR (400 MHz, $CDCl_3$) 7.13 – 7.07 (m, 2H), 6.84 – 6.63 (m, 5H), 3.88 (s, 3H, OCH_3), 3.81 (s, 3H, OCH_3), 3.64 (s, 4H, OCH_2), 2.84 (dd, J=12.1, 2.8, 1H, CH), 1.79 – 1.64 (m, 3H, C*H*$_2$CH$_3$), 1.47 (m, 1H, C*H*$_2$CH$_3$), 0.99 (s, 6H), 0.67 (t, J=7.3, 3H, CH$_2$C*H*$_3$), 0.59 (t, J=7.3, 3H, CH$_2$C*H*$_3$).

^{13}C NMR (126 MHz, $CDCl_3$) 157.1 (*C*OMe), 151.7 (d, J=243.0, CF), 145.4 (d, J=10.8, *C*OMe), 136.8 (d, J=5.4, C), 130.6 (CH), 125.7 (d, J=3.3, CH), 117.0 (d, J=17.9, CH), 112.7 (CH), 112.0 (d, J=2.0, CH), 71.8 (CH_2), 56.2 (OCH_3), 55.4 (OCH_3), 55.1 (CH), 31.2 (C), 29.6 (CH_2), 23.4 (CH_2), 22.2 (CH_3), 12.9 (CH_3), 11.0 (CH_3).

^{11}B NMR (96 MHz, $CDCl_3$) δ_B ppm 29.6 (br. s).

^{19}F NMR (283 MHz, $CDCl_3$) δ_F ppm -136.94 (dd, *J*=13.5, 9.2)

ν_{max} (neat) = 2961, 2933, 2874, 2837, 1757, 1608, 1579, 1511, 1247, 1182, 1129, 1034 cm^{-1}.

$[\alpha]_D^{20}$ −7 (*c* 1, $CHCl_3$).

2-Fluoro-1-methoxy-4-((3*S*,4*R*)-4-(4-methoxyphenyl)hexan-3-yl)benzene (42)

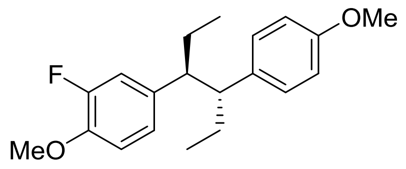

A solution of tertiary boronic ester **41** (150 mg, 0.35 mmol, 1.0 equiv) and TBAF·3H_2O (332 mg, 1.05 mmol, 3.0 equiv) in anhydrous toluene (4.0 mL) was heated under reflux for 3 h. After cooling to ambient temperature, H_2O (20 mL) and diethyl ether (20 mL) were added and the organic phase was washed with H_2O (3 × 15 mL), dried over anhydrous $MgSO_4$, filtered, and the solvent was removed under reduced pressure. The residue was purified by column chromatography (SiO_2, pentane/EtOAc 20:1) to give protodeboronated product **42** (90 mg, 0.28 mmol, 81%) as a white solid. The product was obtained as a mixture of diastereomers (*anti*:*syn* 93:7).

mp 126–127 °C (pentane/EtOAc).

R$_f$ (pentane/EtOAc 30:1) 0.21.

Analytical data of the major *anti* diastereomer.

^{1}H NMR (400 MHz, $CDCl_3$) δ_H ppm 7.07 (AA'BB', *J* = 8.6 Hz, 2 H, H$_{Ar}$), 6.94–6.85 (m, 5 H, H$_{Ar}$), 3.90 (s, 3 H, OCH_3), 3.82 (s, 3 H, OCH_3), 2.52–2.41 (m, 2 H, CH), 1.46–1.35 (m,

2 H, 2×C*H*H), 1.33–1.18 (m, 2 H, 2×CH*H*), 0.552 (t, *J* = 7.3 Hz, 3 H, CH_3), 0.546 (t, *J* = 7.3 Hz, 3 H, CH_3).

^{13}C NMR (126 MHz, $CDCl_3$) δ_C ppm 157.9 (*C*OMe), 152.4 (d, ^1J = 245.1 Hz, CF), 145.6 (d, ^2J = 10.5 Hz, *C*OMe), 137.9 (d, ^3J = 4.8 Hz, C), 136.1 (C), 129.1 (CH), 124.1 (d, ^3J = 3.8 Hz, CH), 115.3 (d, ^2J = 18.1 Hz, CH), 113.6 (CH), 113.1 (CH), 56.3 (OCH_3), 55.2 (OCH_3), 53.6 (CH), 53.4 (CH), 27.31 (CH_2), 27.26 (CH_2), 12.2 (CH_3), 12.1 (CH_3).

^{19}F NMR (470 MHz, $CDCl_3$) δ_F ppm –135.8 (dd, *J* = 12.1, 8.9 Hz, CF).

ν_{max} (neat) = 2957, 1610, 1511, 1440, 1250, 1130, 1025, 831, 759 cm^{-1}.

m/z (%) (ESI^+) 339 ($[M+Na]^+$, 100).

HRMS (ESI^+) calcd. for $C_{20}H_{25}O_2FNa$ $[M+Na]^+$ 339.1731, found 339.1743.

$[\alpha]_D^{22}$ ±0.0 (*c* 0.51, $CHCl_3$).

The analytical data match those reported in literature for the racemic compound.[21,22]

2-Fluoro-4-((2*S*,3*R*)-3-(4-hydroxyphenyl)pentan-2-yl)phenol, Fluorohexestrol (2)

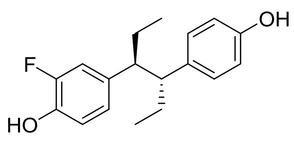

According to a procedure of *Katzenellenbogen* and co-workers,[21] a solution of BBr_3 (1.28 mL, 1.0 M in CH_2Cl_2, 1.28 mmol, 3.0 equiv) was added dropwise to a solution of diaryl **42** (135 mg, 0.43 mmol, 1.0 equiv) in anhydrous CH_2Cl_2 (4.3 mL) at –20 °C. The reaction mixture was stirred for 30 min at –20 °C and then allowed to warm to 4 °C and stirred for 15 h. Afterwards, the mixture was cooled to –78 °C and anhydrous MeOH (0.5 mL) was added dropwise followed by conc. aq. NH_3 solution (0.5 mL). The mixture was allowed to warm to room temperature, the solvent was removed *in vacuo,* and the residue was portioned between H_2O (10 mL) and EtOAc (10 mL). The aqueous layer was extracted with EtOAc (3 × 10 mL) and the combined organic phases were washed with brine (20 mL), dried over anhydrous $MgSO_4$, filtered, and the solvent was removed under reduced pressure to give 2-fluoro-4-(4-(4-hydroxyphenyl)hexan-3-yl)phenol (121 mg, 0.42 mmol, 99%) as off-white solid as a mixture of diastereomers. To separate the isomers, the residue was purified by flash chromatography (SiO_2, pentane/EtOAc 9:1 → 1:1) to afford 2-fluoro-4-((2*S*,3*R*)-3-(4-hydroxyphenyl)pentan-2-yl)phenol (**2**) (89 mg, 0.31 mmol, 72%) as white crystalline solid and 2-fluoro-4-((3*S*,4*S*)-4-(4-hydroxyphenyl)hexan-3-yl)phenol (**47**) (20 mg, 69 µmol, 16%) as a colourless oil, which crystallised upon standing (*vide infra*).

Analytical data for 2-fluoro-4-((2*S*,3*R*)-3-(4-hydroxyphenyl)pentan-2-yl)phenol (**2**)

mp 197–201 °C (pentane/EtOAc). Lit. 200.5–201.5 °C (THF/cyclohexane).[21]

R_f (pentane/EtOAc 4:1) 0.22.

^{1}H NMR (500 MHz, acetone-d6) δ_H ppm 8.35 (s, 1 H, OH), 8.07 (s, 1 H, OH), 7.05 (AA'BB', *J* = 8.6 Hz, 2 H, H_{Ar}), 6.98 (dd, *J* = 12.5, 1.8 Hz, 1 H, H_{Ar}), 6.95 (dd, *J* = 8.9, 8.2 Hz, 1 H, H_{Ar}), 6.88 (dd, *J* = 8.2, 1.8 Hz, 1 H, H_{Ar}), 6.80 (AA'BB', *J* = 8.6 Hz, 2 H, H_{Ar}), 2.56–2.47 (m, 2 H, 2×CH), 1.43–1.35 (m, 2 H, 2×C*H*H), 1.33–1.24 (m, 2 H, 2×CH*H*), 0.52 (t, *J* = 7.4 Hz, 6 H, 2×CH_3).

^{13}C NMR (101 MHz, acetone-d6) δ_C ppm 156.6 (COH), 152.4 (d, ^1J = 239.4 Hz, CF), 143.7 (d, ^2J = 13.0 Hz, COH), 137.9 (d, ^3J = 4.8 Hz, C), 135.9 (C), 130.0 (CH), 125.3 (d, ^3J = 2.7 Hz, CH), 118.3 (d, ^4J = 2.3 Hz, CH), 116.1 (d, ^2J = 17.7 Hz, CH), 116.0 (CH), 54.3 (CH), 54.2 (CH), 28.2 (CH_2), 28.1 (CH_2), 12.59 (CH_3), 12.55 (CH_2).

^{19}F NMR (470 MHz, acetone-d6) δ_F ppm –138.8 (br. m).

ν_{max} (neat) = 3298, 2958, 1600, 1512, 1437, 1222, 1106, 830, 804, 775 cm^{-1}.

m/z (%) (CI^+) 288 ($[M]^+$, 8), 287 ($[M–H]^+$, 26), 269 ($[M–F]^+$, 35), 259 ($[M–Et]^+$, 30), 223 (16), 195 ($[M–Ar]^+$, 99), 177 ($[M–ArF]^+$, 48), 153 ($[ArFC_3H_7]^+$, 46), 135 ($[ArC_3H_7]^+$, 100), 125 ($[ArFCH_2]^+$, 62), 107 ($[ArCH_2]^+$, 35), 93 ($[PhOH]^+$, 7).

HRMS (EI^+) calcd. for $C_{18}H_{21}O_2F$ $[M]^+$ 288.1526, found 288.1535.

$[\alpha]_D^{21}$ +1.0 (*c* 1.0, $CHCl_3$).

The analytical data are consistent with those reported in literature for the racemic compound.[21]

HPLC separation conditions: Chiralpak ADH column with guard, 10.0% *i*PrOH in hexane, flow rate 0.7 mL/min, 20 °C; t_R 23.1 min for *anti*-diastereomer (major) and t_R 25.7 and 34.3 min for *syn*-diastereomers (minor).
d.r. = 100:0.0.

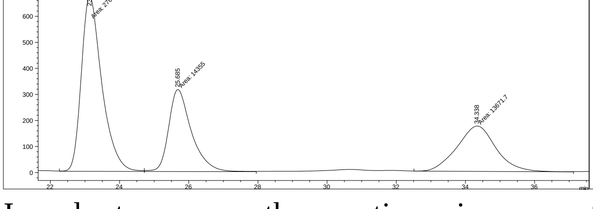

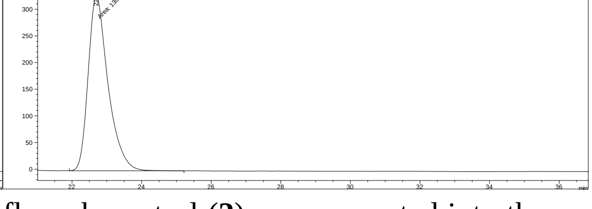

In order to measure the enantiomeric excess, fluorohexestrol (**2**) was converted into the mono protected benzyl ether (**48**).[23]

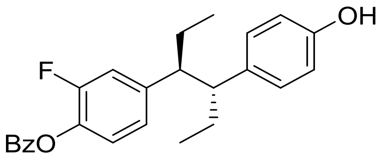

48

HPLC separation conditions: Chiralpak IA column with guard, 5.0% *i*PrOH in hexane, flow rate 0.7 mL/min, 20 °C; t_R 39.0 min for (*R*,*S*)-enantiomer (major) and t_R 43.6 min for (*S*,*R*)-enantiomer (minor).

e.r. = 99.8:1.2.

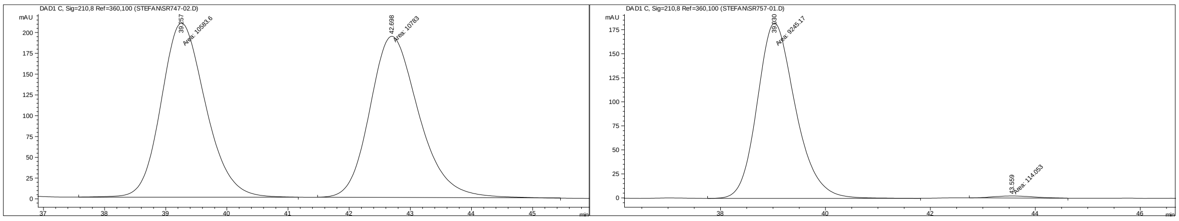

2-Fluoro-4-((3*S*,4*S*)-4-(4-hydroxyphenyl)hexan-3-yl)phenol (47)

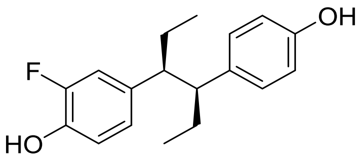

mp 106–107 °C (pentane/EtOAc).

R_f (pentane/EtOAc 4:1) 0.16.

^{1}H NMR (400 MHz, acetone-d6) δ_H ppm 8.20 (br. s, 1 H, OH), 7.96 (br. s, 1 H, OH), 6.76 (AA'BB', J = 8.8 Hz, 2 H, H_{Ar}), 6.71 (m, 1 H, H_{Ar}), 6.62 (AA'BB', J = 8.8 Hz, 2 H, H_{Ar}), 6.61–6.56 (m, 2 H, H_{Ar}), 2.70–2.56 (m, 2 H, 2×CH), 1.94–1.82 (m, 2 H, 2×C*H*H), 1.57–1.46 (m, 2 H, 2×CH*H*), 0.714 (t, J = 7.3 Hz, 3 H, CH_3), 0.707 (t, J = 7.3 Hz, 3 H, CH_3).

^{13}C NMR (126 MHz, acetone-d6) δ_C ppm 156.2 (COH), 151.8 (d, ^1J = 239.4 Hz, CF), 143.3 (d, ^2J = 13.4 Hz, COH), 136.7 (d, ^3J = 4.8 Hz, C), 134.6 (C), 130.7 (CH), 125.9 (d, ^3J = 2.9 Hz, CH), 117.6 (d, ^4J = 2.9 Hz, CH), 116.9 (d, ^2J = 18.1 Hz, CH), 115.3 (CH), 53.3 (CH), 53.2 (CH), 26.99 (CH_2), 26.98 (CH_2), 12.71 (CH_3), 12.68 (CH_2).

^{19}F NMR (470 MHz, acetone-d6) δ_F ppm –139.7 (dd, J = 12.7, 10.6 Hz, CF).

ν_{max} (neat) = 3317, 2961, 1598, 1511, 1439, 1365, 1226, 1111, 827, 777 cm^{-1}.

m/z (%) (CI$^+$) 288 ([M]$^+$, 7), 287 ([M–H]$^+$, 24), 269 ([M–F]$^+$, 45), 259 ([M–Et]$^+$, 25), 239 (32), 223 (12), 195 ([M–Ar]$^+$, 87), 177 ([M–ArF]$^+$, 51), 175 (37), 153 ([ArFC$_3$H$_7$]$^+$, 55), 135 ([ArC$_3$H$_7$]$^+$, 100), 125 ([ArFCH$_2$]$^+$, 58), 107 ([ArCH$_2$]$^+$, 42), 93 ([PhOH]$^+$, 8).

HRMS (EI^+) calcd. for $C_{18}H_{21}O_2F$ $[M]^+$ 288.1526, found 288.1534.

$[\alpha]_D^{21}$ −12.0 (*c* 0.67, $CHCl_3$, for 64% ee).

HPLC separation conditions: Chiralpak ADH column with guard, 10.0% *i*PrOH in hexane, flow rate 0.7 mL/min, 20 °C; t_R 25.7 min for (*R*,*R*)-enantiomer (minor) and 34.3 min for (*S*,*S*)-enantiomer (major).

e.r. = 81.9:18.1.

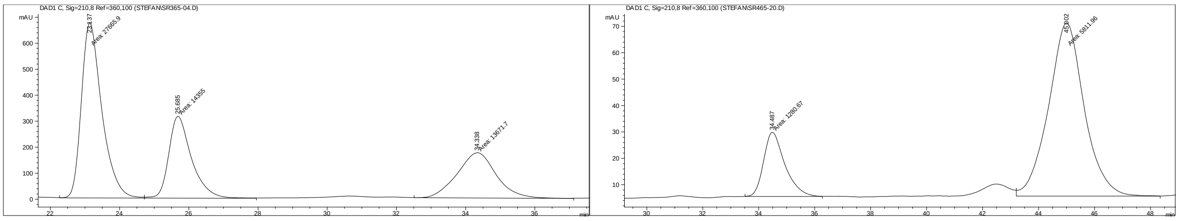

3. X-ray structure of Fluorohexestrol (2)

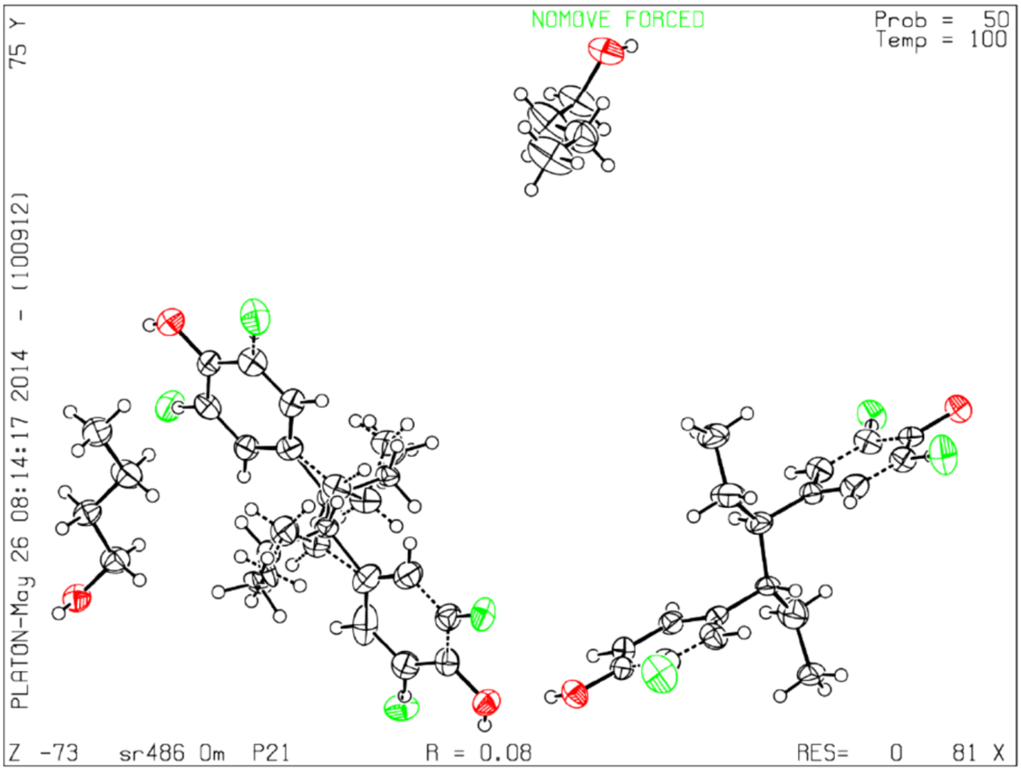

Bond precision: C-C = 0.0106 A Wavelength=1.54178

Cell:	a=5.8233(7)	b=16.811(3)	c=21.303(3)
	alpha=90	beta=97.041(7)	gamma=90
Temperature:	100 K		

	Calculated	Reported
Volume	2069.7(5)	2069.7(5)
Space group	P 21	P21
Hall group	P 2yb	?
Moiety formula	C18 H21 F O2, C4 H10 O	C18 H21 F O2, C4 H10 O
Sum formula	C22 H31 F O3	C22 H31 F O3
Mr	362.47	362.47
Dx,g cm-3	1.163	1.163
Z	4	4
Mu (mm-1)	0.659	0.659
F000	784.0	784.0
F000’	786.39	
h,k,lmax	6,19,25	6,19,25
Nref	7153[3710]	3609
Tmin,Tmax	0.969,0.974	0.574,0.753
Tmin’	0.865	

Correction method= MULTI-SCAN

Data completeness= 0.97/0.50 Theta(max)= 65.820

R(reflections)= 0.0780(2972) wR2(reflections)= 0.2150(3609)

S = 1.021 Npar= Npar = 590

4. ^{1}H NMR and ^{13}C NMR spectra

(*S*)-1-(4-Methoxyphenyl)ethanol ((*S*)-31)

^{1}H NMR (500 MHz, $CDCl_3$)

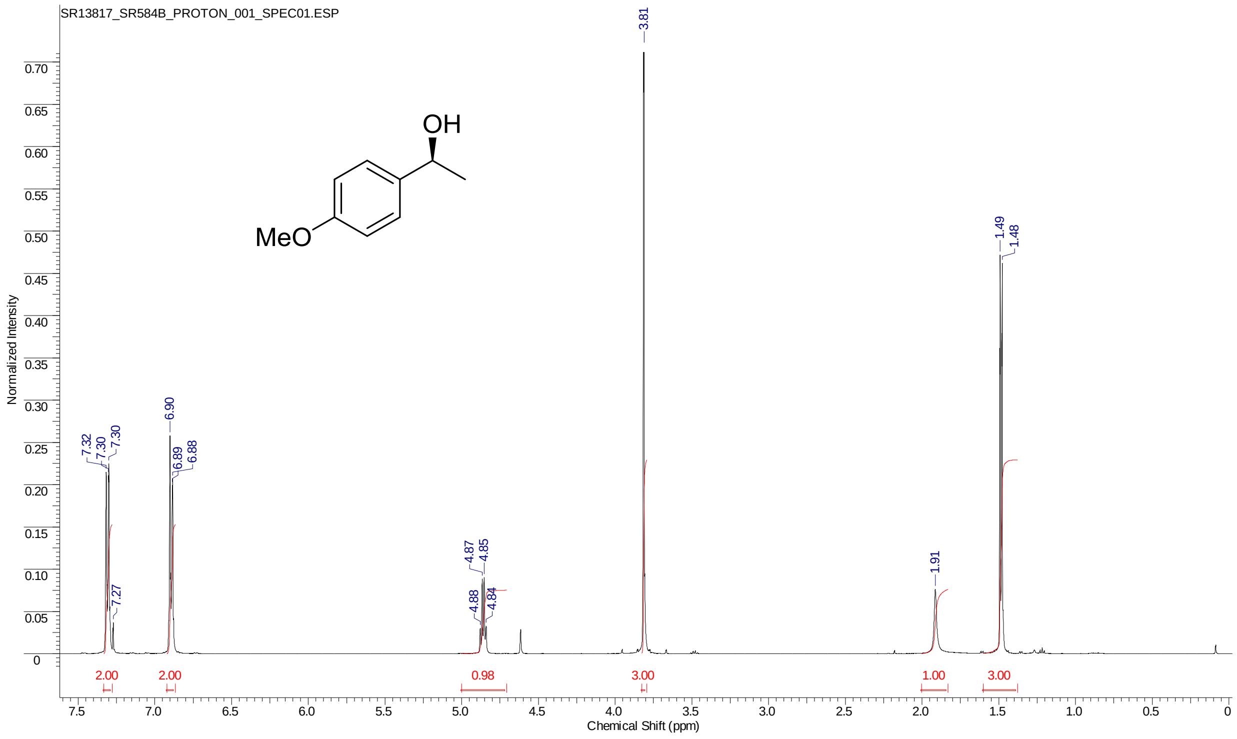

^{13}C NMR (126 MHz, $CDCl_3$)

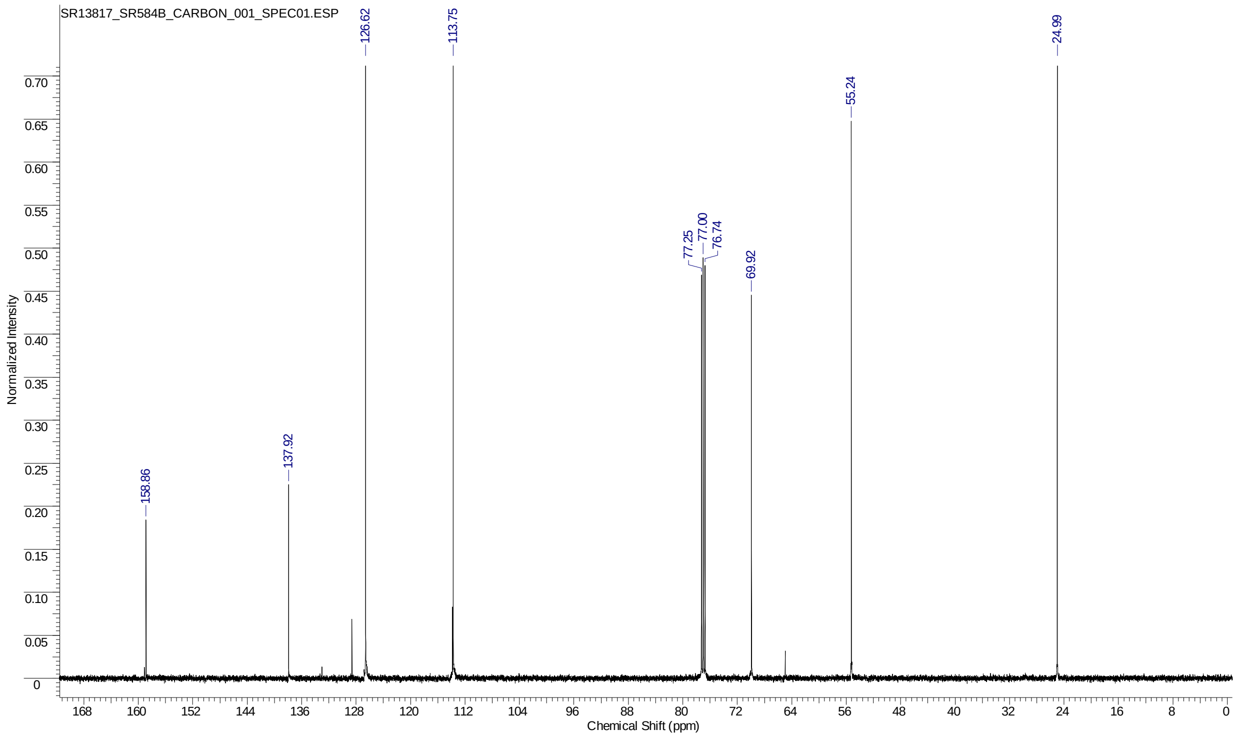

(*S*)-1-(4-Methoxyphenyl)propan-1-ol ((*S*)-43)

^{1}H NMR (400 MHz, $CDCl_3$)

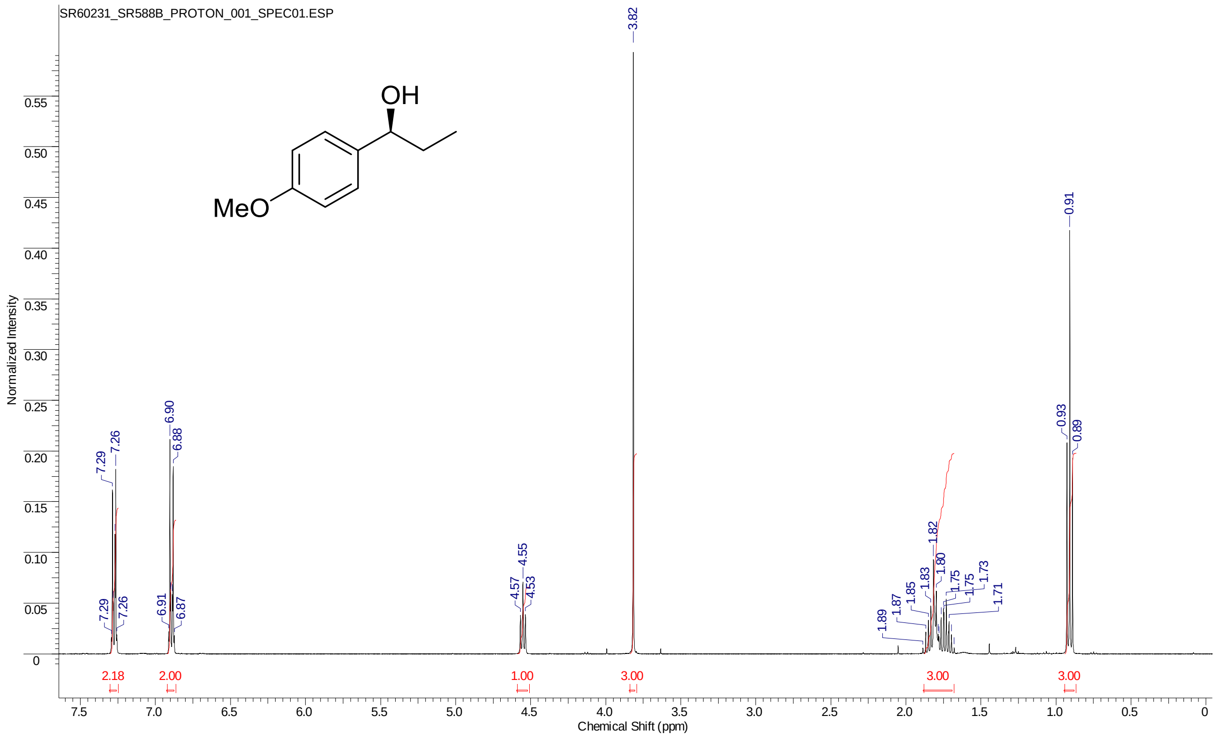

^{13}C NMR (101 MHz, $CDCl_3$)

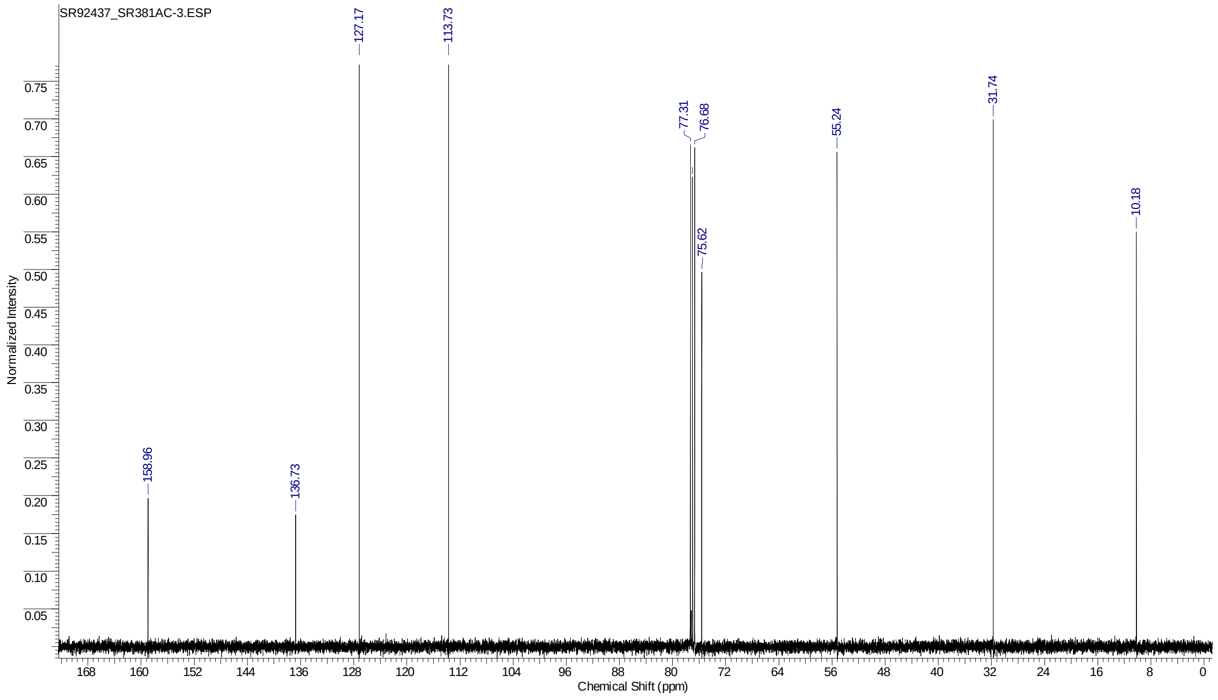

1-(3-Fluoro-4-methoxyphenyl)ethanol (44)

^{1}H NMR (400 MHz, $CDCl_3$)

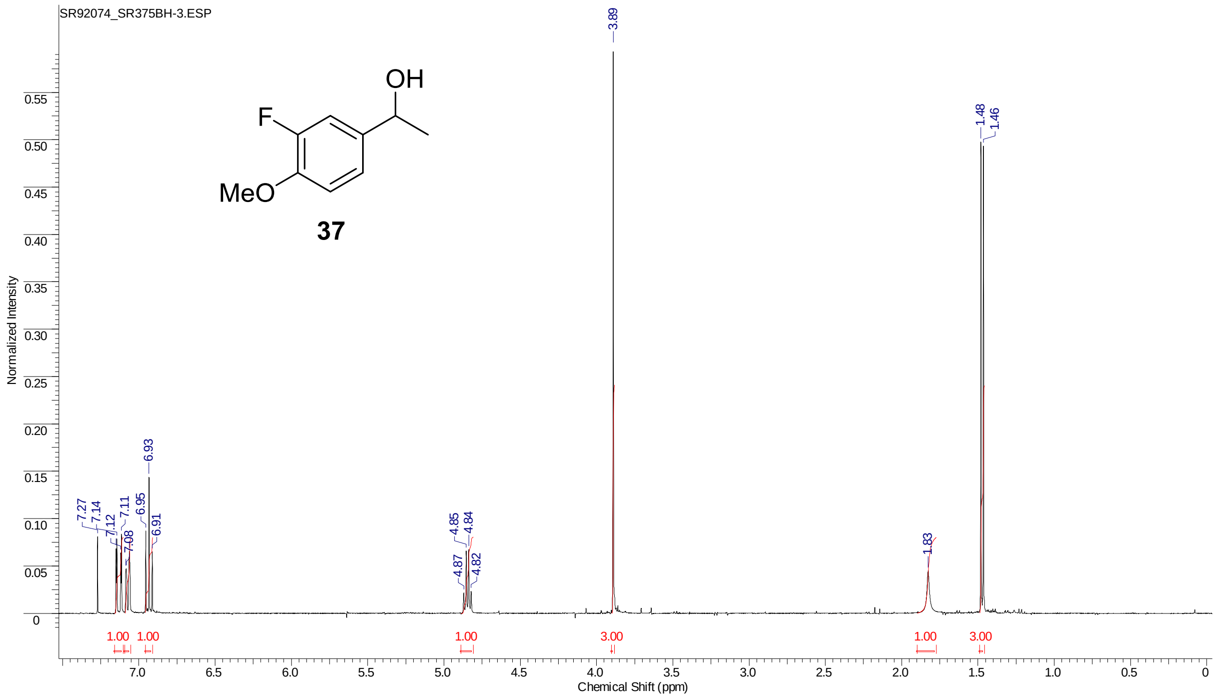

^{13}C NMR (101 MHz, $CDCl_3$)

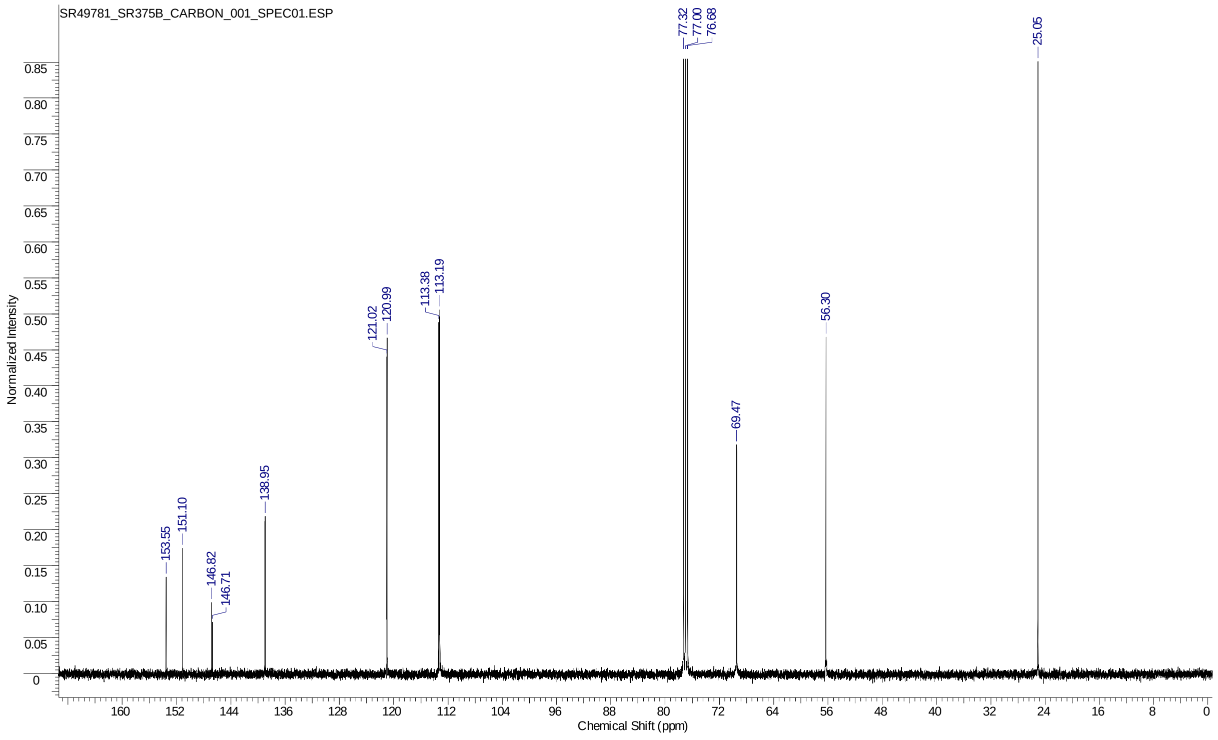

Propyl diisopropylcarbamate (28)

^{1}H NMR (400 MHz, $CDCl_3$)

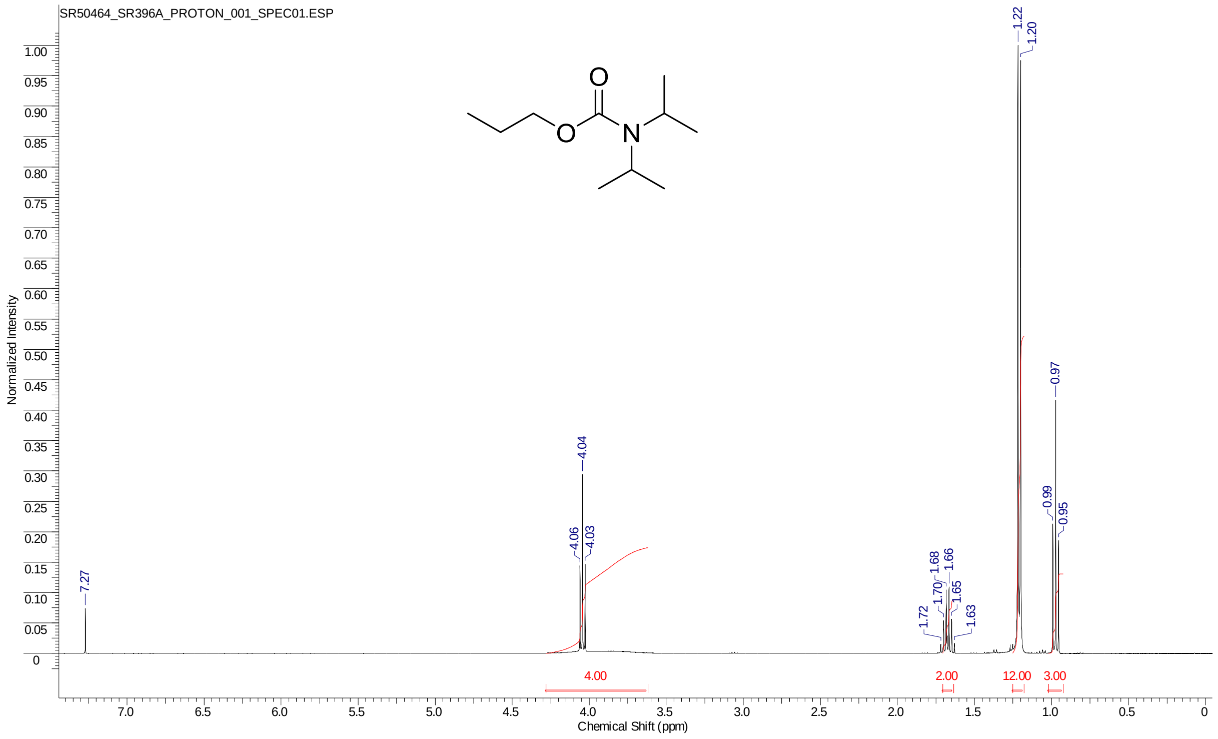

^{13}C NMR (101 MHz, $CDCl_3$)

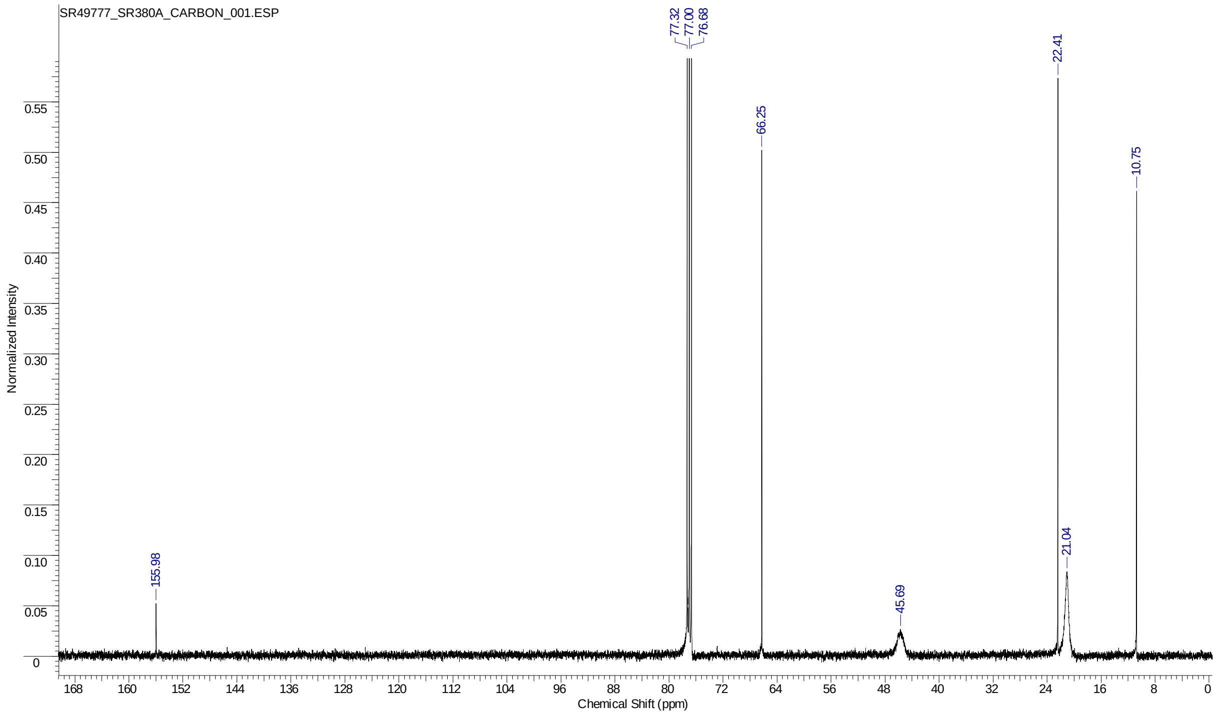

(*S*)-1-Phenylethyl diisopropylcarbamate ((*S*)-5)

^{1}H NMR (400 MHz, $CDCl_3$)

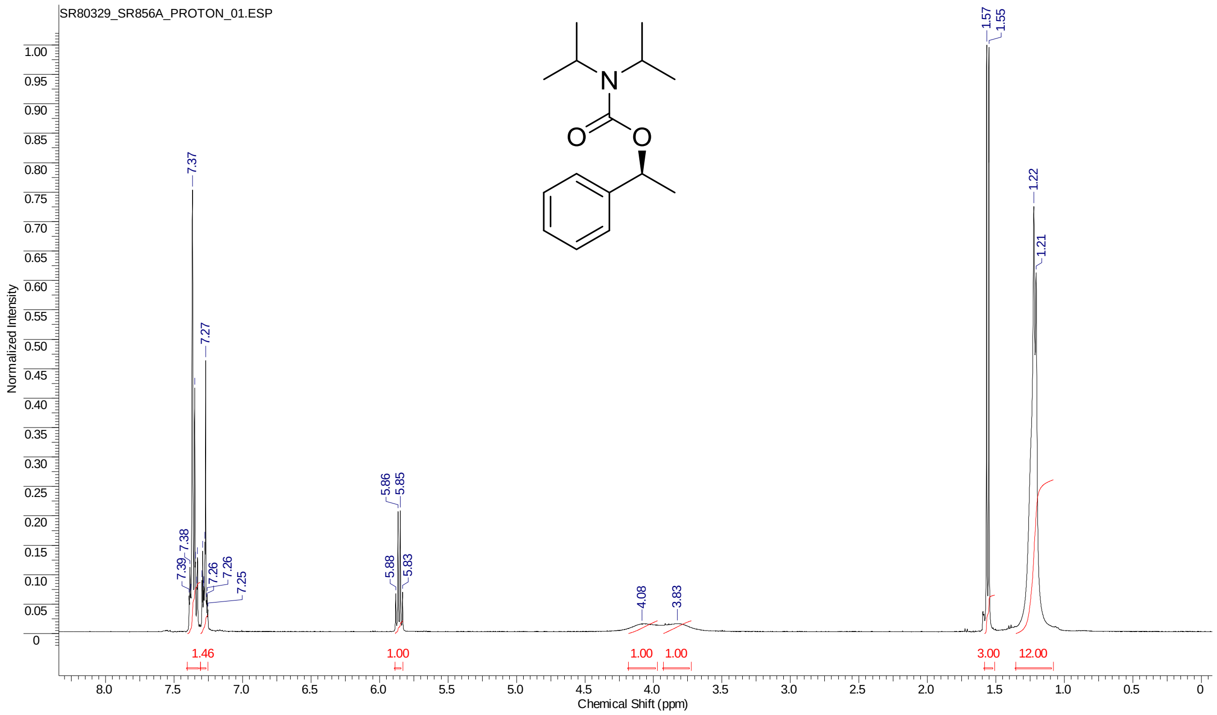

^{13}C NMR (101 MHz, $CDCl_3$)

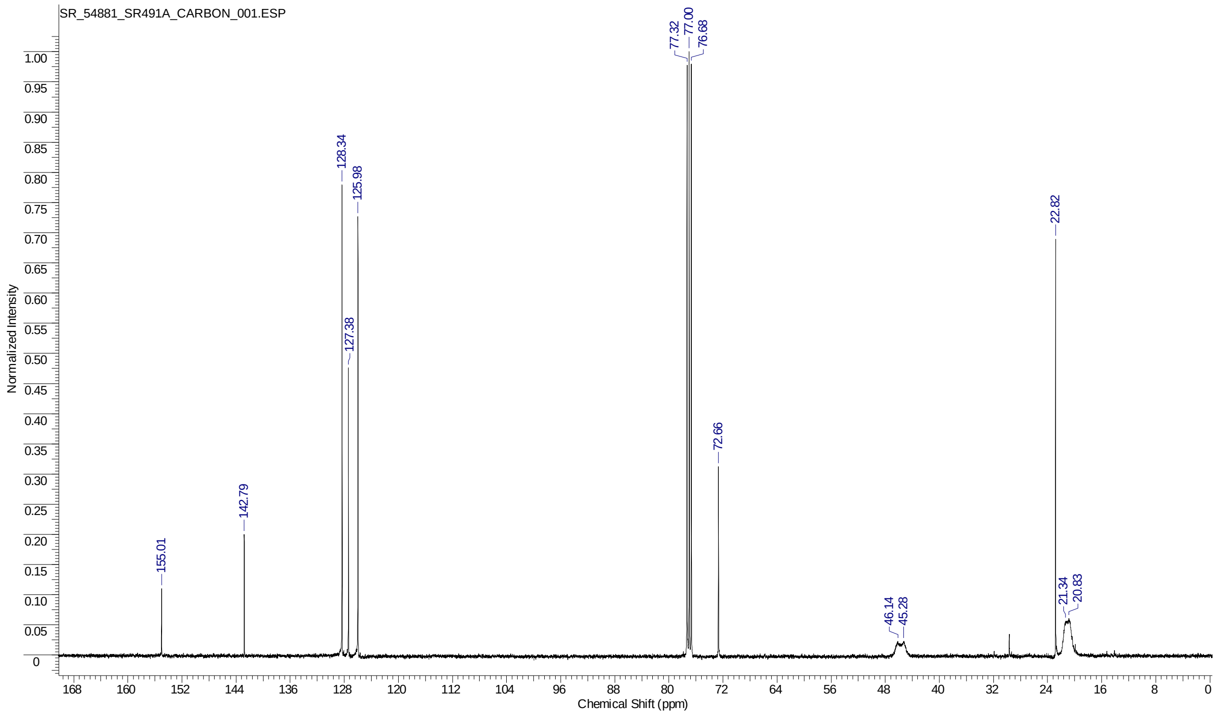

(*S*)-1-Phenylpropyl diisopropylcarbamate ((*S*)-6)

^{1}H NMR (500 MHz, $CDCl_3$)

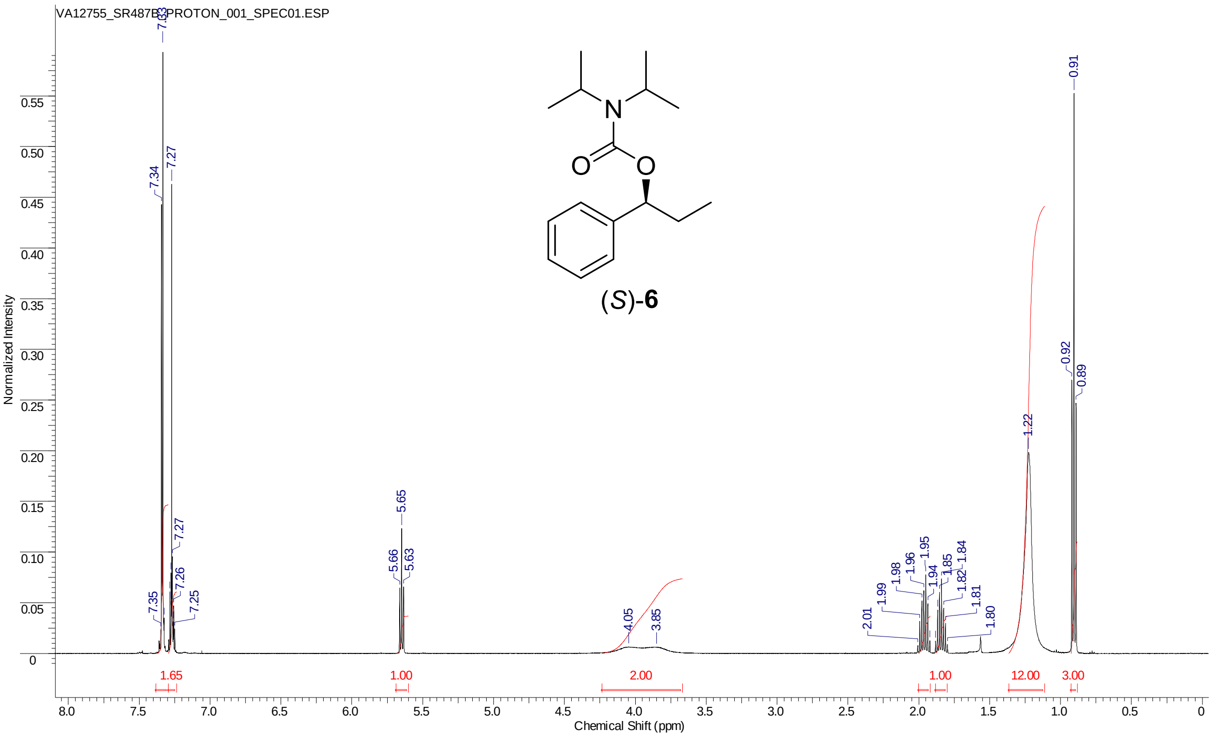

^{13}C NMR (126 MHz, $CDCl_3$)

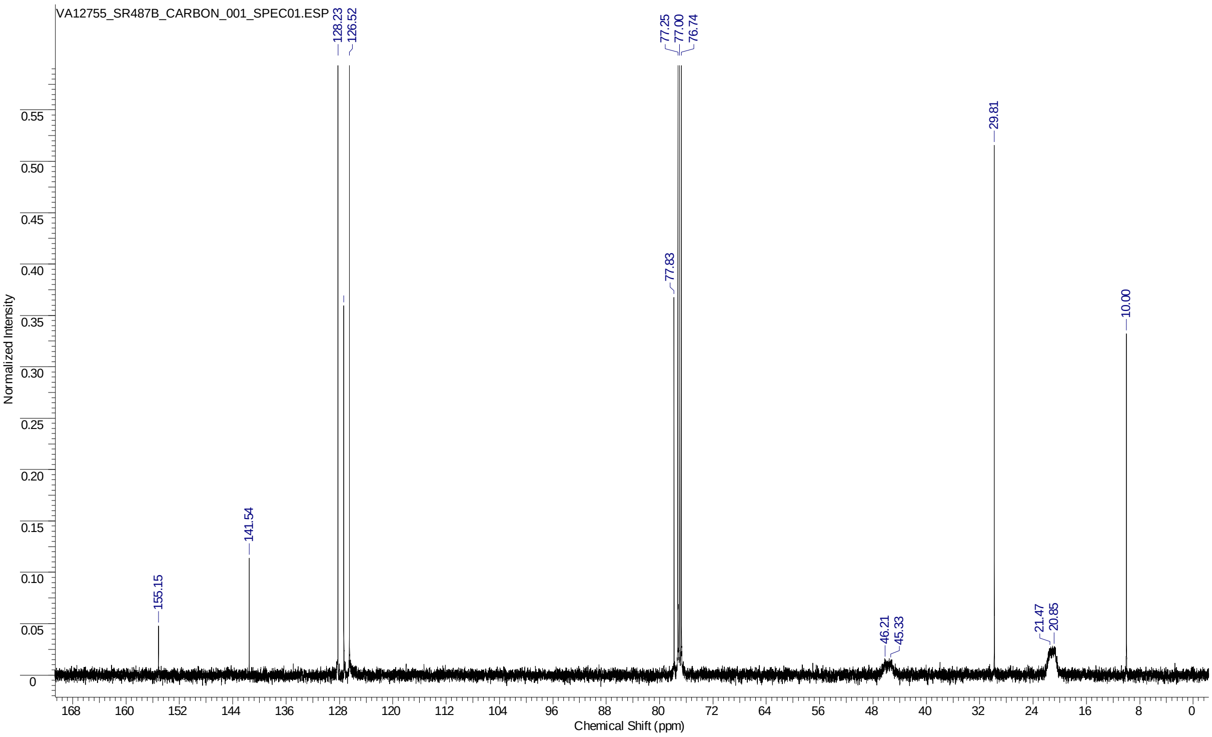

(*S*)-1-(4-Methoxyphenyl)ethyl diisopropylcarbamate ((*S*)-32)

^{1}H NMR (400 MHz, $CDCl_3$)

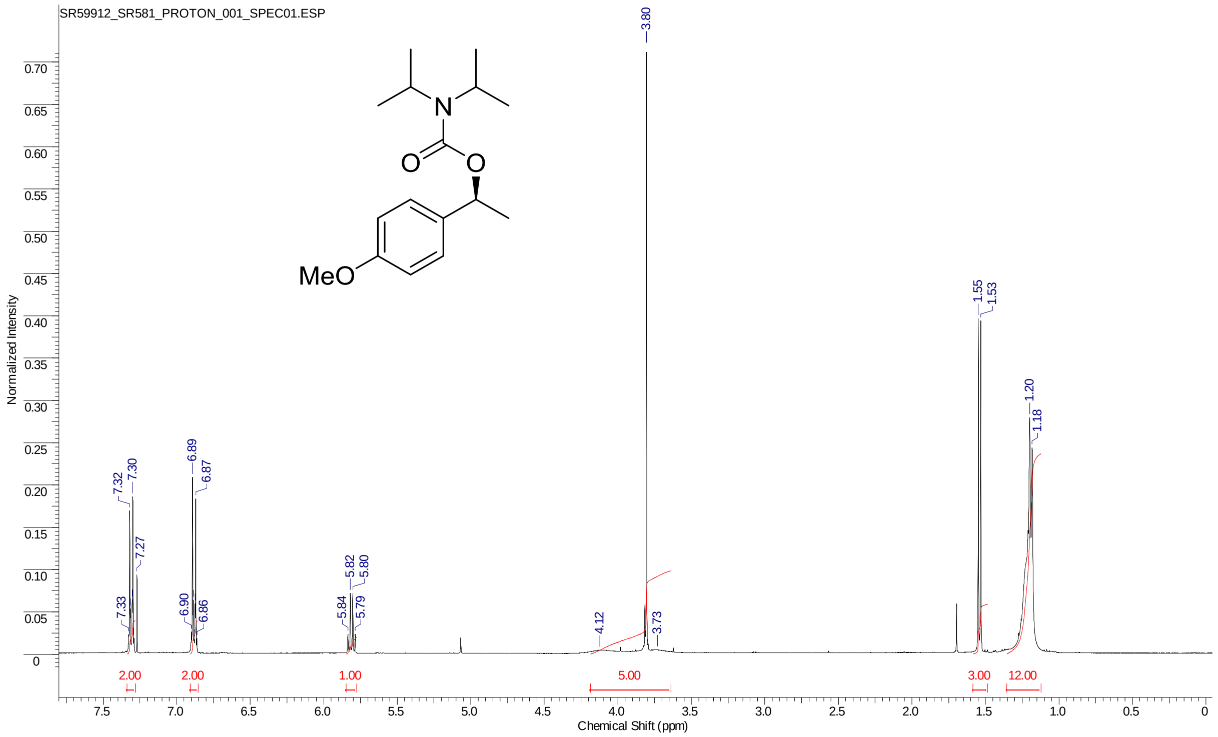

^{13}C NMR (101 MHz, $CDCl_3$)

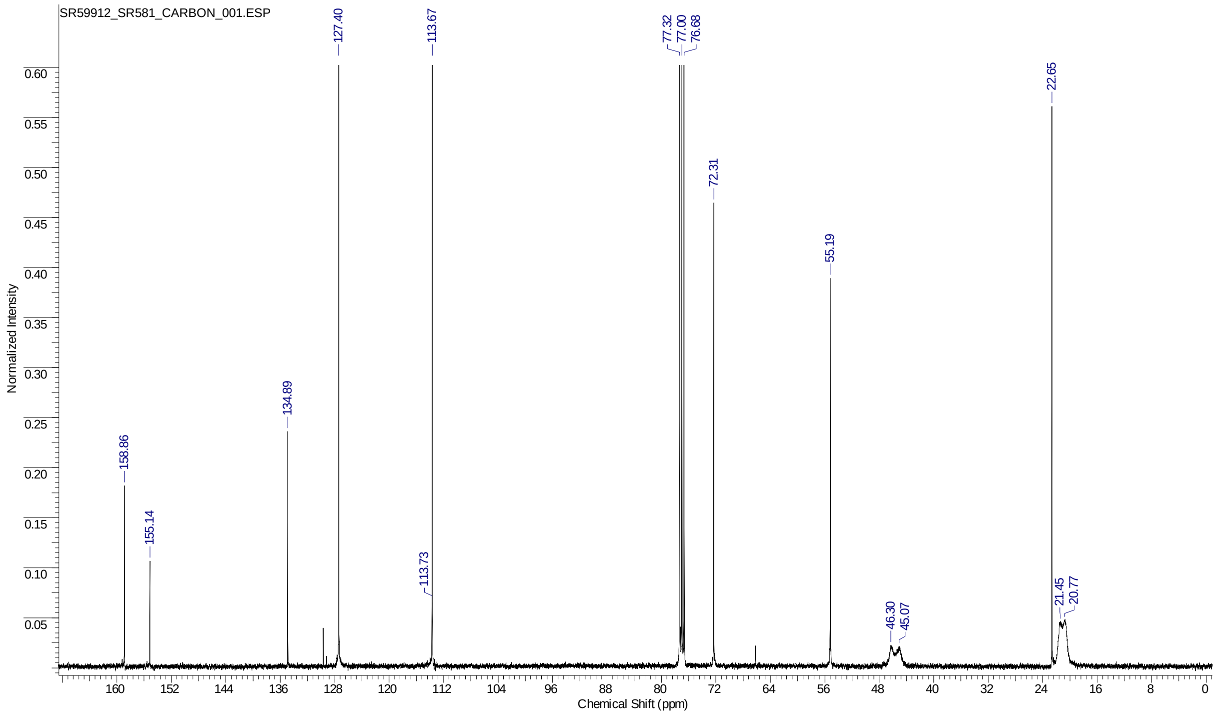

(*R*)-1-(4-Methoxyphenyl)propyl diisopropylcarbamate ((*R*)-39)

^{1}H NMR (400 MHz, $CDCl_3$)

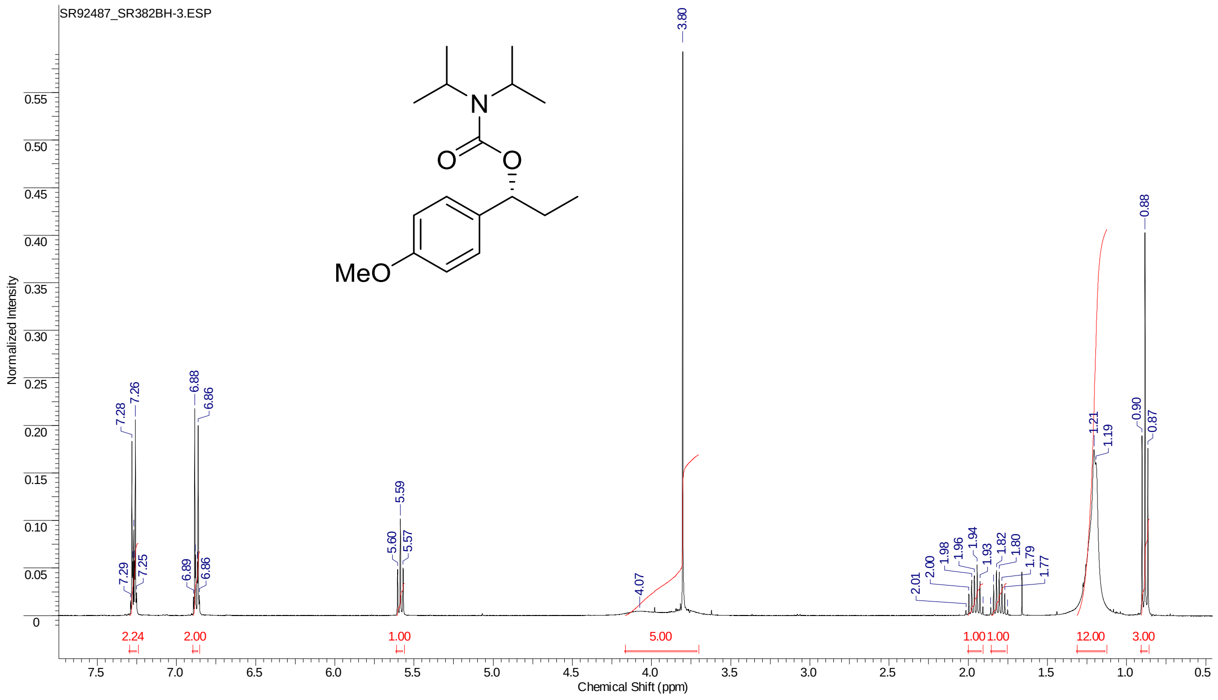

^{13}C NMR (101 MHz, $CDCl_3$)

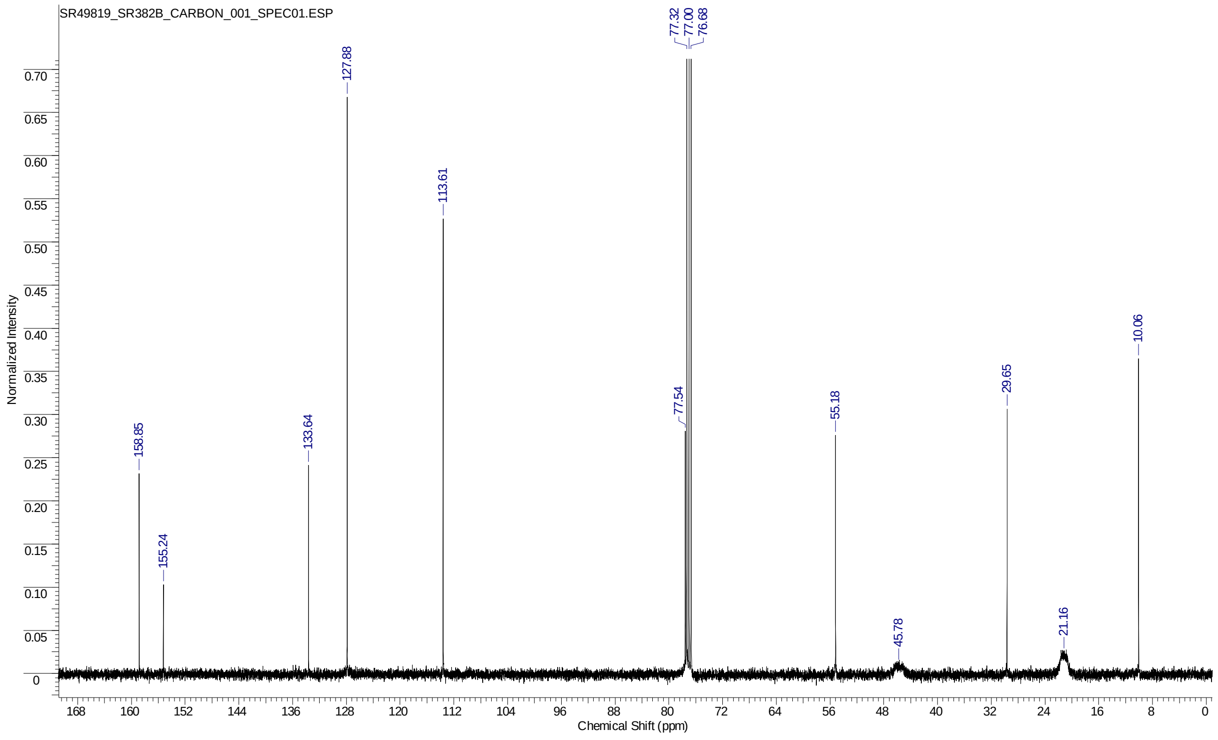

1-(3-Fluoro-4-methoxyphenyl)ethyl diisopropylcarbamate (36)

^{1}H NMR (400 MHz, $CDCl_3$)

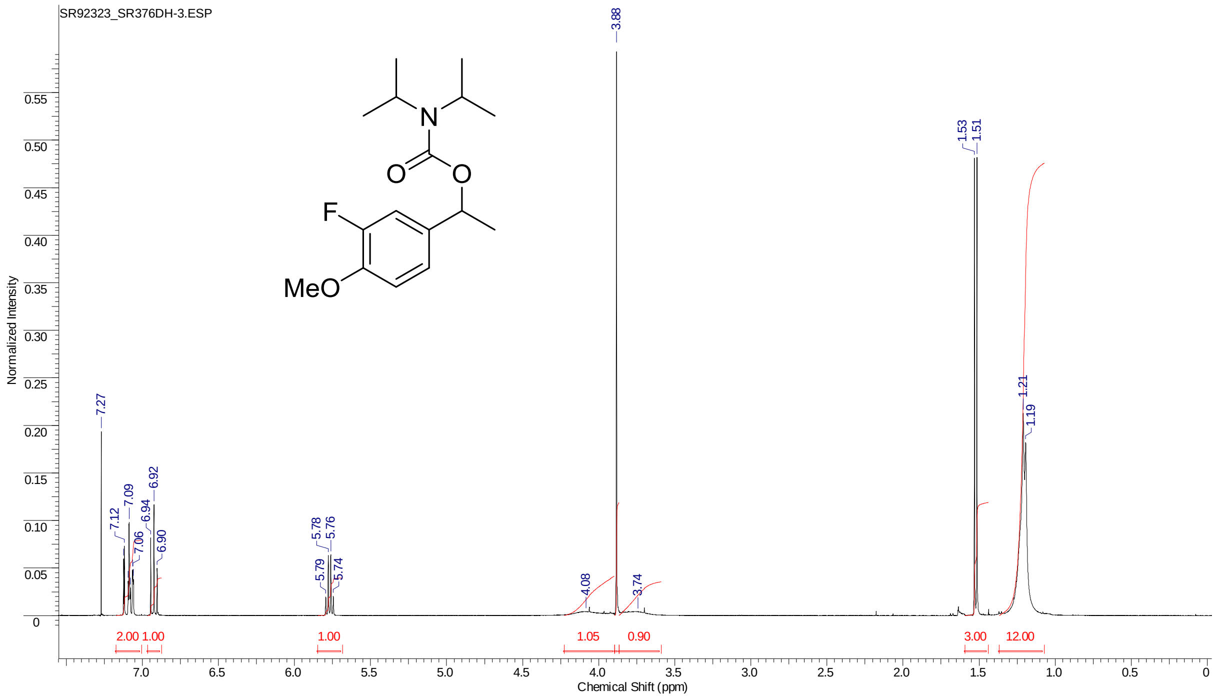

^{13}C NMR (101 MHz, $CDCl_3$)

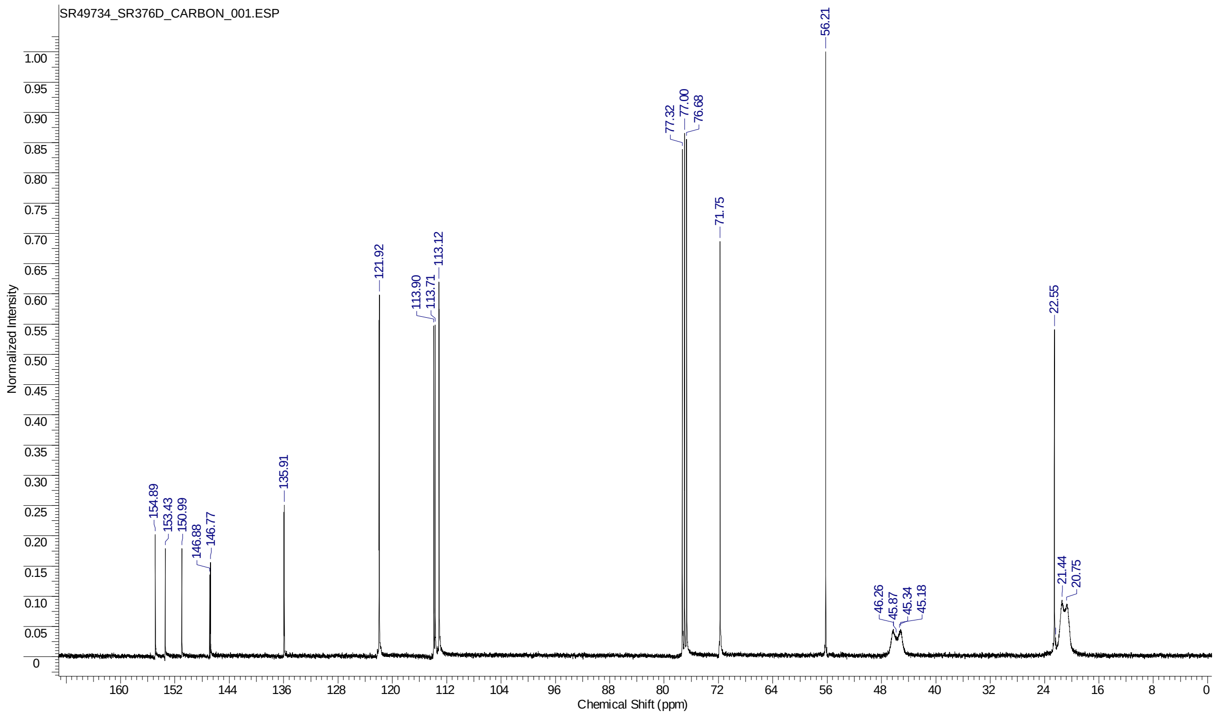

2-(3-Fluoro-4-methoxyphenyl)-4,4,5,5-tetramethyl-1,3,2-dioxaborolane (29)

^{1}H NMR (400 MHz, $CDCl_3$)

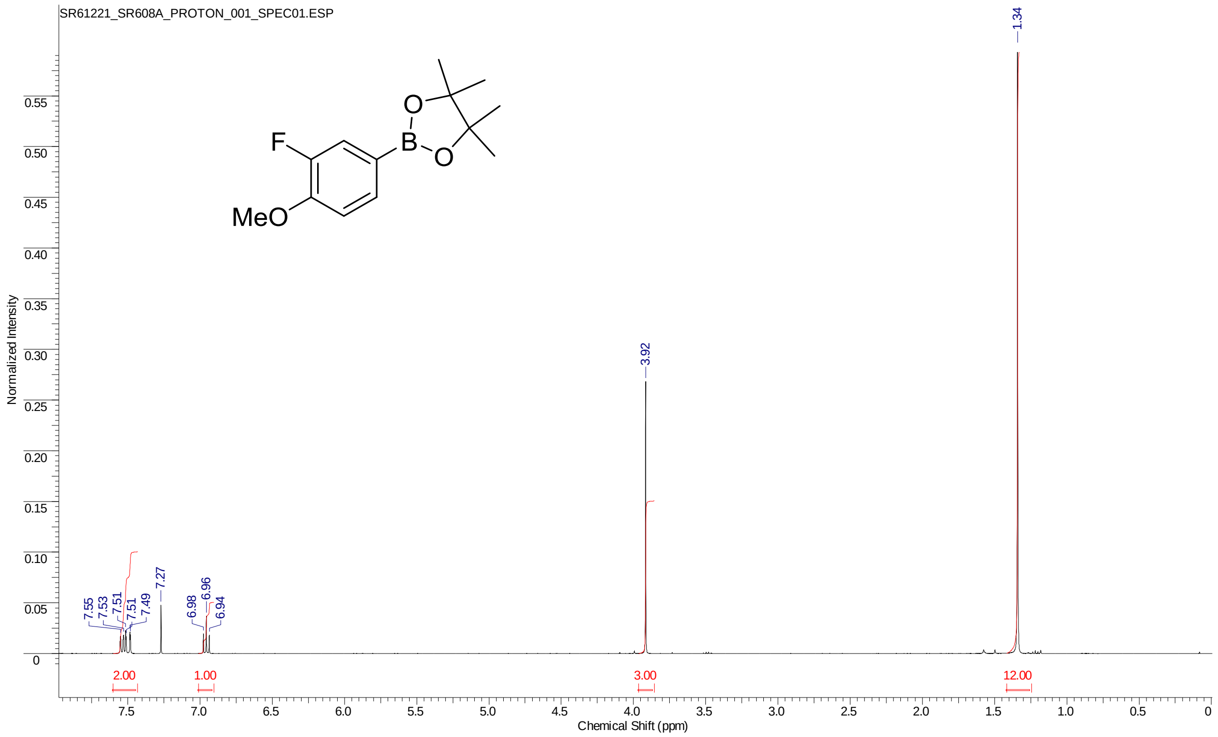

^{13}C NMR (101 MHz, $CDCl_3$)

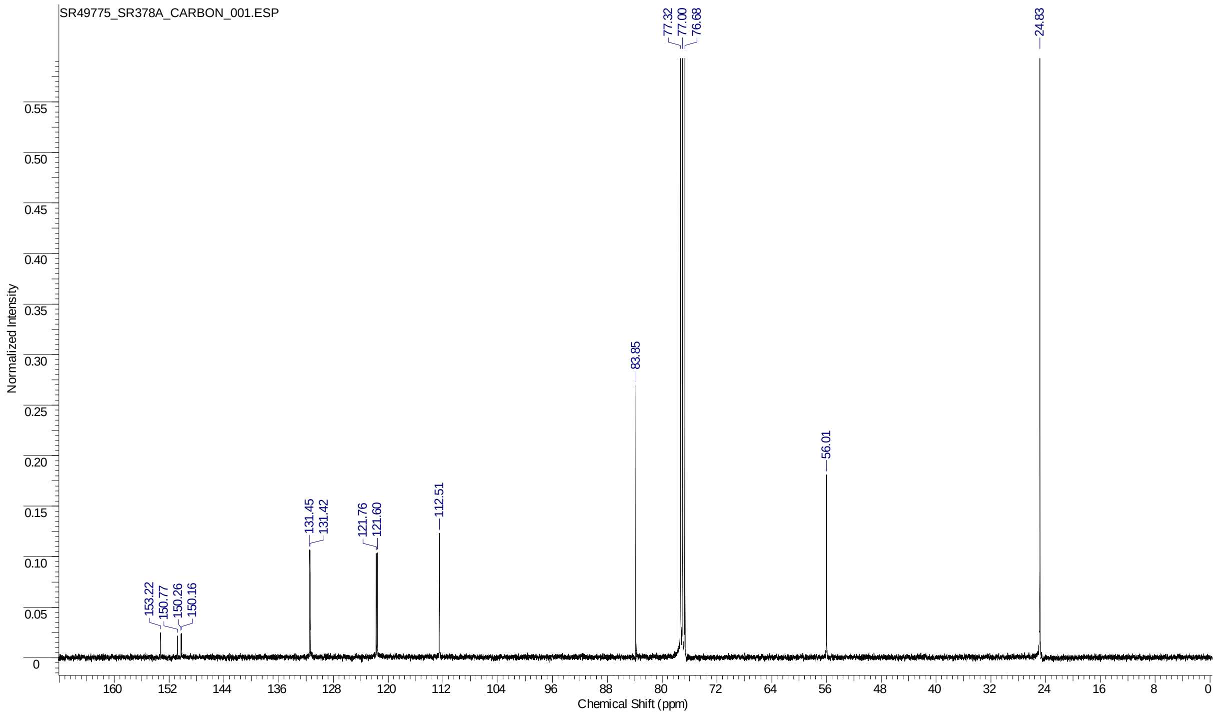

2-(3-fluoro-4-methoxyphenyl)-5,5-dimethyl-1,3,2-dioxaborinane (38)

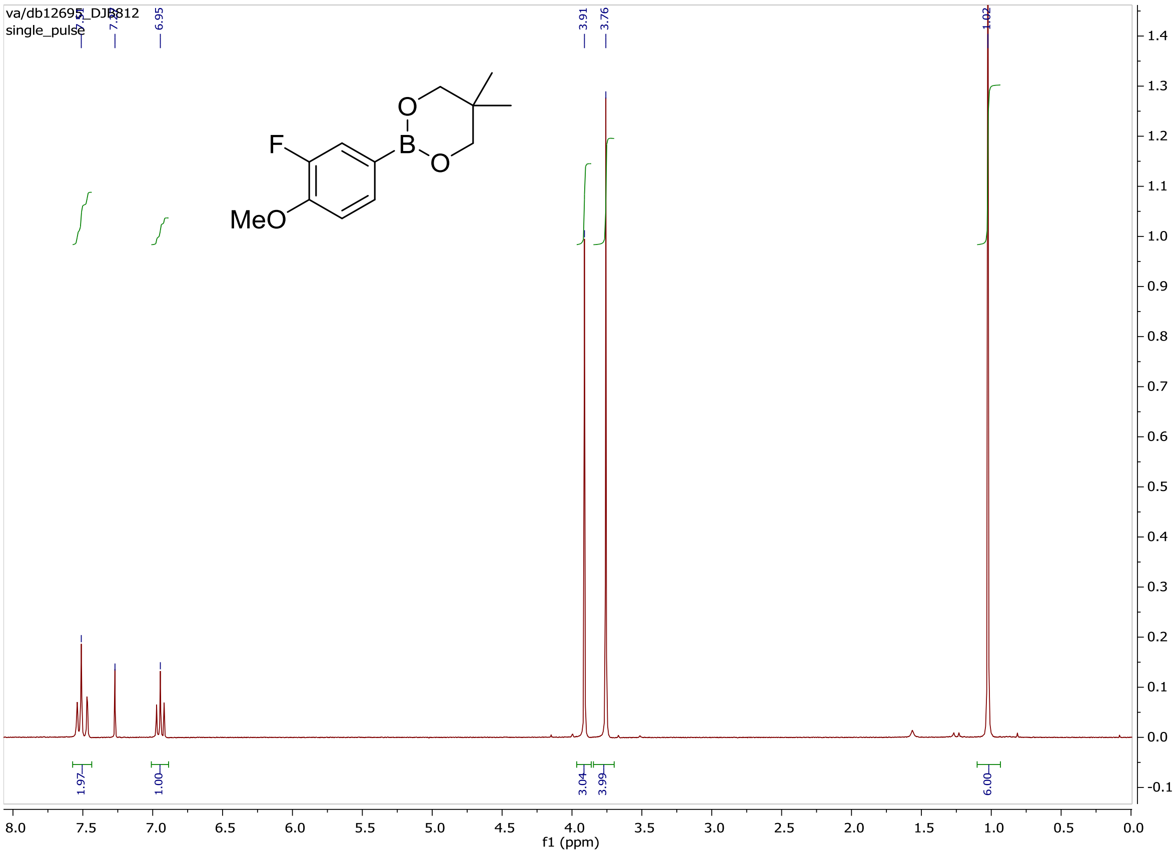

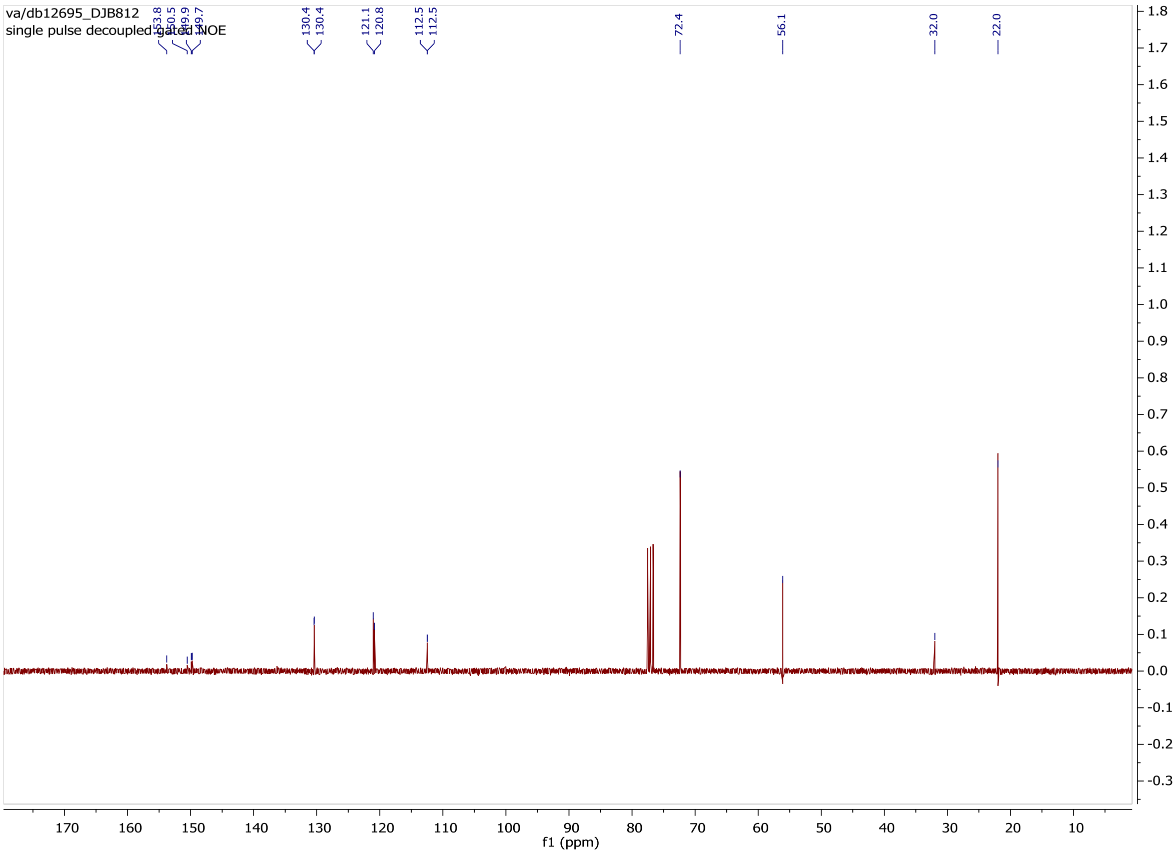

(*S*)-4,4,5,5-Tetramethyl-2-(1-phenylethyl)-1,3,2-dioxaborolane ((*S*)-8)

^{1}H NMR (500 MHz, $CDCl_3$)

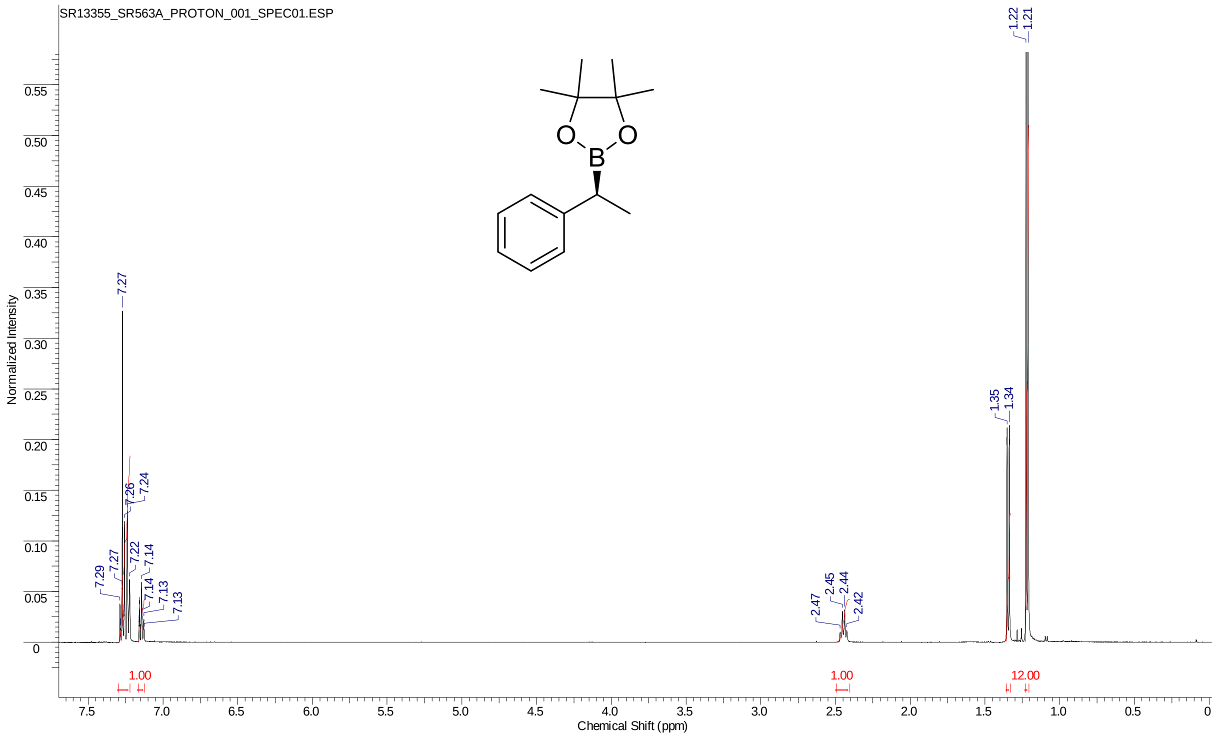

^{13}C NMR (126 MHz, $CDCl_3$)

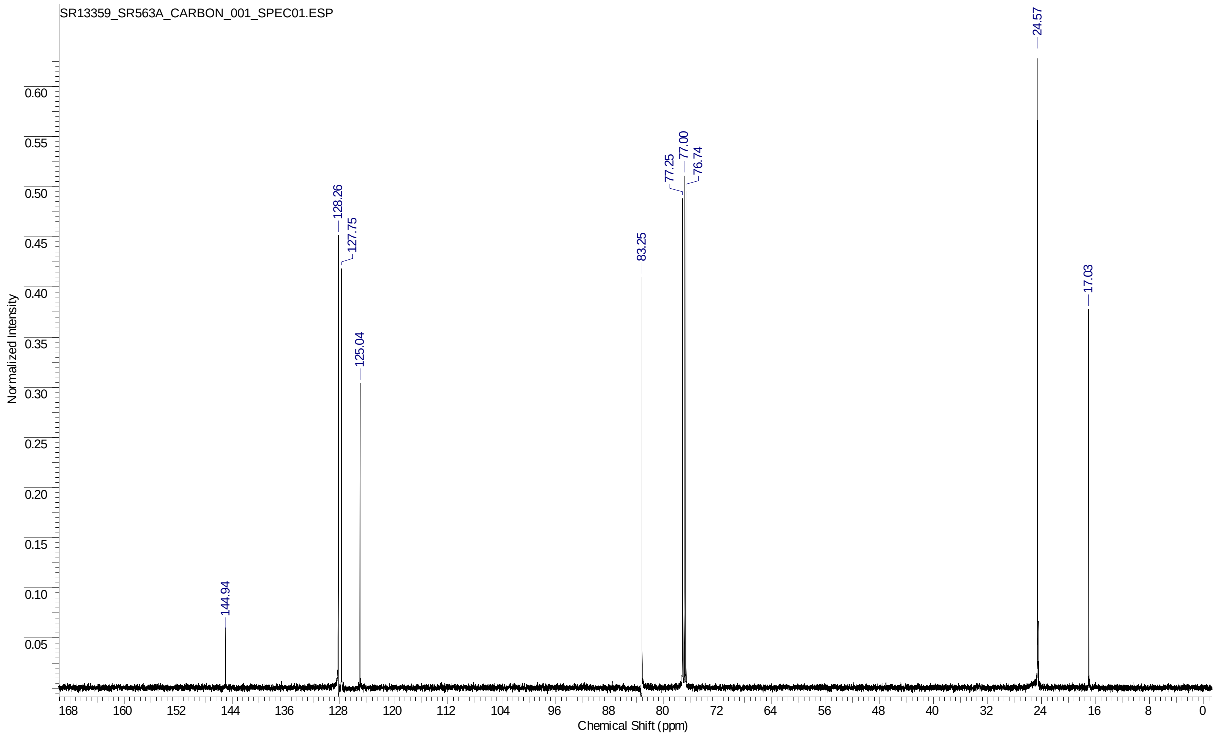

4,4,5,5-Tetramethyl-2-(1-phenylpropyl)-1,3,2-dioxaborolane (9)

^{1}H NMR (400 MHz, $CDCl_3$)

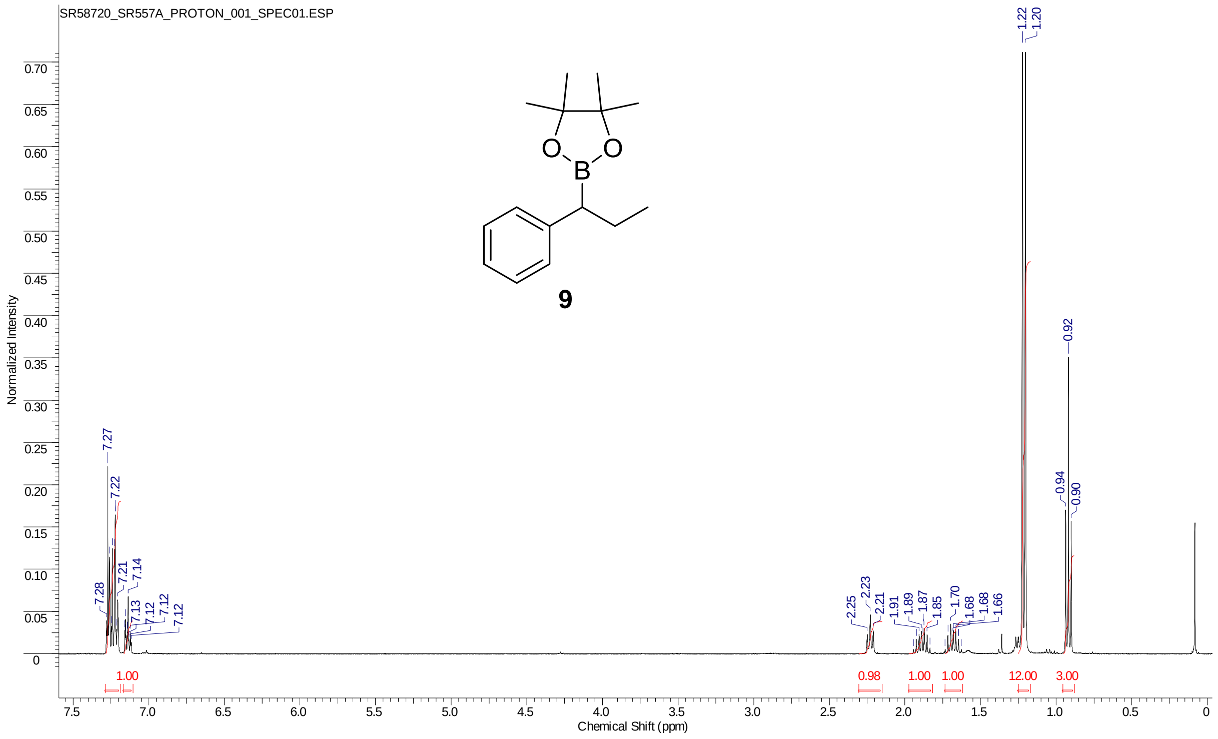

^{13}C NMR (126 MHz, $CDCl_3$)

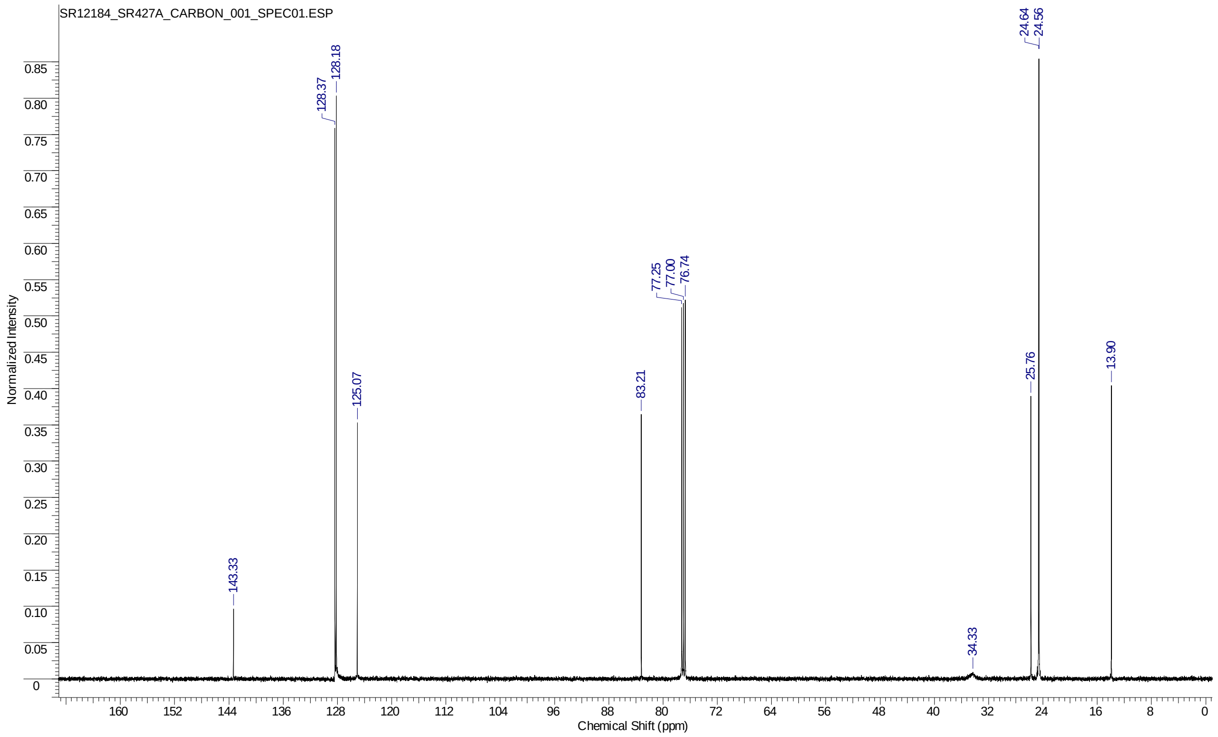

(*S*)-5,5-dimethyl-2-(1-phenylpropyl)-1,3,2-dioxaborinane ((*S*)-11)

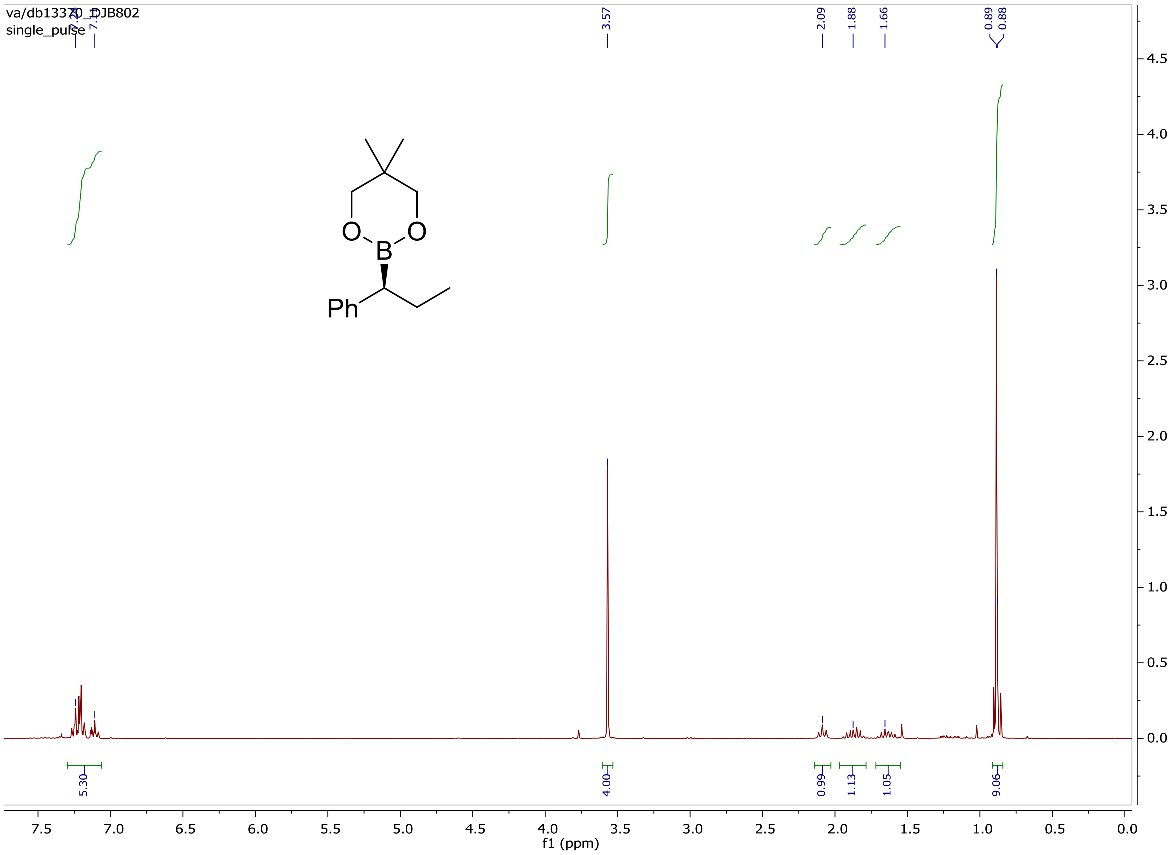

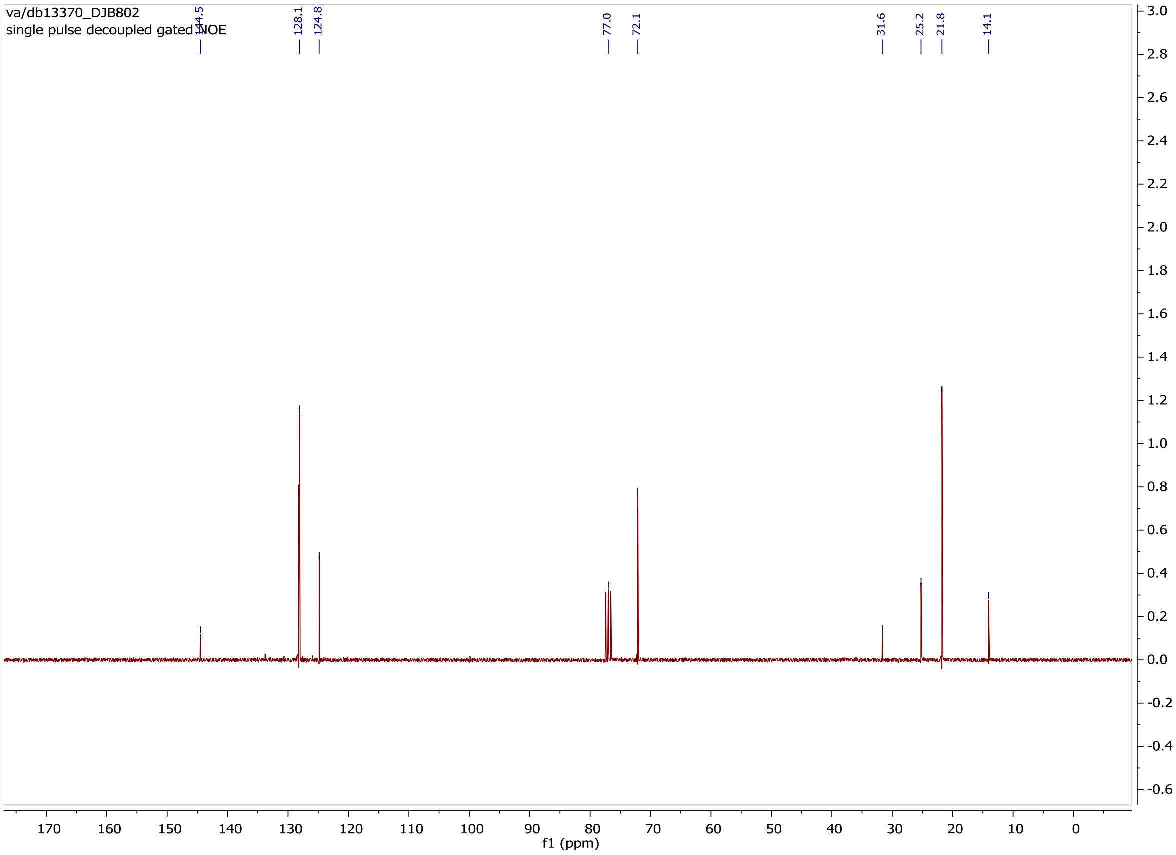

(*R*)-2-(1-(3-fluoro-4-methoxyphenyl)propyl)-5,5-dimethyl-1,3,2-dioxaborinane ((*R*)-40)

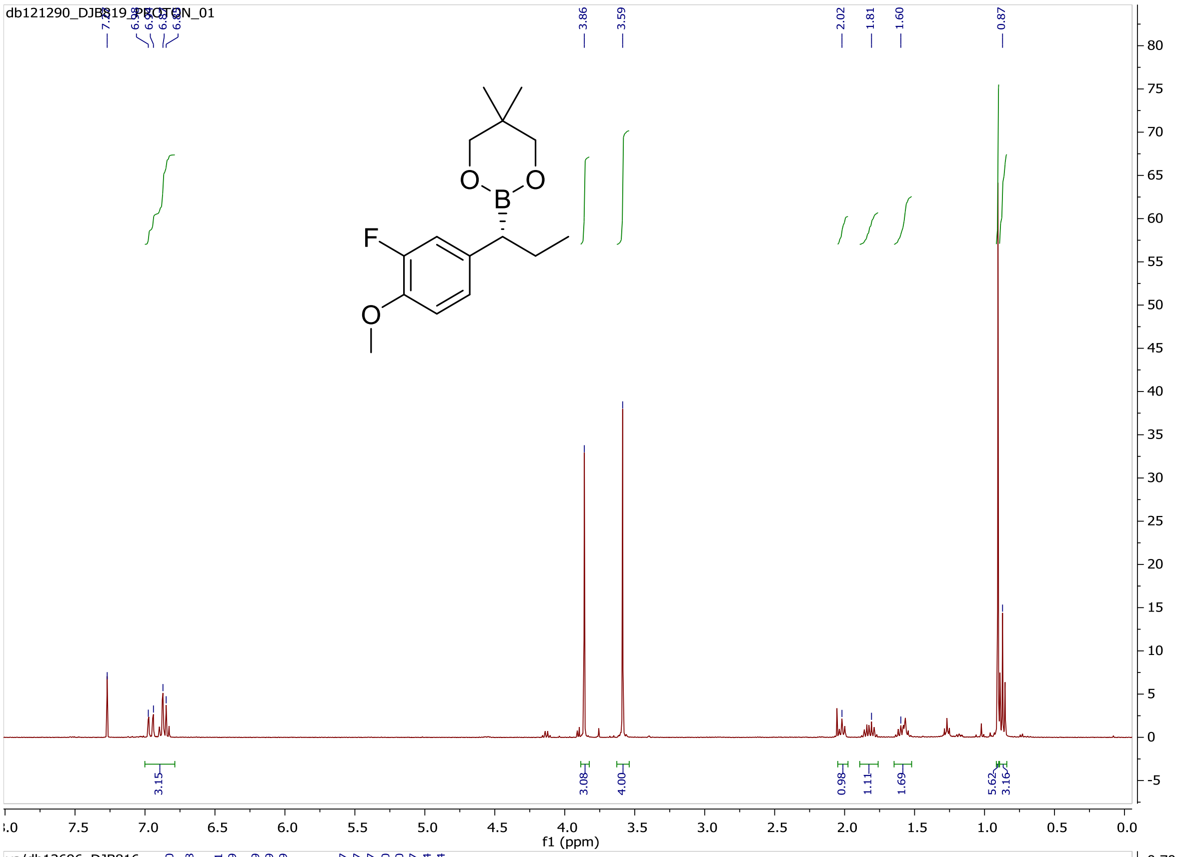

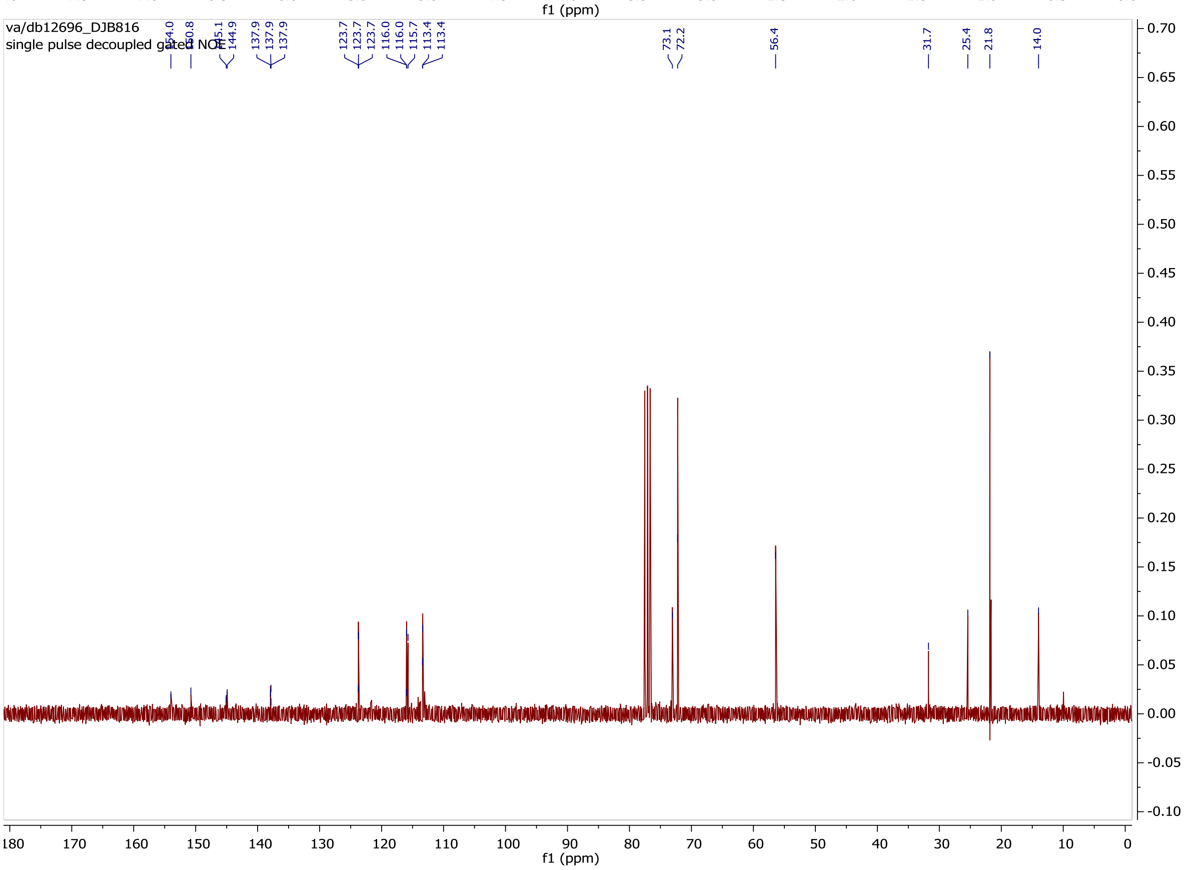

(*S*)-2-(1-(3-Fluoro-4-methoxyphenyl)propyl)-4,4,5,5-tetramethyl-1,3,2-dioxaborolane ((*S*)-30)

^{1}H NMR (400 MHz, $CDCl_3$)

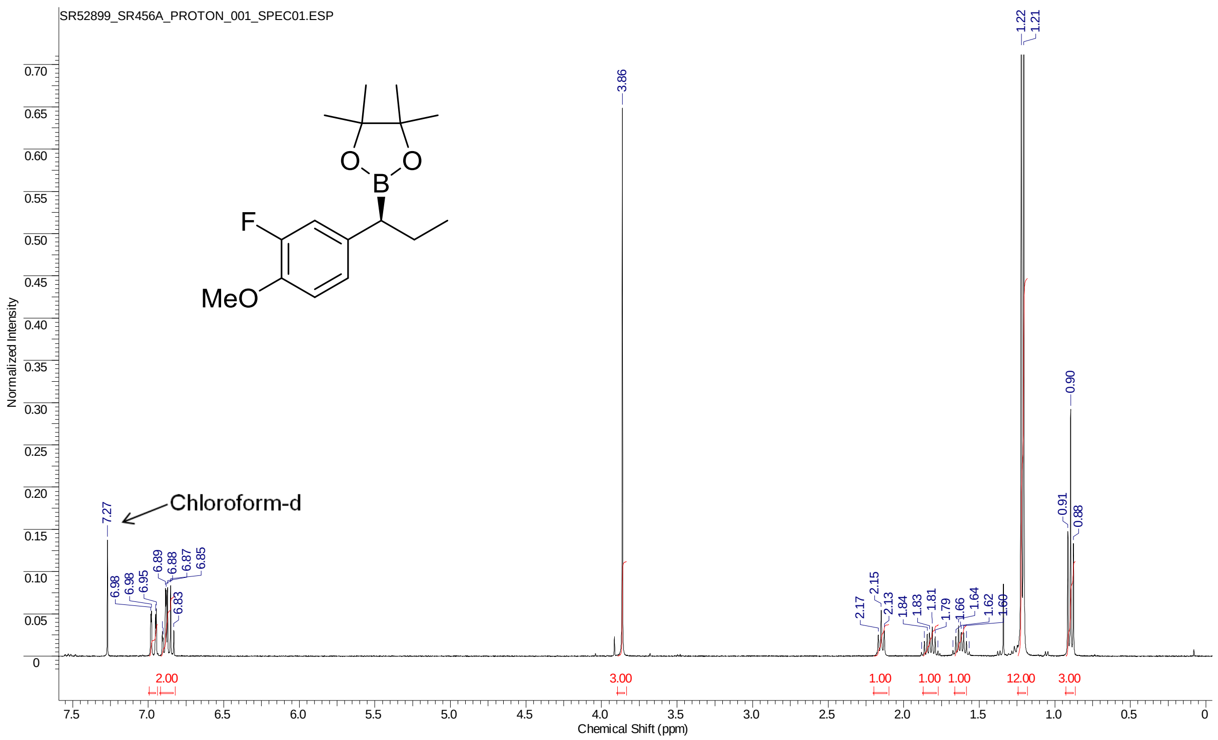

^{13}C NMR (101 MHz, $CDCl_3$)

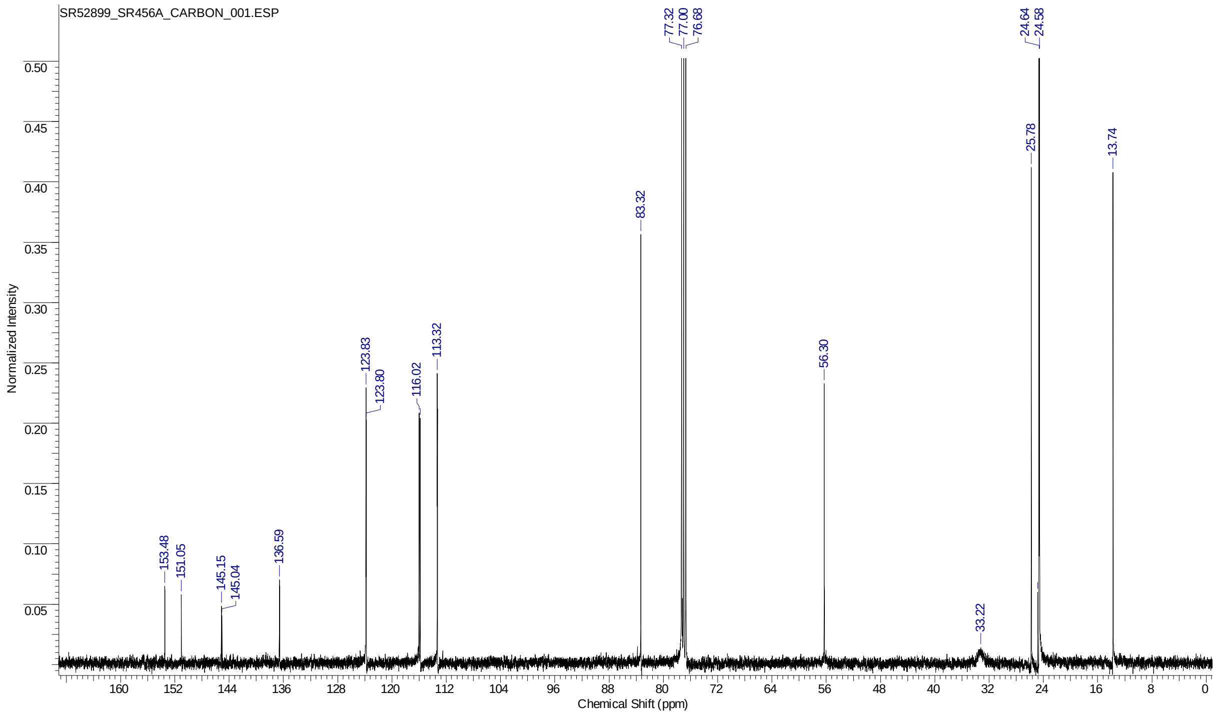

(*S*)-1-(3-Fluoro-4-methoxyphenyl)propan-1-ol ((*S*)-45)

^{1}H NMR (400 MHz, $CDCl_3$)

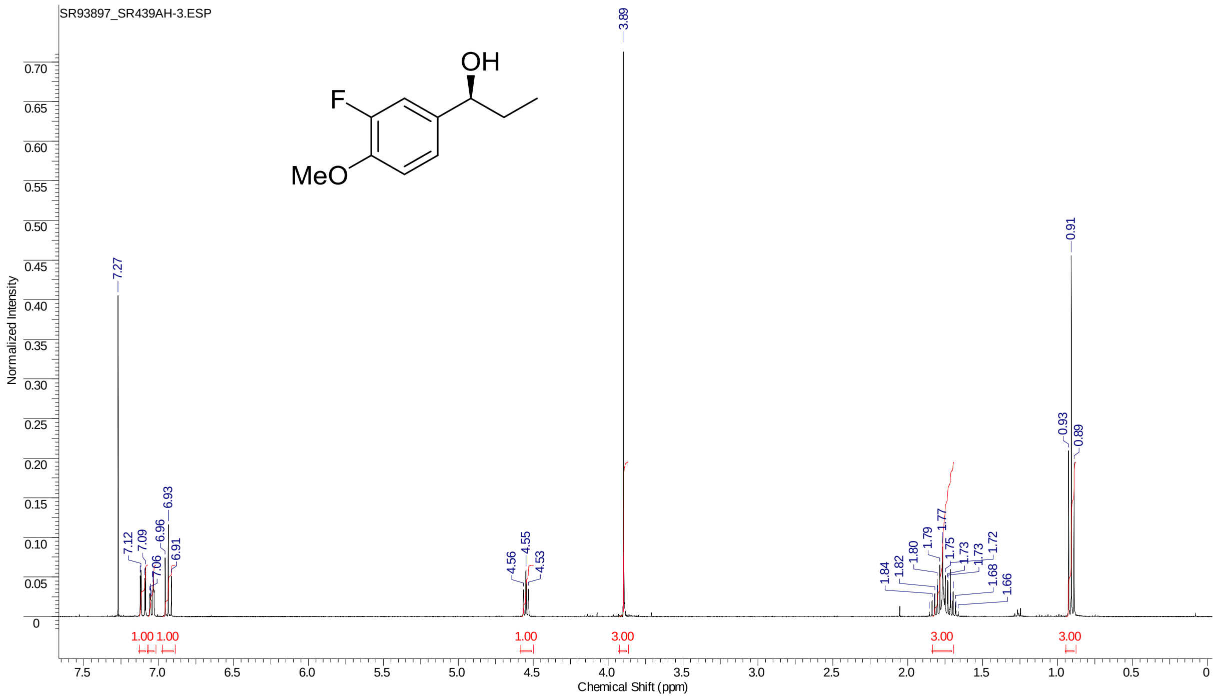

^{13}C NMR (126 MHz, $CDCl_3$)

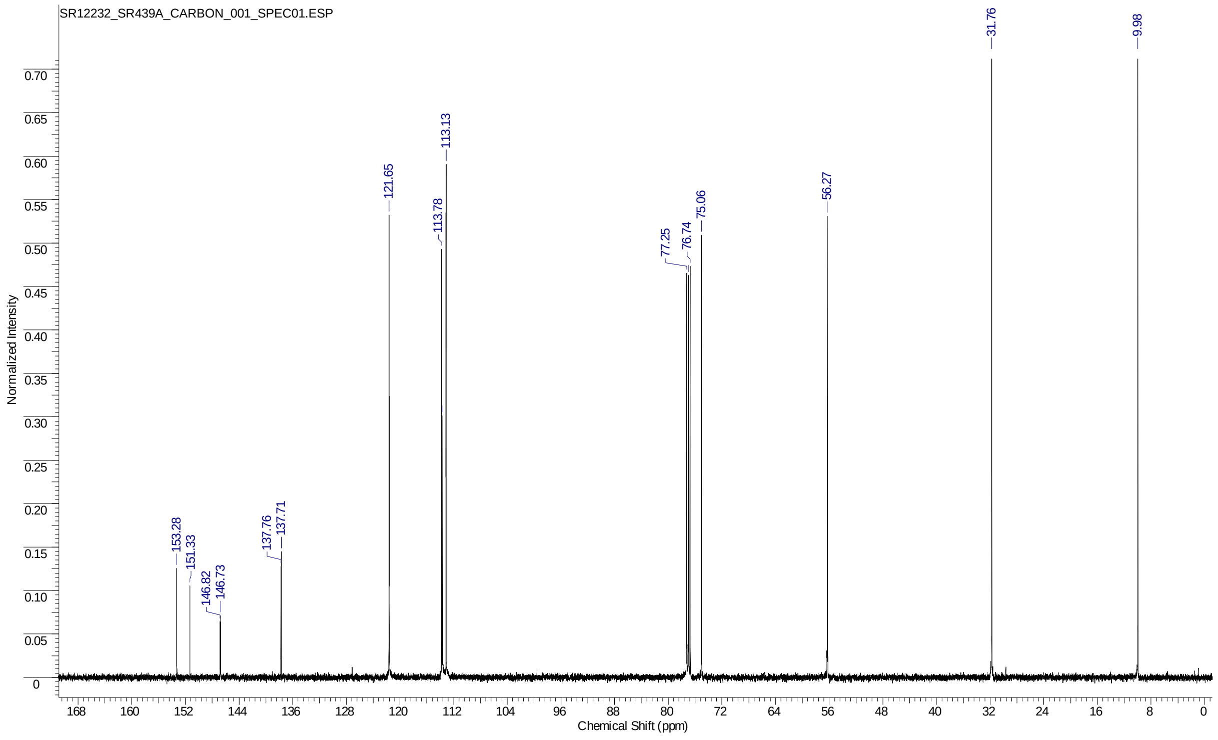

4,4,5,5-Tetramethyl-2-(pentan-3-yl)-1,3,2-dioxaborolane (10)

^{1}H NMR (400 MHz, $CDCl_3$)

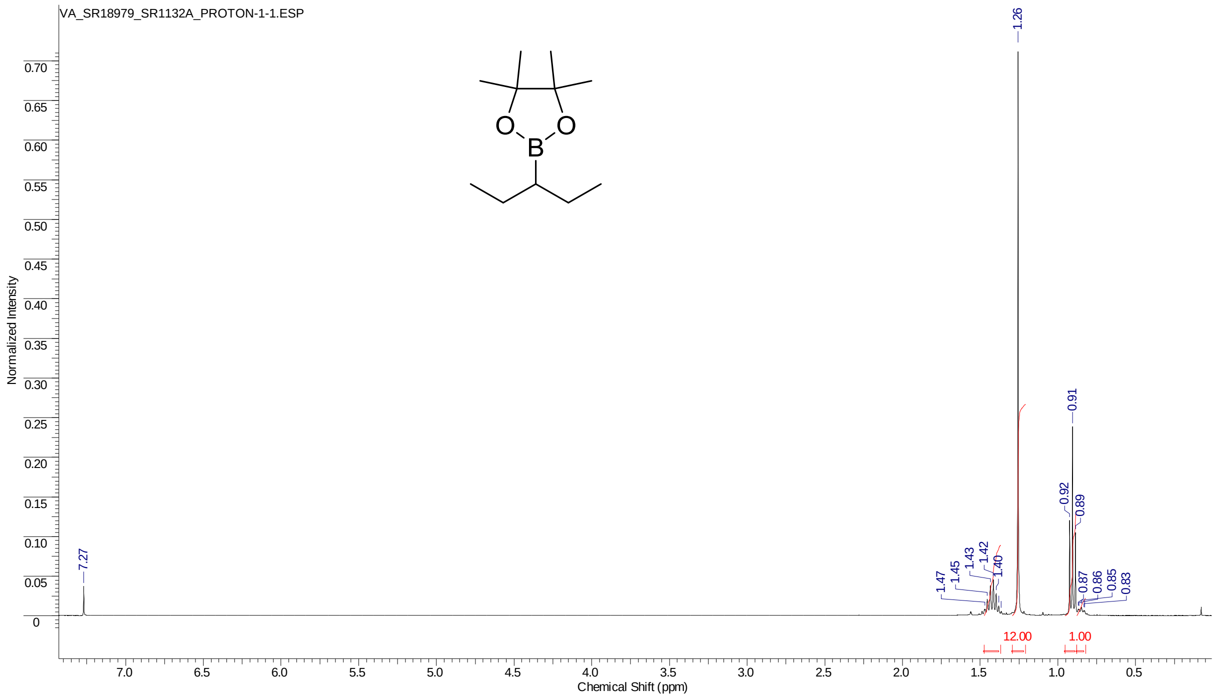

^{13}C NMR (101 MHz, $CDCl_3$)

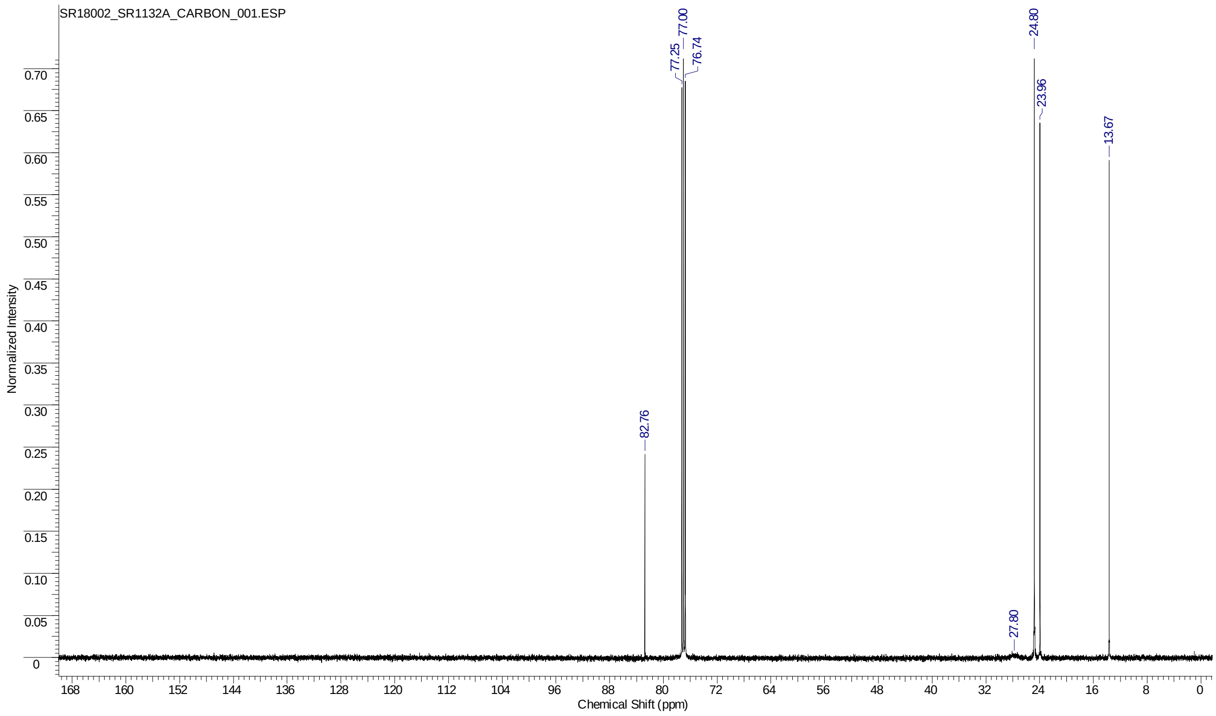

5,5-dimethyl-2-(pentan-3-yl)-1,3,2-dioxaborinane (12)

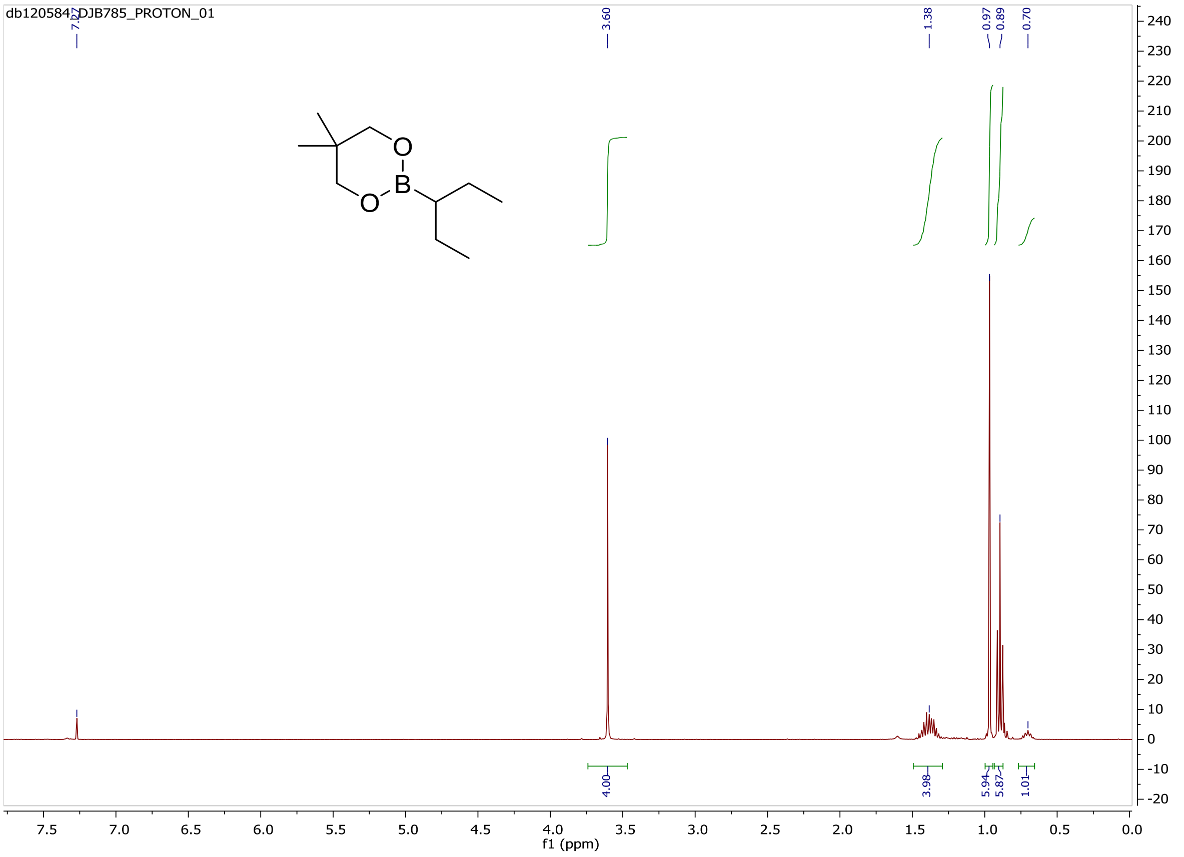

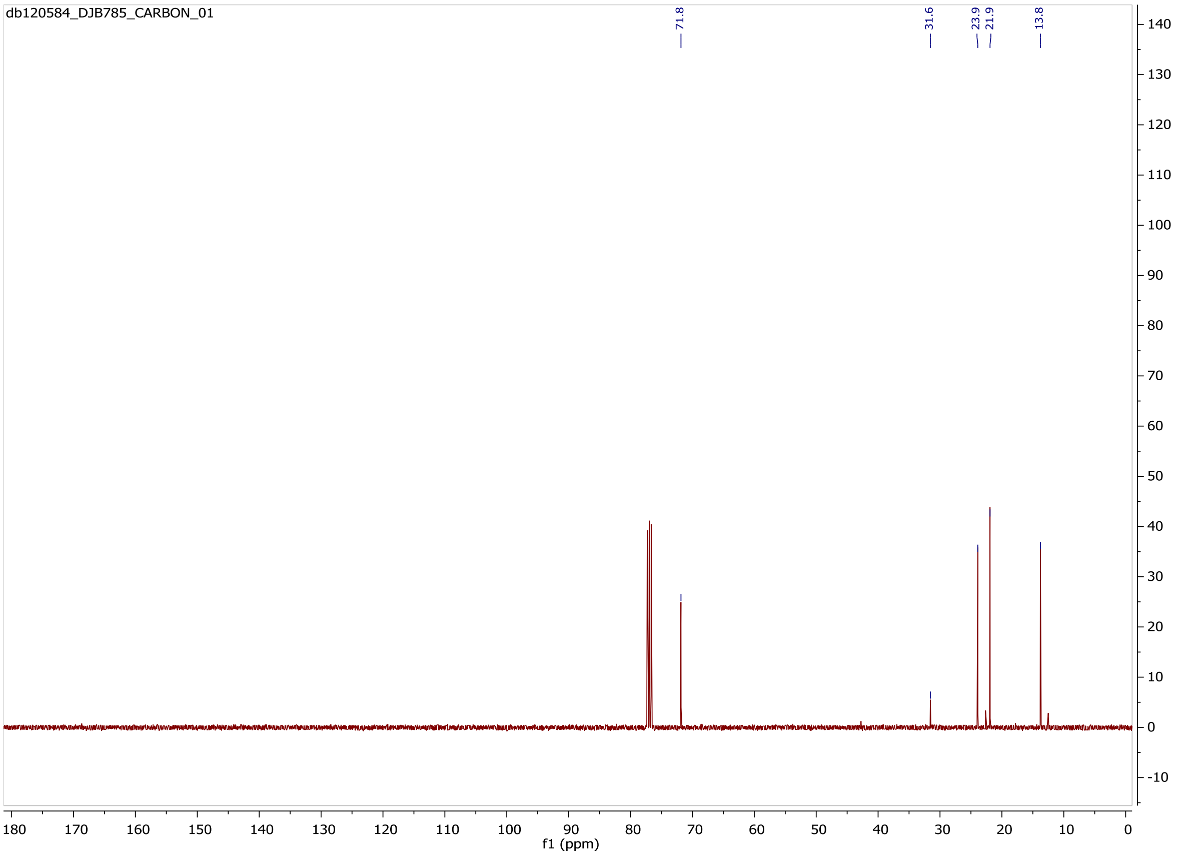

4,4,5,5-Tetramethyl-2-(3-methyl-2-phenylbutan-2-yl)-1,3,2-dioxaborolane (11)

^{1}H NMR (400 MHz, $CDCl_3$)

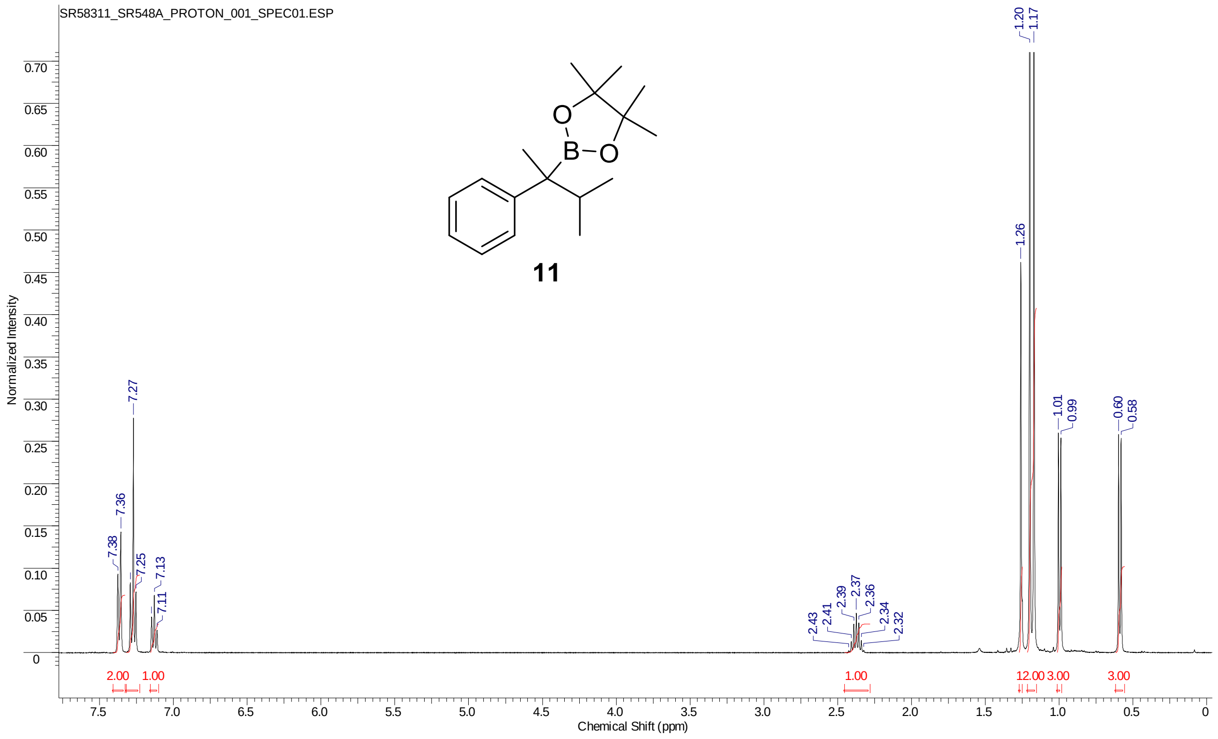

^{13}C NMR (126 MHz, $CDCl_3$)

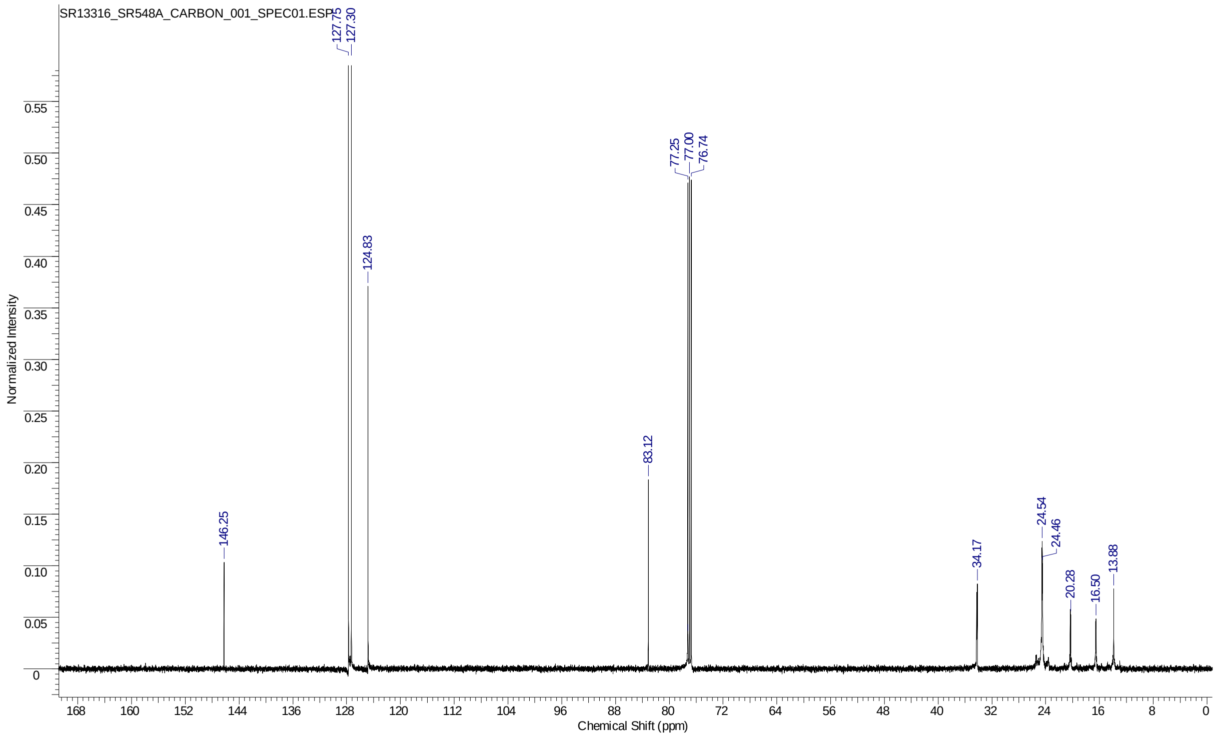

4,4,5,5-Tetramethyl-2-(2-methyl-3-phenylpentan-3-yl)-1,3,2-dioxaborolane (15)

^{1}H NMR (400 MHz, $CDCl_3$)

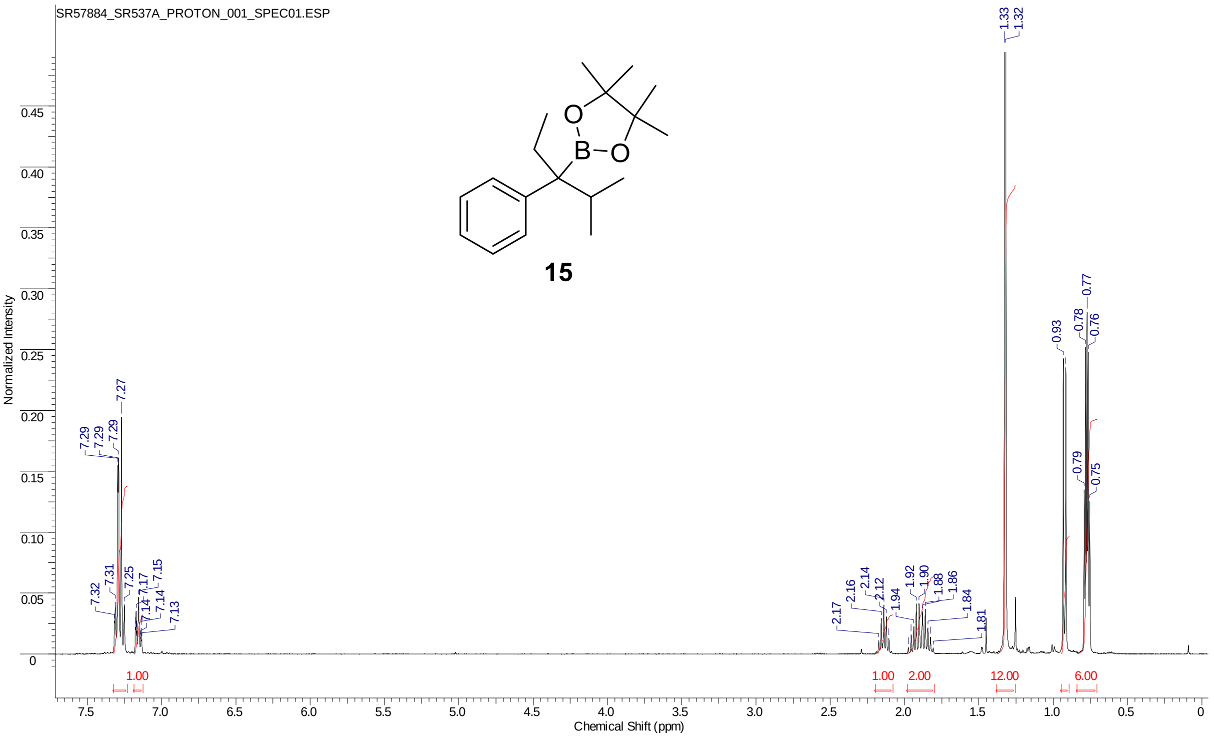

^{13}C NMR (101 MHz, $CDCl_3$)

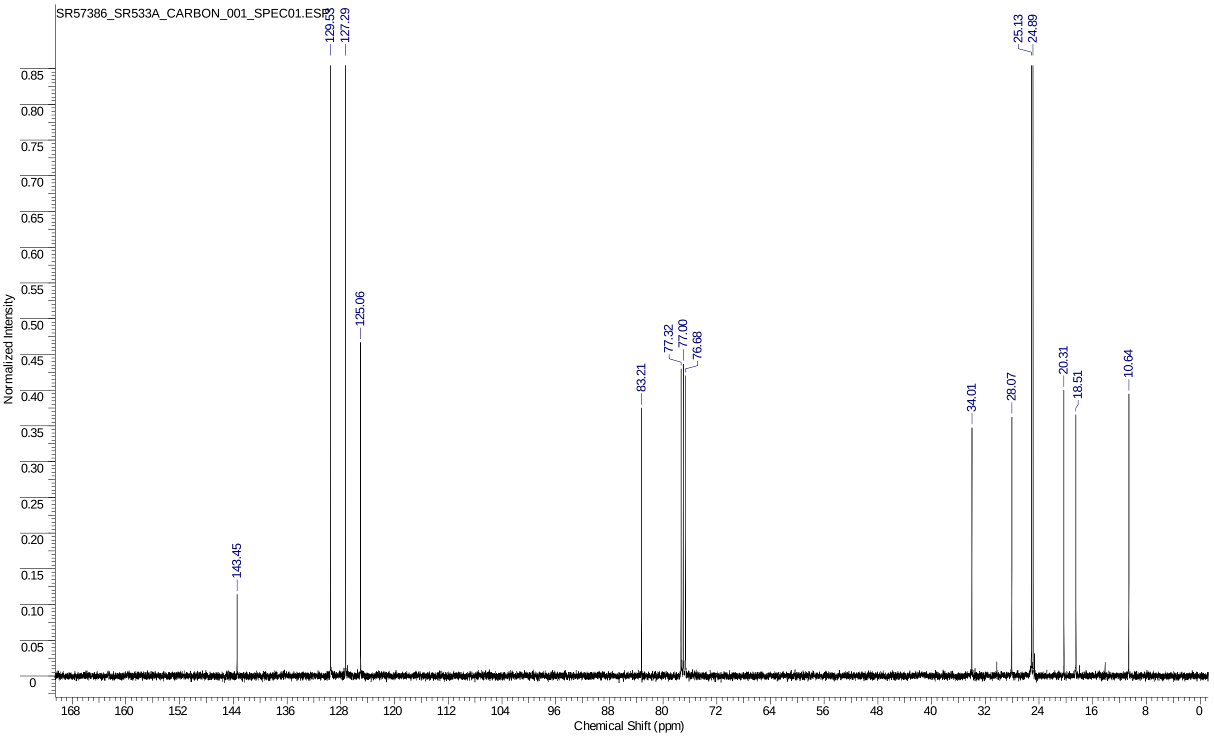

2-(2,3-Diphenylbutan-2-yl)-4,4,5,5-tetramethyl-1,3,2-dioxaborolane (12)

^{1}H NMR (500 MHz, $CDCl_3$)

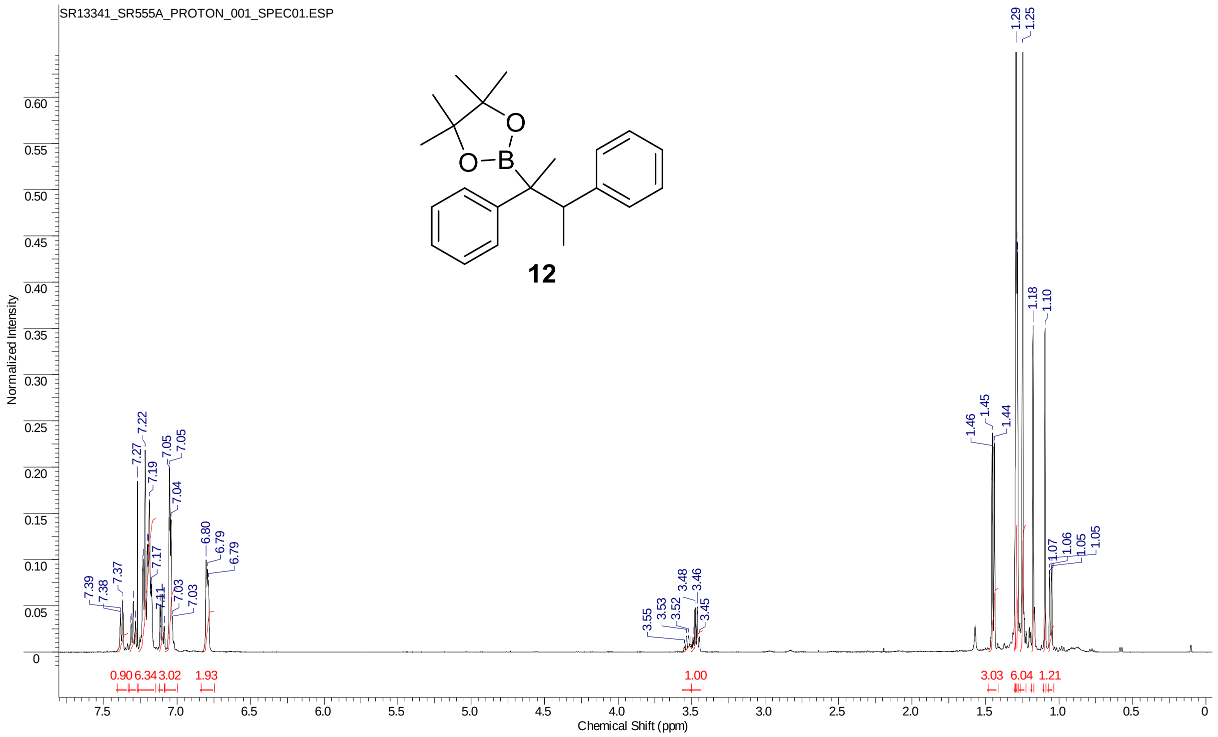

^{13}C NMR (126 MHz, $CDCl_3$)

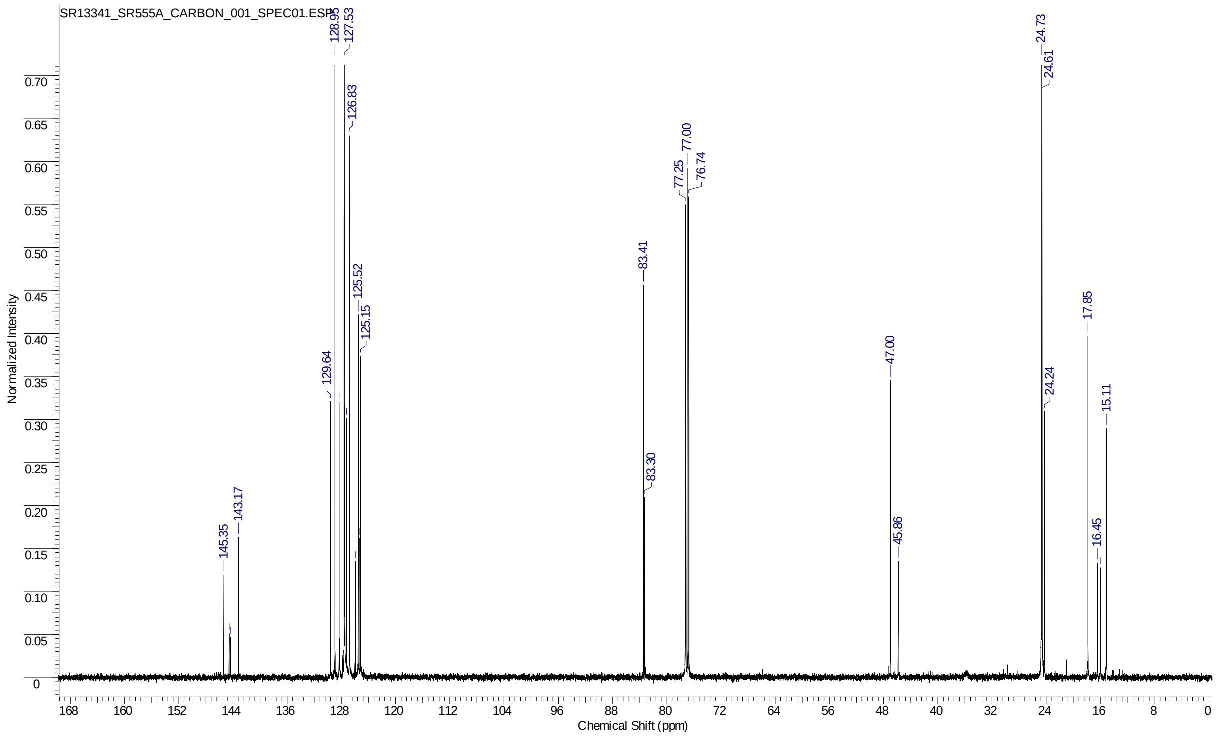

2-(2,3-Diphenylpentan-3-yl)-4,4,5,5-tetramethyl-1,3,2-dioxaborolane (16)

^{1}H NMR (300 MHz, $CDCl_3$)

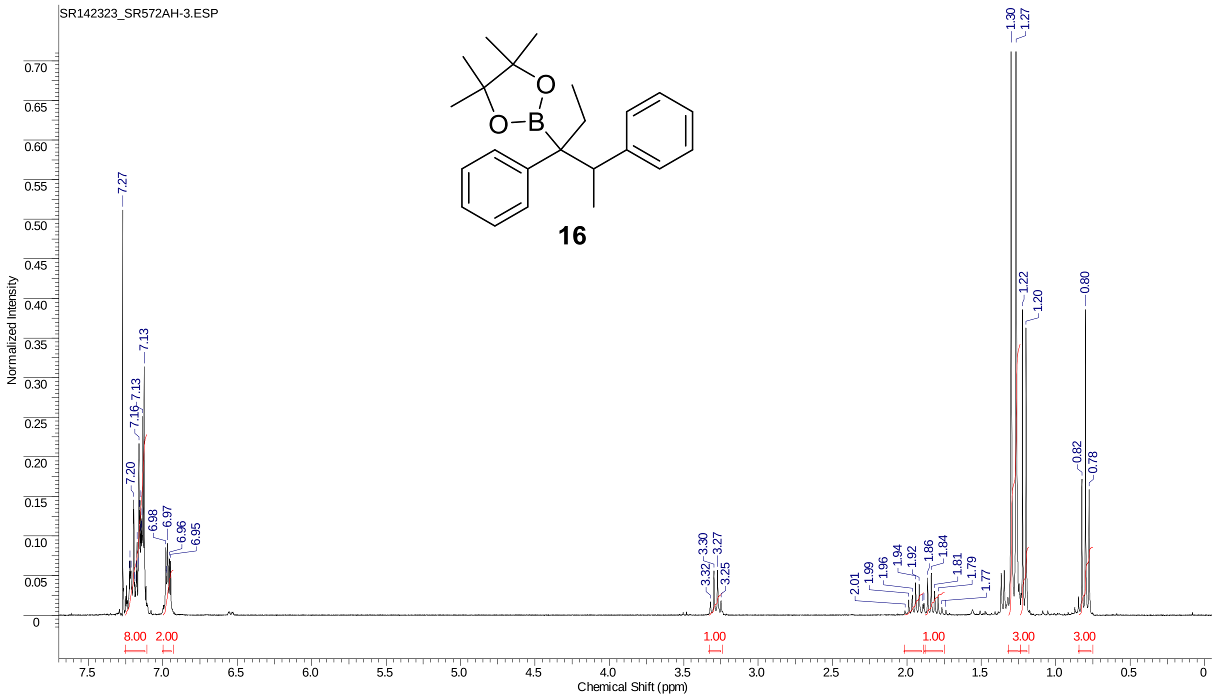

^{13}C NMR (126 MHz, $CDCl_3$)

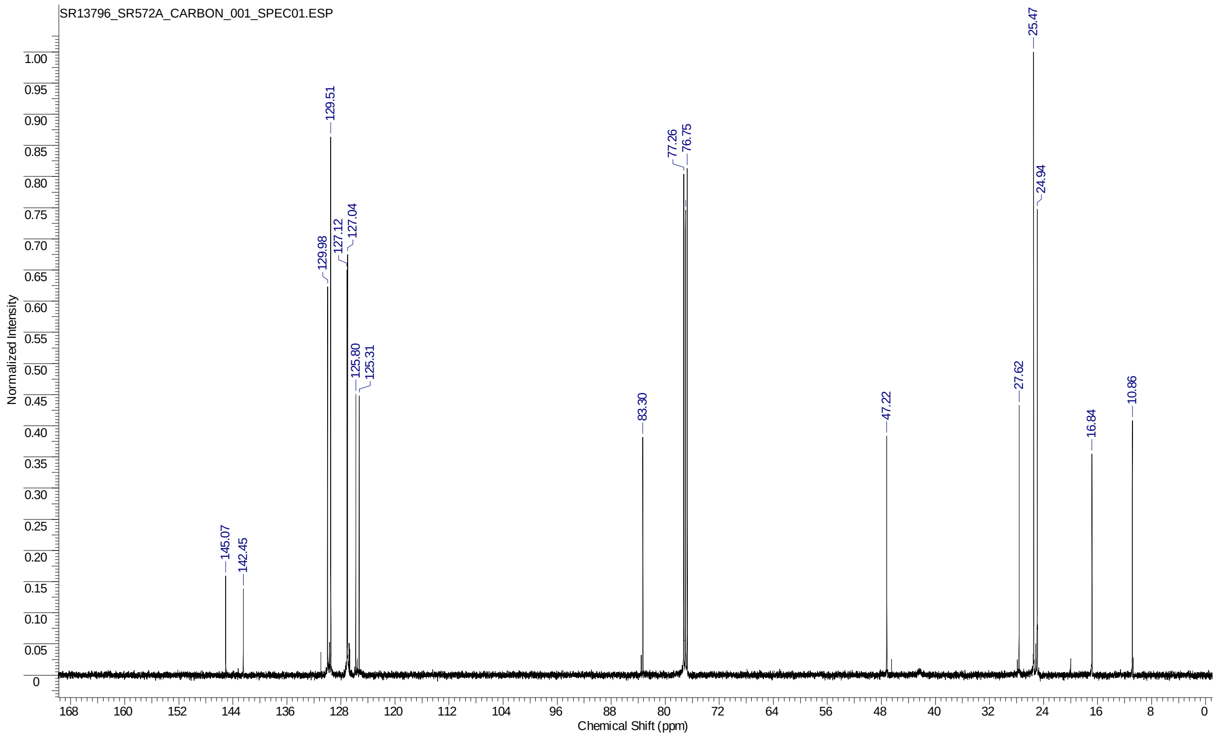

2-(2,3-Diphenylpentan-2-yl)-4,4,5,5-tetramethyl-1,3,2-dioxaborolane (13)

^{1}H NMR (400 MHz, $CDCl_3$)

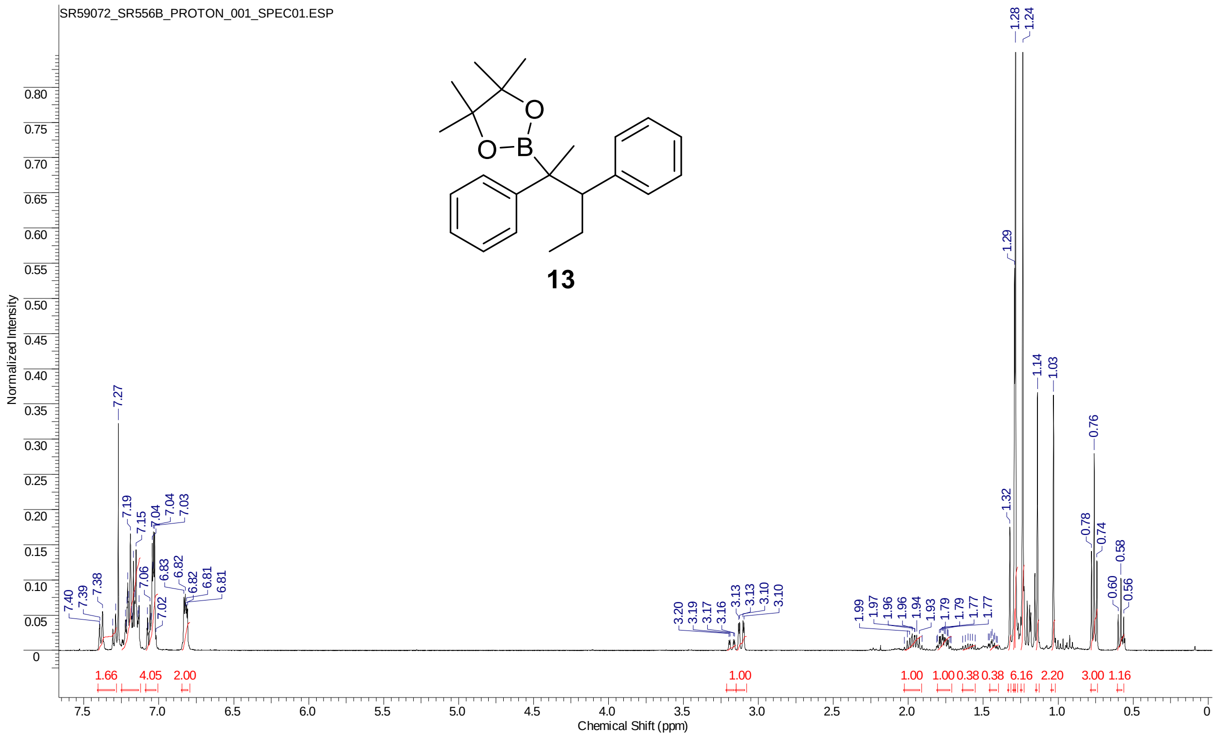

^{13}C NMR (126 MHz, $CDCl_3$)

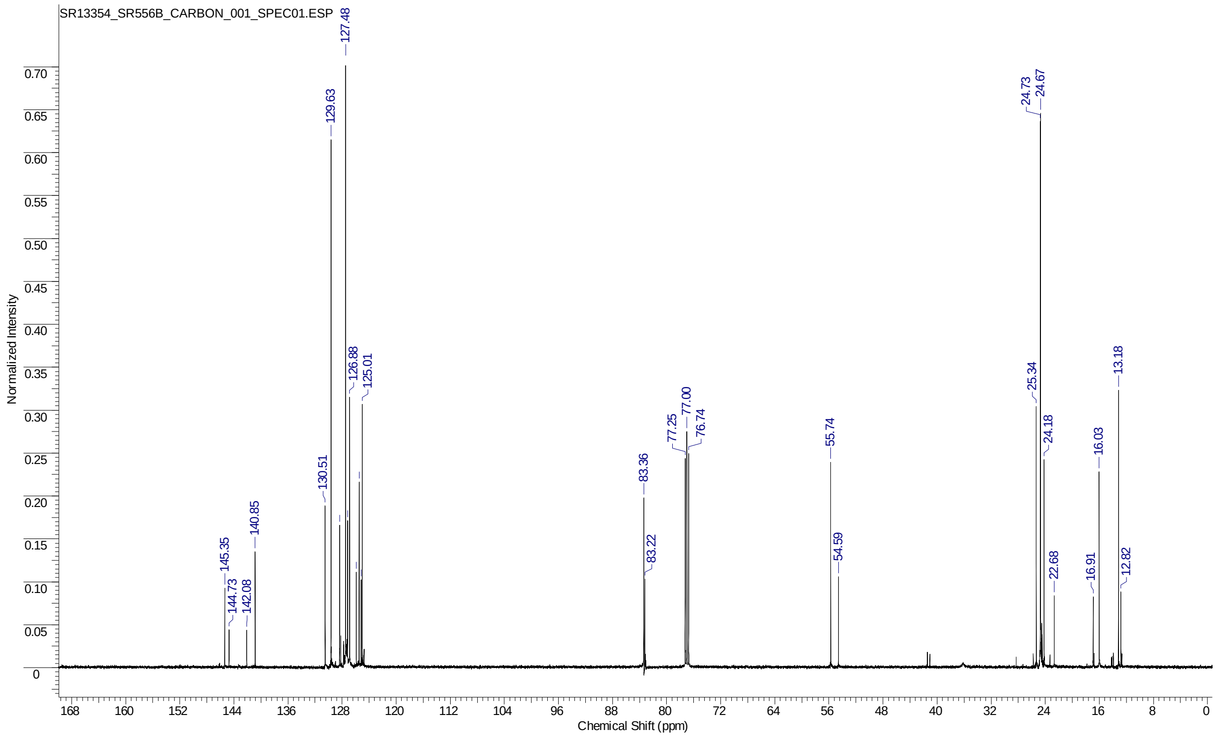

2-(2,3-diphenylpentan-2-yl)-5,5-dimethyl-1,3,2-dioxaborinane (21)

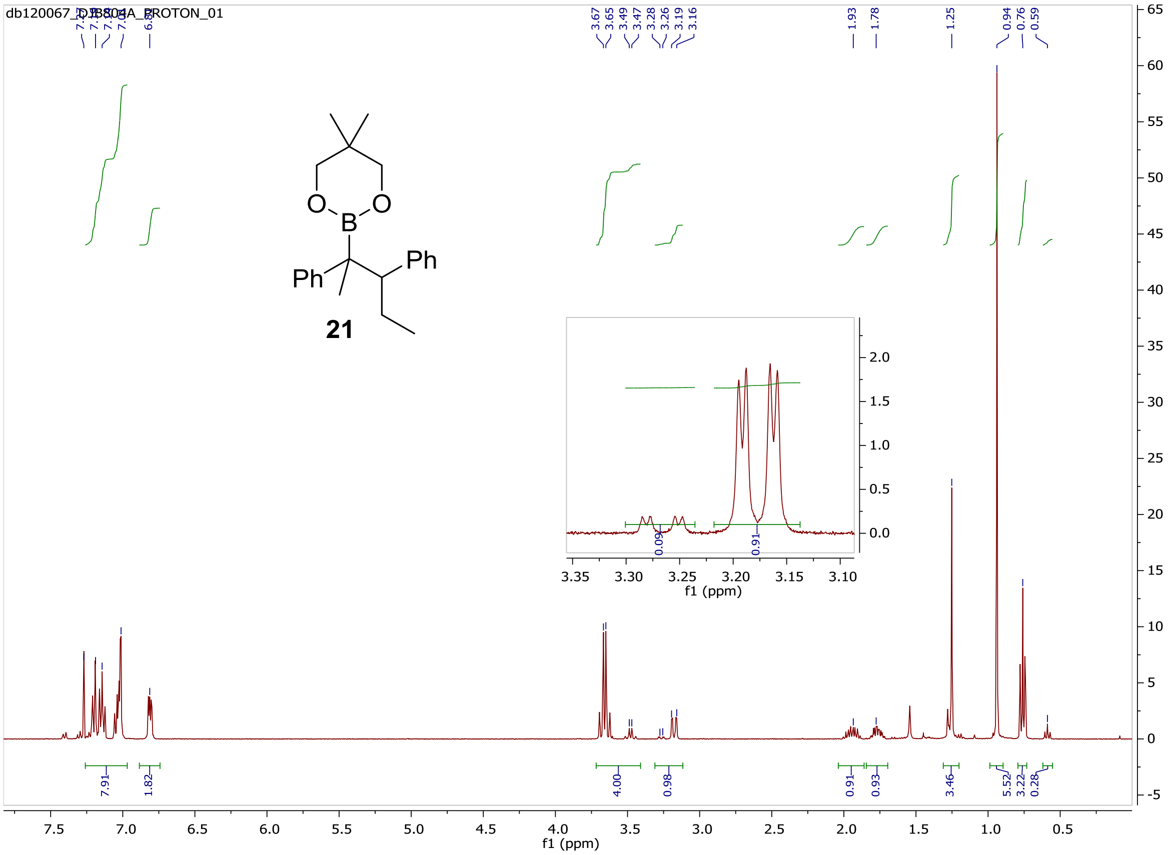

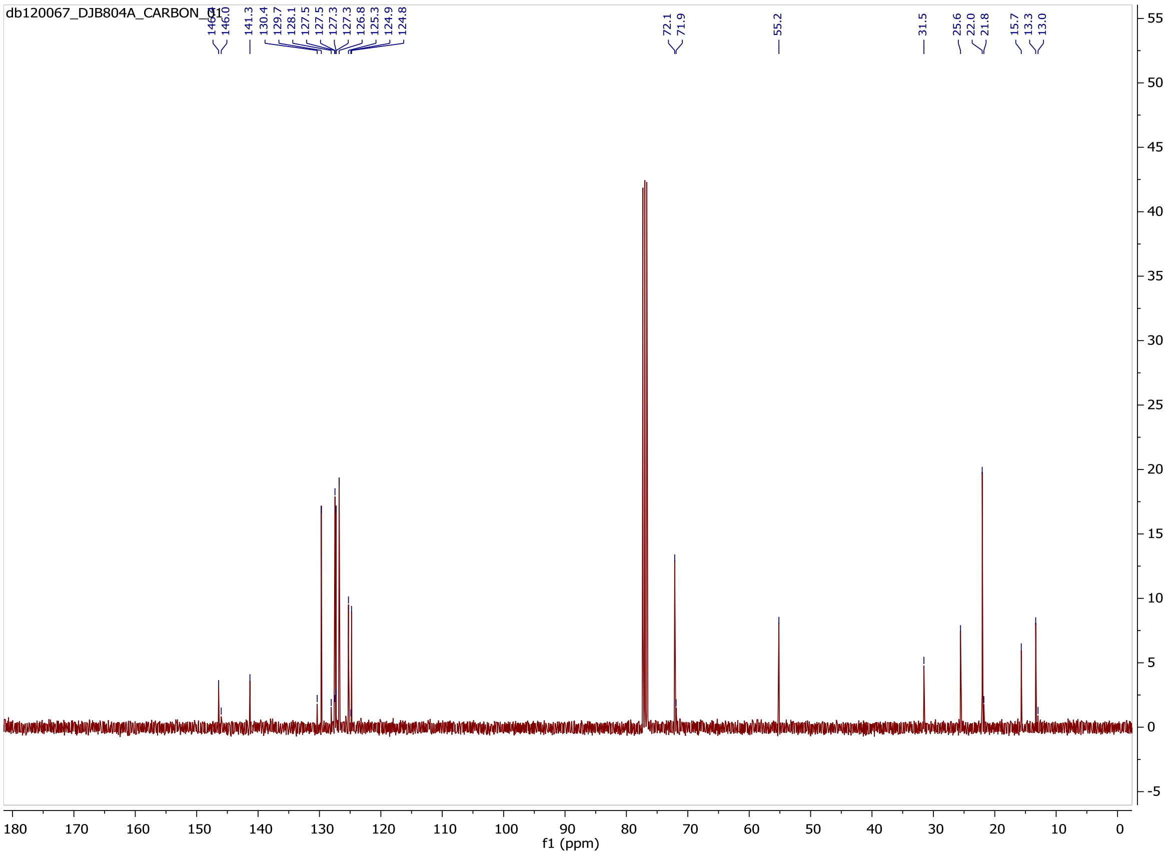

2-((2S,3R)-2,3-diphenylpentan-2-yl)-5,5-dimethyl-1,3,2-dioxaborinane ((2S,3R)-21)

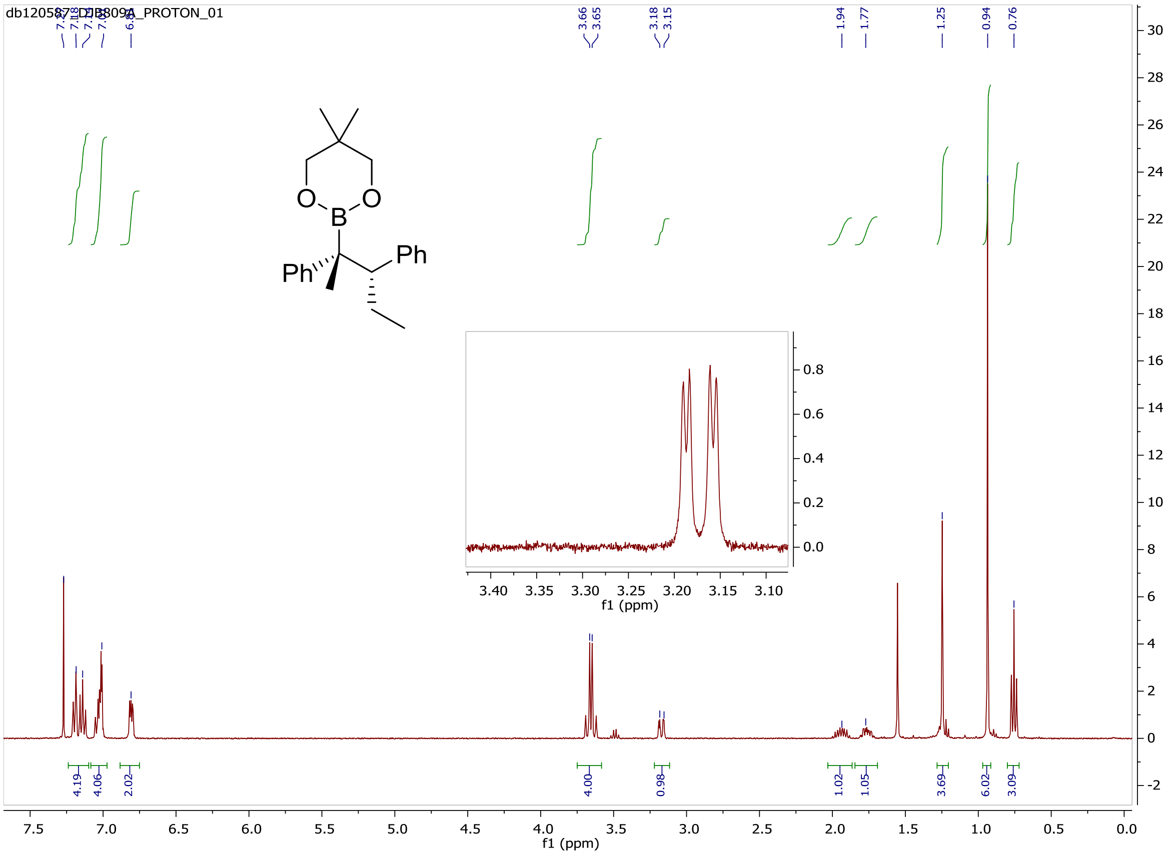

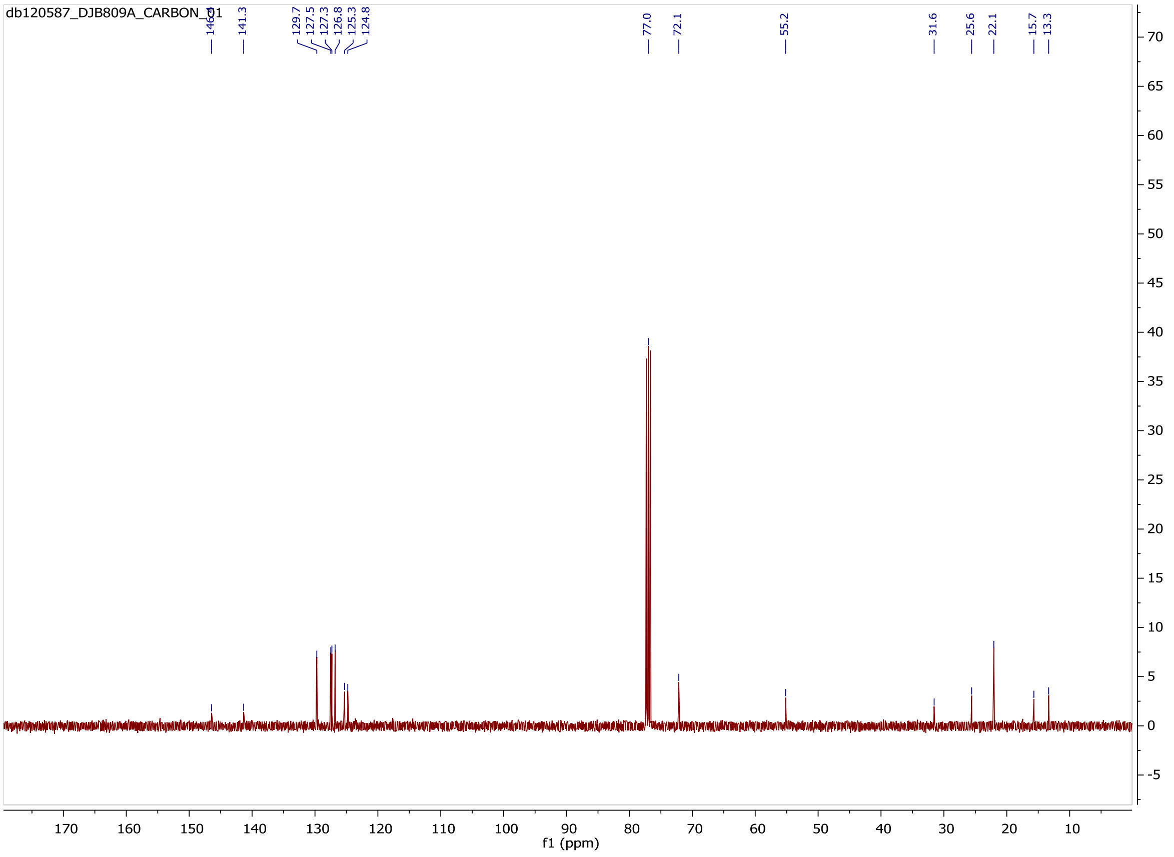

2-((2R,3R)-2,3-diphenylpentan-2-yl)-5,5-dimethyl-1,3,2-dioxaborinane ((2R,3R)-21)

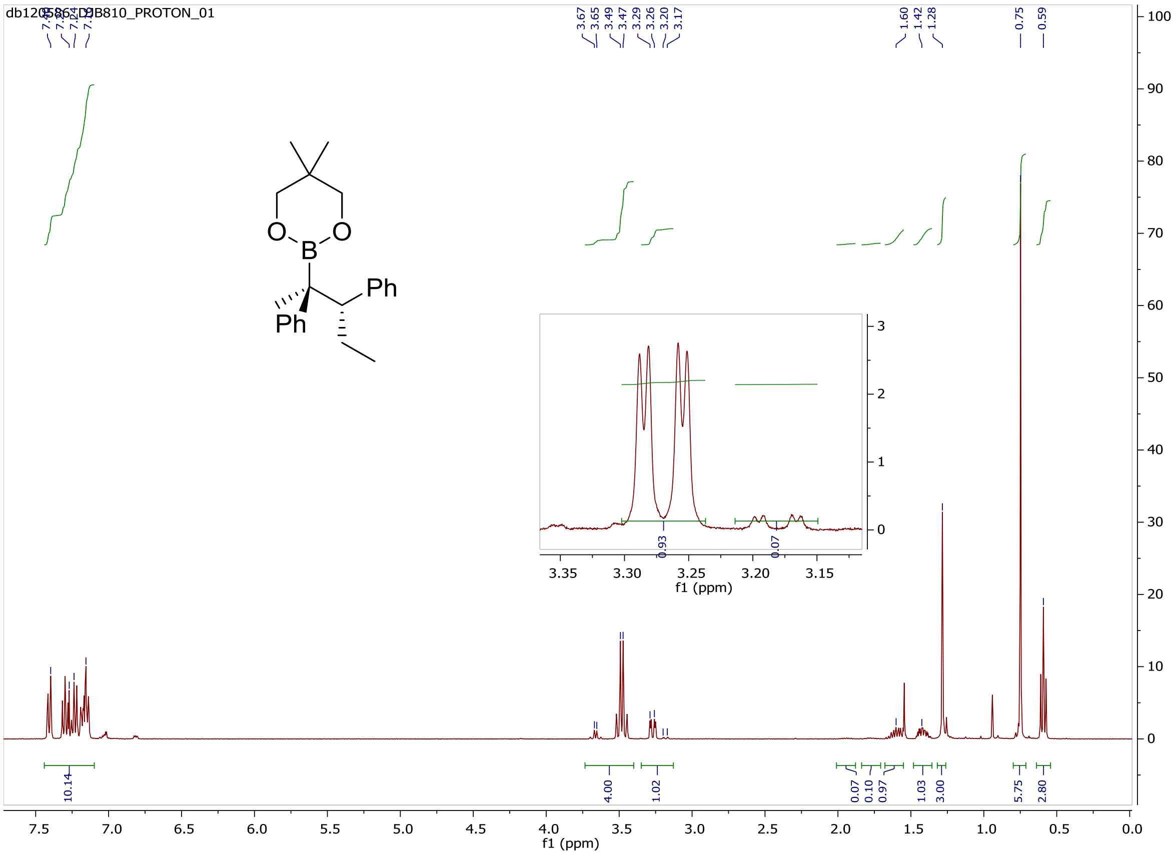

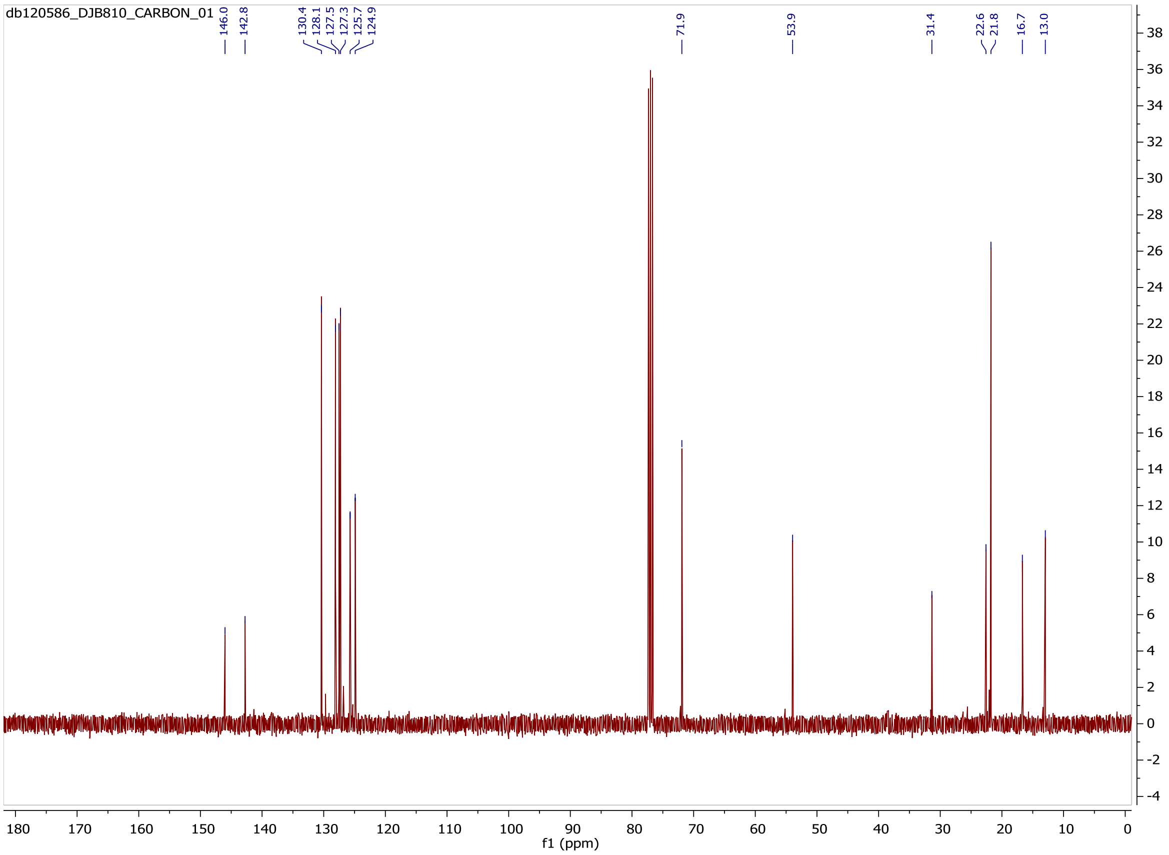

2-(3,4-diphenylhexan-3-yl)-5,5-dimethyl-1,3,2-dioxaborinane 22

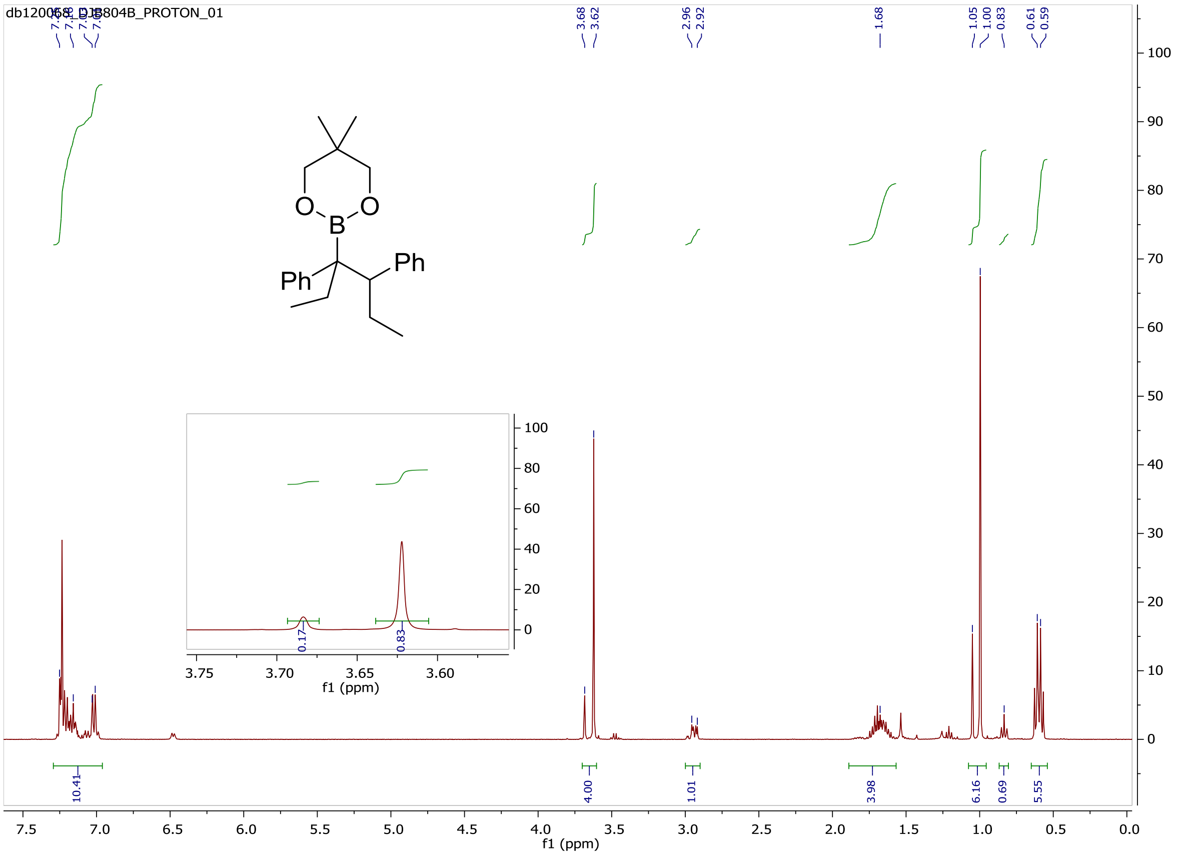

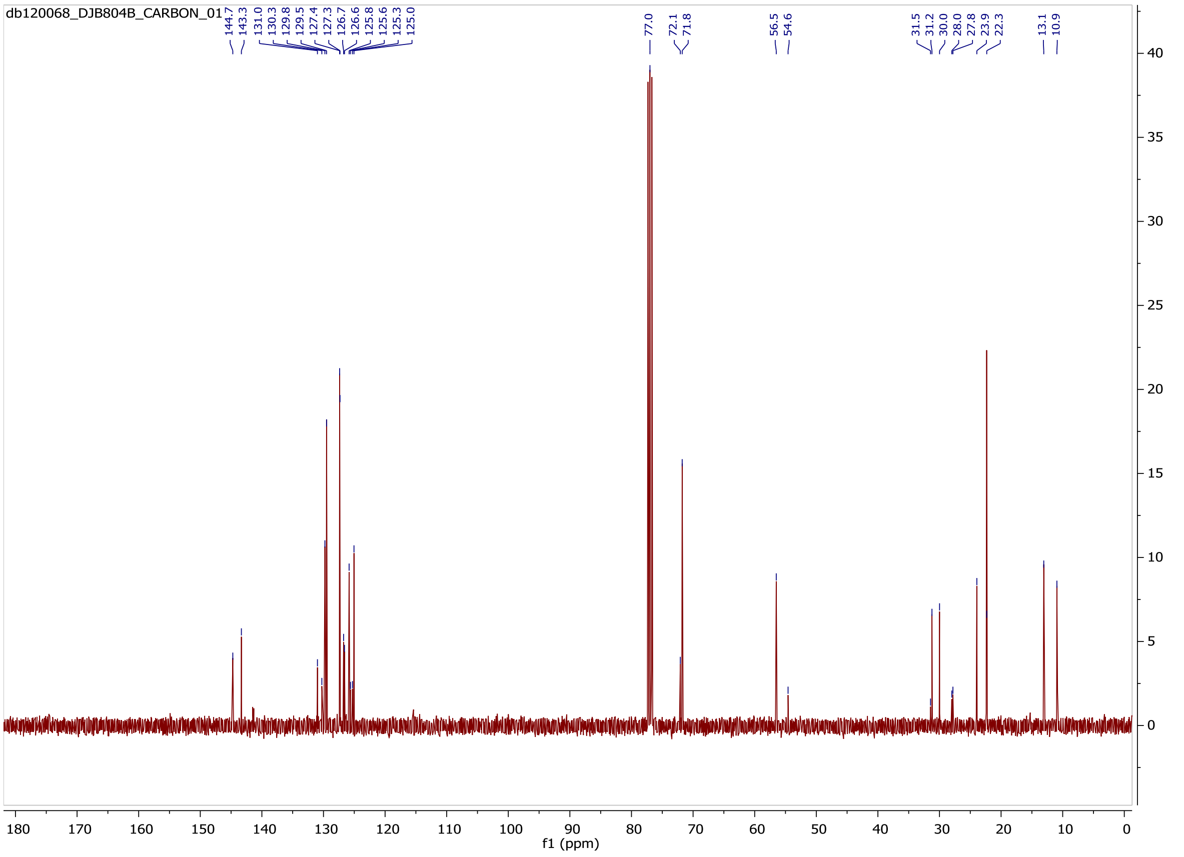

2-((3S,4R)-3,4-diphenylhexan-3-yl)-5,5-dimethyl-1,3,2-dioxaborinane ((3S,4R)-22)

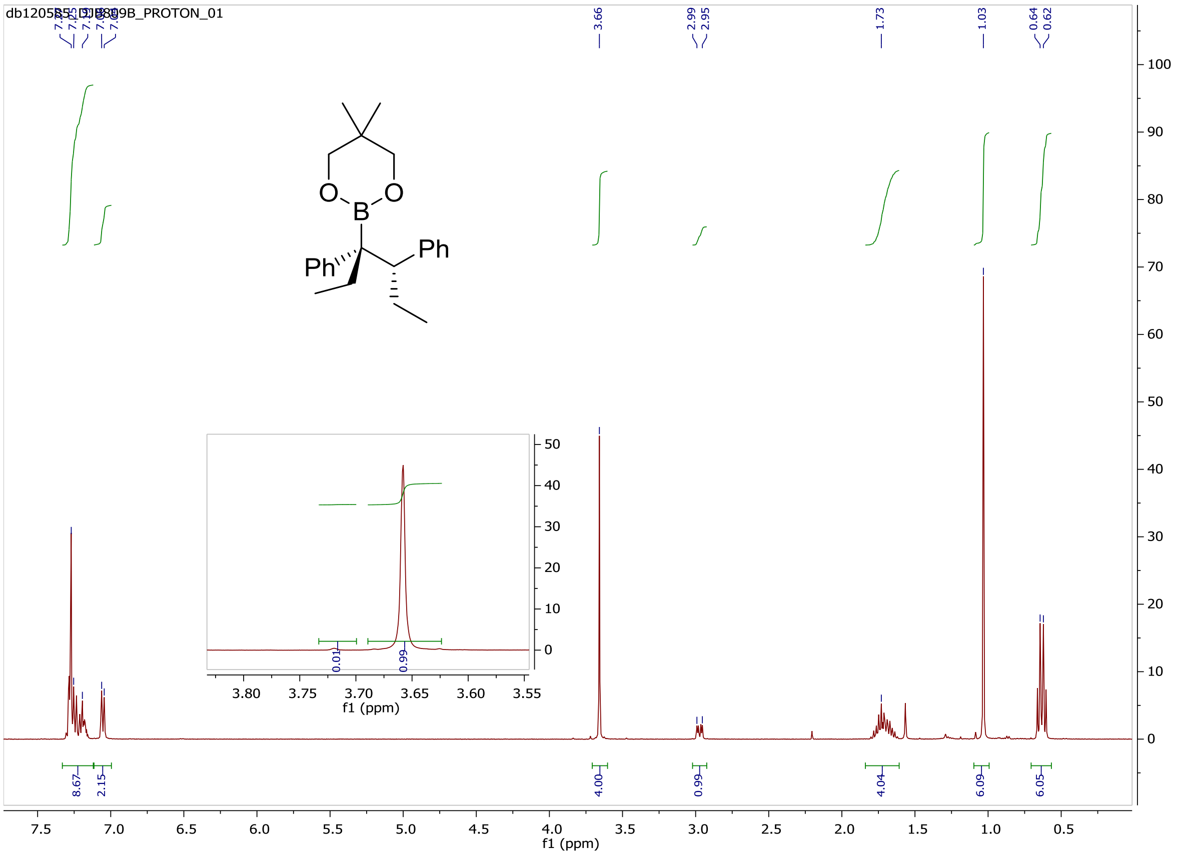

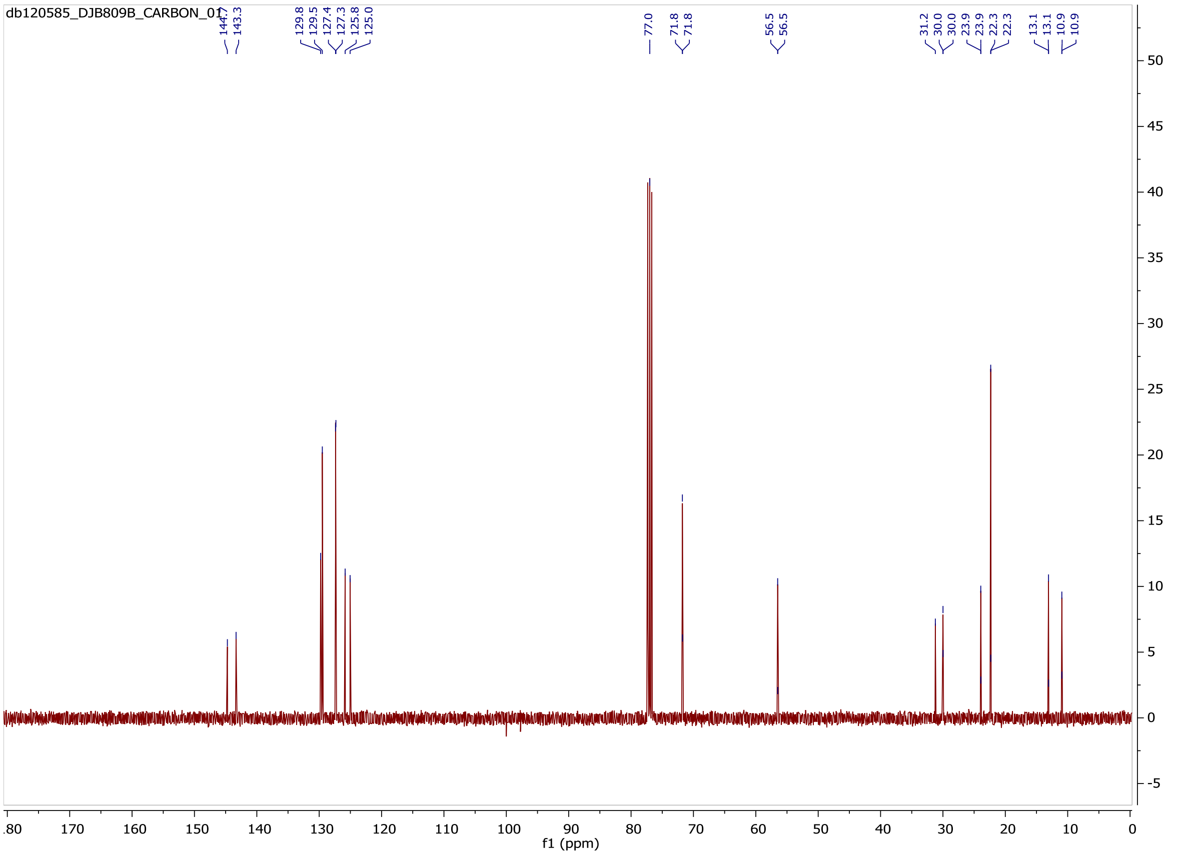

2-((3R,4R)-3,4-diphenylhexan-3-yl)-5,5-dimethyl-1,3,2-dioxaborinane ((3R,4R)-22)

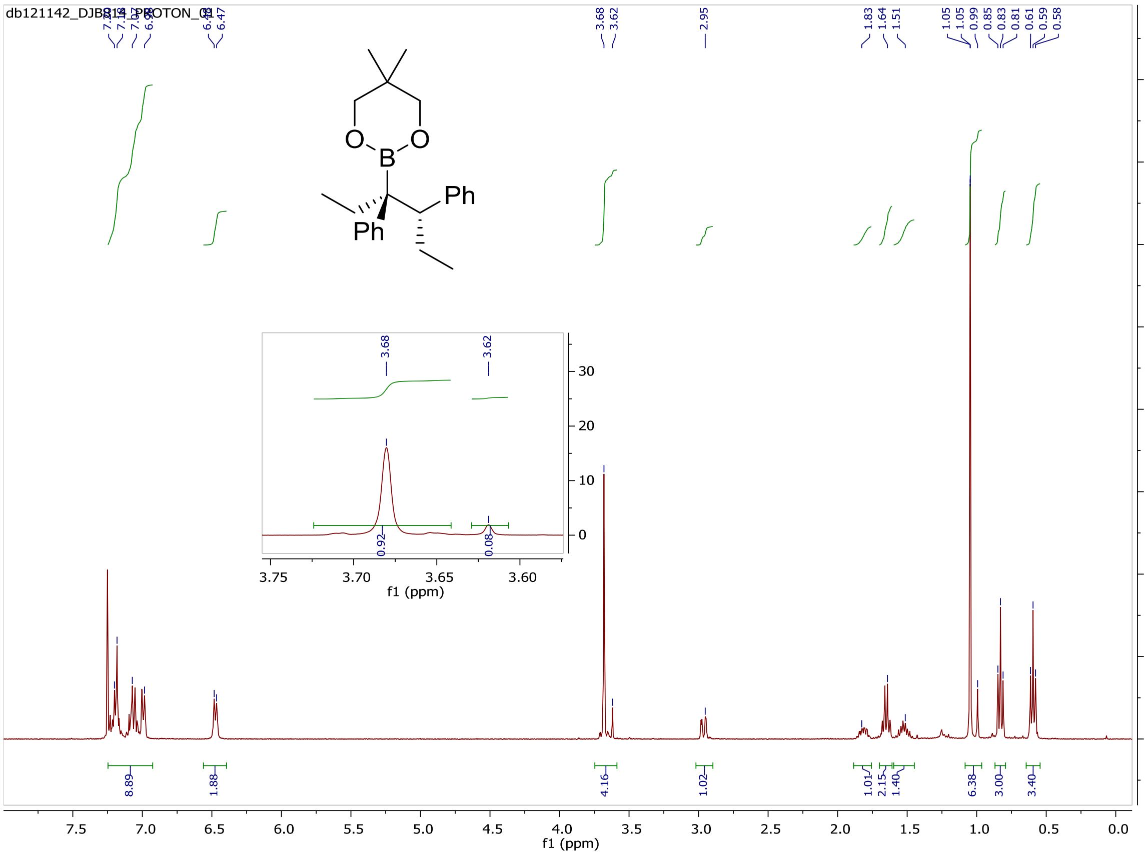

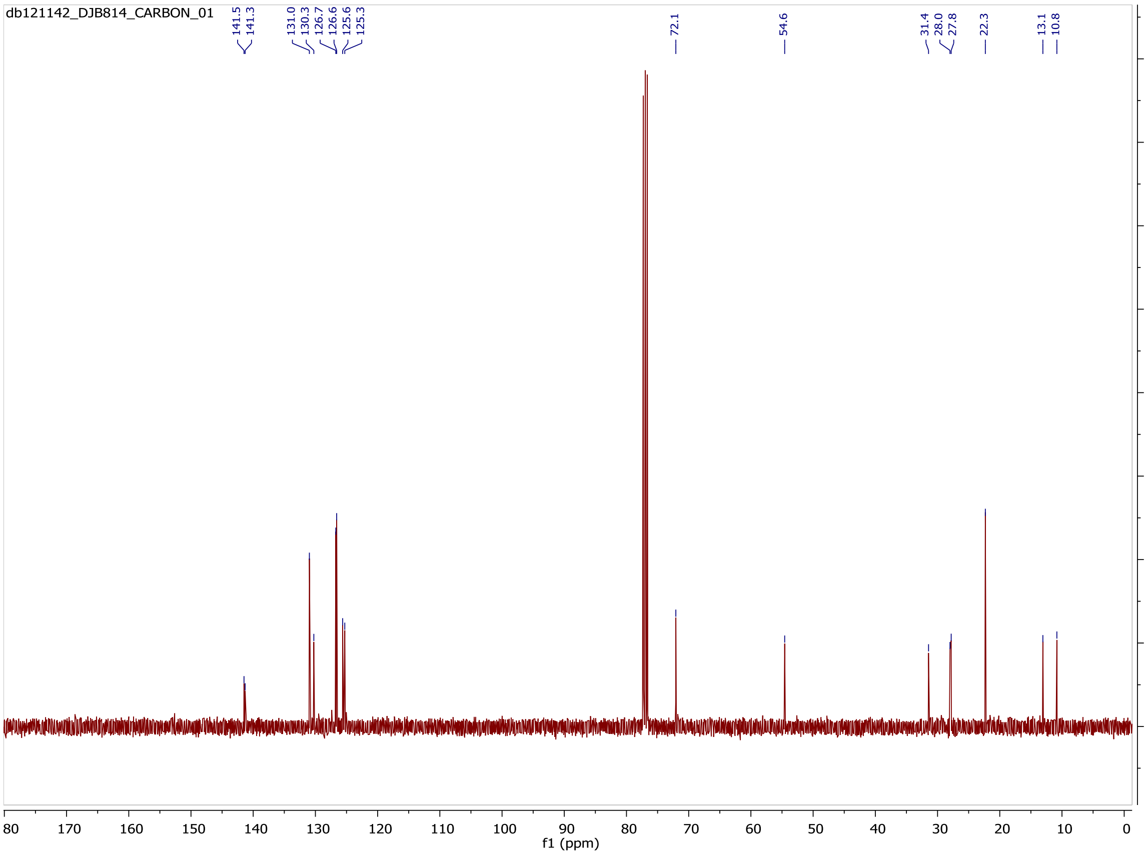

2-(3-Ethyl-2-phenylpentan-2-yl)-4,4,5,5-tetramethyl-1,3,2-dioxaborolane (14)

^{1}H NMR (500 MHz, $CDCl_3$)

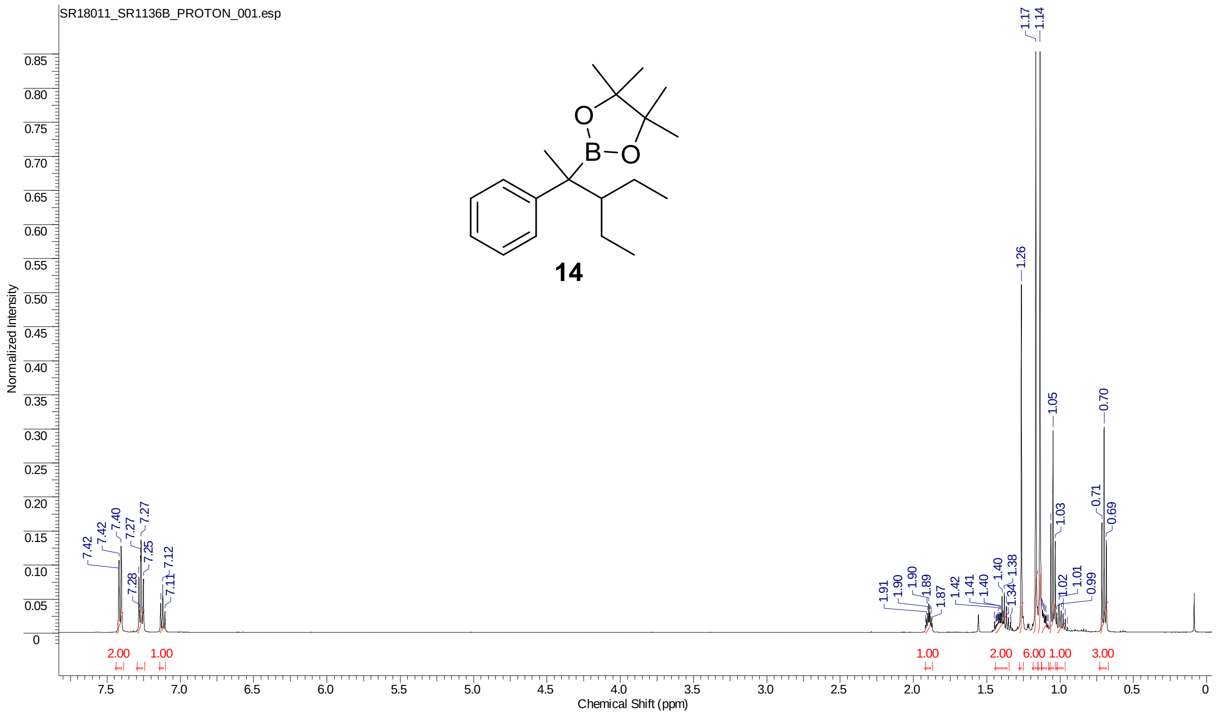

^{13}C NMR (126 MHz, $CDCl_3$)

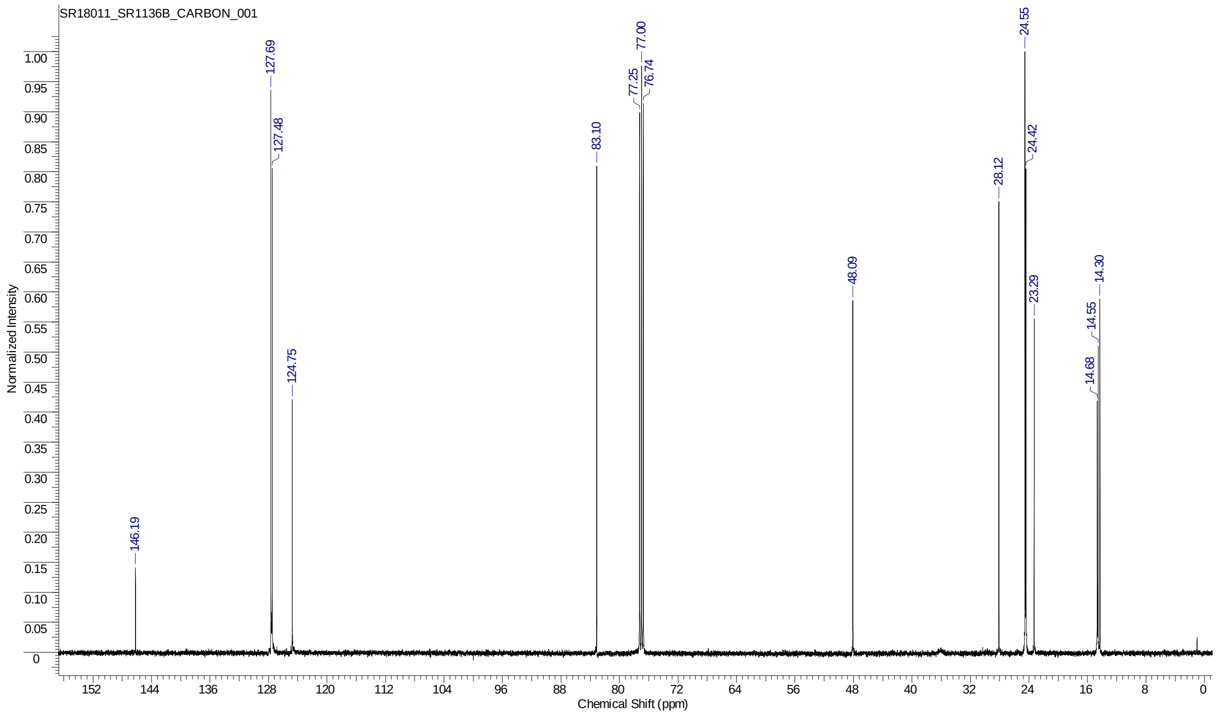

2-(3-ethyl-2-phenylpentan-2-yl)-5,5-dimethyl-1,3,2-dioxaborinane (23)

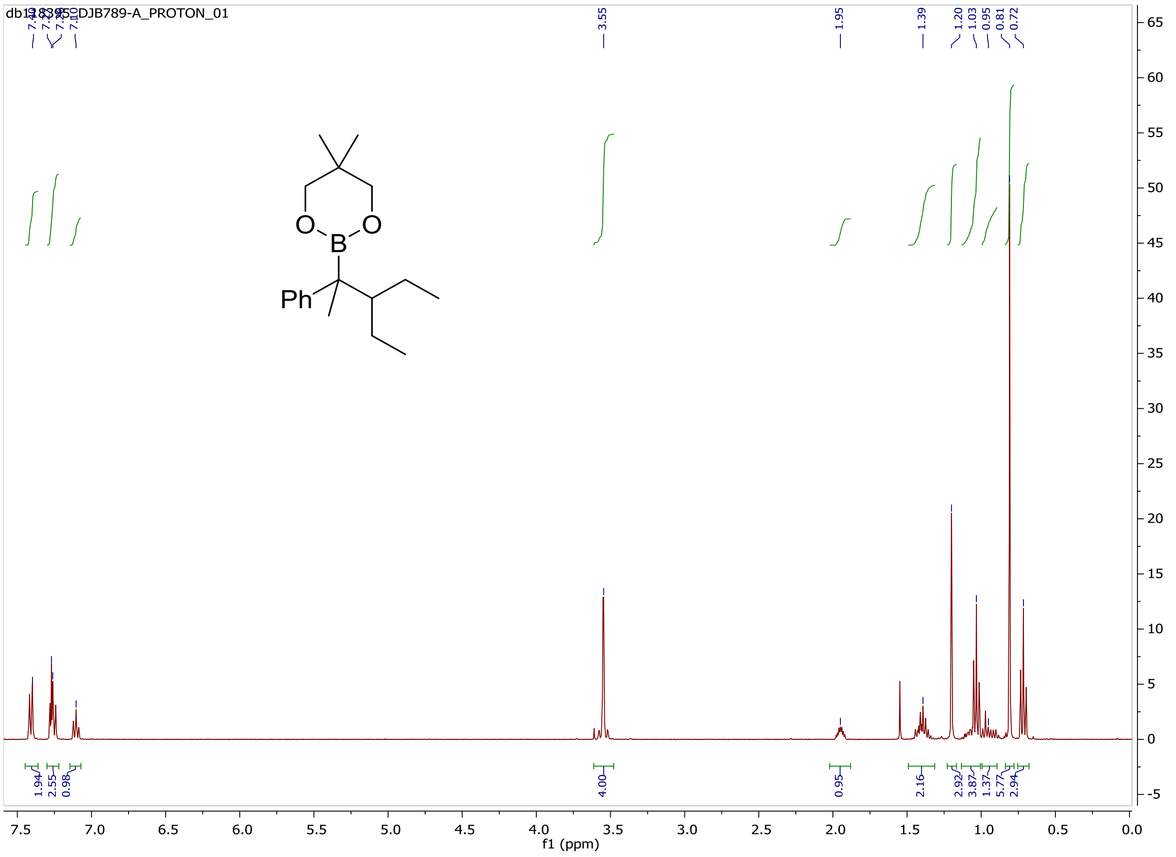

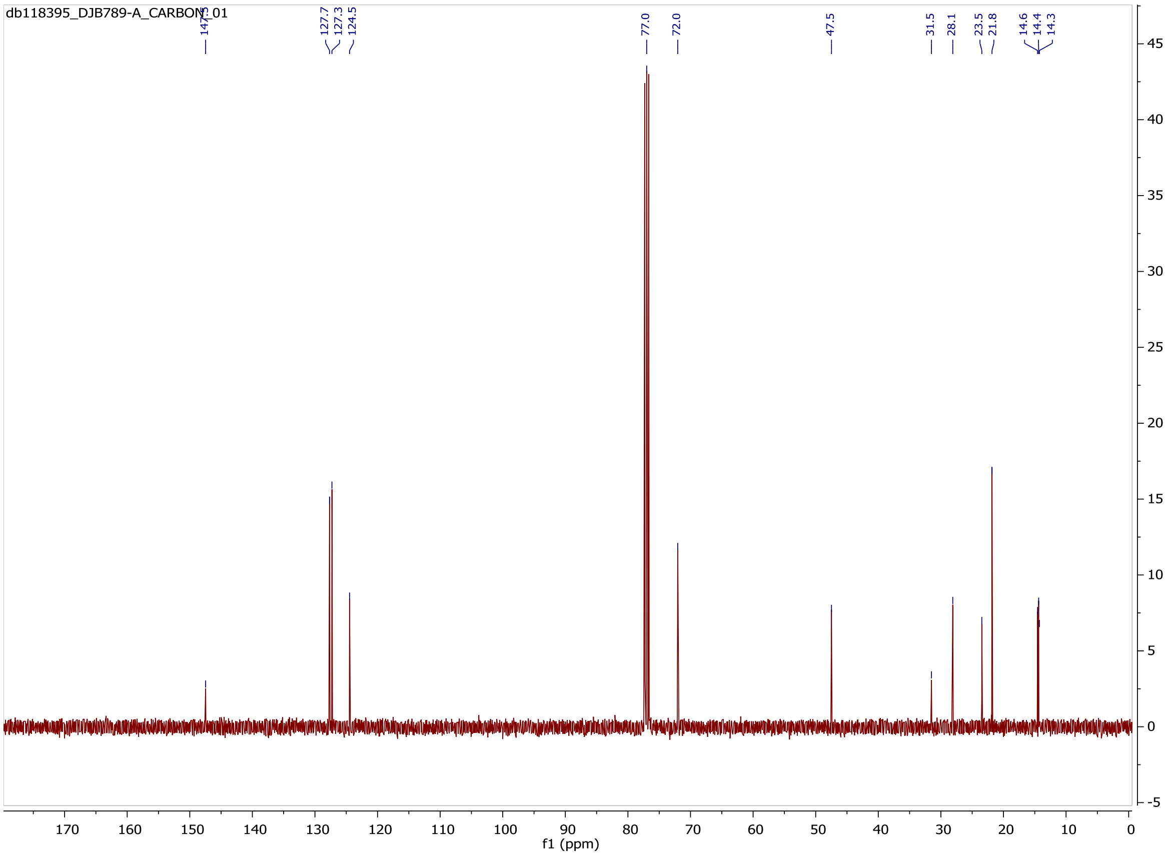

(*S*)-2-(3-ethyl-2-phenylpentan-2-yl)-5,5-dimethyl-1,3,2-dioxaborinane (*S*)-24

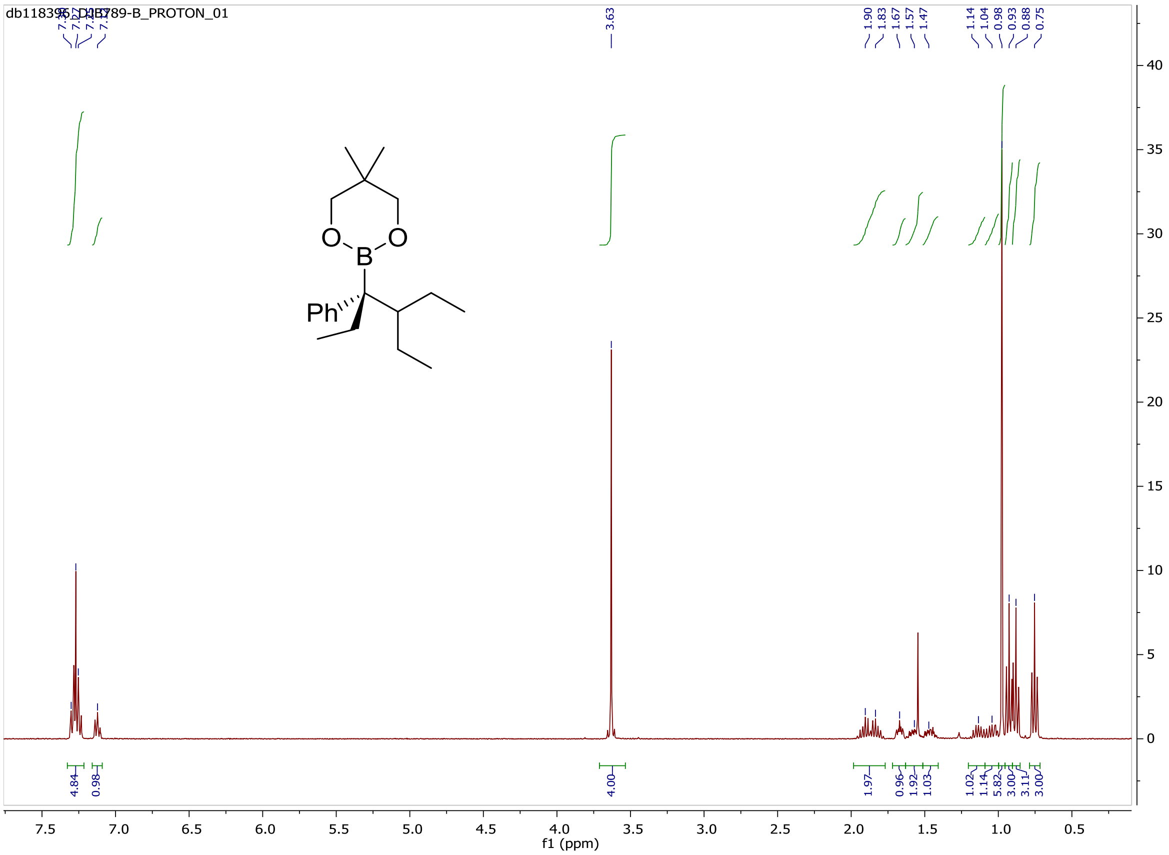

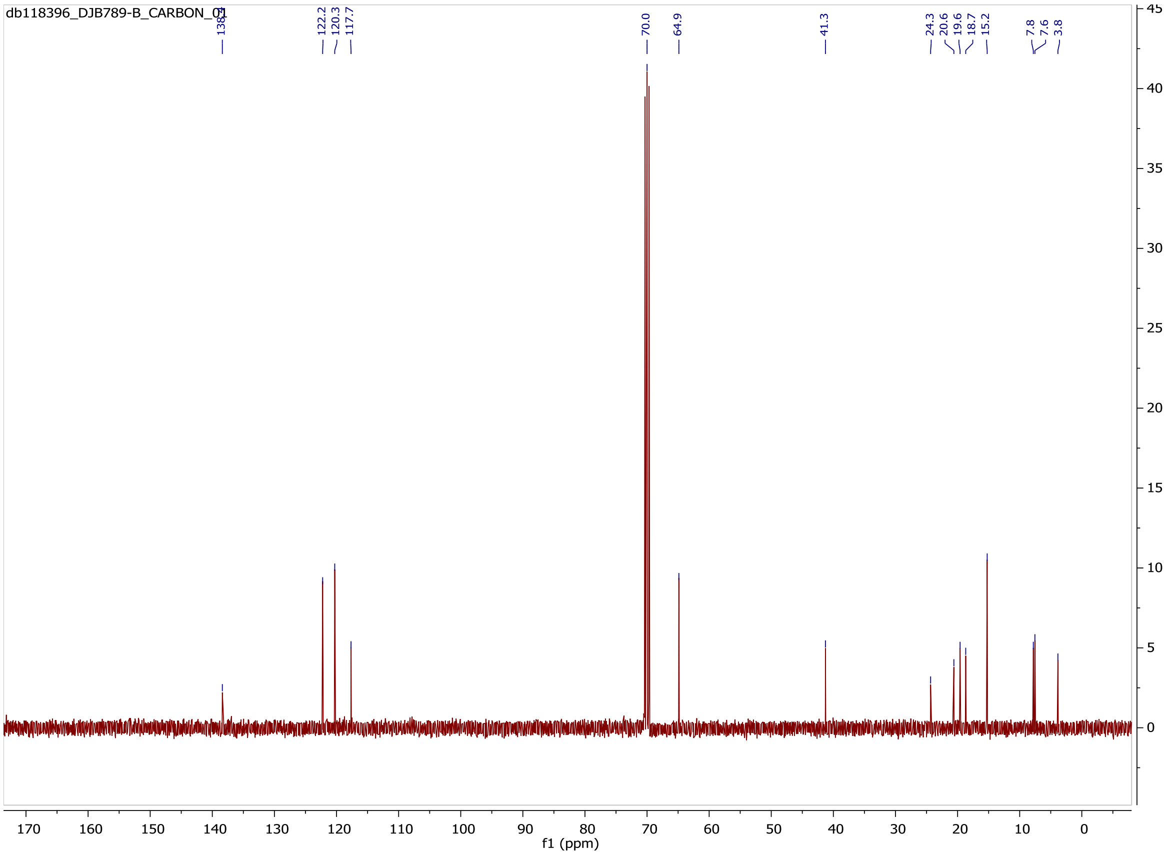

(2*S*,3*R*)-2,3-Diphenylpentan-3-ol ((2*S*,3*R*)-39)

^{1}H NMR (400 MHz, $CDCl_3$)

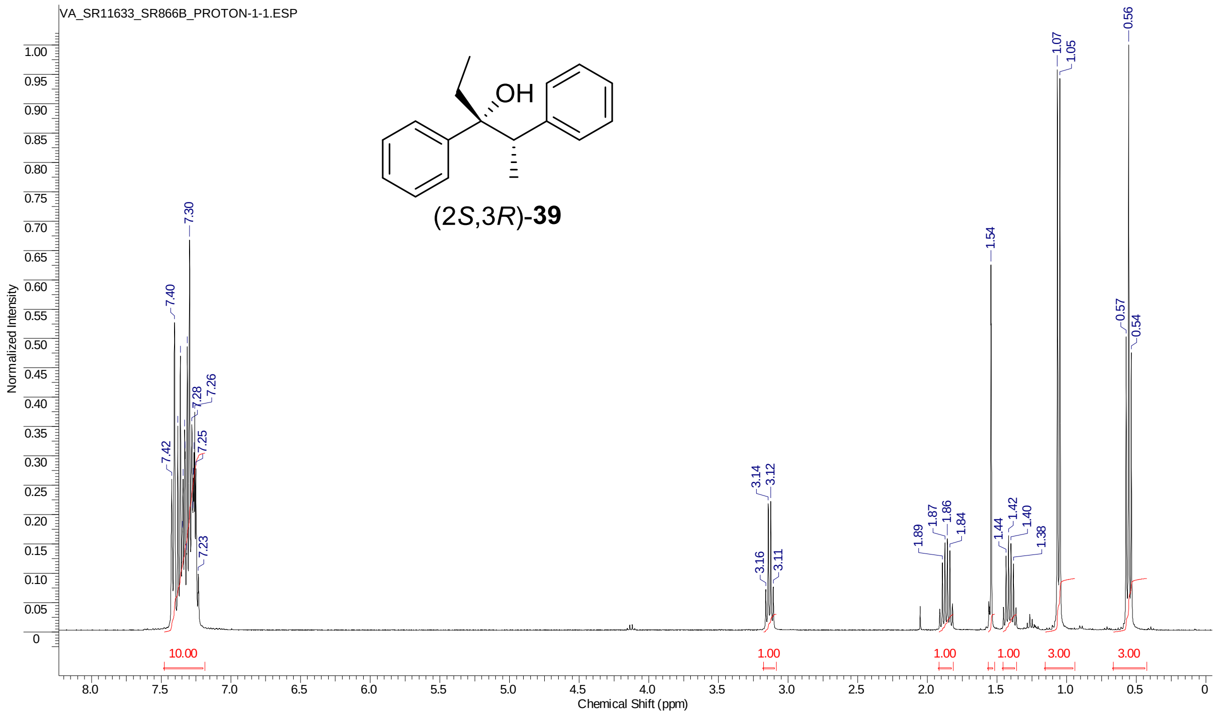

^{13}C NMR (101 MHz, $CDCl_3$)

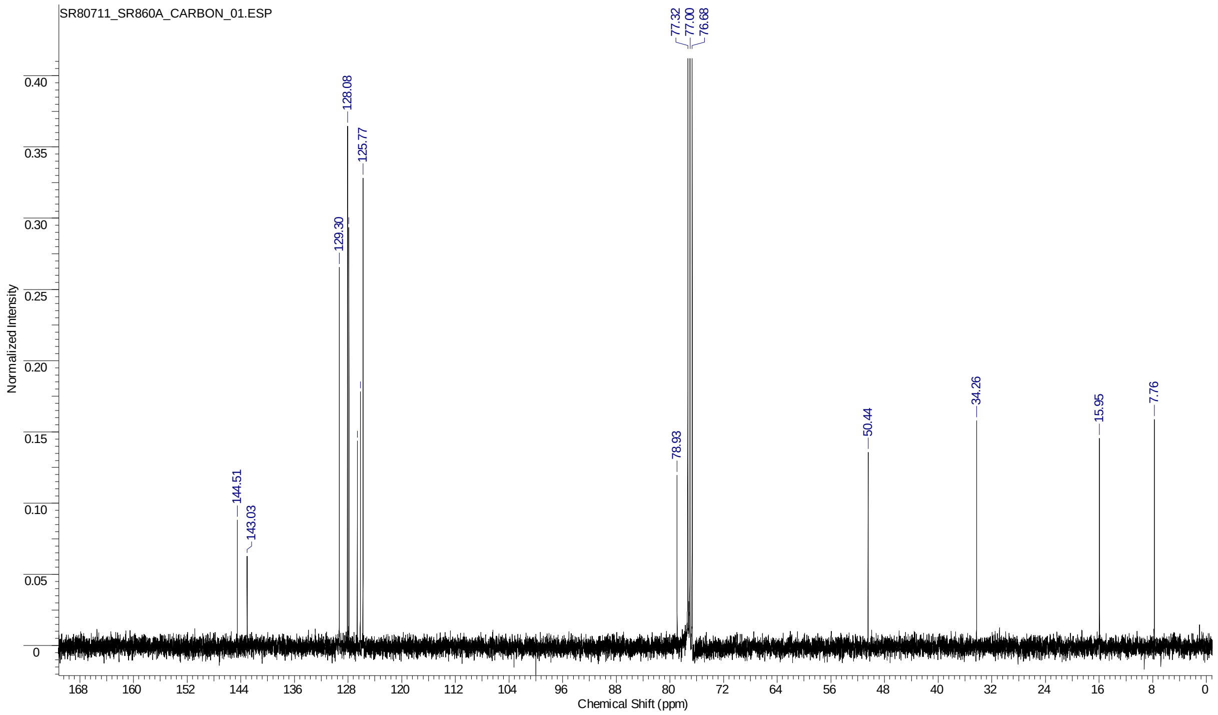

(2*R*,3*R*)-2,3-Diphenylpentan-3-ol ((2*R*,3*R*)-39)

^{1}H NMR (400 MHz, $CDCl_3$)

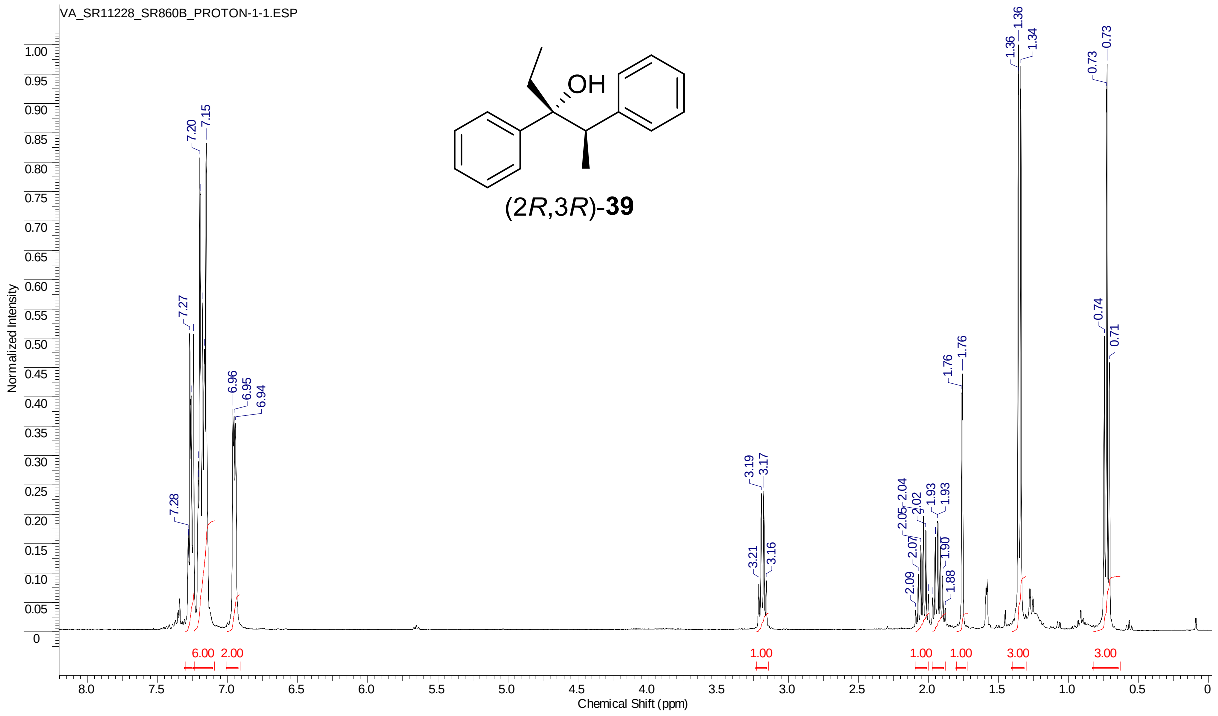

^{13}C NMR (101 MHz, $CDCl_3$)

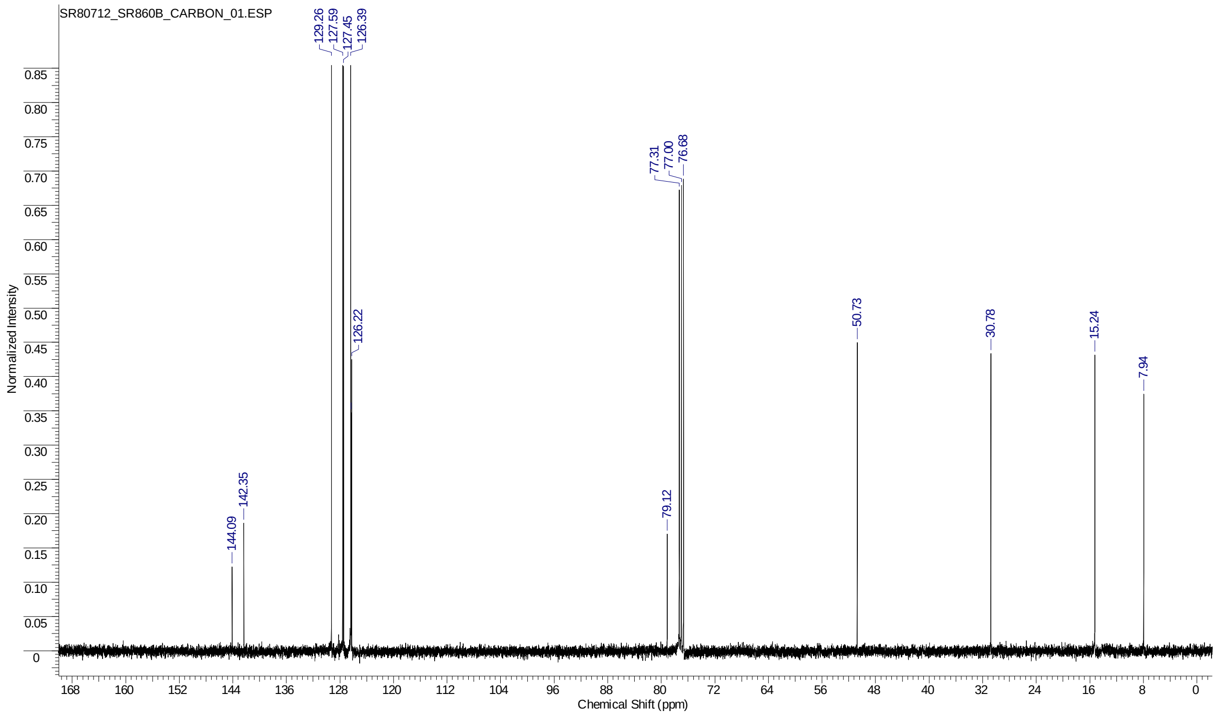

2-((2*S*,3*R*)-3-(3-Fluoro-4-methoxyphenyl)-2-(4-methoxyphenyl)pentan-2-yl)-4,4,5,5-tetramethyl-1,3,2-dioxaborolane (33)

^{1}H NMR (500 MHz, $CDCl_3$)

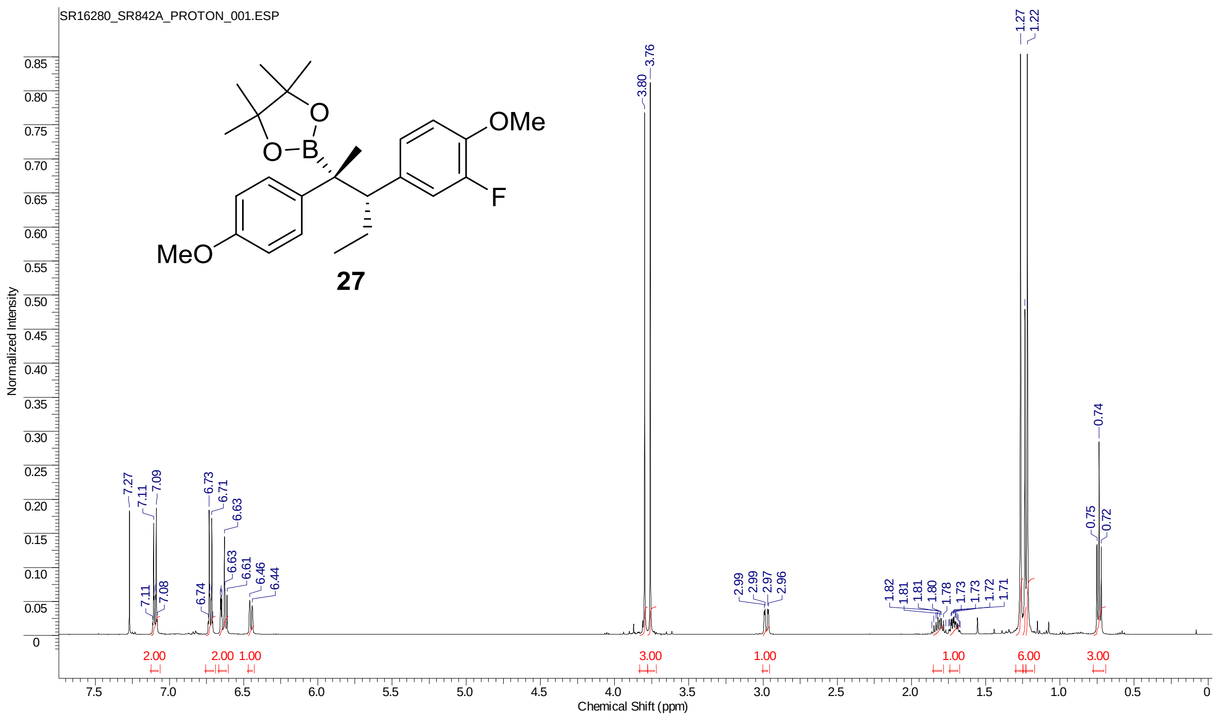

^{13}C NMR (126 MHz, $CDCl_3$)

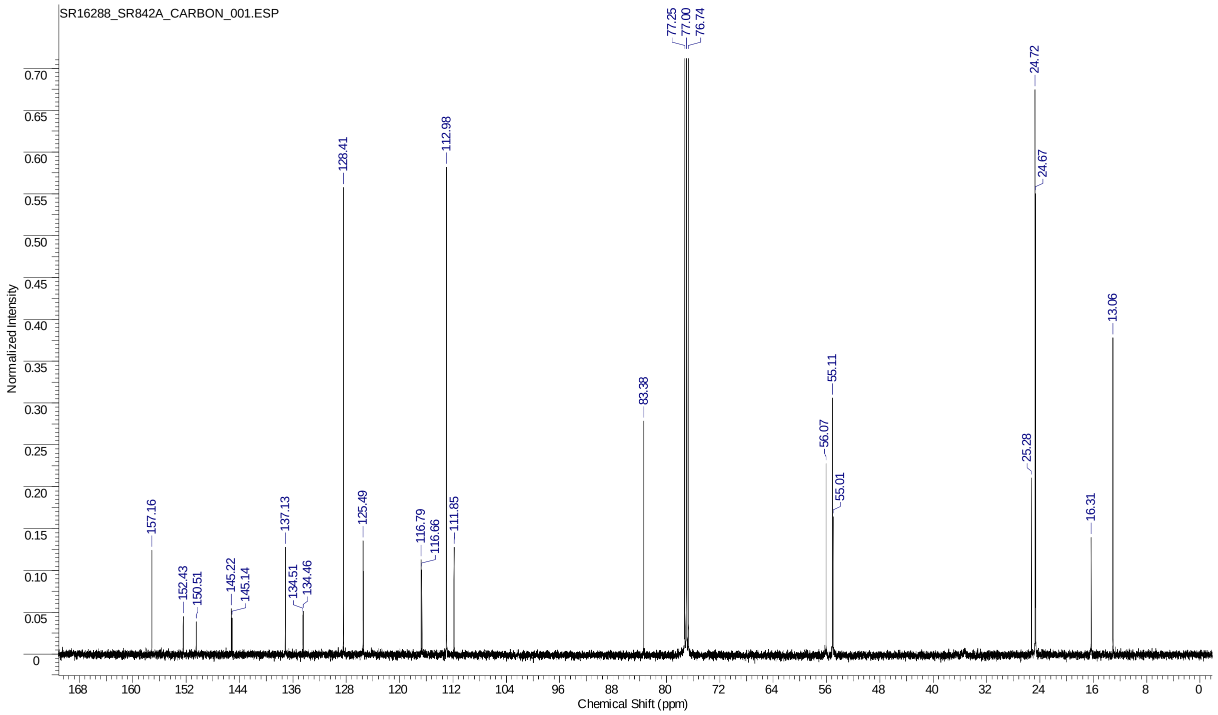

2-Fluoro-1-methoxy-4-((2*S*,3*R*)-2-(4-methoxyphenyl)pentan-3-yl)benzene (34)

^{1}H NMR (400 MHz, $CDCl_3$)

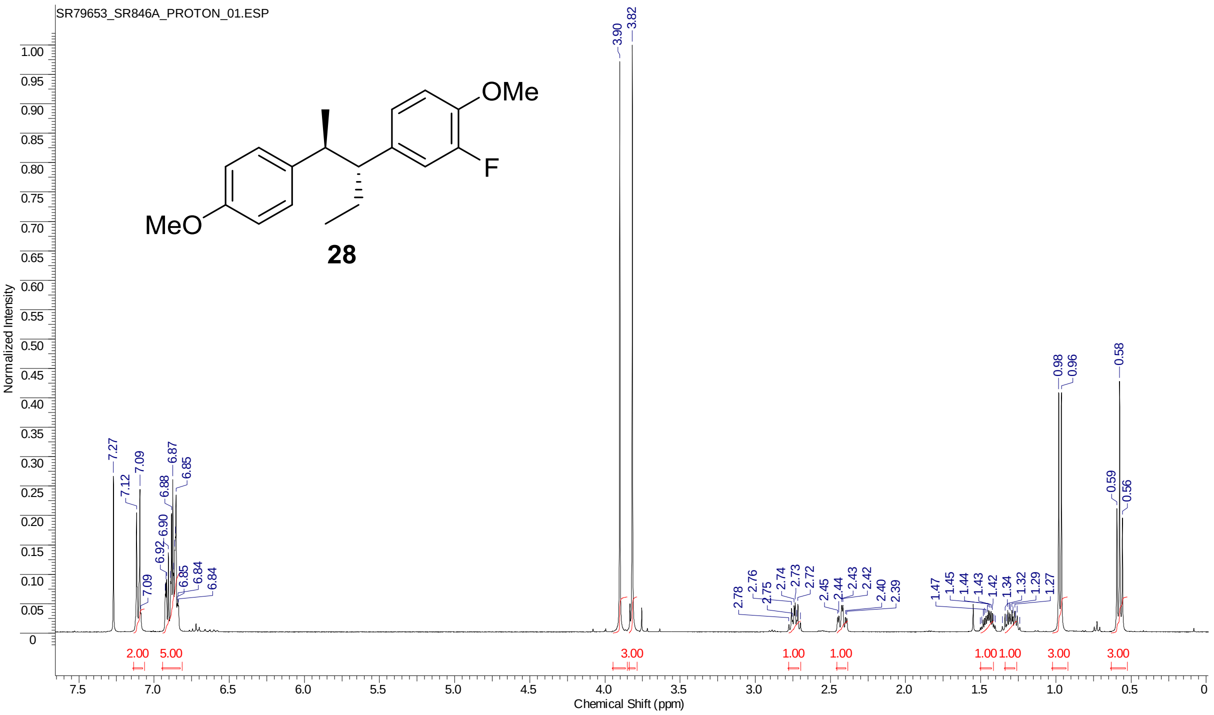

^{13}C NMR (126 MHz, $CDCl_3$)

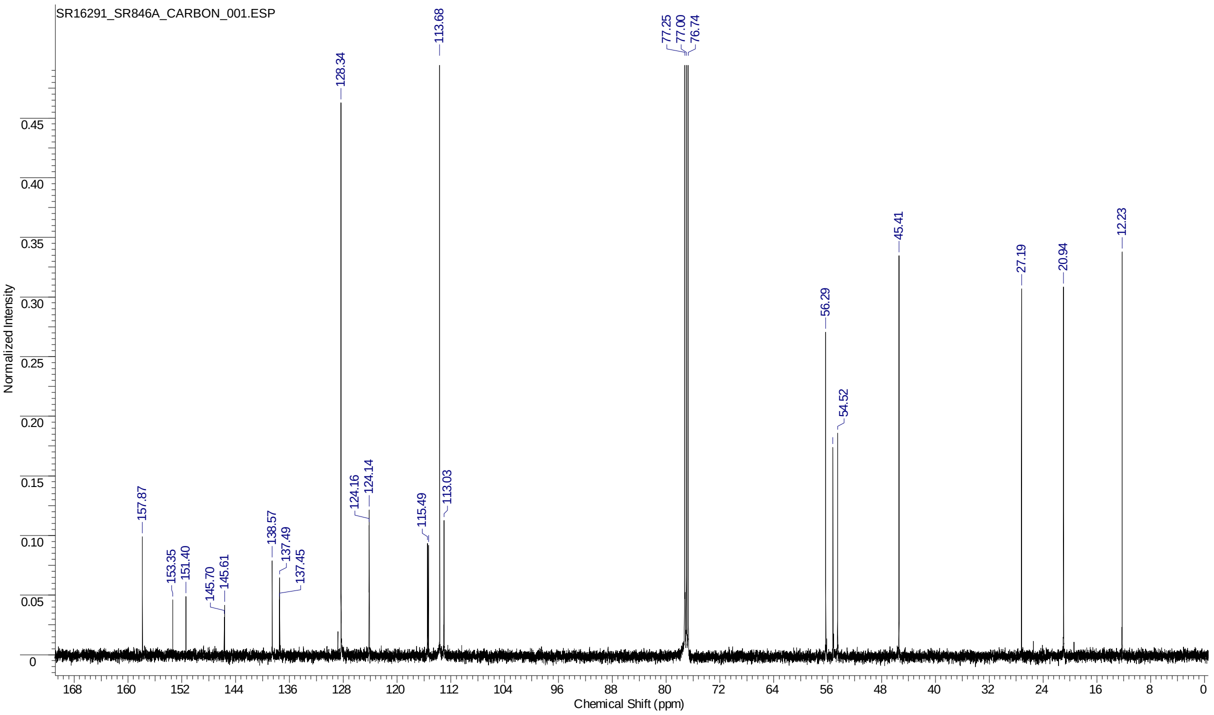

2-Bromo-4-((2*S*,3*R*)-3-(3-fluoro-4-methoxyphenyl)pentan-2-yl)-1-methoxybenzene (35)

^{1}H NMR (500 MHz, $CDCl_3$)

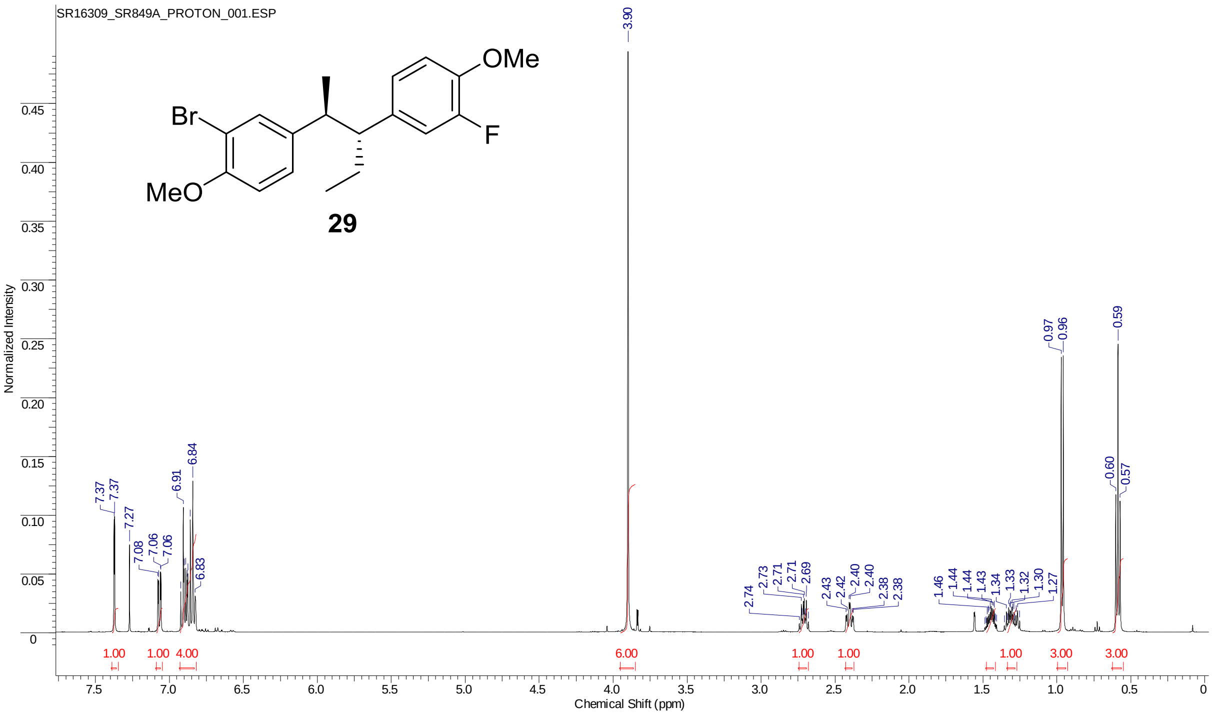

^{13}C NMR (126 MHz, $CDCl_3$)

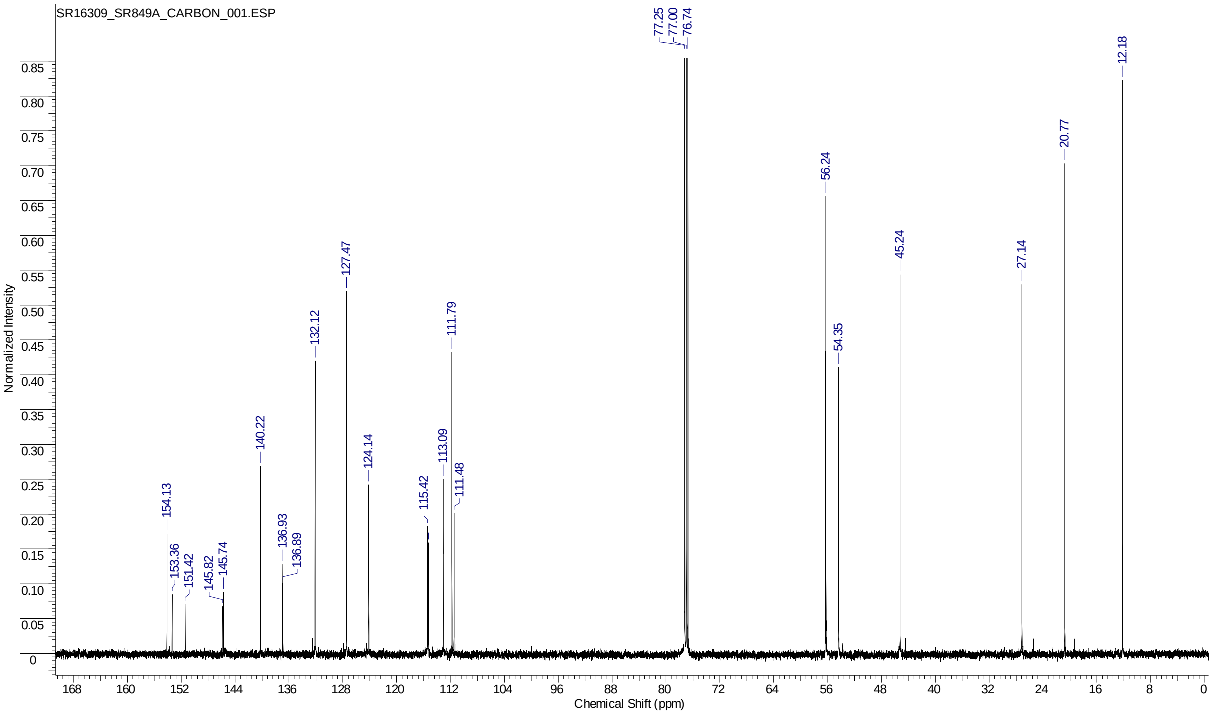

4,4'-((2*S*,3*R*)-Pentane-2,3-diyl)bis(2-fluorophenol), Bifluranol (1)

^{1}H NMR (500 MHz, acetone-d6)

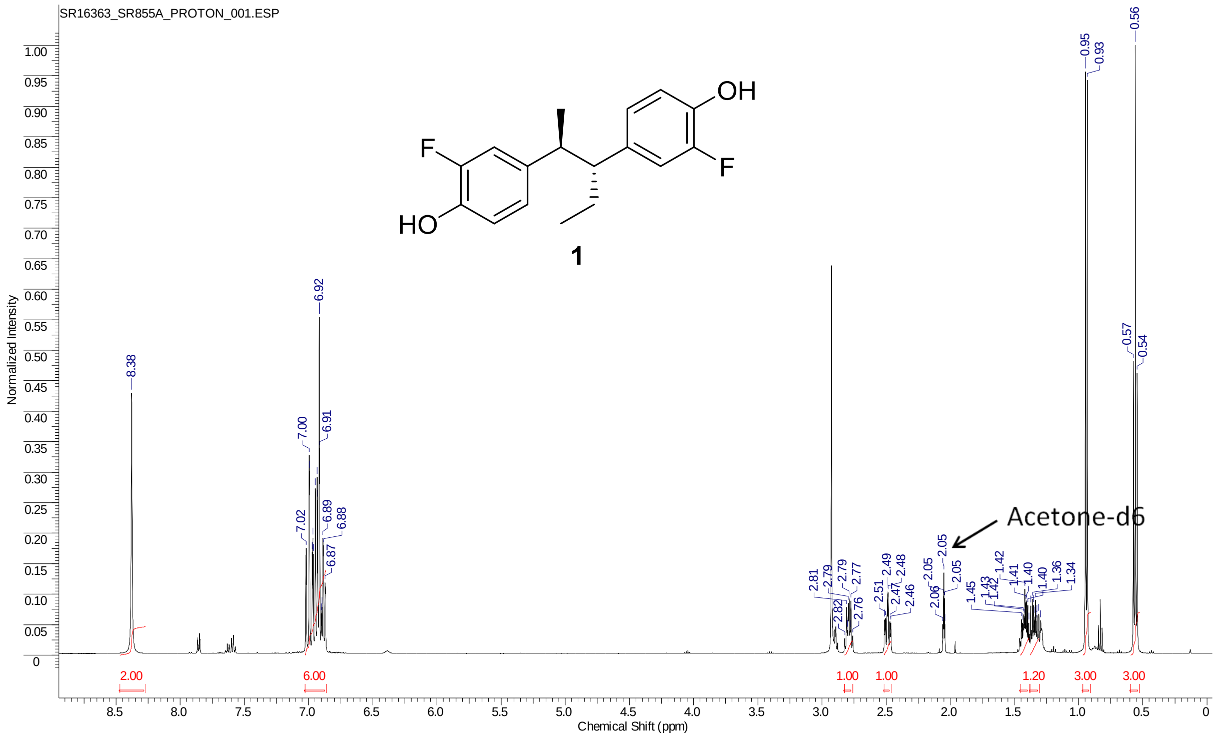

^{13}C NMR (126 MHz, acetone-d6)

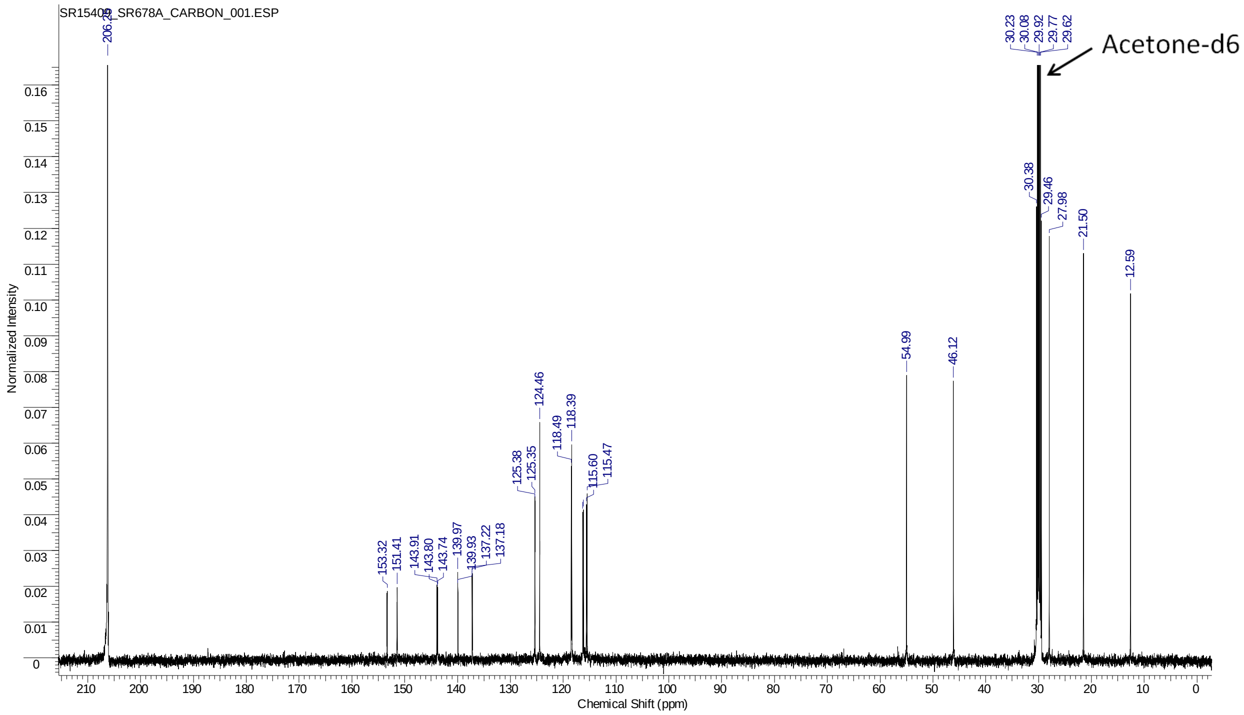

2-((3R,4S)-4-(3-fluoro-4-methoxyphenyl)-3-(4-methoxyphenyl)hexan-3-yl)-5,5-dimethyl-1,3,2-dioxaborinane (3*R*,4*S*)-41

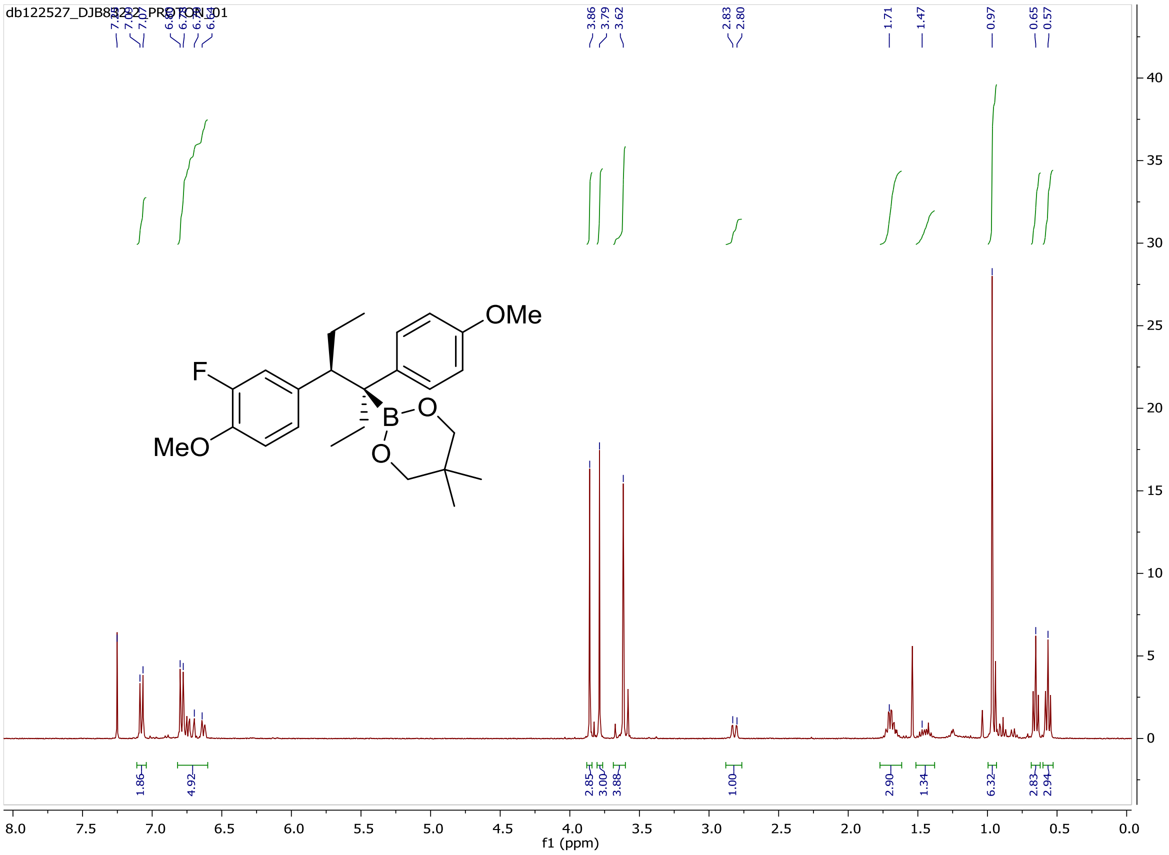

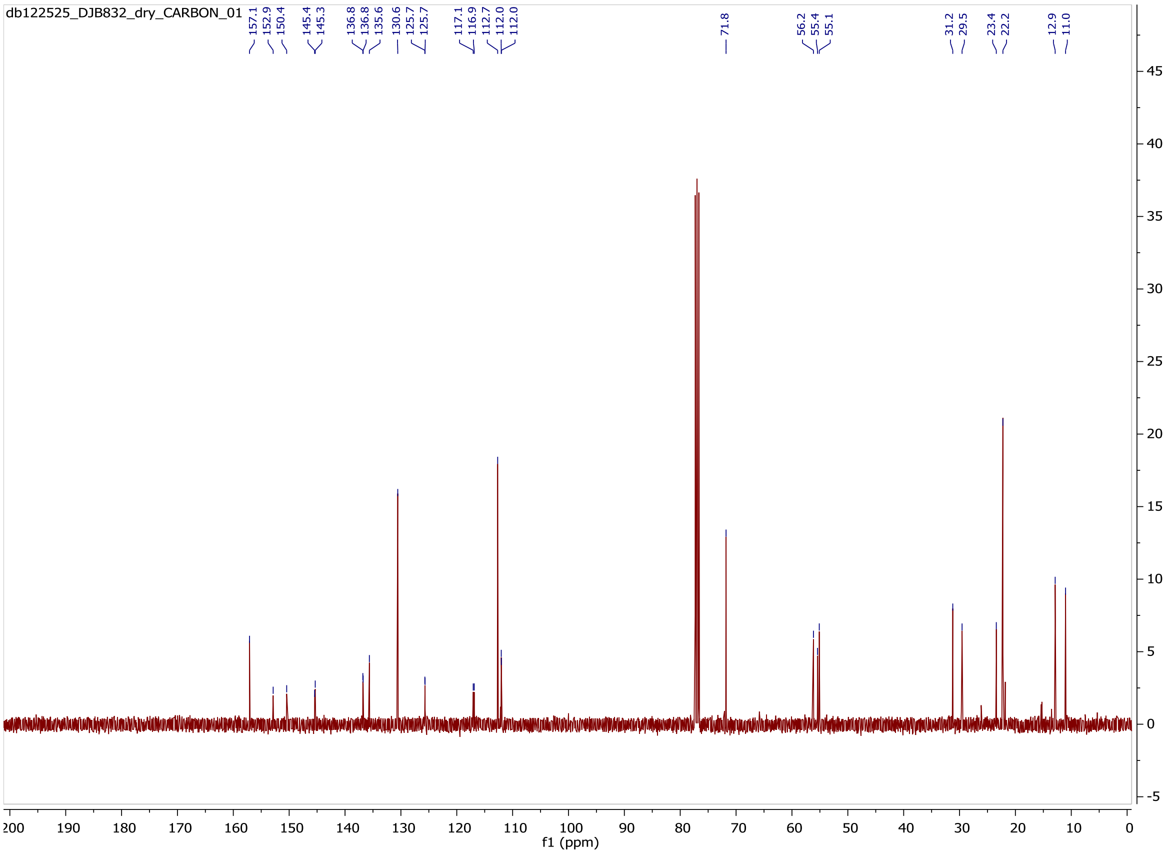

2-Fluoro-1-methoxy-4-((3*S*,4*R*)-4-(4-methoxyphenyl)hexan-3-yl)benzene (42)

^{1}H NMR (400 MHz, $CDCl_3$)

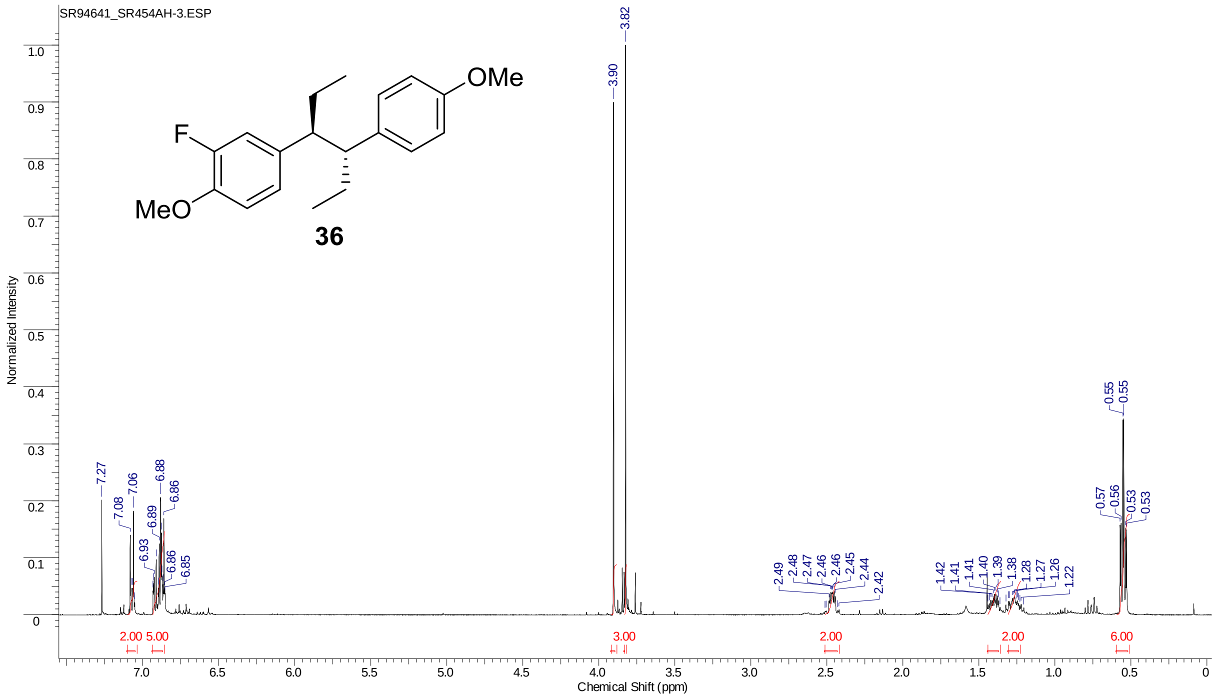

^{13}C NMR (126 MHz, $CDCl_3$)

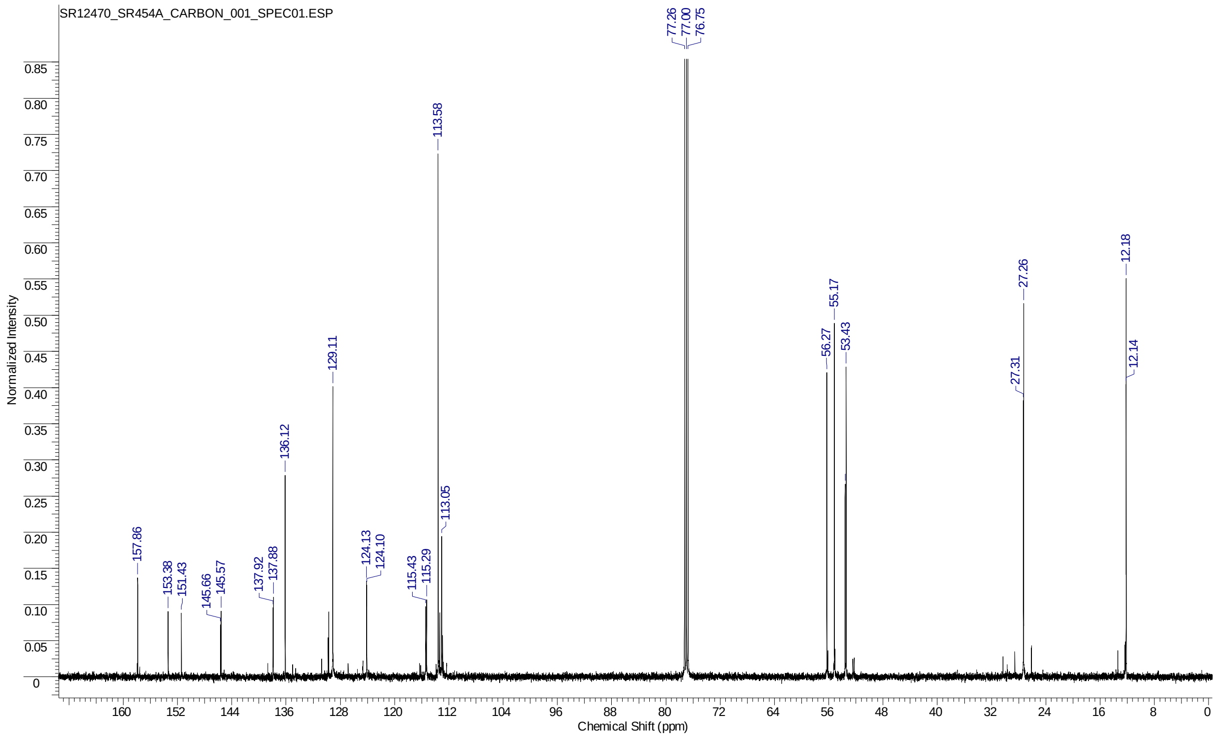

2-Fluoro-4-((2*S*,3*R*)-3-(4-hydroxyphenyl)pentan-2-yl)phenol, Fluorohexestrol (2)

^{1}H NMR (500 MHz, acetone-d6)

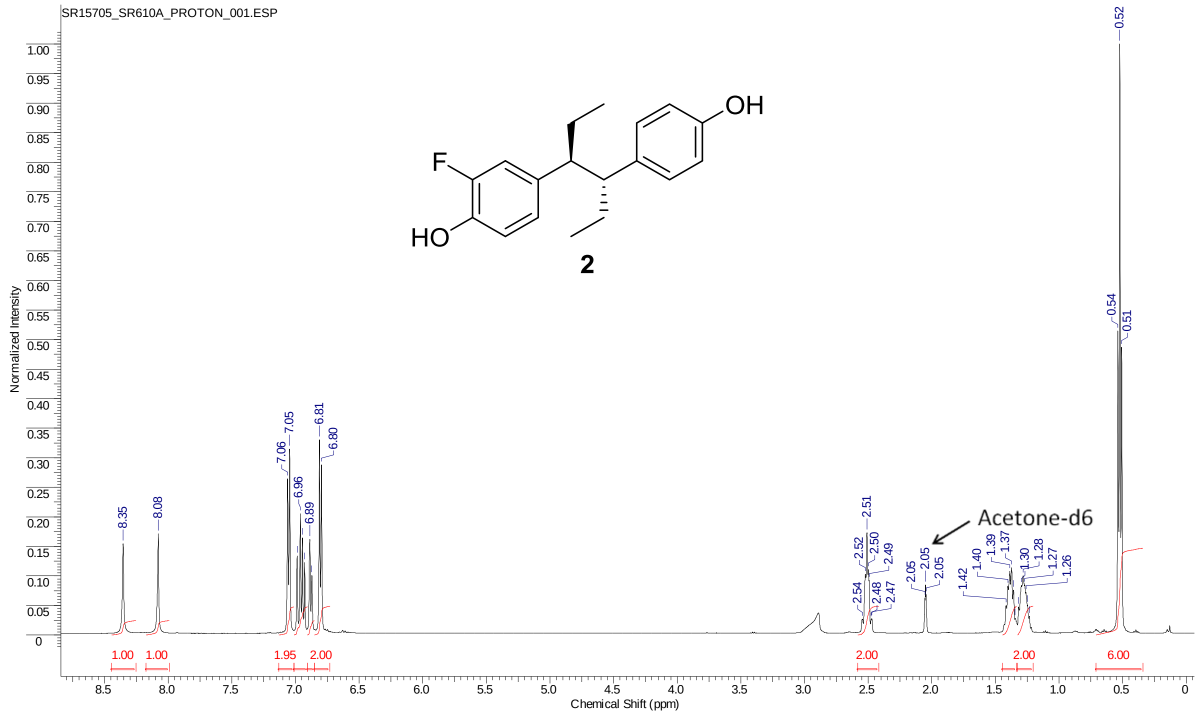

^{13}C NMR (101 MHz, acetone-d6)

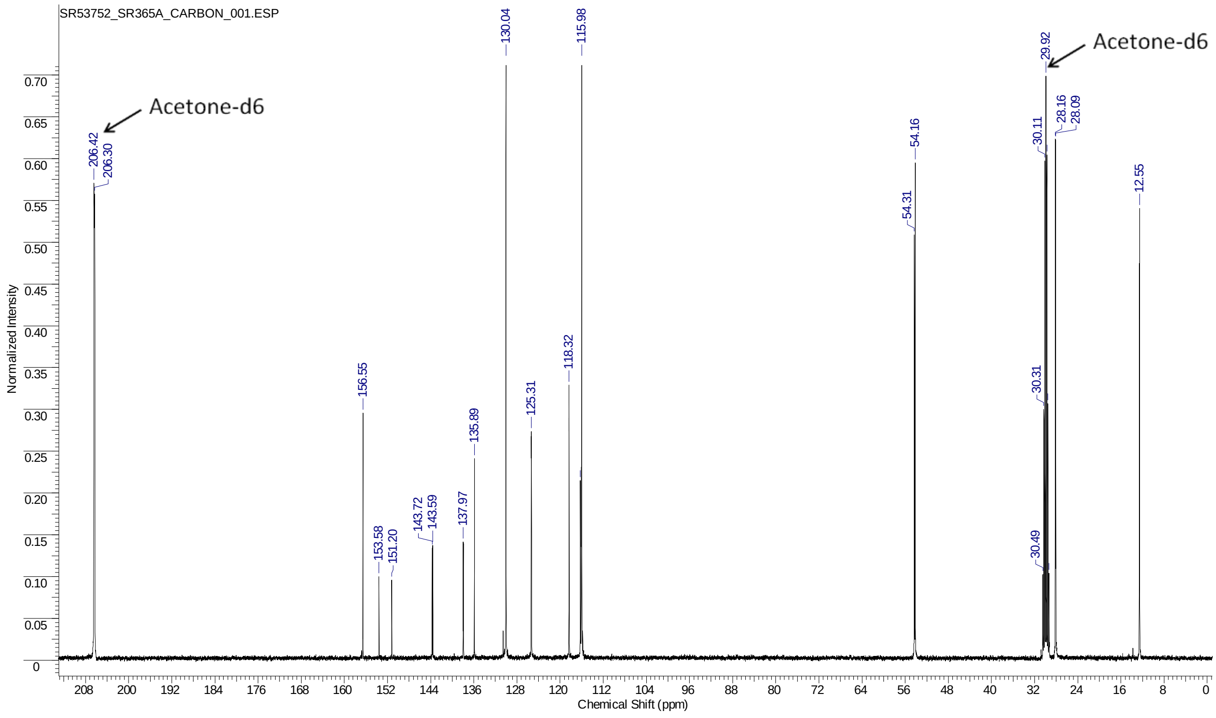

2-Fluoro-4-((3*S*,4*S*)-4-(4-hydroxyphenyl)hexan-3-yl)phenol (47)

^{1}H NMR (400 MHz, acetone-d6)

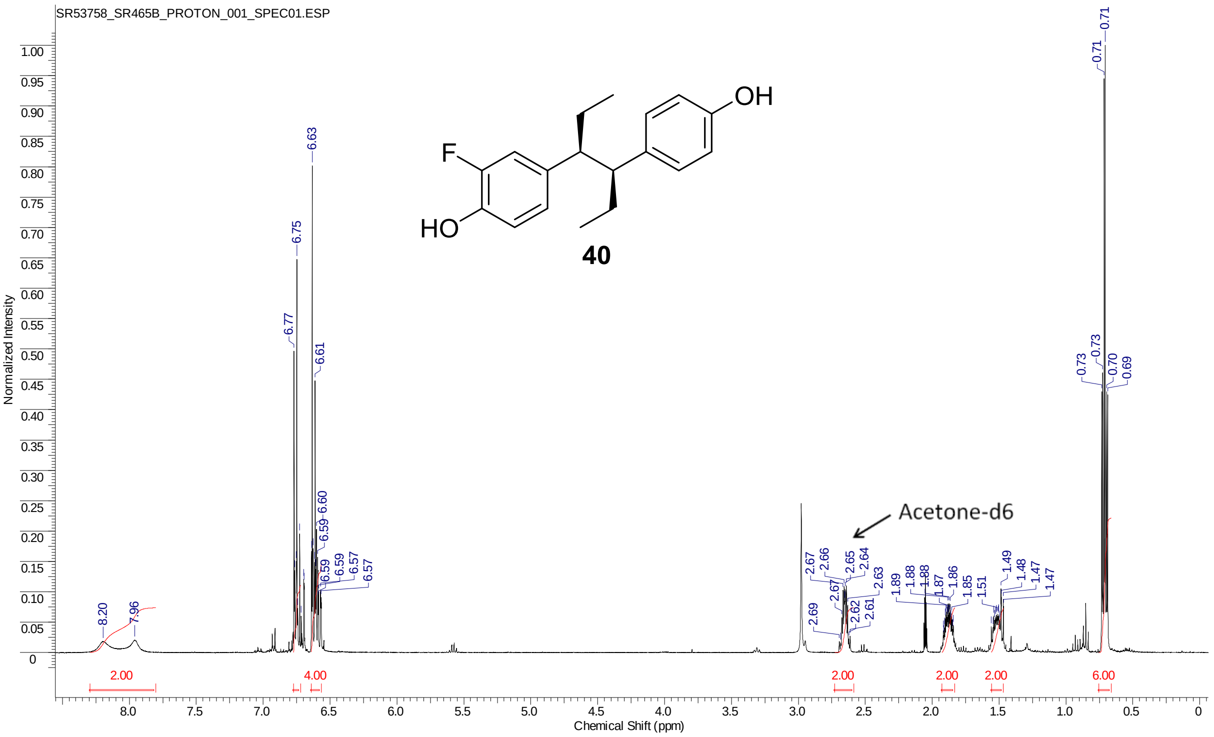

^{13}C NMR (126 MHz, acetone-d6)

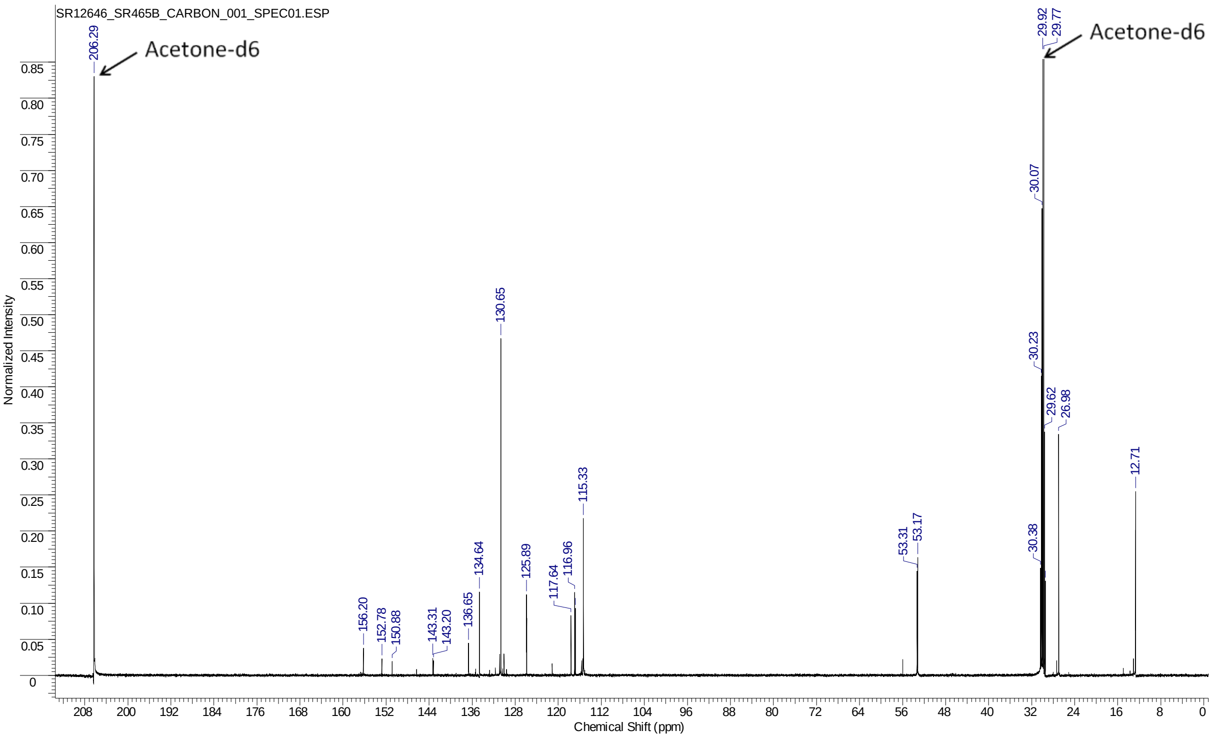

5. Determination of diastereomeric ratio of tertiary boronic esters *via* ^{1}H NMR

2-(2,3-Diphenylbutan-2-yl)-4,4,5,5-tetramethyl-1,3,2-dioxaborolane (15)

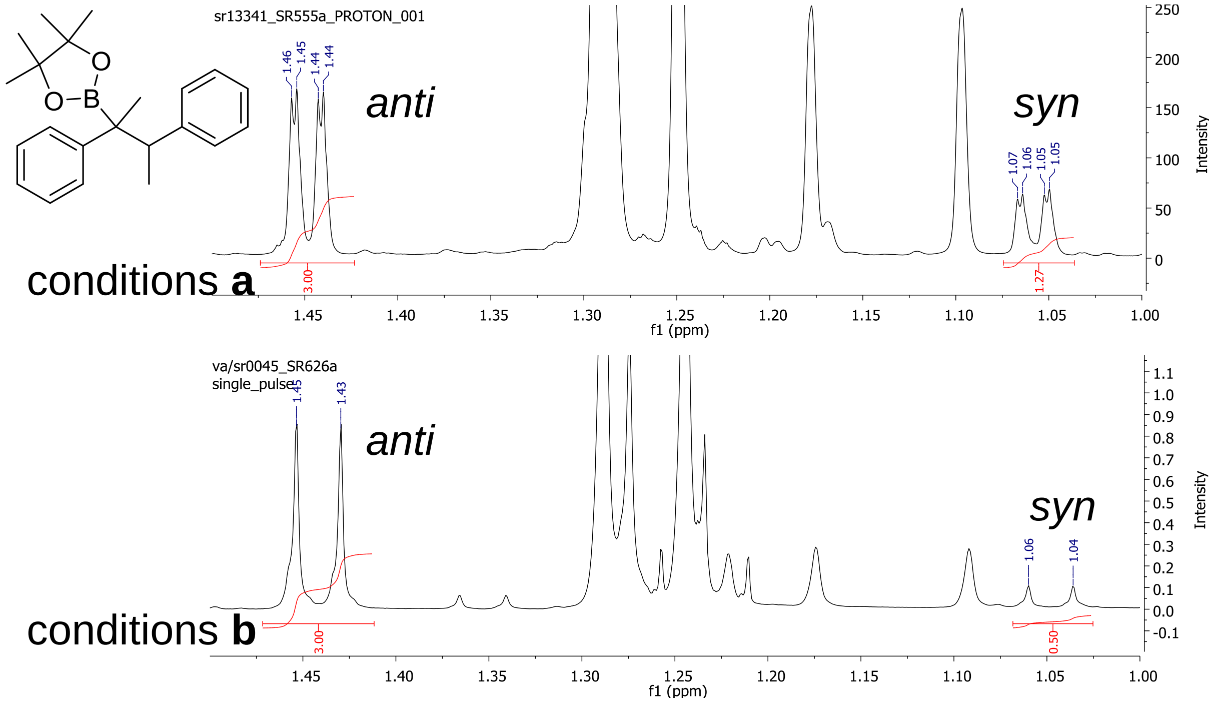

2-(2,3-Diphenylpentan-3-yl)-4,4,5,5-tetramethyl-1,3,2-dioxaborolane (16)

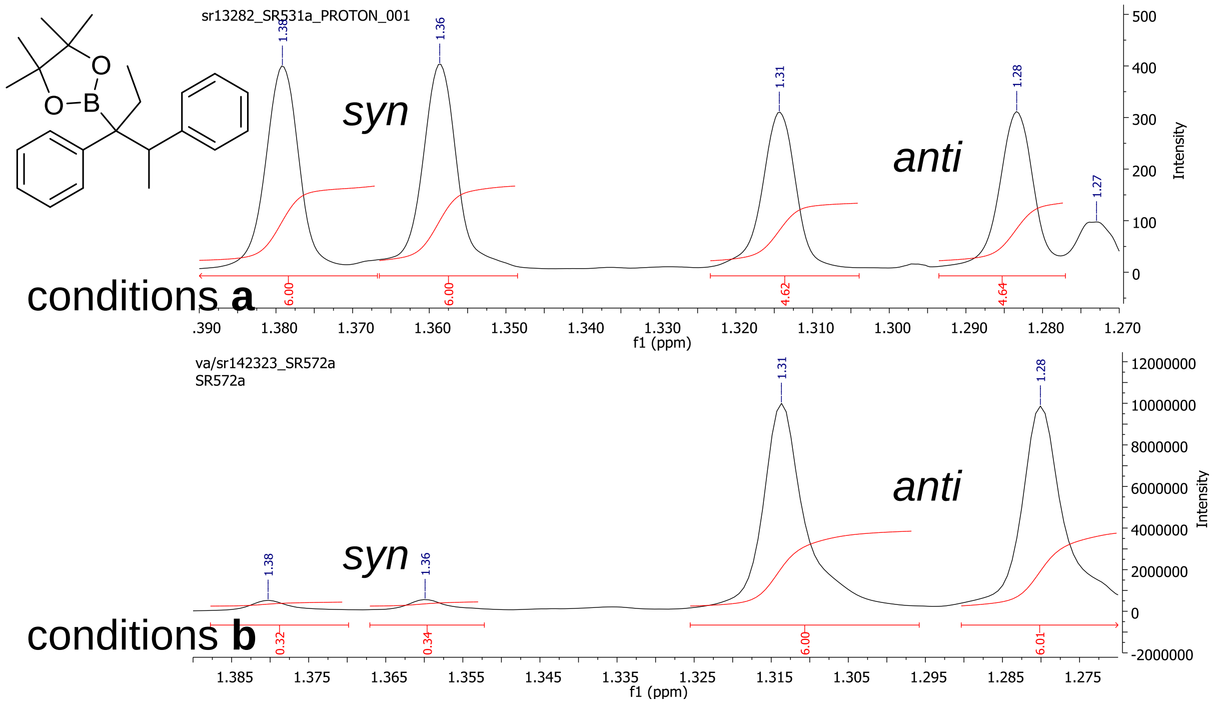

2-(2,3-Diphenylpentan-2-yl)-4,4,5,5-tetramethyl-1,3,2-dioxaborolane (17)

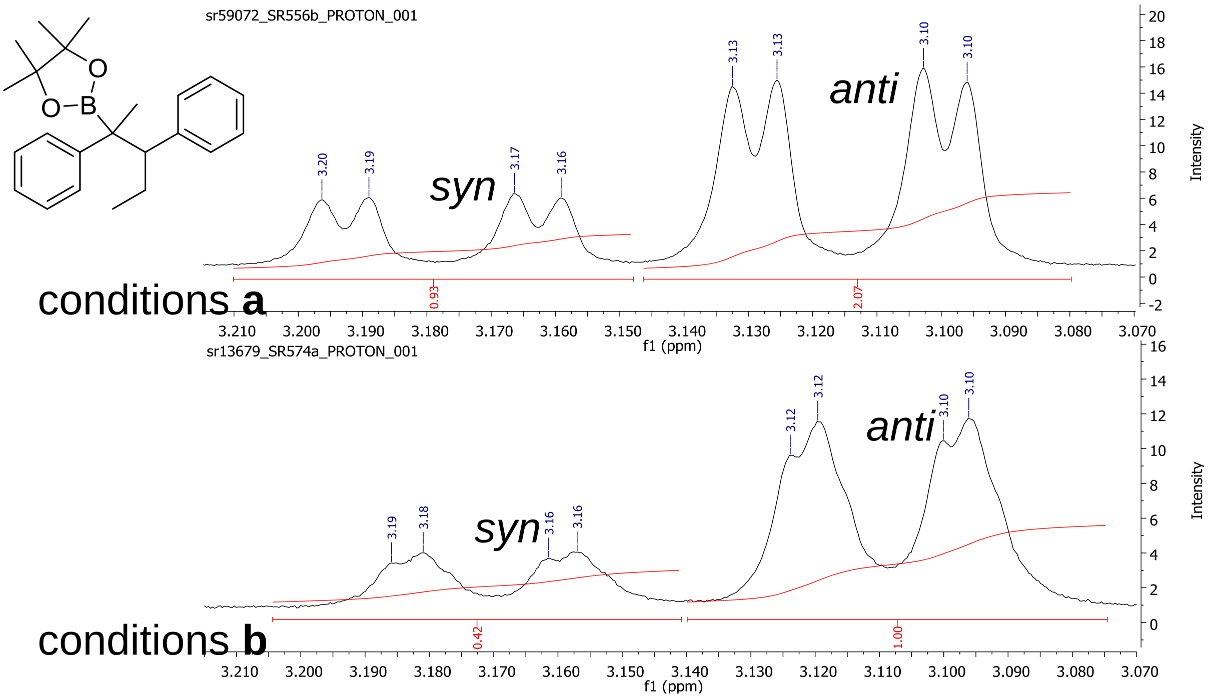

2-(3,4-Diphenylhexan-3-yl)-4,4,5,5-tetramethyl-1,3,2-dioxaborolane (18)

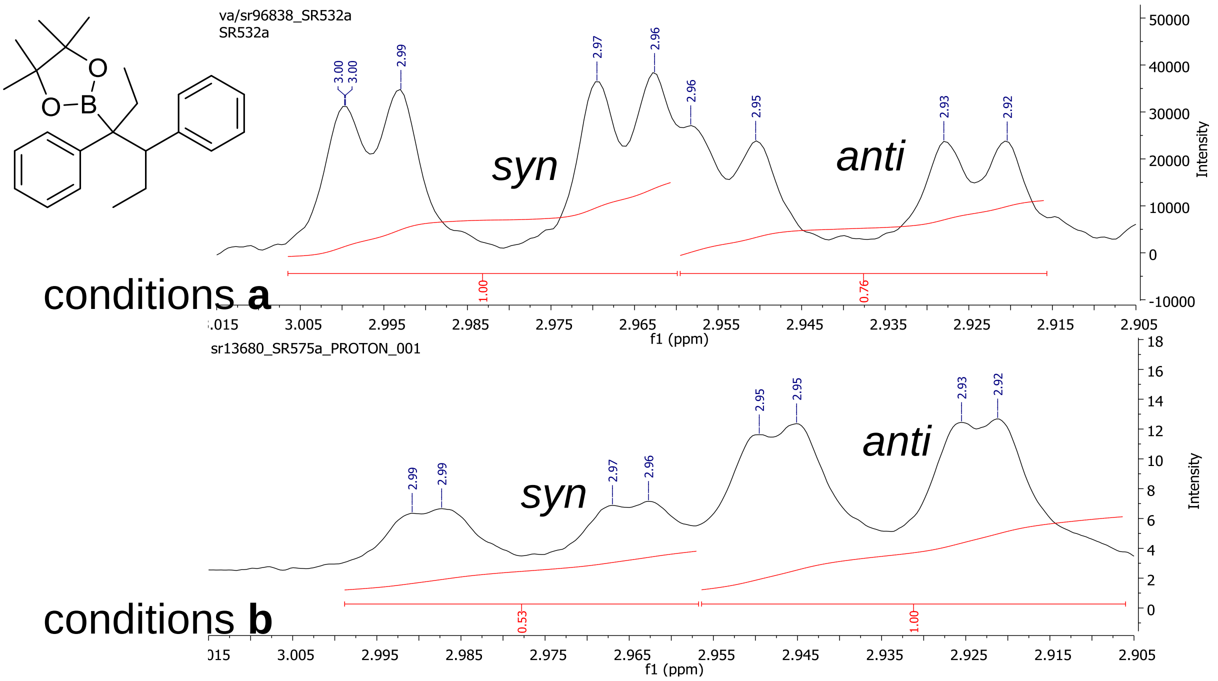

6. References

(1) Burchat, A. F.; Chong, J. M.; Nielsen, N. *J. Organomet. Chem.* **1997**, *542*, 281–283.

(2) Nicolic, N. A.; Beak, P. *Org. Synth.* **1997**, *74*, 23–26.

(3) Pangborn, A. B.; Giardello, M. A.; Grubbs, R. H.; Rosen, R. K.; Timmers, F. J. *Organometallics* **1996**, *15*, 1518–1520.

(4) Ou, L.; Ludwig, D.; Pan, J.; Xu, J. H. *Org. Process Res. Dev.* **2008**, *12*, 192–195.

(5) MacLellan, P.; Clayden, J. *Chem. Commun.* **2011**, *47*, 3395–3397.

(6) Dean, M. A.; Hitchcock, S. R. *Tetrahedron: Asymmetry* **2008**, *19*, 2563–2567.

(7) Alonso, E.; Ramón, D. J.; Yus, M. *Tetrahedron* **1996**, *52*, 14341–14348.

(8) Bagutski, V.; Ros, A.; Aggarwal, V. K. *Tetrahedron* **2009**, *65*, 9956–9960.

(9) Carstens, A.; Hoppe, D. *Tetrahedron* **1994**, *50*, 6097–6108.

(10) Alonso, E.; Guijarro, D.; Martínez, P.; Ramón, D. J.; Yus, M. *Tetrahedron* **1999**, *55*, 11027–11038.

(11) Bagutski, V.; French, R. M.; Aggarwal, V. K. *Angew. Chem. Int. Ed.* **2010**, *49*, 5142–5145.

(12) Stymiest, J. L.; Bagutski, V.; French, R. M.; Aggarwal, V. K. *Nature* **2008**, *456*, 778–782.

(13) Roush, W.; Walts, A. E.; Hoong, L. K. *J. Am. Chem. Soc.* **1985**, *107*, 8186–8190.

(14) Roesner, S.; Brown, C. A.; Mohiti, M.; Pulis, A. P.; Rasappan, R.; Blair, D. J.; Essafi, S.; Leonori, D.; Aggarwal, V. K. *Chem. Commun.* **2014**, *50*, 4053–4055.

(15) Chen, A.; Ren, L.; Crudden, C. M. *J. Org. Chem.* **1999**, *64*, 9704–9710.

(16) Endo, K.; Hirokami, M.; Takeuchi, K.; Shibata, T. *Synlett* **2008**, 3221–3233.

(17) Beak, P.; Carter, L. G. *J. Org. Chem.* **1981**, *46*, 2363–2373.

(18) Lata, C. J.; Crudden, C. M. *J. Am. Chem. Soc.* **2010**, *132*, 131–137.

(19) Stymiest, J. L.; Dutheuil, G.; Mahmood, A.; Aggarwal, V. K. *Angew. Chem. Int. Ed.* **2007**, *46*, 7491–7494.

(20) Hesse, M. J.; Butts, C. P.; Willis, C. L.; Aggarwal, V. K. *Angew. Chem. Int. Ed.* **2012**, *51*, 12444–12448.

(21) Heiman, D. F.; Senderoff, S. G.; Katzenellenbogen; J. A., Neeley, R. J. *J. Med. Chem.* **1980**, *23*, 994–1002.

(22) Hebel, D.; Lerman, O.; Rozen, S. *Bull. Soc. Chim. Fr.* **1986**, *6*, 861–863.

(23) Wymann, W. E.; Davis, R.; Patterson, J. W.; Pfister, J. R. *Synth. Commun.* **1988**, *18*, 1379–1384.
